# The Diels-Alder Cycloaddition Reaction of Substituted Hemifullerenes with 1,3-Butadiene: Effect of Electron-Donating and Electron-Withdrawing Substituents

**DOI:** 10.3390/molecules21020200

**Published:** 2016-02-12

**Authors:** Martha Mojica, Francisco Méndez, Julio A. Alonso

**Affiliations:** 1 Departamento de Química, División de Ciencias Básicas e Ingeniería, Universidad Autónoma Metropolitana-Iztapalapa, 09340 México D. F., Mexico; fm@xanum.uam.mx; 2 Departamento de Física Teórica, Atómica y Óptica, Universidad de Valladolid, 47011 Valladolid, Spain; jaalonso@fta.uva.es

**Keywords:** Diels-Alder, DFT, polycyclic aromatic hydrocarbons (PAHs)

## Abstract

The Diels-Alder (DA) reaction provides an attractive route to increase the number of six member rings in substituted Polycyclic Aromatic Hydrocarbons (PAHs). The density functional theory (DFT) B3LYP method has been used in this work to inquire if the substitution of H over the edge of triindenetriphenylene (pristine hemifullerene **1**) and pentacyclopentacorannulene (pristine hemifullerene **2**), could improve the DA cycloaddition reaction with 1,3-butadiene. The substituents tested include electron-donating (NH_2_, OMe, OH, Me, *i*-Pr) and electron-withdrawing groups (F, COOH, CF_3_, CHO, CN, NO_2_). The electronic, kinetic and thermodynamic parameters of the DA reactions of the substituted hemifullerenes with 1,3-butadiene have been analyzed. The most promising results were obtained for the NO_2_ substituent; the activation energy barriers for reactions using this substituent were lower than the barriers for the pristine hemifullerenes. This leads us to expect that the cycloadditions to a starting fullerene fragment will be possible.

## 1. Introduction

The Diels-Alder (DA) cycloaddition reaction is one of the most widely used methodologies in organic synthesis nowadays [1,2,3]. It has been used to “grow” polycyclic aromatic hydrocarbons (PAH) by increasing the number of six-membered rings [4]; for example, in the synthesis of substituted coronenes by the stepwise DA reaction of perylene with maleic anhydride [5] or maleimide [6], and the synthesis of a variety of size- and shape-controlled peryacenes by the DA cycloaddition of bisanthene with several arynes [7] and substituted quinones [8]. Fort and coworkers [9] proposed the application of the DA reaction for metal-free growth of single-walled carbon nanotubes (SWNTs). The highly exothermic aromatization of each newly formed six-membered ring by loss of two hydrogen atoms would be expected to occur spontaneously even at moderate temperatures and in the absence of oxidizing agents. These authors have demonstrated that the DA reaction of diethyl acetylenedicarboxylate with the bay regions of 4,11-dimesitylbisanthene (a polycyclic aromatic hydrocarbon) occurs at 120 °C. Fort and Scott [10] have also proved that the use of nitroethylene as a masked-acetylene in a DA reaction with the bay regions of perylene or 7,14-dimesitylbisanthene leads to a rapid conversion of the bay regions of the PAH into new unsubstituted benzene rings.

**Figure 1:**
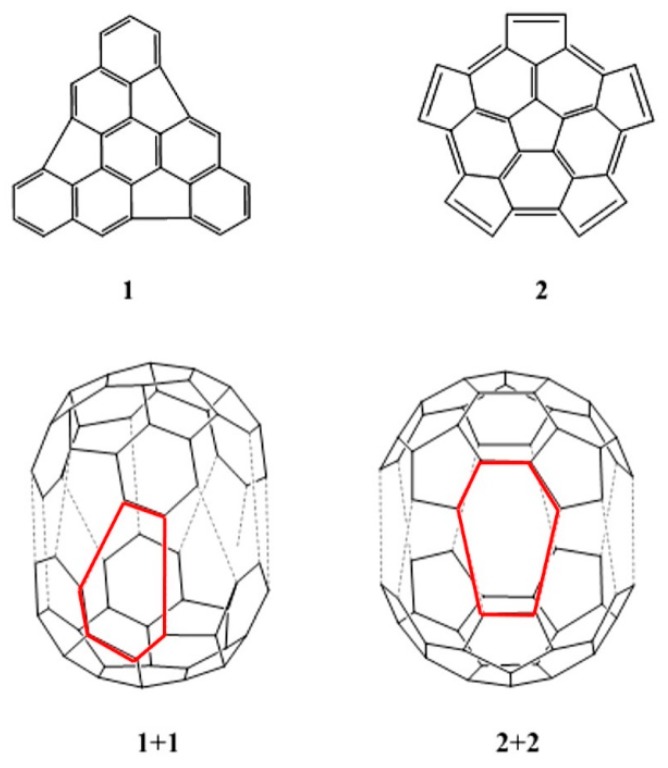
Triindenetriphenylene (fragment **1**) and pentacyclopentacorannulene (fragment **2**), and the approaching of two triindenetriphenylene fragments, **1+1**, and two pentacyclopentacorannulene fragments, **2+2**, to form C_60_.

The DA cycloaddityion reactions have also been proposed as a way to synthesized C_60_ by a dimerization strategy that involves the formation of new 6 member rings (Figure 1). Sygula and Rabideau [11] suggested the dimerization of two identical hemispherical C_30_ fragments as an intriguing possibility to synthesize C_60_, and mentioned that this dimerization would take place via a series of DA cycloaddition reactions. Scott [12] emphasized that this particularly appealing strategy for assembling carbon fullerenes resemble the hypothetical synthesis of dodecahedrane by the dimerization of triquinacene proposed by Woodward, Fukunaga and Kelly [13] (see also refs. [14,15,16]).

**Figure 2:**
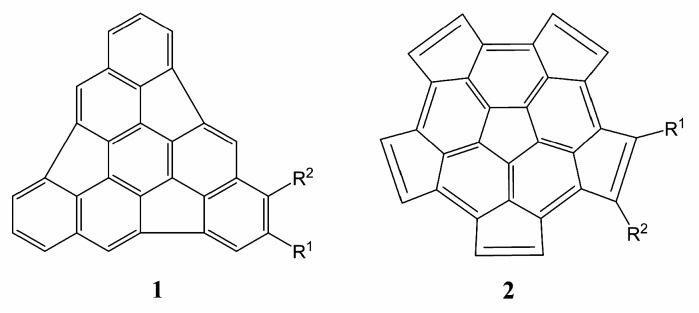
Substituted triindenetriphenylene (**1**) and pentacyclopentacorannulene (**2**). R^1^ and R^2^ indicate the substituents. Two cases have been studied: (a) R^1^ = R^2^ = NH_2_, OMe, OH, Me (methyl), *i*-Pr (iso-propyl), H, F, COOH, CF_3_, CHO, CN, NO_2_; (b) R^1^ = NH_2_, OMe, OH, Me, *i*-Pr, H, F, COOH, CF_3_, CHO, CN, NO_2_, R^2^ = H.

In order to evaluate the possibility of stitching two hemifullerenes together using a DA reaction mechanism, Mojica *et al.* [17] performed a theoretical study of the DA reactions between fragments **1** and **2** with ethylene and butadiene molecules to obtain new six-membered carbon rings on the rims of the two fragments. The study supported the feasibility of a systematic method to obtain fullerenes by DA reactions with a starting fragment, and also suggested the possibility of assembling fullerenes by DA dimerization reactions of fragments **1** and **2**. The DA reactions of 1,3-butadiene with the rim of fragments **1** and **2** have activation energy values of 33.1 kcal/mol and 22.6 kcal/mol, respectively, close to the activation energy (24.8 kcal/mol) for the DA reaction of 1,3-butadiene with ethylene [17]. The DA reactions of 1,3-butadiene with fragments **1** and **2** are normal electron-demand DA reactions: 1,3-butadiene is the diene and the fragments act as dienophiles. Therefore, the promotion of the DA reactions of the fragments to the status of a general synthetic method needs the development of ways to increase their activity with a selected diene. Therefore, we consider that the key to realizing the potential of the fragments **1** and **2** should be increasing their electron-poor nature to reduce the activation energies of those reactions. Plater and coworkers stated that the dimerization process should be metal catalyzed, and their calculations predicted a reduction of the enthalpy of formation (Δ_f_H°) in some complexes of the type **1+1**–M^+^ → C_60_–M^+^ [18]. However, the effects of adding electron-withdrawing substituents on the hydrocarbon moiety of the hemifullerenes to favor the dimerization reaction have not been studied. In this work we explore the effect of electron-withdrawing and electron-releasing substituents on the fragments **1** and **2** (Figure 2), investigating the DA reactions of the substituted fragments with 1,3-butadiene. The effect of different substituents on the kinetic and thermodynamic parameters characterizing the DA reaction is analyzed, as well as the aromatization of the obtained adducts.

## 2. Results and Discussion

Hydrogen atoms of the fragments **1** and **2** were substituted with electron-withdrawing (F, COOH, CF_3_, CHO, CN, NO_2_) and electron-donating (NH_2_, OH, methyl (Me), OMe, *iso*-propyl (*i*-Pr)) groups (Figure 2). Even when only the electron-withdrawing groups produce the desired electronic effect on the fragments, the analysis of the electron-donating substitution should help improve our understanding of the behavior of the fragments. Two options were considered for the substitutions on each fragment: (a) in order to maximize the electronic effect induced by the substituents, two equal substituents were arranged, one on each side of the dienophilic double bond (R^1^ = R^2^ in Figure 2); and (b) only one substituent group (R^1^) was considered (that is, R^2^ = H), in order to avoid or reduce the steric hindrance due the presence of big substituents. Hemifullerenes can be substituted making use of the same procedures employed in the functionalization of carbon nanotubes [19], since hemifullerenes can be considered as ultra-short, one side capped single wall carbon nanotubes. Also, substituted hemifullerenes can be synthetized in a bottom up manner from substituted reactants, an approach followed by Scott [20] and Sygula [21].

### 2.1. Frontier Molecular Orbitals

The DA reaction can be interpreted in terms of the Frontier Molecular Orbitals (FMO) theory as the interaction between the highest occupied molecular orbital (HOMO) of one of the molecules and the lowest unoccupied molecular orbital (LUMO) of the other molecule. When the interaction occurs between the HOMO of the diene and the LUMO of the dienophile, the reaction is called Normal Electron Demand Diels-Alder (NEDDA) reaction, and when the interaction takes place between the HOMO of the dienophile and the LUMO of the diene, the reaction is called Inverse Electron Demand Diels-Alder (IEDDA) reaction [22]. The presence of electron-withdrawing or electron-donating groups in the dienophile should favor the NEDDA and IEDDA reactions, respectively.

**Figure 3:**
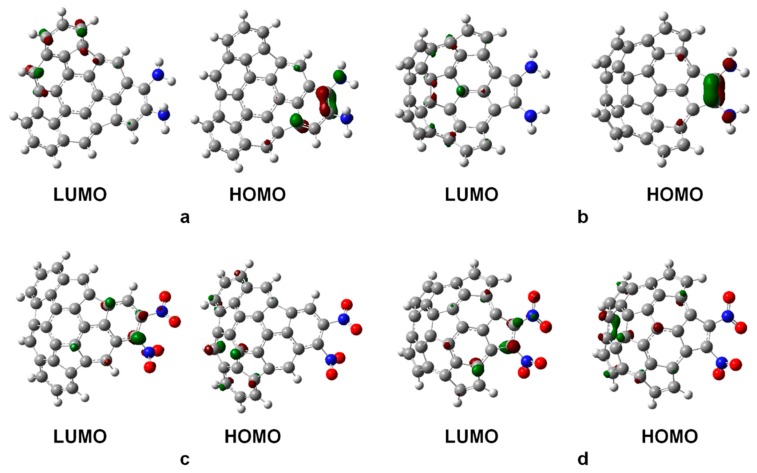
Frontier molecular orbitals HOMO and LUMO of fragments **1** and **2** substituted with two NH_2_ molecules (panels **a** and **b**), or two NO_2_ molecules (panels **c** and **d**). The substituent molecules are on the right side of the fragments. The large grey, blue and red spheres represent carbon, nitrogen and oxygen atoms, respectively, and hydrogen atoms are represented by the small grey spheres. The dark red and green surfaces represent the positive and negative lobes of the calculated molecular orbitals respectively, plotted at 0.08 au.

We first consider the substitution of two H atoms by two electron-withdrawing or two electron-donating groups. The HOMO and LUMO orbitals were obtained from the canonical orbitals calculated at the HF/6-31G(d,p)//B3LYP/6-31G(d,p) level of theory. That is, the geometries were optimized at the B3LYP/6-31G(d,p) level and then a calculation at the HF/6-31G(d,p) level was performed to obtain the orbital energies. Figure 3 shows the HOMO and LUMO orbitals of fragments **1** and **2** substituted with two NH_2_ or NO_2_ groups. Results for all the substituents of the list are collected in Figures S1 to S4. In most cases of substitution with electron-donating groups (for instance, NH_2_ in Figure 3), the HOMO orbitals of the substituted fragments are localized in regions around the atoms involved in the reaction. Similarly, for substitution with electron-withdrawing groups (for instance, NO_2_ in Figure 3), the LUMO orbitals of the substituted fragments are in most cases localized around the atoms involved in the reaction. No substitution of the two original H atoms, and substitution by two F atoms, appear to be limiting cases separating the electron-withdrawing and electron-donating groups, and substantial delocalization of the HOMO and LUMO orbitals is observed. The LUMO orbitals of the non-substituted fragments are mostly over the rim of the fragments, where the reactions should take place.

For substitution of a single H atom, the qualitative shapes of the HOMO and LUMO orbitals are similar to those for double substitution, but those orbitals are in all cases less localized around the atoms involved in the reaction (Figures S3 and S4).

In the fragments substituted with electron-donating groups, the HOMO orbitals are mostly over carbon atoms neighbors to the substituents. In the case of substitution with electron-withdrawing groups, the LUMO orbitals are also over carbon atoms neighbors to the substituents. Those features suggest that fragments substituted with electron-donating groups would prefer IEDDA-type reactions. In contrast, fragments substituted with electron-withdrawing groups would prefer NEDDA-type reactions.

The energies of the HOMO and LUMO orbitals of the substituted fragments, as well as the HOMO-LUMO gaps for the NEDDA reactions, calculated as ΔE(NEDDA) = E(LUMO dienophile) − E(HOMO diene), and for the IEDDA reactions, calculated as ΔE(IEDDA) = E(LUMO diene) − E(HOMO dienophile), are shown in Table 1 and Table S2. The quantity δΔE = ΔE(IEDDA) − ΔE(NEDDA) indicates whether the NEDDA or the IEDDA reaction is favored: positive values of δΔE favor NEDDA reactions, and negative values favor IEDDA reactions. For comparison we include the values for the reactions with the non-substituted fragments (row for H in the middle of the Table).

Some relevant features can be observed form Table 1 and Table S2. In the reactions of butadiene with fragments substituted with electron-donating groups, the IEDDA gaps, ΔE(IEDDA), are smaller than the IEDDA gaps for reactions with non-substituted fragments. On the other hand, the IEDDA gaps for the reactions with fragments substituted with electron-withdrawing groups are larger than the IEDDA gaps for reactions with non-substituted fragments. NEDDA gaps, ΔE(NEDDA), show the opposite trend: the NEDDA gaps for the reactions with fragments substituted with electron-withdrawing groups are smaller than the NEDDA gaps for reactions with the non-substituted fragments, and the NEDDA gaps for the reactions with fragments substituted with electron-donating groups are, except in a couple of cases, larger than the NEDDA gaps for the reactions with non-substituted fragments. It can be noticed that δΔE is positive in general; that is, the NEDDA reaction is preferred. The only exception is the reaction with **1**-NH_2_ (double substitution). In fact, δΔE is smaller for the fragments substituted with electron-donating groups. The lowest reaction gaps are the NEDDA gaps for the fragments substituted with electron-withdrawing groups, and therefore, according to the FMO theory, those reactions should be easier than the other studied reactions. In the family of fragments substituted with electron-withdrawing groups, NEDDA gaps for double substitution are smaller. The substitution with NO_2_ leads to the smallest NEDDA gaps. The NEDDA gaps for the fragments substituted with electron-withdrawing groups are lower than the NEDDA gaps for the non-substituted fragments. Similarly, the IEDDA gaps for fragments substituted with electron-donating groups are lower than the IEDDA gaps for the non-substituted fragments. This purely electronic point of view opens up some expectations for the positive effect of substitution in enhancing the cycloaddition DA reactions of the fullerene fragments, and this is investigated in the next Sections of the paper.

**Table 1:** HOMO and LUMO energies (in eV) of the di-substituted (R^1^ = R^2^) and mono-substituted (R^2^ = H) fragment **2**, and HOMO-LUMO gaps (in eV) for the NEDDA and IEDDA reactions with 1,3-butadiene. ΔE(NEDDA) = E(LUMO dienophile) − E(HOMO diene) and ΔE(IEDDA) = E(LUMO diene) − E(HOMO dienophile). Also, δΔE = ΔE(IEDDA) − ΔE(NEDDA). Results for reaction with the non-substituted fragment (row for H in the middle of the Table) are included for comparison.

Substituent R^1^	E(HOMO)		E(LUMO)		ΔE(NEDDA)		ΔE(IEDDA)		δΔE	
	R^2^ = R^1^	R^2^ = H	R^2^ = R^1^	R^2^ = H	R^2^ = R^1^	R^2^ = H	R^2^ = R^1^	R^2^ = H	R^2^ = R^1^	R^2^ = H
NH_2_	−6.51	−6.78	0.84	0.72	9.62	9.49	9.96	10.23	0.35	0.73
OMe	−7.03	−7.06	0.66	0.62	9.43	9.39	10.48	10.51	1.05	1.12
OH	−6.97	−7.05	0.65	0.53	9.43	9.31	10.42	10.50	0.99	1.20
Me	−7.08	−7.10	0.72	0.68	9.49	9.45	10.53	10.55	1.04	1.10
*i*-Pr	−7.06	−7.09	0.70	0.68	9.48	9.46	10.51	10.53	1.03	1.08
H	−7.18	−7.18	0.67	0.67	9.45	9.45	10.63	10.63	1.18	1.18
F	−7.40	−7.26	0.37	0.48	9.15	9.25	10.85	10.71	1.70	1.46
COOH	−7.40	−7.31	−0.01	0.26	8.77	9.03	10.85	10.75	2.08	1.72
CF_3_	−7.62	−7.42	−0.05	0.33	8.72	9.11	11.07	10.87	2.35	1.76
CHO	−7.48	−7.35	−0.37	0.19	8.40	8.96	10.93	10.80	2.53	1.83
CN	−7.79	−7.52	−0.52	0.09	8.24	8.86	11.24	10.96	3.00	2.10
NO_2_	−7.91	−7.60	−0.73	−0.18	8.04	8.59	11.36	11.04	3.31	2.45

E(HOMO butadiene) = −8.77; E(LUMO butadiene) = 3.45.

### 2.2. Thermodynamic Parameters

**Scheme 1:**
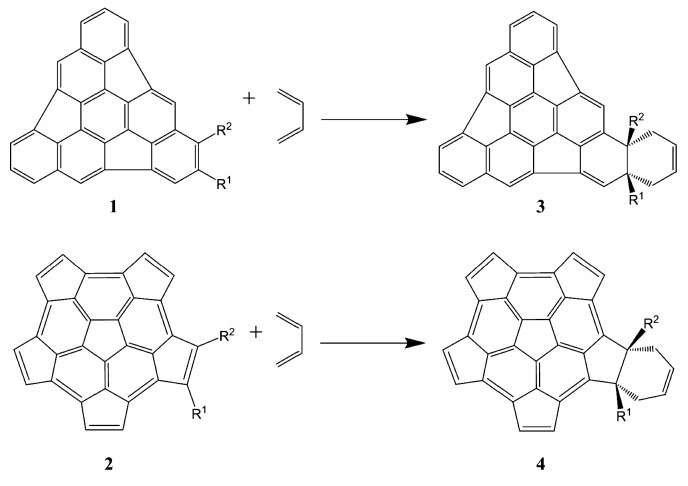
DA reactions for the substituted fragments **1** (**upper panel**) and **2** (**lower panel**).

The evaluation of the global reactions in terms of the thermodynamic parameters allows us to determine the stability of the proposed products and therefore, the feasibility of the reactions. The proposed reactions are shown in Scheme 1 and the standard Gibbs energies ΔrG°, standard enthalpies ΔrH°, and standard entropies TΔrS° of reaction are shown in Table 2 and Table S3.

**Table 2:** Standard Gibbs energies ΔrG°, enthalpies ΔrH°, and entropies TΔrS° for the reaction of butadiene with the substituted fragment **2**.

Substituent R^1^	ΔrG° (kcal·mol^−1^)		ΔrH° (kcal·mol^−1^)		TΔrS° (kcal·mol^−1^)	
	R^2^ = R^1^	R^2^ = H	R^2^ = R^1^	R^2^ = H	R^2^ = R^1^	R^2^ = H
NH_2_	2.43	−3.93	−13.87	−19.10	−16.30	−15.17
OMe	1.44	−4.64	−15.29	−19.79	−16.72	−15.15
OH	−9.10	−9.33	−24.94	−24.51	−15.84	−15.18
Me	2.43	−6.54	−13.38	−21.75	−15.80	−15.21
*i*-Pr	15.88	2.04	−1.07	−14.18	−16.96	−16.22
H	−13.05	−13.05	−27.88	−27.88	−14.82	−14.82
F	−18.56	−15.38	−33.99	−30.30	−15.43	−14.93
COOH	3.66	−5.01	−12.41	−20.06	−16.07	−15.05
CF_3_	−1.23	−8.47	−17.54	−24.26	−16.30	−15.78
CHO	4.30	−4.82	−10.47	−19.43	−14.77	−14.61
CN	3.45	−5.43	−11.90	−20.46	−15.36	−15.02
NO_2_	−7.70	−10.50	−23.81	−25.46	−16.10	−14.96

All the reactions are entropically disfavored. For fragment **1** (Table S3), the trends in the thermodynamic parameters across the two families studied are the same for the mono- and di-substituted fragments. However, the values of ΔrG° and ΔrH° for the mono-substituted fragments are, in most cases, less positive (or more negative) than those calculated for the di-substituted fragments. All the reactions are non-spontaneous (ΔrG° > 0), and only the reactions with fragment **1** substituted with OH, Me, F, CF_3_ and NO_2_ are exothermic (ΔrH° < 0). The reactions with fragment **1** substituted with F and NO_2_ groups are less non-spontaneous than the reactions with the pristine fragment **1**, while the reactions with the other substituents have larger values of ΔrG° than those obtained for the non-substituted fragment.

For fragment **2** (Table 2), a difference is observed between mono substitution and double substitution. All the reactions are exothermic (ΔrH° < 0) and entropically disfavored (TΔrS° < 0); however, the trends in the Gibbs free energies are different for mono and double substitution. In the case of the di-substituted fragment **2** only the reactions with the fragment substituted with OH, F, CF_3_ and NO_2_ groups are spontaneous (ΔrG° < 0). On the other hand, all the reactions with mono-substituted fragment are spontaneous (ΔrG° < 0), except for the fragment substituted with *i*-Pr. Only the fragments mono- and disubstituted with F lead to reactions thermodynamically more favorable than the reactions of the non-substituted fragment, even when the fragment mono- and disubstituted with NO_2_ was expected to be more favorable.

**Scheme 2:**
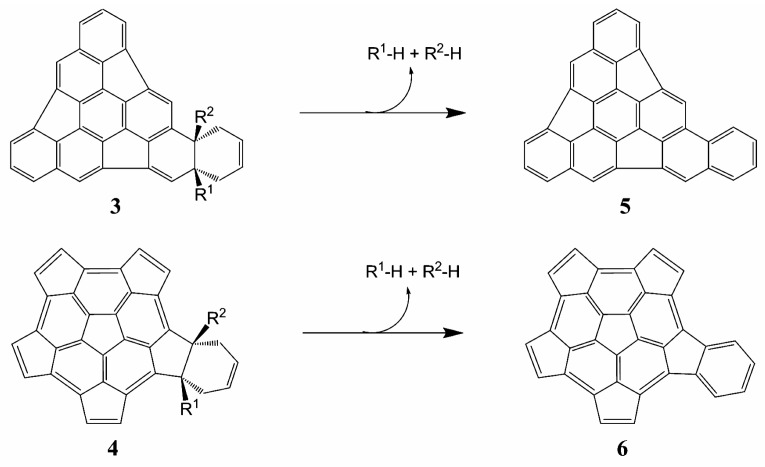
In the aromatization reactions, the substituents R^1^ and R^2^ and two hydrogen atoms (one in case of mono substitution) are eliminated.

We have also investigated the aromatization process in which the elimination of the substituents R^1^ and R^2^ and two H atoms (one in the case of monosubstitution) in the formed adducts leads to a new aromatic ring in the fragment. The proposed aromatization reactions are shown in Scheme 2 and the standard Gibbs energies ΔrG°, standard enthalpies ΔrH°, and standard entropies TΔrS° of those reactions are given in Table 3 and Table S4.

All the aromatization reactions are spontaneous (ΔrG° < 0), exothermic (ΔrH° < 0) and favored by entropy (TΔrS° > 0), which indicates that the formed cycloadducts are unstable and willing to aromatize. This favors the use of substituents in the DA reaction because of the easy elimination of the substituent from the final product. As previously highlighted by Scott [23], the aromatization step following the DA cycloaddition reaction occurs spontaneously without the presence of any oxidizing agent. Also, it is interesting to observe that in all the examples cited by Scott, the aromatization reactions were obtained by loss of two equivalents of H_2_. Our calculations support the elimination of R^1^-H + H_2_ for the mono-substituted system and R^1^-H + R^2^-H for the di-substituted systems.

**Table 3:** Standard Gibbs energies ΔrG°, enthalpies ΔrH°, and entropies TΔrS° of the aromatization reaction of the DA cycloadduct **4** in Scheme 2.

Substituent R^1^	ΔrG° (kcal·mol^−1)^		ΔrH° (kcal·mol^−1^)		TΔrS° (kcal·mol^−1^)	
	R^2^ = R^1^	R^2^ = H	R^2^ = R^1^	R^2^ = H	R^2^ = R^1^	R^2^ = H
NH_2_	−48.33	−28.37	−24.23	−7.66	24.11	20.71
OMe	−54.12	−31.89	−28.05	−10.85	26.07	21.04
OH	−38.72	−25.92	−14.94	−5.58	23.78	20.35
Me	−53.70	−30.18	−29.28	−9.46	24.42	20.72
*i*-Pr	−74.94	39.90	−46.76	−17.25	28.18	22.65
H	−10.48	−10.48	6.51	6.51	16.99	16.99
F	−28.48	−17.54	−7.38	1.42	21.10	18.96
COOH	−30.95	−16.12	−4.73	5.32	26.22	21.44
CF_3_	−39.97	−20.27	−13.68	1.06	26.29	21.33
CHO	−28.53	−16.68	−3.95	4.04	24.57	20.72
CN	−28.48	−17.17	−6.31	2.40	22.17	19.57
NO_2_	−40.66	−19.16	−15.27	1.79	25.40	20.96

### 2.3. Kinetic Parameters

**Figure 4:**
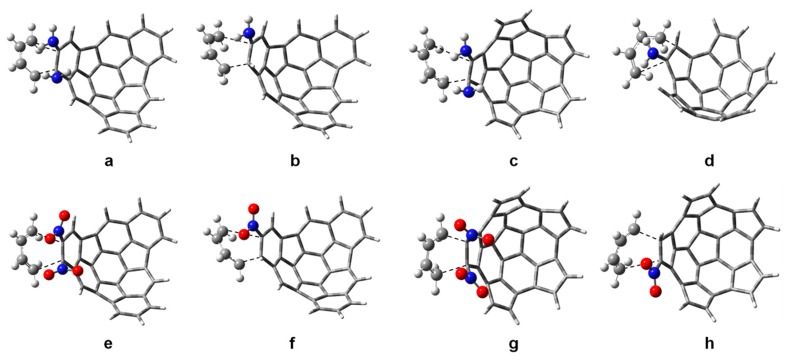
Transition state structures for the reaction of butadiene with: (**a**) fragment **1** substituted with two NH_2_ groups; (**b**) fragment **1** substituted with one NH_2_ group; (**c**) fragment **2** substituted with two NH_2_ groups; (**d**) fragment **2** substituted with one NH_2_ group; (**e**) fragment **1** substituted with two NO_2_ groups; (**f**) fragment **1** substituted with one NO_2_ group; (**g**) fragment **2** substituted with two NO_2_ groups; (**h**) fragment **2** substituted with one NO_2_ group.

The transition state structures of the proposed reactions are shown in Figure 4, Figures S5 and S6. The distances r1 and r2 for the transition states (r1 is the distance between carbon 1 of butadiene and carbon 29 of the fragment, and r2 is the distance between carbon 4 of butadiene and carbon 30 of the fragment; see Figure S7) are given in Table 4 and Table S5. Small values of Δr = r1 − r2 suggest synchronous reactions, while large values of Δr suggest asynchronous reactions. In general, the presence of substituents makes the reactions asynchronous. However, the reactions of fragment **1** di-substituted with *i*-Pr, CF_3_ and CN, and fragment **2** di-substituted with Me, F, CF_3_ and CN, preserve the synchronicity of the non-substituted fragments **1** and **2**. The mono-substituted fragments follow the trend of the di-substituted fragments and the synchronicity of the reactions is preserved for the same substituent groups. This similarity in the behavior of mono- and disubstituted fragments can be understood from the size and shape of the substituents. The F atom is just bigger than the H atom, and the linear shape of CN is like that of a C-H bond, just longer. The tetrahedral geometry of CF_3_ and Me makes these substituents occupy a spherical-like space, like a big single atom. The *i*-Pr substituent is also tetrahedral but this effect is only observed in fragment **1**, which has more space available for accommodating the substituent groups.

**Table 4:** Distances r1 and r2, in Å, for the transition states of reactions of fragment **2** shown in Figure S6. Also Δr = r1 − r2.

Substituent R^1^	r1		r2		Δr	
	R^2^ = R^1^	R^2^ = H	R^2^ = R^1^	R^2^ = H	R^2^ = R^1^	R^2^ = H
NH_2_	3.48	3.28	1.88	1.91	1.60	1.37
OMe	3.27	2.98	1.88	1.93	1.39	1.05
OH	2.94	3.34	1.91	1.91	1.03	1.43
Me	2.26	2.38	2.26	2.17	0	0.21
*i*-Pr	2.93	2.44	1.79	2.11	1.14	0.33
H	2.27	2.27	2.27	2.27	0	0
F	2.30	2.33	2.27	2.22	0.03	0.11
COOH	3.06	2.68	1.97	1.99	1.09	0.69
CF_3_	2.27	2.34	2.27	2.21	0.01	0.13
CHO	2.69	2.69	1.97	1.98	0.72	0.71
CN	2.24	2.64	2.24	2.02	0	0.62
NO_2_	2.19	3.04	1.78	1.96	0.41	1.08

Table 5 and Table S6 summarize the calculated activation energies Ea, the standard activation Gibbs energies Δ^‡^G°, standard activation enthalpies Δ^‡^H°, and standard activation entropies TΔ^‡^S° of the proposed DA reactions. In the case of double substitution of the fragments **1** and **2**, only the fragments substituted with NO_2_ present lower activation energy than the non-substituted fragments. In the case of the mono-substituted fragments, the activation energy values are closer to those obtained for the non-substituted fragments. However, only the reactions of fragment **1** substituted with one NH^2^, OMe, OH, COOH, CHO or NO_2_ group have lower Ea than the non-substituted fragment **1**; and in the case of fragment **2**, only the activation energy barriers for the fragments mono-substituted with NO_2_ is lower than the activation barriers for the non-substituted fragment **2**. For both fragments **1** and **2**, mono- and disubstituted, the lowest activation barriers are observed for substitution with NO_2_ groups.

**Table 5:** Activation energy Ea, standard activation Gibbs energy Δ^‡^G°, standard activation enthalpy Δ^‡^H°, and standard activation entropy TΔ^‡^S° for the Diels-Alder reactions of butadiene with the substituted fragment **2**. Data for the reaction with the non-substituted fragment (H) are given as reference.

Substituent R^1^	Ea (kcal·mol^−1^)		Δ^‡^G° (kcal·mol^−1^)		Δ^‡^H° (kcal·mol^−1^)		TΔ^‡^S° (kcal·mol^−1^)	
	R^2^ = R^1^	R^2^ = H	R^2^ = R^1^	R^2^ = H	R^2^ = R^1^	R^2^ = H	R^2^ = R^1^	R^2^ = H
NH_2_	28.06	24.03	41.38	36.61	27.28	23.54	−14.11	−13.07
OMe	29.21	26.82	42.63	40.32	28.40	25.87	−14.22	−14.45
OH	27.99	24.03	40.70	36.46	27.37	23.47	−13.34	−12.99
Me	30.73	26.60	44.42	39.92	29.86	25.91	−14.55	−14.01
*i*-Pr	46.86	31.34	61.52	45.17	45.67	30.52	−15.85	−14.66
H	22.61	22.61	35.52	35.52	22.02	22.02	−13.51	−13.51
F	25.60	24.67	38.39	37.65	25.12	24.14	−13.27	−13.51
COOH	23.60	22.88	37.52	36.34	22.98	22.25	−14.54	−14.09
CF_3_	25.23	23.22	39.30	37.32	24.42	22.50	−14.88	−14.82
CHO	27.54	22.98	40.88	36.14	26.88	22.37	−14.01	−13.78
CN	27.18	23.55	40.58	36.58	26.56	23.03	−14.03	−13.56
NO_2_	19.80	19.03	35.78	32.18	18.22	18.51	−17.56	−13.67

Whereas the presence of two substituents enhances the electronic effect by reducing the HOMO-LUMO gap, and therefore favoring the reactions, the steric hindrance produced by the agglomeration of atoms in the di-substituted fragments increases the activation energy Ea, making these reactions less favored than the reactions with the non-substituted fragments. The reactions with the mono-substituted fragments appear to be improved. The activation energies are similar or even smaller, when the substituents are COOH, CHO and NO_2_, than the reactions with the non-substituted fragments.

The intrinsic reaction coordinates, IRC, of the proposed DA reactions were calculated and are shown in Figures S8 to S11. The review of the reaction mechanisms shows that the transition structures obtained (IRC = 0) lead to products (forward direction) and reagents (reverse direction) without evidence of activated complexes or intermediates.

## 3. Computational Methods

The calculations described in this work have been carried out using the density functional theory with the implementation provided in the Gaussian 09 (G09) program package [24]. The geometries of substituted triindenetriphenylene **1** and pentacyclopentacorannulene **2** depicted in Figure 2 have been fully optimized at the B3LYP level of theory, with the 6-31G(d,p) basis set to expand the electronic wave functions. The B3LYP exchange-correlation functional has been used to study the pi-sigma conversion in C-C bond formation in the Diels-Alder cycloaddition reaction of oxazole with ethylene [25], and the sp^2^-sp conversion for carbenes [26]. Also the addition of polarization functions improves the description of the pi-sigma conversion in the C-C bond formation [27]. First, a test of the accuracy of the B3LYP method in comparison with experiment and with other theoretical methods has been performed for the Diels-Alder cycloaddition reaction between 1,3-butadiene and ethylene. We have chosen this reaction because it is a well studied reaction, and their experimental kinetic and thermodynamic parameters are known. In the test, the B3LYP method has been compared to other well known methods, some of them including dispersion effects. The results for the activation energy, standard activation Gibbs energy, standard activation enthalpy, and standard activation entropy for the Diels-Alder reaction between ethylene and butadiene, given in Table S1 of the Supplementary Materials, indicate that the results of the B3LYP method are fully satisfactory in comparison with experiment. The same result was obtained by comparing the standard Gibbs energies, enthalpies, and entropies for the reaction, and by comparing the standard Gibbs energies, enthalpies and entropies for the aromatization, although in the later cases the set of theoretical methods tested was not as complete as in Table S1. Based on the satisfactory results delivered by the reaction between 1,3-butadiene and ethylene, we have decided to use the same method to study the Diels-Alder reactions between the substituted hemifullerenes and 1,3-butadiene. This method and basis set have been often used in the study of fullerene reactivity [28,29], leading to good agreement with geometrical parameters of fullerene fragments obtained by X-ray diffraction [30]. The methodology has proven to describe satisfactorily the DA cycloaddition reactions over fullerenes and their fragments [17,31,32]. Therefore, the kinetic and thermodynamic parameters were also calculated at the B3LYP/6-31G(d,p) level of theory. The transition states (TS) for the reactions of the substituted fragments **1** and **2** with 1,3-butadiene (see all the structures in the Supplementary Materials) were located with the QST2 G09 optimization option of the code; for all of them, vibrational frequency analyses were carried out. A single imaginary frequency was located for each transition state. All frequencies are real for the minima. The electronic energies of the minima and the transition states were corrected by the subtraction of zero-point energies. The Intrinsic Reaction Coordinates (IRCs) were determined from the corresponding TS using the IRC G09 keyword. In each case the Forward and Reverse sections were calculated independently. In the case of the fragments substituted with NO_2_ the TS was first located using the Nudge Elastic Band (NEB) method [33] implemented in the DACAPO code [34] and, in a second step, reoptimized with the G09 code and treated as the rest of the molecules following the previously mentioned method. The rigid-rotor-harmonic-oscillator approximation was employed to compute the H, S and G state functions.

## 4. Conclusions

The Diels-Alder cycloaddition reaction is one of the most commonly utilized reactions in chemical synthesis of six member carbon rings. The study of the electronic, thermodynamic and kinetic parameters of the DA reaction of butadiene with the rims of substituted pristine hemifullerenes performed in this work suggest that the reactions over triindenetriphenylene **1** and pentacyclopentacorannulene **2** may be favored by the use of specific substituents.

The reactions with the hemifullerene **1** are not favored thermodynamically, that is, the Gibbs free energies of the reactions are positive, despite the employed substituent. On the other hand, the reactions with fragment **2** substituted with F, NO_2_ and a few other groups are thermodynamically favored. However, only the reactions with the fragment **2** substituted with one or two F atoms are thermodynamically preferred over the reaction with the pristine fragment **2**.

In terms of activation energies, reactions with fragment **1** substituted with one NH_2_, OMe, OH, COOH, CHO, NO_2_ group, or two NO_2_ groups, have lower activation energies than the reaction with the non-substituted fragment. The reactions with fragment **2** mono- and di-substituted with NO_2_ groups have lower activation energies than the reaction with the non-substituted fragment (although other substituents like F, COOH and CF_3_ have activation energies only a little higher than the pristine fragment **2**).

It is known that the electronic effect of the electron-withdrawing and electron-donating groups is important in typical DA reactions. But in this case, where the system attached to the dienophile is big, the steric hindrance of the substituents plays such an important role that the reaction barriers are comparable with those for the non-substituted fragments. Also, the aromatic nature of fragments **1** and **2** counteracts the electronic effect, producing a delocalization of the electronic density over the whole fragment that may have some influence on the lack of reduction of the activation barriers.

Taking the electronic, thermodynamic, and kinetic parameters into account, it appears that the most promising electron-withdrawing substituent favoring the DA cycloaddition reaction of fragments **1** and **2** with 1, 3-butadiene is NO_2_. This conclusion supports chemical intuition. It is also consistent with the finding of Fort and Scott, mentioned in the Introduction, that the use of nitro-derivatives of ethylene in DA reactions provides an efficient way to increase the number of benzene rings in polycyclic aromatic hydrocarbons.

Carbon fullerenes are traditionally produced by the arc-discharge vaporization of graphite, by chemical vapor deposition, and by combustion processes [35,36]. These methods, however, are not very efficient, and new methods based on a bottom-up chemical synthesis approach would be more desirable and controllable. We hope that this theoretical study can guide experimentalists and potentially hasten the rational, scalable synthesis of fullerenes.

## Supplementary Materials

Supplementary materials can be accessed at: http://www.mdpi.com/1420-3049/21/2/200/s1.
