# Supplementary material for: The Diels-Alder Cycloaddition Reaction of Substituted Hemifullerenes with 1,3-Butadiene: Effect of Electron-Donating and Electron-Withdrawing Substituents
Source: Molecules. 2016 Feb 12;21(2):200. doi: 10.3390/molecules21020200 (PMC6272991; doi:10.3390/molecules21020200)
Supplement: Supplementary file 1 [file molecules-21-00200-s001.pdf]

Supplementary Materials: The Diels-Alder Cycloaddition Reaction of Substituted Hemifullerenes with 1,3-butadiene: Effect of Electron-Donating and Electron-Withdrawing Substituents

Martha Mojica [1], Francisco Méndez [1,*] and Julio A. Alonso [2]

Table S1. Activation energy Ea, standard activation Gibbs energy Δ‡G°, standard activation enthalpy Δ‡H°, and standard activation entropy TΔ‡S° for the Diels-Alder reaction between ethylene and butadiene. Comparison between the experimental values and the calculated values using the B3LYP, PBE, M06L, M06, PBE0 and MP2 methods. The experimental values are taken from K. N. Houk, R. J. Loncharich, J. F. Blake, W. L. Jorgensen, J. Am.Chem. Soc. 1989, 111, 9172, and V. Guner, K. S. Khuong, A. G. Leach, P. S. Lee, M. D. Bartberger, K. N. Houk, J. Phys. Chem. A 2003, 107, 11445.

Method	Ea kcal·mol^{-1}	Δ‡G° kcal·mol^{-1}	Δ‡H° kcal·mol^{-1}	TΔ‡S° kcal·mol^{-1}
B3LYP	24.80	36.35	23.41	−12.93
PBE	15.72	27.09	14.47	−12.62
M06L	19.87	31.28	18.56	−12.71
M06	20.89	32.35	19.53	−12.82
PBE0	19.34	30.85	18.00	−12.85
MP2	19.39	30.98	18.00	−12.98
M06-2X	17.47	29.54	16.06	−13.48
Exp	27.5 [a] 24.2–26.7 [b]	-	24.2 [d]	−13.32 [f]
Calculated	25.9 [c]	-	24.9 [e]	−12.76 [g]

[a] Ea Experimental; [b] Ea Experimental from the cyclohexane cyclorevertion and the experimental heath of reaction; [c] Ea calculated; [d] Δ‡H° Experimental; [e] Δ‡H° calculated; [f] TΔ‡S° Experimental, [g] TΔ‡S° calculated.

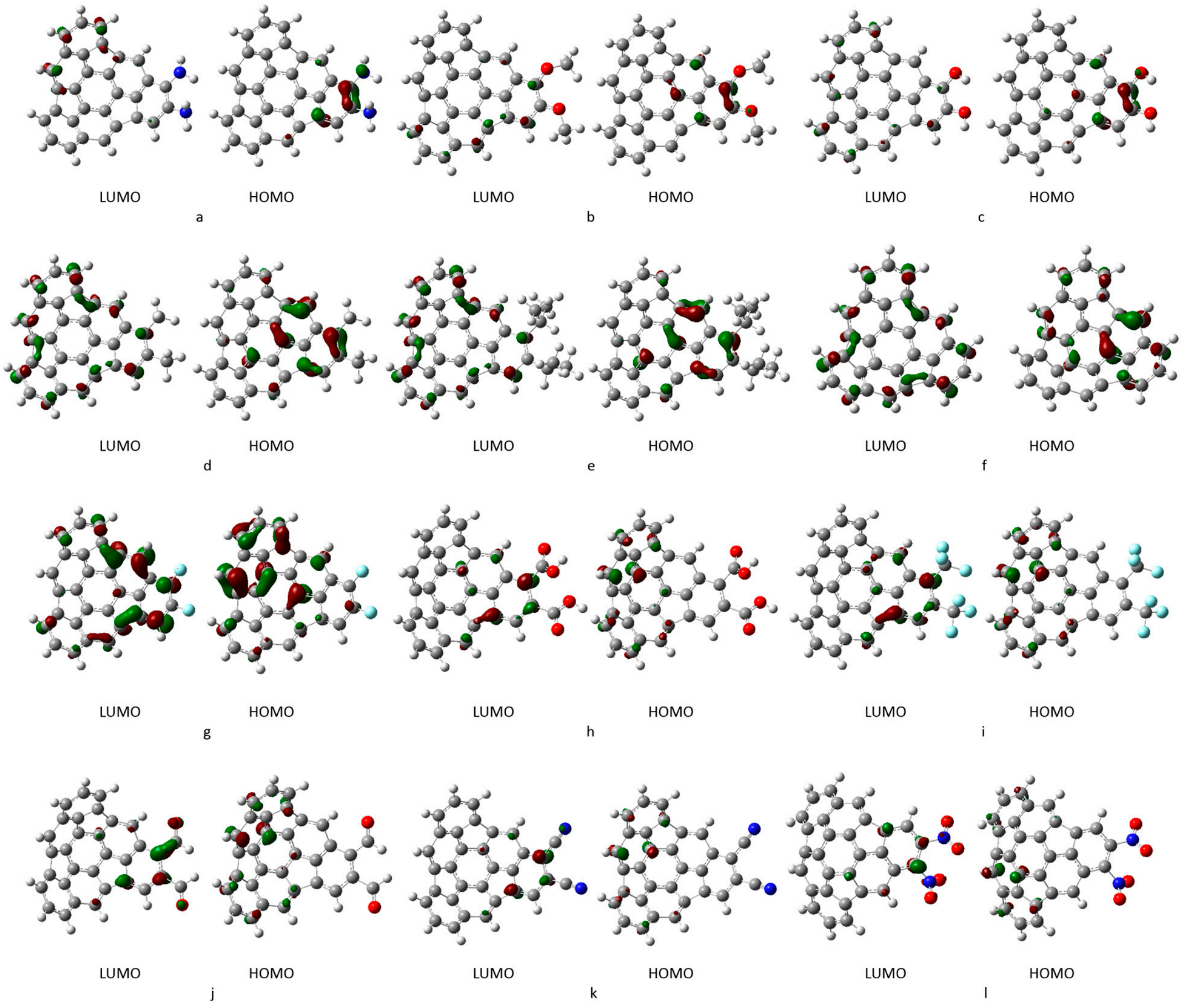

Figure S1. Frontier molecular orbitals HOMO and LUMO of the fragment 1 substituted with two equal molecular groups: (**a**) NH_2; (**b**) Ome; (**c**) OH; (**d**) Me; (**e**) *i*-Pr; (**f**) H; (**g**) F; (**h**) COOH; (**i**) CF_3; (**j**) CHO; (**k**) CN; (**l**) NO_2. The substituent molecules are on the right side of the fragment. The large grey, blue and red spheres represent carbon, nitrogen and oxygen atoms, respectively, and hydrogen atoms are represented by the small grey spheres. The dark red and green surfaces represent the positive and negative lobes of the calculated molecular orbitals respectively, plotted at 0.08 au.

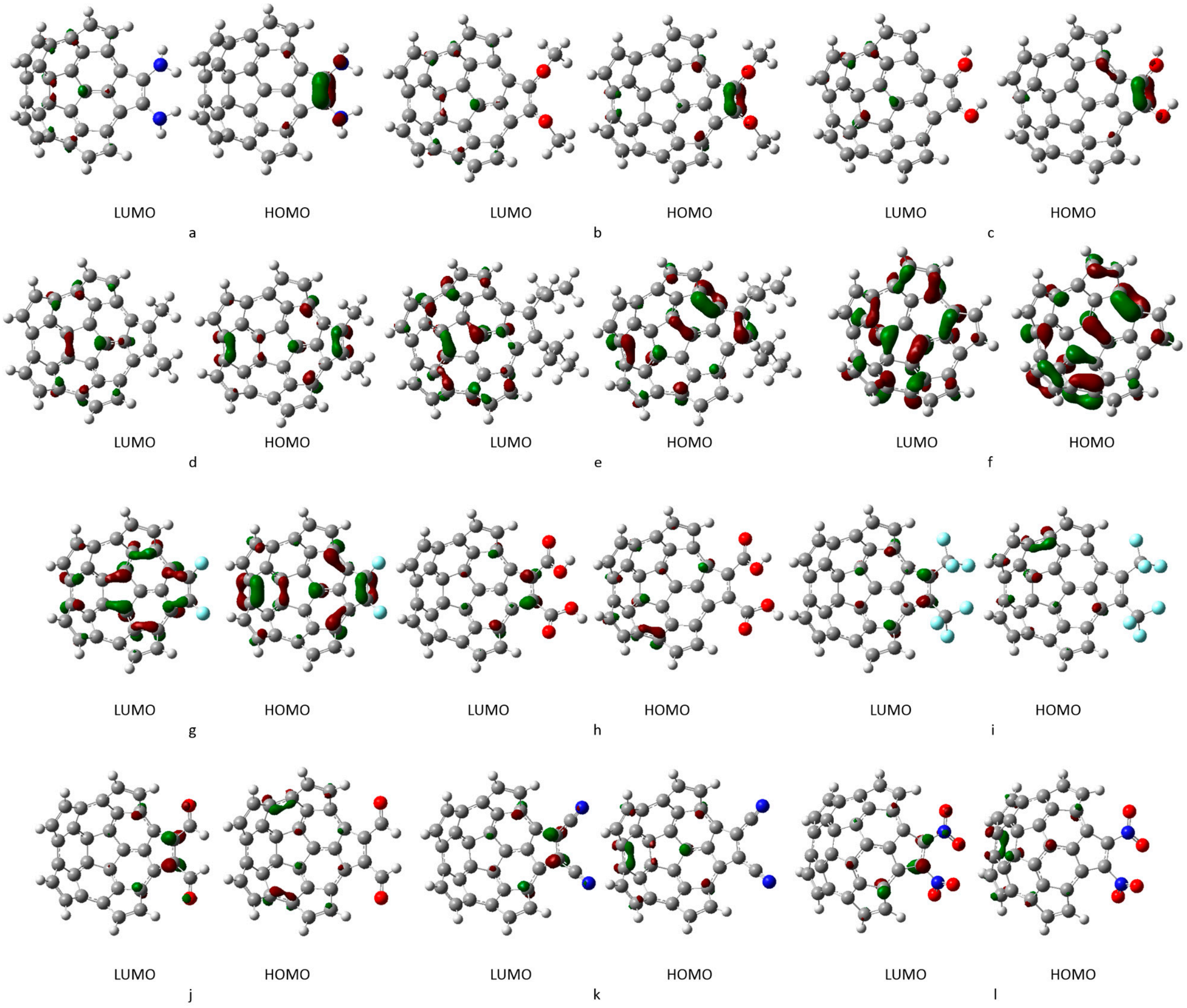

Figure S2. Frontier molecular orbitals HOMO and LUMO of the fragment **2** substituted with two equal molecular groups: (**a**) NH_2; (**b**) OMe; (**c**) OH; (**d**) Me; (**e**) *i*-Pr; (**f**) H; (**g**) F; (**h**) COOH; (**i**) CF_3; (**j**) CHO; (**k**) CN; (**l**) NO_2. The substituent molecules are on the right side of the fragment. The large grey, blue and red spheres represent carbon, nitrogen and oxygen atoms, respectively, and hydrogen atoms are represented by the small grey spheres. The dark red and green surfaces represent the positive and negative lobes of the calculated molecular orbitals respectively, plotted at 0.08 au.

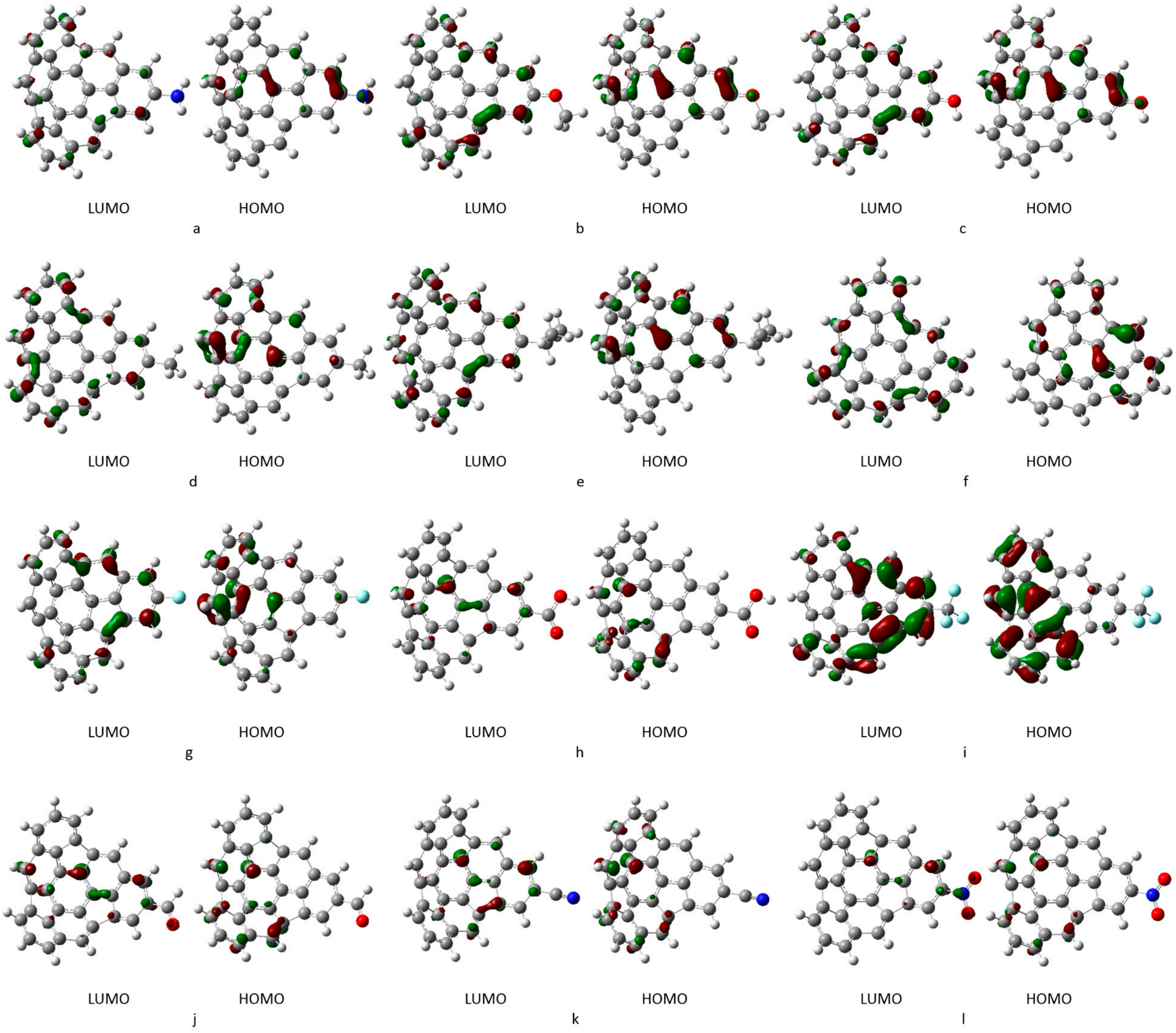

Figure S3. Frontier molecular orbitals HOMO and LUMO of the fragment **1** substituted with one molecular group: (**a**) NH_2; (**b**) OMe; (**c**) OH; (**d**) Me; (**e**) *i*-Pr; (**f**) H; (**g**) F; (**h**) COOH; (**i**) CF_3; (**j**) CHO; (**k**) CN; (**l**) NO_2. The substituent molecules are on the right side of the fragment. The large grey, blue and red spheres represent carbon, nitrogen and oxygen atoms, respectively, and hydrogen atoms are represented by the small grey spheres. The dark red and green surfaces represent the positive and negative lobes of the calculated molecular orbitals respectively, plotted at 0.08 au.

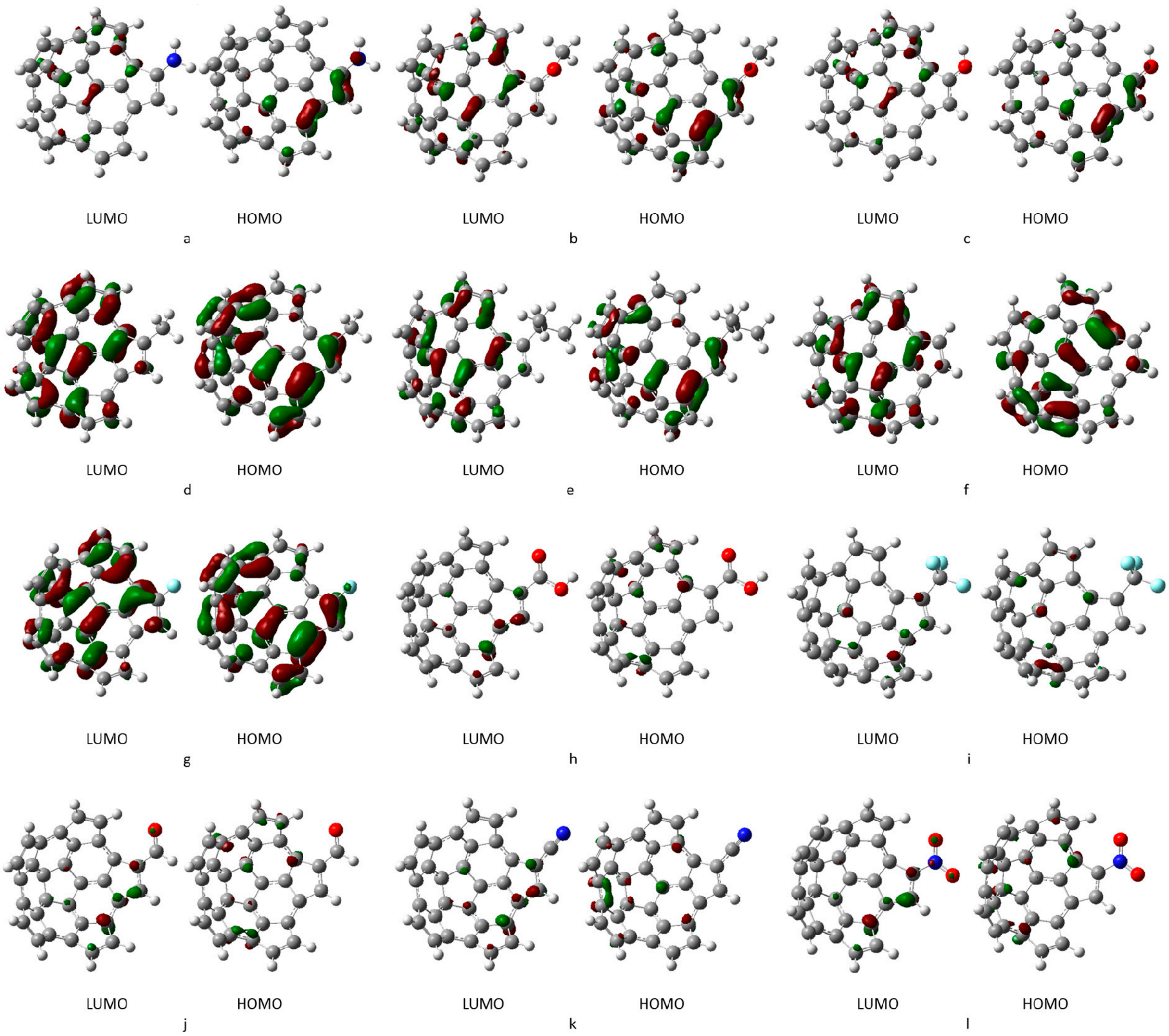

Figure S4. Frontier molecular orbitals HOMO and LUMO of the fragment **2** substituted with one molecular group: (**a**) NH_2; (**b**) OMe; (**c**) OH; (**d**) Me; (**e**) *i*-Pr; (**f**) H; (**g**) F; (**h**) COOH; (**i**) CF_3; (**j**) CHO; (**k**) CN; (**l**) NO_2. The substituent molecules are on the right side of the fragment. The large grey, blue and red spheres represent carbon, nitrogen and oxygen atoms, respectively, and hydrogen atoms are represented by the small grey spheres. The dark red and green surfaces represent the positive and negative lobes of the calculated molecular orbitals respectively, plotted at 0.08 au.

Table S2. HOMO and LUMO energies (in eV) of the di-substituted ($R^1 = R^2$) and mono-substituted fragment **1**, and HOMO-LUMO gaps ΔE, for the NEDDA and IEDDA reactions with 1,3-butadiene. ΔENEDDA = ELUMOdienophile − EHOMOdiene and ΔEIEDDA = ELUMOdiene − EHOMOdienopile. Also, δΔE = ΔEIEDDA − ΔENEDDA. Results for the reaction with the non-substituted fragment (row for H in the middle of the Table) are included for comparison.

**Substituent R^1**	**EHOMO**		**ELUMO**		**ΔENEDDA**		**ΔEIEDDA**		**δΔE**	
	**$R^2 = R^1$**	**$R^2 = H$**	**$R^2 = R^1$**	**$R^2 = H$**	**$R^2 = R^1$**		**$R^2 = R^1$**	**$R^2 = H$**	**$R^2 = R^1$**	**$R^2 = H$**
NH_2	−6.70	−6.95	1.48	1.38	10.26	10.16	10.15	10.40	−0.11	0.25
OMe	−7.01	−7.10	1.39	1.31	10.16	10.09	10.46	10.55	0.29	0.46
OH	−7.02	−7.17	1.38	1.27	10.16	10.04	10.47	10.62	0.31	0.58
Me	−7.13	−7.23	1.42	1.38	10.20	10.16	10.58	10.67	0.39	0.52
i-Pr	−7.15	−7.21	1.47	1.38	10.18	10.15	10.60	10.66	0.42	0.50
H	−7.28	−7.28	1.38	1.38	10.15	10.15	10.73	10.73	0.58	0.58
F	−7.50	−7.38	1.08	1.16	9.42	9.87	10.96	10.83	1.53	0.96
COOH	−7.53	−7.43	0.71	1.09	9.48	9.86	10.98	10.88	1.50	1.02
CF_3	−7.67	−7.51	0.65	1.10	9.42	9.87	11.12	10.96	1.70	1.08
CHO	−7.57	−7.48	0.32	1.02	9.10	9.80	11.02	10.92	1.92	1.13
CN	−7.85	−7.61	0.17	0.90	8.94	9.67	11.29	11.05	2.35	1.38
NO_2	−7.94	−7.67	−0.03	0.67	8.75	9.44	11.39	11.11	2.64	1.67

EHOMO butadieno = −8.77, ELUMO butadieno = 3.45.

Table S3. Standard Gibbs energies ΔrG°, enthalpies ΔrH°, and entropies TΔrS° for the reaction of butadiene whit the substituted fragment **1**.

**Substituent R^1**	**ΔrG° ($kcal·mol^{-1}$)**		**ΔrH° ($kcal·mol^{-1}$)**		**TΔrS° ($kcal·mol^{-1}$)**	
	**$R^2 = R^1$**	**$R^2 = H$**	**$R^2 = R^1$**	**$R^2 = H$**	**$R^2 = R^1$**	**$R^2 = H$**
NH_2	25.06	17.78	9.56	2.83	−15.50	−15.88
OMe	22.26	15.45	6.18	0.58	−16.08	−14.87
OH	15.05	12.03	−0.62	−2.74	−15.67	−14.78
Me	16.03	12.19	−0.27	−3.69	−16.30	−15.88
i-Pr	26.27	16.40	9.25	0.49	−17.01	−15.91
H	10.34	10.34	−4.28	−4.28	−14.62	−14.62
F	5.97	7.88	−9.00	−6.74	−14.97	−14.62
COOH	15.33	15.24	−0.33	0.51	−15.65	−14.74
CF_3	11.32	10.73	−5.16	−5.13	−16.48	−15.86
CHO	17.05	16.27	2.18	1.56	−14.87	−14.71
CN	23.95	16.52	9.14	1.91	−14.82	−14.61
NO_2	3.83	10.55	−12.19	−4.59	−16.02	−15.14

Table S4. Standard Gibbs energies ΔrG°, enthalpies ΔrH°, and entropies TΔrS° for the aromatization reaction of the DA cycloadduct 3 in Scheme **1**.

**Substituent R^1**	**ΔrG° ($kcal·mol^{-1}$)**		**ΔrH° ($kcal·mol^{-1}$)**		**TΔrS° ($kcal·mol^{-1}$)**	
	**$R^2 = R^1$**	**R^2 = H**	**$R^2 = R^1$**	**R^2 = H**	**$R^2 = R^1$**	**R^2 = H**
NH_2	−65.89	−46.18	−41.55	−25.75	24.33	20.43
OMe	−68.02	−44.73	−42.34	−23.76	25.68	20.97
OH	−54.80	−40.80	−31.14	−20.85	23.66	19.95
Me	−64.19	−42.89	−39.18	−21.85	25.01	21.04
i-Pr	−83.86	−47.47	−55.81	−25.55	28.05	21.92
H	−26.56	−26.56	−9.80	−9.80	16.77	16.77
F	−43.77	−32.55	−22.97	−13.84	20.79	18.71
COOH	−38.94	−29.87	−12.84	−8.86	26.10	21.02
CF_3	−49.38	−32.76	−22.94	−11.30	26.43	21.47
CHO	−39.14	−31.57	−15.01	−10.88	24.14	20.69
CN	−45.23	−33.42	−23.57	−14.24	21.66	19.17
NO_2	−45.84	−31.90	−20.39	−10.91	25.45	20.99

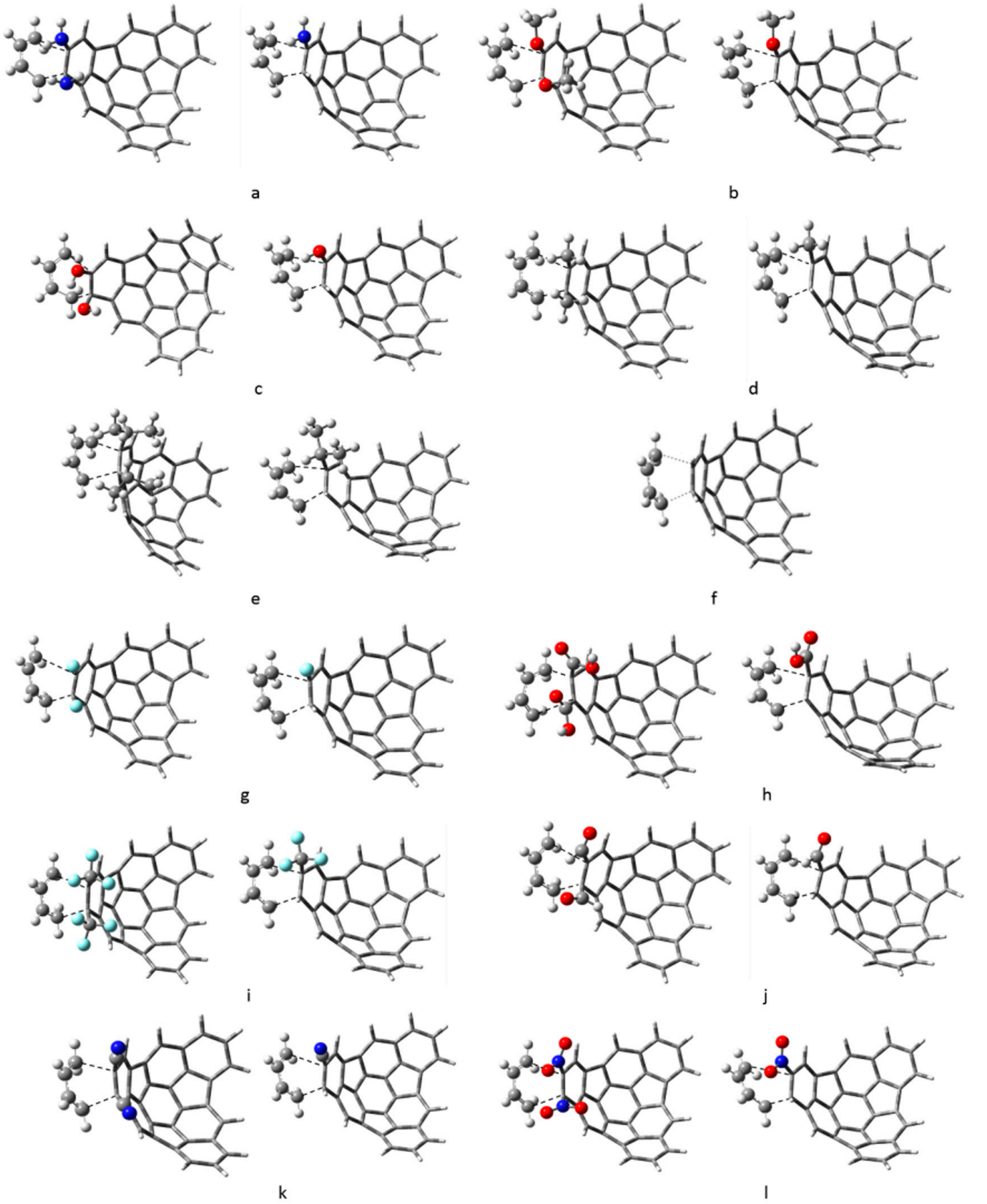

Figure S5. Transition state structures for the reaction of butadiene with fragment **1** substituted with two and one groups: (**a**) NH_2; (**b**) OMe; (**c**) OH; (**d**) Me; (**e**) *i*-Pr; (**f**) H; (**g**) F; (**h**) COOH; (**i**) CF_3; (**j**) CHO; (**k**) CN; (**l**) NO_2.

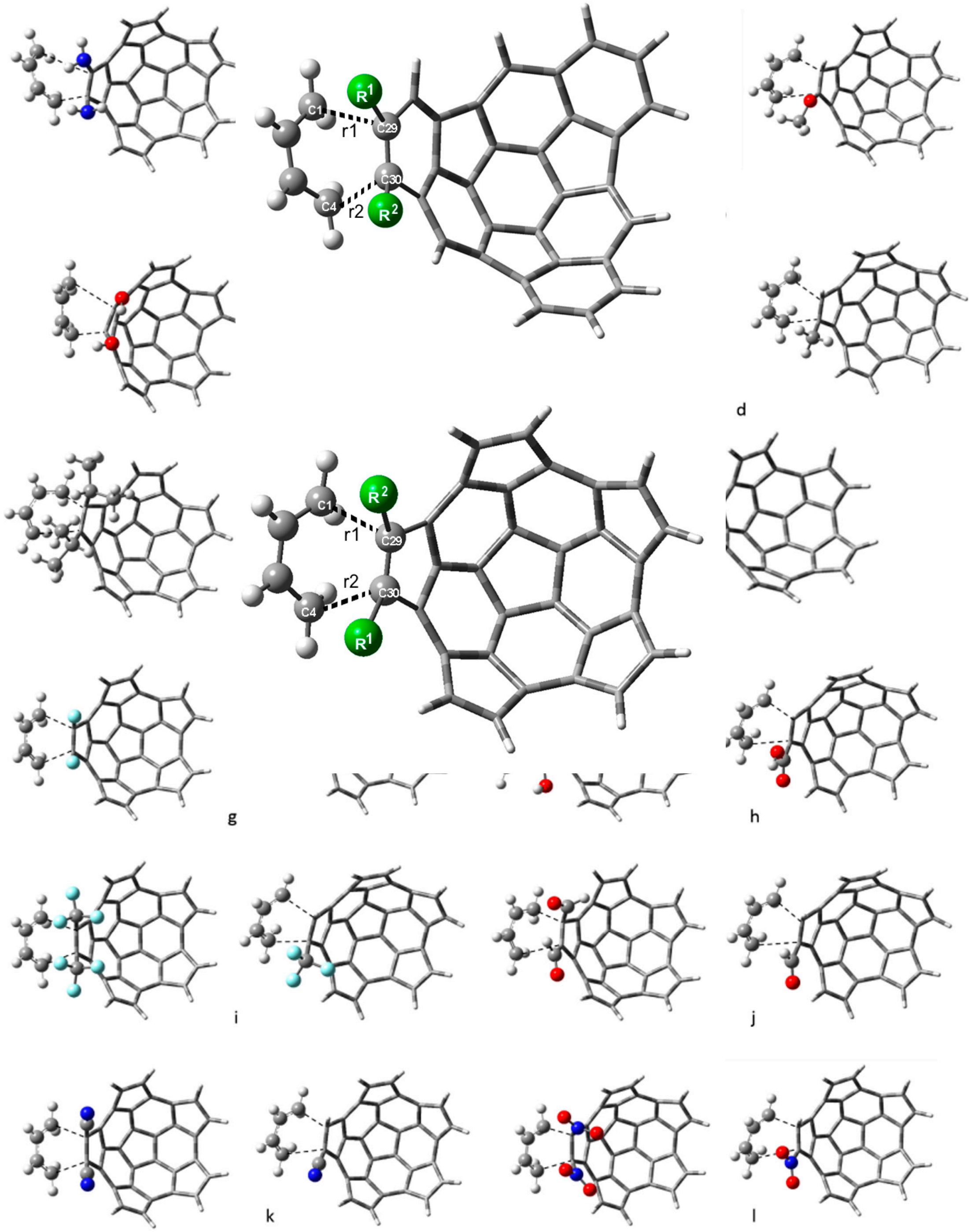

Figure S6. Transition state structures for the reaction of butadiene with fragment **2** substituted with two and one groups: (**a**) NH_2; (**b**) OMe; (**c**) OH; (**d**) Me; (**e**) *i*-Pr; (**f**) H; (**g**) F; (**h**) COOH; (**i**) CF_3; (**j**) CHO; (**k**) CN; (**l**) NO_2.

Figure S7. Definition of the transition distances r1 and r2**.**

Table S5. Distances r1 and r2, in Å, for the transition states of reactions of fragment **1** shown in Figure S5. Also Δr = r1 − r2.

Substituent R^1	r1		r2		Δr	
	$R^2 = R^1$	$R^2 = H$	$R^2 = R^1$	$R^2 = H$	$R^2 = R^1$	$R^2 = H$
NH_2	2.61	2.59	1.67	1.87	0.74	0.72
OMe	2.63	2.63	1.67	1.70	0.96	0.93
OH	2.76	2.72	1.68	1.71	1.08	1.01
Me	2.55	2.58	1.86	1.86	0.69	0.72
i-Pr	2.36	2.66	1.99	1.80	0.37	0.86
H	2.36	2.36	2.02	2.02	0.34	0.34
F	2.84	2.36	1.72	2.01	1.12	0.35
COOH	2.74	2.61	1.76	1.84	0.98	0.77
CF_3	2.36	2.36	2.04	2.03	0.32	0.33
CHO	2.67	2.61	1.83	1.85	0.84	0.76
CN	2.23	2.64	2.08	1.84	0.15	0.80
NO_2	2.55	3.20	2.42	1.73	0.13	1.47

Table S6. Activation energy Ea, standard activation Gibbs energy Δ‡G°, standard activation enthalpy Δ‡H°, and standard activation entropy TΔ‡S° for the Diels-Alder reactions of butadiene with the substituted fragment **1**. Data for the reaction with the non-substituted fragment (H) are given as reference.

Substituent R^1	Ea (kcal·mol^{-1})		Δ‡G° (kcal·mol^{-1})		Δ‡H° (kcal·mol^{-1})		TΔ‡S° (kcal·mol^{-1})	
	$R^2 = R^1$	R^2 = H	$R^2 = R^1$	R^2 = H	$R^2 = R^1$	R^2 = H	$R^2 = R^1$	R^2 = H
NH_2	38.94	31.29	53.03	45.14	37.73	30.15	−15.29	−14.99
OMe	34.82	32.51	49.55	46.44	33.59	31.55	−15.97	−14.90
OH	35.06	31.24	49.16	45.05	33.85	30.13	−15.31	−14.92
Me	40.37	35.51	54.30	49.08	39.31	34.68	−14.99	−14.39
i-Pr	54.40	36.88	68.53	50.79	53.41	36.09	−15.12	−14.70
H	33.11	33.11	46.03	46.03	32.47	32.47	−13.56	−13.56
F	33.50	34.23	47.08	47.03	32.73	33.67	−14.35	−13.36
COOH	35.98	32.61	50.39	46.25	35.07	31.90	−15.32	−14.35
CF_3	37.65	34.10	51.87	47.79	36.80	33.40	−15.06	−14.39
CHO	35.96	32.58	49.66	45.98	35.16	31.88	−14.51	−14.10
CN	38.52	33.64	51.80	46.77	37.87	33.03	−13.93	−13.74
NO_2	32.79	28.71	47.19	42.52	31.88	27.90	−15.31	−14.62

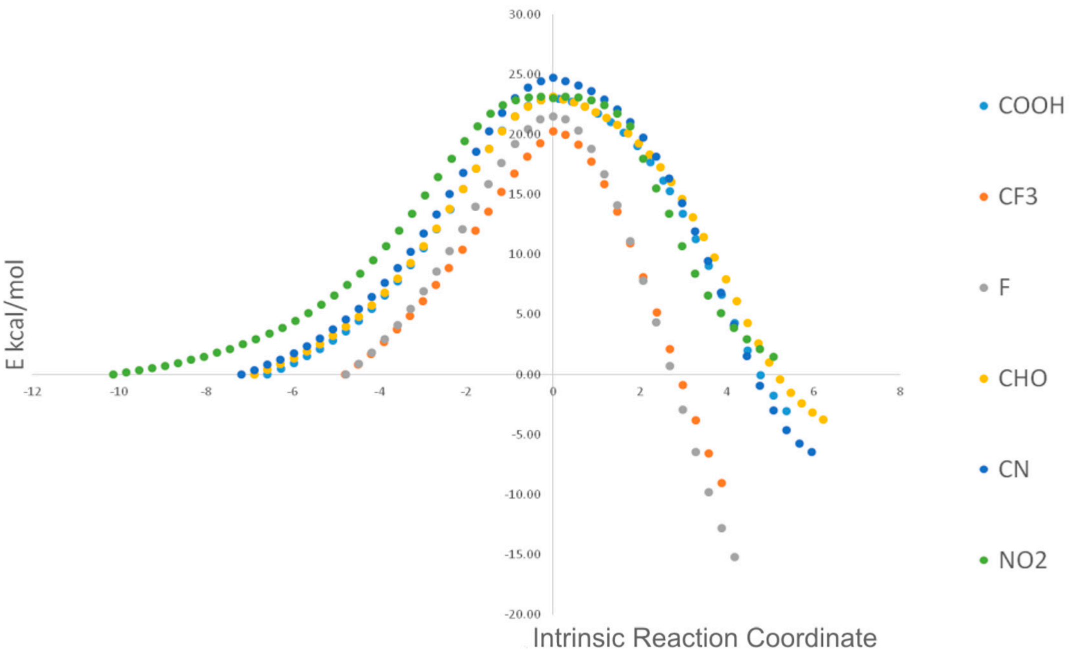

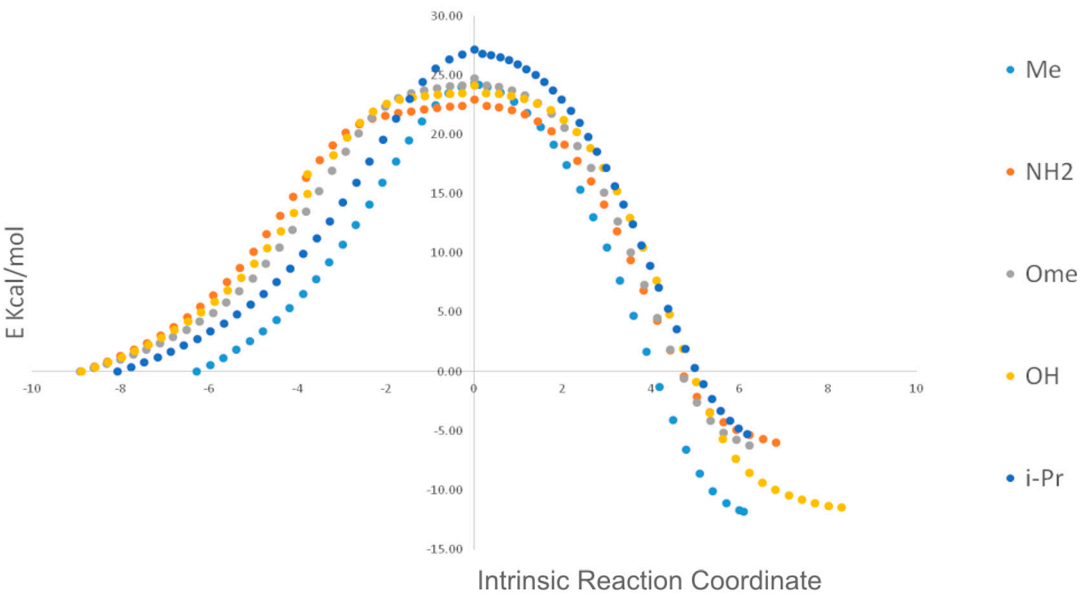

Figure S8. Intrinsic Reaction Coordinate for the reaction of 1,3-butadiene with mono-substituted fragment **1**.

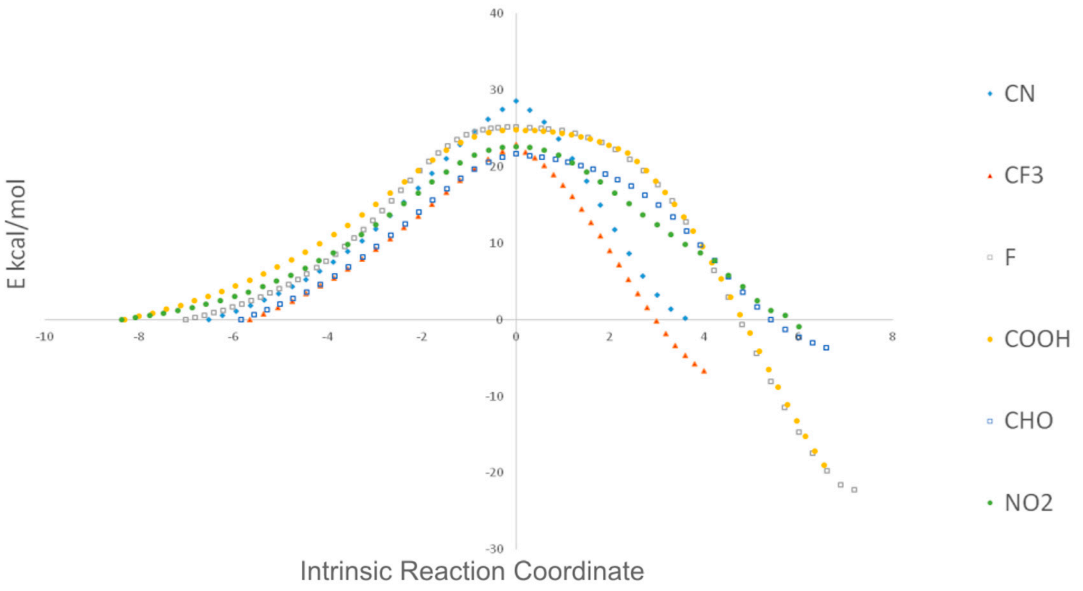

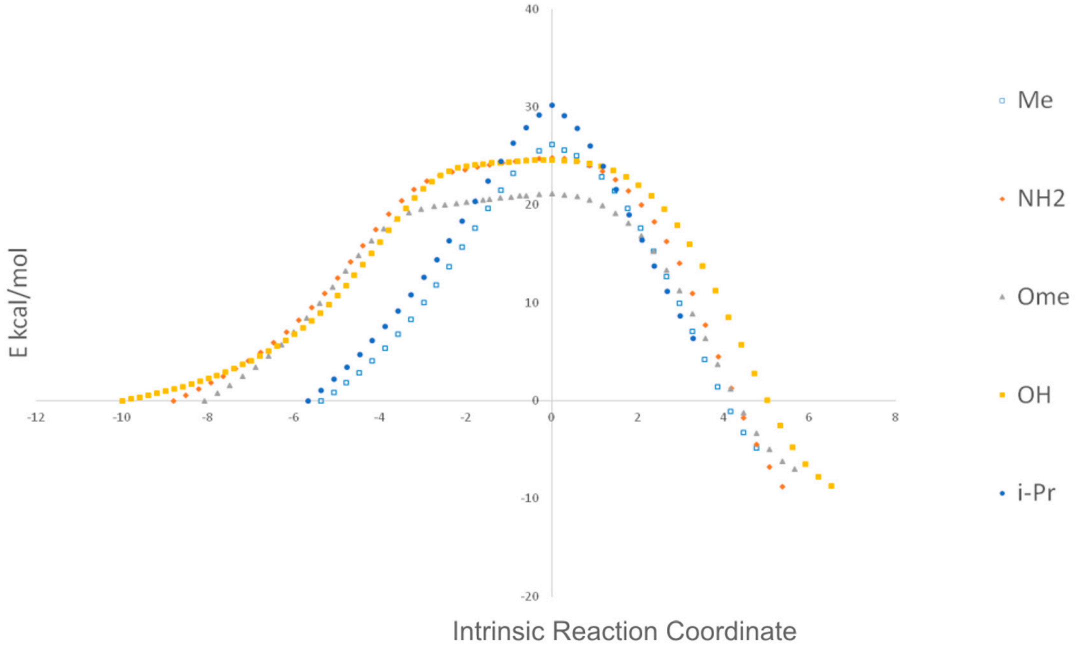

Figure S9. Intrinsic Reaction Coordinate for the reaction of 1,3-butadiene with di-substituted fragment **1**.

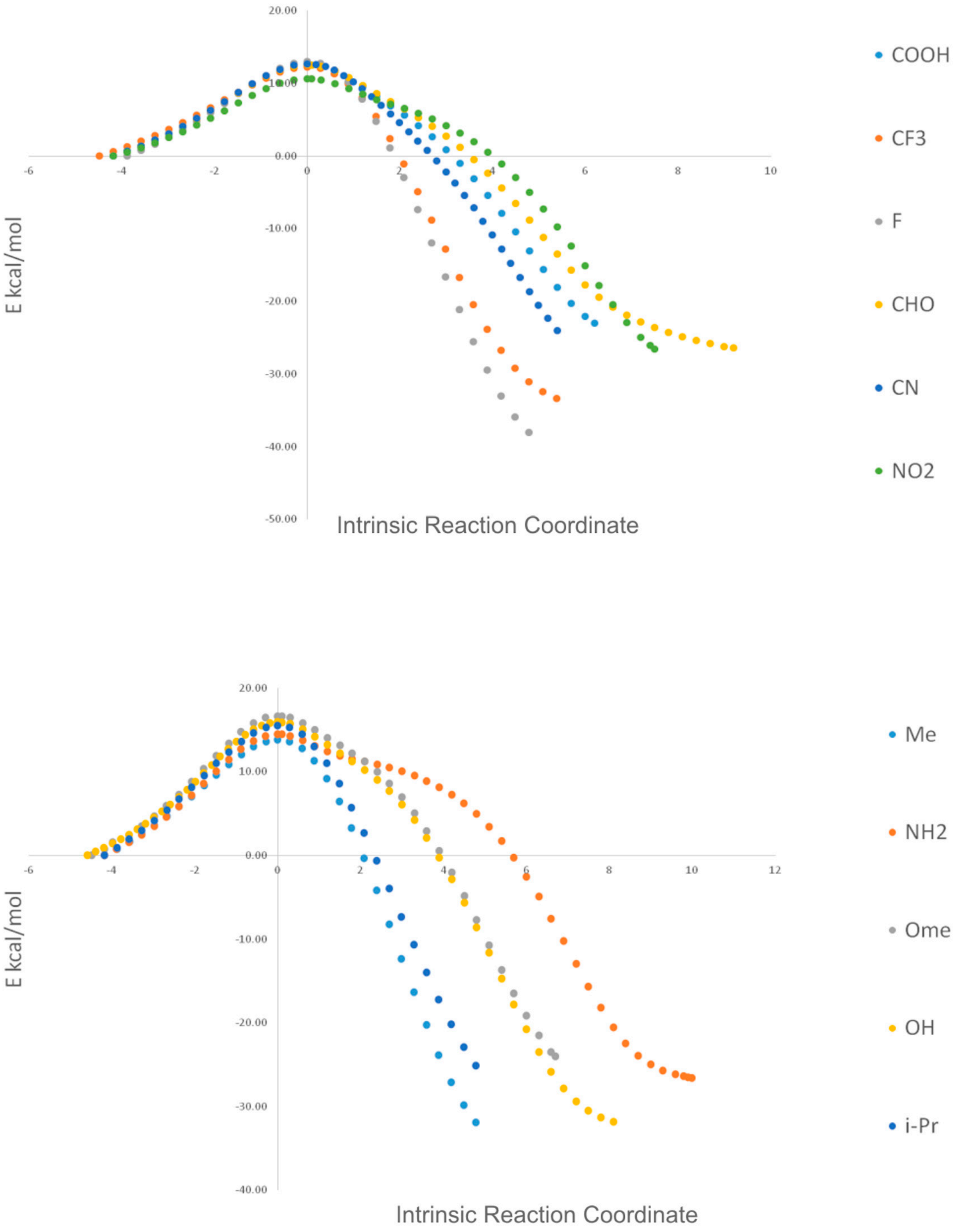

Figure S10. Intrinsic Reaction Coordinate for the reaction of 1,3-butadiene with mono-substituted fragment **2**.

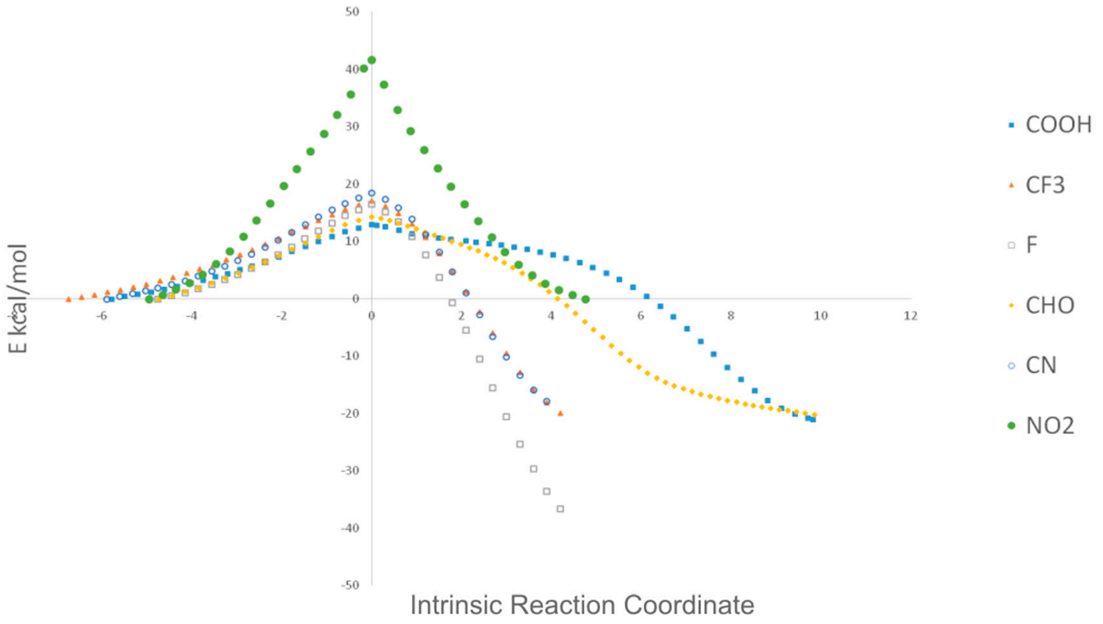

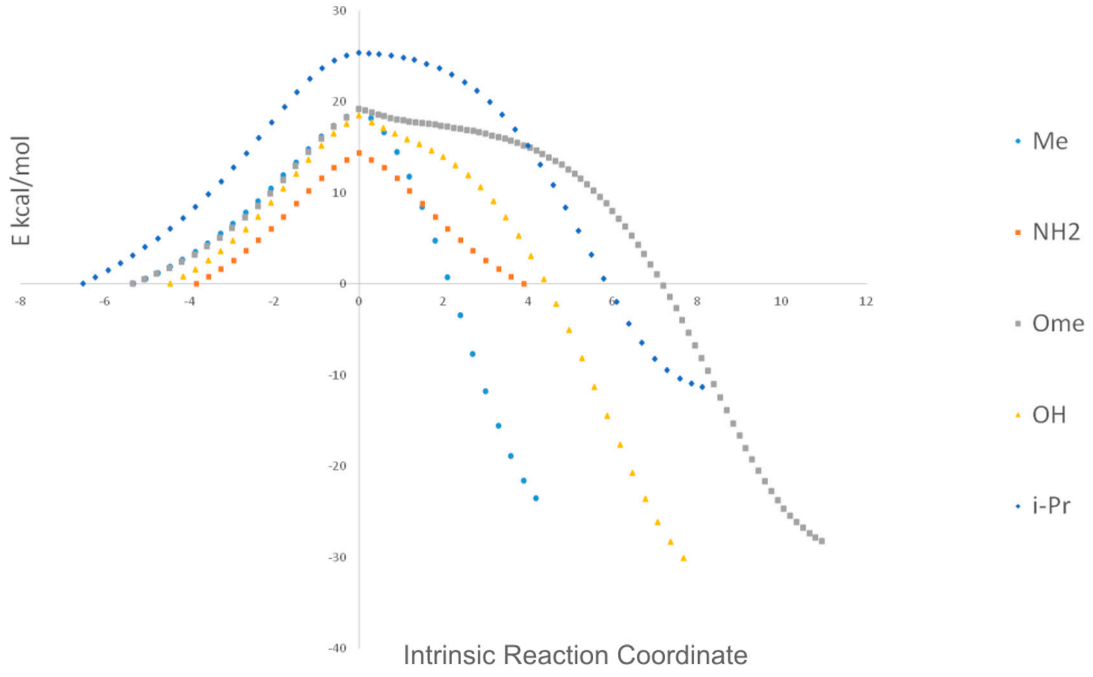

Figure S11. Intrinsic Reaction Coordinate for the reaction of 1,3-butadiene with di-substituted fragment **2**.

Fragment 1

```
Molecular formula: C30H12
Charge = 0 Multiplicity = 1
                                                  Hartree/particle
Thermal correction to Energy= 0.332792
Thermal correction to Enthalpy= 0.333736
Thermal correction to Gibbs Free Energy= 0.273526
Sum of electronic and zero-point Energies= -1150.083705
Sum of electronic and thermal Energies= -1150.066947
Sum of electronic and thermal Enthalpies= -1150.066003
Sum of electronic and thermal Free Energies= -1150.126213

E (Thermal) KCal/Mol CV Cal/Mol-Kelvin S Cal/Mol-Kelvin
 208.830 77.395 126.723


 Cartesian coordinates

Atom X Y Z
 C -1.297727 -0.504713 -1.324324
 C -1.082265 0.905737 -1.312770
 C 0.211558 1.376136 -1.325430
 C 1.325136 0.483986 -1.313755
 C 1.085804 -0.871716 -1.325130
 C -0.243607 -1.389970 -1.311253
 C 2.406165 1.052915 -0.547839
 C 3.302233 0.179565 0.054601
 C 3.056302 -1.253963 0.070946
 C 1.897092 -1.737282 -0.571430
 C 3.678563 -2.188937 0.943734
 C 3.059574 -3.400721 1.221635
 C 1.764289 -3.734658 0.742442
 C 1.142516 -2.878878 -0.148395
 C -0.291047 -2.607928 -0.541360
 C -1.495784 -2.947461 0.060062
 C -2.615134 -2.018597 0.072353
 C -2.453983 -0.773961 -0.571986
 C -3.066006 0.450874 -0.151234
 C -4.118835 0.341635 0.738875
 C -4.477207 -0.946474 1.219594
 C -3.736991 -2.088767 0.943948
 C 0.556478 2.511620 -0.572282
 C -0.440924 3.271637 0.074141
 C -1.805706 2.768924 0.057703
 C -2.114651 1.556658 -0.545263
 C 0.058526 4.276223 0.948552
 C 1.417859 4.346206 1.224113
 C 2.354608 3.393681 0.740456
 C 1.923205 2.428849 -0.151667
 H 4.082100 0.556490 0.709534
 H 4.604849 -1.934185 1.448158
 H 3.545519 -4.086661 1.906669
 H 1.267288 -4.615444 1.134267
 H -1.560575 -3.811829 0.714113
 H -4.633942 1.212866 1.128690
 H -5.315016 -1.023718 1.903758
 H -3.979909 -3.017536 1.449613
 H -2.521789 3.256338 0.712629
 H -0.625018 4.948161 1.457077
 H 1.769647 5.108261 1.910776
 H 3.366599 3.403534 1.130490
```

Fragment 1 substituted with one NH_2 group

Molecular formula: C30H13N
Charge = 0 Multiplicity = 1

Hartree/particle

Thermal correction to Energy= 0.350028
Thermal correction to Enthalpy= 0.350972
Thermal correction to Gibbs Free Energy= 0.287968
Sum of electronic and zero-point Energies= -1205.425543
Sum of electronic and thermal Energies= -1205.407307
Sum of electronic and thermal Enthalpies= -1205.406363
Sum of electronic and thermal Free Energies= -1205.469367

E (Thermal) KCal/Mol CV Cal/Mol-Kelvin S Cal/Mol-Kelvin
219.646 83.408 132.603

Cartesian coordinates

Atom X Y Z
C -1.181905 1.241735 -1.319929
C 0.238482 1.182979 -1.462992
C 0.839948 -0.054590 -1.555559
C 0.075840 -1.259820 -1.486927
C -1.293207 -1.167811 -1.355024
C -1.948898 0.097744 -1.253030
C 0.833189 -2.288202 -0.814692
C 0.124673 -3.288778 -0.157987
C -1.319767 -3.198928 0.010936
C -1.986384 -2.082711 -0.542146
C -2.094264 -3.939577 0.948226
C -3.334003 -3.462135 1.359932
C -3.851823 -2.199938 0.957027
C -3.158305 -1.465905 0.008977
C -3.083448 -0.002242 -0.367155
C -3.488530 1.150572 0.298589
C -2.680554 2.361812 0.258531
C -1.492618 2.346103 -0.506367
C -0.300260 3.079771 -0.193095
C -0.431653 4.100802 0.733384
C -1.697849 4.310182 1.347263
C -2.780153 3.456360 1.163809
C 2.081258 -0.290477 -0.942530
C 2.797092 0.764117 -0.333891
C 2.150097 2.068326 -0.255571
C 0.854570 2.259792 -0.726675
C 3.933450 0.363280 0.416217
C 4.197069 -0.994574 0.638533
C 3.278049 -2.012985 0.221672
C 2.188647 -1.666108 -0.551805
H 0.647238 -4.042363 0.427329
H -1.693669 -4.845436 1.395977
H -3.894975 -4.036169 2.091999
H -4.741651 -1.812736 1.445725
H -4.289367 1.111487 1.033729
H 0.415877 4.702775 1.049537
H -1.793522 5.124669 2.059749
H -3.676160 3.590812 1.764326
H 2.620768 2.818404 0.376287
H 3.429126 -3.028496 0.580793
N 5.354885 -1.387222 1.313934
H 5.788006 -0.670729 1.880547
H 5.291000 -2.268527 1.805163
H 4.570611 1.108512 0.887051

Fragment 1 substituted with one OMe group

Molecular formula: C31H14O
Charge = 0 Multiplicity = 1

Hartree/particle
Thermal correction to Energy= 0.367124
Thermal correction to Enthalpy= 0.368069
Thermal correction to Gibbs Free Energy= 0.301971
Sum of electronic and zero-point Energies= -1264.575106
Sum of electronic and thermal Energies= -1264.555693
Sum of electronic and thermal Enthalpies= -1264.554749
Sum of electronic and thermal Free Energies= -1264.620847

E (Thermal) KCal/Mol CV Cal/Mol-Kelvin S Cal/Mol-Kelvin
230.374 86.586 139.115

Cartesian coordinates

Atom	X	Y	Z
C	-1.235628	1.270262	-1.323159
C	0.176081	1.243715	-1.538419
C	0.798306	0.022222	-1.679771
C	0.066761	-1.200361	-1.584877
C	-1.295193	-1.140919	-1.379360
C	-1.972179	0.107973	-1.228758
C	0.882151	-2.216211	-0.962718
C	0.231804	-3.238694	-0.279656
C	-1.203495	-3.184549	-0.035076
C	-1.923800	-2.079337	-0.540907
C	-1.910401	-3.951831	0.933735
C	-3.137257	-3.507168	1.414797
C	-3.704089	-2.253565	1.053130
C	-3.078498	-1.495021	0.077536
C	-3.056246	-0.026483	-0.286022
C	-3.450261	1.110146	0.413131
C	-2.673456	2.340355	0.343853
C	-1.527395	2.359614	-0.482394
C	-0.337824	3.118516	-0.224332
C	-0.443458	4.127489	0.718498
C	-1.680048	4.301270	1.399897
C	-2.750361	3.423649	1.264666
C	2.075169	-0.191524	-1.130622
C	2.798254	0.876721	-0.551206
C	2.127868	2.167123	-0.426428
C	0.806018	2.330428	-0.824814
C	3.978239	0.495148	0.131870
C	4.272610	-0.859386	0.324740
C	3.372199	-1.901091	-0.047080
C	2.233276	-1.564021	-0.762826
H	0.801194	-3.984155	0.271402
H	-1.466111	-4.852109	1.350339
H	-3.645578	-4.100755	2.169325
H	-4.575218	-1.891491	1.592118
H	-4.209511	1.045302	1.189415
H	0.405602	4.745876	0.996303
H	-1.756711	5.106370	2.125150
H	-3.616208	3.531231	1.912871
H	2.616066	2.921784	0.185950
H	3.546532	-2.916192	0.289834
O	5.446376	-1.099721	0.986457
C	5.809836	-2.438565	1.279736
H	6.793917	-2.385183	1.747771
H	5.107757	-2.909981	1.979904
H	5.876087	-3.050922	0.371414
H	4.640458	1.226457	0.584411

Fragment 1 substituted with one OH group

Molecular formula: C30H12O
Charge = 0 Multiplicity = 1
Hartree/particle
Thermal correction to Energy= 0.337274
Thermal correction to Enthalpy= 0.338219
Thermal correction to Gibbs Free Energy= 0.275539
Sum of electronic and zero-point Energies= -1225.299321
Sum of electronic and thermal Energies= -1225.281305
Sum of electronic and thermal Enthalpies= -1225.280361
Sum of electronic and thermal Free Energies= -1225.343041

E (Thermal) KCal/Mol CV Cal/Mol-Kelvin S Cal/Mol-Kelvin
211.643 82.163 131.921

Cartesian coordinates

Atom X Y Z
C -1.232684 1.239518 -1.350418
C 0.184885 1.201158 -1.522097
C 0.804986 -0.026784 -1.609077
C 0.064190 -1.243234 -1.502763
C -1.302909 -1.172070 -1.340394
C -1.977516 0.083718 -1.245335
C 0.854151 -2.246226 -0.829850
C 0.178393 -3.246483 -0.138600
C -1.263014 -3.178274 0.061774
C -1.961371 -2.083770 -0.495588
C -2.002458 -3.914604 1.030308
C -3.240299 -3.450532 1.462235
C -3.789053 -2.204887 1.048818
C -3.130796 -1.476913 0.071475
C -3.090173 -0.019377 -0.332513
C -3.499544 1.138508 0.321616
C -2.714563 2.362793 0.241006
C -1.544027 2.353575 -0.550297
C -0.358575 3.114055 -0.277967
C -0.487062 4.149495 0.632726
C -1.742683 4.348022 1.271317
C -2.813276 3.472011 1.128305
C 2.063764 -0.230050 -1.017444
C 2.774807 0.851009 -0.448312
C 2.108186 2.146248 -0.380392
C 0.798471 2.304120 -0.821500
C 3.937783 0.487246 0.279185
C 4.212331 -0.859608 0.515745
C 3.318855 -1.908156 0.147580
C 2.202399 -1.596178 -0.608200
H 0.726987 -3.979917 0.448473
H -1.575870 -4.804932 1.485014
H -3.774008 -4.020041 2.217761
H -4.673732 -1.823798 1.551386
H -4.282497 1.099269 1.075740
H 0.3565060 4.771654 0.918703
H -1.836713 5.173524 1.971111
H -3.697651 3.601857 1.746715
H 2.582827 2.916461 0.223150
H 3.496154 -2.911415 0.531010
O 5.351650 -1.138054 1.222842
H 5.422160 -2.092852 1.349472
H 4.589802 1.230595 0.726622

Fragment 1 substituted with one Me group

Molecular formula: C31H14
Charge = 0 Multiplicity = 1

Hartree/particle

Thermal correction to Energy= 0.361318
Thermal correction to Enthalpy= 0.362262
Thermal correction to Gibbs Free Energy= 0.298181
Sum of electronic and zero-point Energies= -1189.377335
Sum of electronic and thermal Energies= -1189.358806
Sum of electronic and thermal Enthalpies= -1189.357862
Sum of electronic and thermal Free Energies= -1189.421944

E (Thermal) KCal/Mol CV Cal/Mol-Kelvin S Cal/Mol-Kelvin
226.731 83.364 134.871

Cartesian coordinates

Atom	X	Y	Z
C	-1.207174	1.241183	-1.330697
C	0.214144	1.194210	-1.464549
C	0.828055	-0.037830	-1.550610
C	0.077221	-1.250367	-1.470801
C	-1.293554	-1.171043	-1.343351
C	-1.960524	0.088771	-1.252899
C	0.843323	-2.264530	-0.787113
C	0.142078	-3.266785	-0.124249
C	-1.303386	-3.189828	0.039980
C	-1.980051	-2.085277	-0.524393
C	-2.072828	-3.929643	0.982268
C	-3.317944	-3.460616	1.387128
C	-3.847354	-2.207138	0.972008
C	-3.159179	-1.475094	0.018683
C	-3.097576	-0.014244	-0.370368
C	-3.515908	1.141558	0.281769
C	-2.721940	2.361739	0.230855
C	-1.531697	2.351100	-0.530405
C	-0.348739	3.101957	-0.222689
C	-0.494560	4.131535	0.692185
C	-1.764706	4.332913	1.300510
C	-2.836661	3.464662	1.123987
C	2.067552	-0.253787	-0.923904
C	2.770825	0.817922	-0.334703
C	2.114669	2.116505	-0.271796
C	0.816442	2.289098	-0.743598
C	3.913043	0.428839	0.421669
C	4.188458	-0.914756	0.671601
C	3.284159	-1.943828	0.259437
C	2.190217	-1.625482	-0.523628
H	0.670625	-4.009383	0.469515
H	-1.664291	-4.827259	1.439210
H	-3.874660	-4.033365	2.123381
H	-4.741805	-1.824344	1.455664
H	-4.318515	1.101800	1.014967
H	0.344986	4.746644	1.004201
H	-1.871391	5.153824	2.003910
H	-3.735560	3.595212	1.720946
H	2.582296	2.879965	0.346128
H	3.445079	-2.953769	0.629589
C	5.410928	-1.306144	1.470265
H	6.149932	-1.814723	0.839012
H	5.899098	-0.433146	1.911455
H	5.155219	-1.996417	2.281951
H	4.549324	1.188423	0.869946

Fragment 1 substituted with one *i*-Pr group

```
Molecular formula: C33H18
Charge = 0 Multiplicity = 1
                                                  Hartree/particle
Thermal correction to Energy= 0.420864
Thermal correction to Enthalpy= 0.421808
Thermal correction to Gibbs Free Energy= 0.351429
Sum of electronic and zero-point Energies= -1267.951792
Sum of electronic and thermal Energies= -1267.930592
Sum of electronic and thermal Enthalpies= -1267.929647
Sum of electronic and thermal Free Energies= -1268.000027

E (Thermal) KCal/Mol CV Cal/Mol-Kelvin S Cal/Mol-Kelvin
 264.096 93.604 148.126
```

```
 Cartesian coordinates

Atom X Y Z
 C -1.236467 1.265149 -1.240615
 C 0.183188 1.222130 -1.389888
 C 0.795267 -0.006036 -1.524075
 C 0.044989 -1.220243 -1.478599
 C -1.324510 -1.145050 -1.335212
 C -1.989896 0.110863 -1.195173
 C 0.817589 -2.256847 -0.836439
 C 0.121987 -3.281181 -0.201843
 C -1.321961 -3.209978 -0.021801
 C -2.003639 -2.086765 -0.541505
 C -2.082823 -3.981851 0.901540
 C -3.323902 -3.526983 1.334025
 C -3.856630 -2.259800 0.967584
 C -3.177151 -1.495437 0.033547
 C -3.118237 -0.022009 -0.305459
 C -3.528895 1.110673 0.390547
 C -2.734817 2.331800 0.373735
 C -1.552312 2.347127 -0.399421
 C -0.366361 3.087497 -0.078535
 C -0.502796 4.085724 0.871884
 C -1.766630 4.265987 1.499604
 C -2.840303 3.403756 1.304874
 C 2.040112 -0.243886 -0.914518
 C 2.750801 0.807367 -0.296128
 C 2.095538 2.104807 -0.185096
 C 0.793517 2.292756 -0.637926
 C 3.896972 0.394659 0.436939
 C 4.176576 -0.958935 0.642182
 C 3.270137 -1.969457 0.199097
 C 2.165773 -1.625783 -0.562530
 H 0.655633 -4.043454 0.361670
 H -1.670332 -4.894812 1.323180
 H -3.873941 -4.124863 2.055163
 H -4.746279 -1.893810 1.472615
 H -4.324086 1.045799 1.130014
 H 0.339933 4.690193 1.195845
 H -1.866146 5.062651 2.231372
 H -3.733071 3.513747 1.915032
 H 2.569120 2.847052 0.453836
 H 3.437284 -2.993077 0.525978
 C 5.412132 -1.378748 1.435614
 H 5.396910 -2.474860 1.493195
 C 5.391304 -0.846752 2.880841
 H 5.438566 0.247330 2.905483
 H 4.479619 -1.152570 3.403127
 H 6.250924 -1.226571 3.443702
 C 6.713336 -0.977279 0.716121
 H 6.802199 0.111650 0.636666
 H 7.587970 -1.344002 1.264434
 H 6.748809 -1.388409 -0.297334
 H 4.531277 1.145043 0.902450
```

Fragment 1 substituted with one F group

Molecular formula: C30H11F
Charge = 0 Multiplicity = 1
Hartree/particle
Thermal correction to Energy= 0.324600
Thermal correction to Enthalpy= 0.325544
Thermal correction to Gibbs Free Energy= 0.263507
Sum of electronic and zero-point Energies= -1249.323965
Sum of electronic and thermal Energies= -1249.306372
Sum of electronic and thermal Enthalpies= -1249.305428
Sum of electronic and thermal Free Energies= -1249.367465

E (Thermal) KCal/Mol CV Cal/Mol-Kelvin S Cal/Mol-Kelvin
203.690 80.350 130.569

Cartesian coordinates

Atom X Y Z
C -1.204229 1.232581 -1.335716
C 0.215604 1.179869 -1.479982
C 0.825019 -0.054080 -1.560479
C 0.070771 -1.263693 -1.470423
C -1.298266 -1.178834 -1.331940
C -1.960860 0.083498 -1.244457
C 0.837887 -2.277501 -0.788780
C 0.140091 -3.272772 -0.111972
C -1.303549 -3.189680 0.063705
C -1.980737 -2.085963 -0.501662
C -2.066913 -3.921035 1.017410
C -3.306897 -3.445055 1.429575
C -3.835551 -2.192464 1.010754
C -3.153399 -1.468529 0.046947
C -3.090822 -0.010366 -0.352070
C -3.499965 1.150929 0.296131
C -2.702646 2.368228 0.230395
C -1.518728 2.349047 -0.540401
C -0.331595 3.098824 -0.247423
C -0.466297 4.134723 0.661867
C -1.730901 4.344029 1.278890
C -2.806625 3.477632 1.116834
C 2.069943 -0.270404 -0.944656
C 2.781650 0.806968 -0.369377
C 2.129052 2.108167 -0.310826
C 0.826890 2.277871 -0.770545
C 3.932766 0.428160 0.376220
C 4.162952 -0.918962 0.603129
C 3.290230 -1.971153 0.230221
C 2.187934 -1.642602 -0.539879
H 0.671588 -4.013914 0.480829
H -1.657449 -4.817020 1.476500
H -3.859247 -4.010914 2.174279
H -4.724678 -1.803818 1.499444
H -4.296640 1.118691 1.036053
H 0.377606 4.749277 0.962814
H -1.829573 5.169855 1.977557
H -3.700273 3.615088 1.720009
H 2.604502 2.875101 0.296109
H 3.483169 -2.965690 0.618638
F 5.251385 -1.264501 1.328908
H 4.593201 1.156967 0.834338

Fragment 1 substituted with one COOH group

Molecular formula: C31H12O2
Charge = 0 Multiplicity = 1

Hartree/particle

Thermal correction to Energy= 0.349992
Thermal correction to Enthalpy= 0.350936
Thermal correction to Gibbs Free Energy= 0.283834
Sum of electronic and zero-point Energies= -1338.646976
Sum of electronic and thermal Energies= -1338.627331
Sum of electronic and thermal Enthalpies= -1338.626386
Sum of electronic and thermal Free Energies= -1338.693489

E (Thermal) KCal/Mol CV Cal/Mol-Kelvin S Cal/Mol-Kelvin
219.623 87.424 141.229

Cartesian coordinates

Atom X Y Z
C -2.045402 1.213422 -1.313051
C -0.655302 1.149523 -1.628804
C -0.072267 -0.089530 -1.800111
C -0.819253 -1.294394 -1.622512
C -2.160327 -1.199020 -1.315447
C -2.793089 0.067947 -1.135104
C 0.017080 -2.319947 -1.049438
C -0.602451 -3.316054 -0.300713
C -2.012412 -3.222554 0.052252
C -2.744003 -2.107826 -0.415082
C -2.659828 -3.957059 1.086173
C -3.835098 -3.474858 1.651930
C -4.399282 -2.213378 1.313331
C -3.833842 -1.485734 0.279426
C -3.805983 -0.024353 -0.111586
C -4.120544 1.135203 0.590424
C -3.328355 2.347772 0.434681
C -2.249716 2.326478 -0.477526
C -1.030289 3.066379 -0.329974
C -1.043218 4.096544 0.595709
C -2.219569 4.309447 1.366728
C -3.313157 3.450846 1.334771
C 1.233222 -0.318918 -1.335887
C 2.021882 0.750248 -0.854668
C 1.395607 2.058512 -0.707959
C 0.047893 2.241456 -0.997834
C 3.250839 0.355353 -0.264438
C 3.529232 -1.000343 -0.065940
C 2.580249 -2.032511 -0.343726
C 1.392855 -1.696076 -0.961648
H -0.008639 -4.066933 0.215533
H -2.205628 -4.861178 1.482860
H -4.297149 -4.043832 2.453551
H -5.217708 -1.822261 1.911314
H -4.819581 1.102797 1.423238
H -0.164002 4.703469 0.793114
H -2.224418 5.130650 2.077634
H -4.123658 3.589387 2.045477
H 1.953819 2.818317 -0.165808
H 2.814002 -3.035738 -0.003716
C 4.802750 -1.431251 0.567556
O 5.091852 -2.581994 0.834700
O 5.650577 -0.403005 0.832416
H 6.432071 -0.810364 1.240654
H 3.957620 1.095832 0.093967

Fragment 1 substituted with one CF_3 group

Molecular formula: C31H11F3
Charge = 0 Multiplicity = 1

Hartree/particle

Thermal correction to Energy= 0.340468
Thermal correction to Enthalpy= 0.341412
Thermal correction to Gibbs Free Energy= 0.272006
Sum of electronic and zero-point Energies= -1487.116059
Sum of electronic and thermal Energies= -1487.095653
Sum of electronic and thermal Enthalpies= -1487.094708
Sum of electronic and thermal Free Energies= -1487.164114

E (Thermal) KCal/Mol CV Cal/Mol-Kelvin S Cal/Mol-Kelvin
213.647 89.920 146.077

Cartesian coordinates

Atom X Y Z
C 2.088414 -1.050182 -1.263137
C 0.714272 -1.327799 -1.530559
C -0.148815 -0.269261 -1.724426
C 0.297759 1.083590 -1.616845
C 1.631027 1.318775 -1.355005
C 2.550197 0.244722 -1.154218
C -0.740445 1.907319 -1.048245
C -0.354001 3.051519 -0.357114
C 1.047262 3.307440 -0.053607
C 2.007945 2.376637 -0.508716
C 1.530958 4.216551 0.929712
C 2.801960 4.048261 1.468192
C 3.638517 2.941541 1.152707
C 3.232772 2.058128 0.166247
C 3.540293 0.615859 -0.172513
C 4.139143 -0.406293 0.557624
C 3.650172 -1.776040 0.474910
C 2.571784 -2.047033 -0.396566
C 1.565433 -3.044747 -0.178100
C 1.845890 -4.002458 0.782342
C 3.060499 -3.900943 1.515916
C 3.920252 -2.812684 1.412919
C -1.460117 -0.333132 -1.223626
C -1.958948 -1.533320 -0.672517
C -1.039060 -2.649669 -0.500593
C 0.305454 -2.524427 -0.834194
C -3.231099 -1.411260 -0.049029
C -3.805488 -0.154846 0.114069
C -3.140370 1.058613 -0.233089
C -1.927390 0.982762 -0.889720
H -1.093615 3.663006 0.154848
H 0.887387 5.004668 1.311515
H 3.138597 4.743289 2.231988
H 4.542452 2.779702 1.733275
H 4.834241 -0.175253 1.361803
H 1.140081 -4.789157 1.033762
H 3.278311 -4.666998 2.254472
H 4.760390 -2.727397 2.097091
H -1.386662 -3.493868 0.090345
H -3.570022 2.002052 0.087595
C -5.164959 -0.036901 0.750354
F -5.562065 -1.182385 1.346211
F -6.113629 0.285584 -0.160801
F -5.192525 0.936374 1.692663
H -3.733062 -2.280543 0.361616

Fragment 1 substituted with one CHO group

Molecular formula: C31H12O
Charge = 0 Multiplicity = 1

Hartree/particle

Thermal correction to Energy= 0.343395
Thermal correction to Enthalpy= 0.344340
Thermal correction to Gibbs Free Energy= 0.279544
Sum of electronic and zero-point Energies= -1263.400880
Sum of electronic and thermal Energies= -1263.382126
Sum of electronic and thermal Enthalpies= -1263.381181
Sum of electronic and thermal Free Energies= -1263.445977

E (Thermal) KCal/Mol CV Cal/Mol-Kelvin S Cal/Mol-Kelvin
215.484 83.976 136.373

Cartesian coordinates

Atom	X	Y	Z
C	1.598977	-0.971084	-1.271930
C	0.236939	-1.360032	-1.441082
C	-0.718790	-0.374911	-1.587122
C	-0.374882	1.010740	-1.527435
C	0.950363	1.353976	-1.360970
C	1.962171	0.358112	-1.208974
C	-1.435390	1.762176	-0.903901
C	-1.095047	2.945574	-0.255523
C	0.298817	3.313734	-0.048537
C	1.298052	2.452670	-0.555168
C	0.772717	4.273594	0.890385
C	2.085177	4.213015	1.345898
C	2.983919	3.169644	0.988277
C	2.585603	2.241494	0.040656
C	2.982740	0.822029	-0.300803
C	3.708041	-0.138136	0.398234
C	3.326310	-1.543478	0.363050
C	2.217345	-1.913056	-0.430164
C	1.311197	-2.984191	-0.133998
C	1.731862	-3.901630	0.814532
C	2.980652	-3.692212	1.462899
C	3.741032	-2.540465	1.291056
C	-1.982276	-0.532371	-0.996760
C	-2.348945	-1.764204	-0.407881
C	-1.333593	-2.802683	-0.288065
C	-0.027419	-2.575517	-0.708899
C	-3.586235	-1.727160	0.287239
C	-4.257025	-0.515253	0.472518
C	-3.709298	0.743057	0.067829
C	-2.533716	0.751522	-0.652588
H	-1.845960	3.507306	0.295346
H	0.095399	5.015466	1.304986
H	2.414955	4.944218	2.078252
H	3.933414	3.087709	1.509918
H	4.434644	0.159740	1.150933
H	1.110519	-4.738556	1.120867
H	3.308420	-4.427087	2.192452
H	4.615223	-2.377952	1.915986
H	-1.574459	-3.665066	0.329307
H	-4.218802	1.641746	0.401086
C	-5.527867	-0.525171	1.228231
H	-5.864997	-1.535467	1.550960
O	-6.182689	0.464854	1.498319
H	-3.995432	-2.629574	0.737339

Fragment 1 substituted with one CN group

```
Molecular formula: C31H11N
Charge = 0 Multiplicity = 1
                                                   Hartree/particle
Thermal correction to Energy= 0.332444
Thermal correction to Enthalpy= 0.333389
Thermal correction to Gibbs Free Energy= 0.268969
Sum of electronic and zero-point Energies= -1242.327866
Sum of electronic and thermal Energies= -1242.309293
Sum of electronic and thermal Enthalpies= -1242.308349
Sum of electronic and thermal Free Energies= -1242.372768

E (Thermal) KCal/Mol CV Cal/Mol-Kelvin S Cal/Mol-Kelvin
 208.612 83.358 135.582


 Cartesian coordinates

Atom X Y Z
 C 1.466879 -1.049333 -1.280654
 C 0.077924 -1.326004 -1.450924
 C -0.796089 -0.266957 -1.585090
 C -0.342527 1.085789 -1.510985
 C 1.005810 1.320062 -1.342373
 C 1.935718 0.245534 -1.204297
 C -1.338053 1.911663 -0.874667
 C -0.904240 3.056344 -0.212822
 C 0.514700 3.310939 -0.006717
 C 1.440904 2.378339 -0.525101
 C 1.065244 4.220701 0.940204
 C 2.369830 4.051283 1.390687
 C 3.181718 2.943284 1.020040
 C 2.708787 2.059252 0.064507
 C 2.991382 0.616551 -0.293717
 C 3.638757 -0.406056 0.393251
 C 3.145398 -1.775884 0.344167
 C 2.008832 -2.046646 -0.449818
 C 1.020970 -3.045163 -0.162927
 C 1.368124 -4.004056 0.774230
 C 2.630969 -3.902493 1.421152
 C 3.480699 -2.813377 1.259903
 C -2.068563 -0.329824 -0.994045
 C -2.529613 -1.532701 -0.412451
 C -1.599834 -2.650838 -0.305271
 C -0.281847 -2.523867 -0.728497
 C -3.755726 -1.413351 0.289878
 C -4.326715 -0.149101 0.488014
 C -3.681686 1.068620 0.094993
 C -2.511151 0.987716 -0.632478
 H -1.606242 3.670110 0.347038
 H 0.450260 5.010037 1.364112
 H 2.758658 4.746583 2.128947
 H 4.123094 2.781254 1.537573
 H 4.388287 -0.175405 1.147014
 H 0.682246 -4.791811 1.072865
 H 2.900343 -4.669313 2.141573
 H 4.366492 -2.728541 1.883809
 H -1.907360 -3.496146 0.305703
 H -4.099278 2.011204 0.433486
 C -5.561824 -0.058539 1.212196
 N -6.564693 0.021755 1.797327
 H -4.232301 -2.281582 0.734265
```

Fragment 1 substituted with one NO2 group

Molecular formula: C30H11NO2
Charge = 0 Multiplicity = 1
Hartree/particle
Thermal correction to Energy= 0.337170
Thermal correction to Enthalpy= 0.338115
Thermal correction to Gibbs Free Energy= 0.271542
Sum of electronic and zero-point Energies= -1354.582741
Sum of electronic and thermal Energies= -1354.563420
Sum of electronic and thermal Enthalpies= -1354.562476
Sum of electronic and thermal Free Energies= -1354.629049

E (Thermal) KCal/Mol CV Cal/Mol-Kelvin S Cal/Mol-Kelvin
211.578 85.828 140.115

Cartesian coordinates

Atom X Y Z
C -1.996410 1.228916 -1.311518
C -0.600193 1.168807 -1.595929
C -0.010887 -0.068387 -1.761263
C -0.758816 -1.275988 -1.604828
C -2.106073 -1.184232 -1.325645
C -2.744906 0.080880 -1.153788
C 0.065943 -2.302061 -1.017720
C -0.566509 -3.302146 -0.285020
C -1.983776 -3.213208 0.037177
C -2.706847 -2.097658 -0.441355
C -2.651182 -3.953068 1.054470
C -3.838916 -3.474834 1.596753
C -4.397947 -2.212960 1.250872
C -3.812513 -1.480300 0.231774
C -3.779542 -0.017491 -0.153211
C -4.111693 1.139014 0.545894
C -3.319705 2.354192 0.411516
C -2.221404 2.338854 -0.476801
C -1.008252 3.081913 -0.300395
C -1.043332 4.108455 0.628690
C -2.236778 4.315621 1.374414
C -3.326994 3.454134 1.315540
C 1.283677 -0.295781 -1.269106
C 2.058348 0.774838 -0.765660
C 1.427770 2.082353 -0.628214
C 0.086345 2.260941 -0.945559
C 3.273812 0.383464 -0.149579
C 3.526881 -0.971970 0.036308
C 2.609647 -2.018676 -0.252759
C 1.436849 -1.675052 -0.896007
H 0.017752 -4.053899 0.240668
H -2.203986 -4.857936 1.457106
H -4.316895 -4.047351 2.386284
H -5.229603 -1.825786 1.832835
H -4.828888 1.102233 1.362853
H -0.170242 4.716819 0.847220
H -2.259308 5.134158 2.087858
H -4.153141 3.588170 2.008761
H 1.973586 2.841435 -0.073270
H 2.841150 -3.018428 0.092501
O 4.996386 -2.540825 0.902860
O 5.578342 -0.443547 0.988816
N 4.792268 -1.344140 0.691250
H 3.987199 1.102306 0.235261

Fragment 2

Molecular formula: C30H10
Charge = 0 Multiplicity = 1

Hartree/particle

Thermal correction to Energy= 0.307666
Thermal correction to Enthalpy= 0.308610
Thermal correction to Gibbs Free Energy= 0.250112
Sum of electronic and zero-point Energies= -1148.792523
Sum of electronic and thermal Energies= -1148.776376
Sum of electronic and thermal Enthalpies= -1148.775432
Sum of electronic and thermal Free Energies= -1148.833930

E (Thermal) KCal/Mol CV Cal/Mol-Kelvin S Cal/Mol-Kelvin
193.063 75.571 123.120

Cartesian coordinates

Atom X Y Z
C 1.213429 -0.121090 1.521538
C 0.490188 1.116732 1.521531
C -0.910536 0.811348 1.521541
C -1.052951 -0.615231 1.521457
C 0.259685 -1.191490 1.521377
C 0.949599 2.162708 0.758602
C 0.049767 3.051981 0.048236
C -1.315222 2.754645 0.048352
C -1.763302 1.571364 0.758462
C -2.887511 0.990394 0.048720
C -3.026633 -0.399643 0.049068
C -2.039518 -1.191491 0.758775
C -1.834237 -2.439818 0.048344
C -0.555086 -3.001364 0.048017
C 0.502913 -2.307666 0.758376
C 1.753998 -2.498475 0.048761
C 2.683695 -1.455744 0.049361
C 2.350356 -0.234914 0.758921
C 2.213302 2.101832 0.048309
C 2.917850 0.895530 0.048324
C 0.900872 3.750147 -0.923546
C 2.152271 3.201769 -0.922535
C -2.379825 3.035617 -0.923055
C -3.288754 2.015531 -0.922989
C -3.624124 -1.325667 -0.921228
C -2.933583 -2.504617 -0.922935
C 0.140981 -3.853845 -0.924005
C 1.476009 -3.563238 -0.923391
C 3.710769 -1.057614 -0.921597
C 3.845692 0.301984 -0.922628
H 0.564549 4.527574 -1.595857
H 2.952779 3.480713 -1.594048
H -2.397373 3.882573 -1.595244
H -4.132308 1.935960 -1.595019
H -4.436084 -1.080778 -1.592243
H -3.118440 -3.331088 -1.595471
H -0.342920 -4.548544 -1.596869
H 2.205111 -3.993892 -1.596046
H 4.223140 -1.732804 -1.593252
H 4.480976 0.862242 -1.594948

Fragment 2 substituted with one NH_2 group

```
Molecular formula: C30H11N
Charge = 0 Multiplicity = 1
                                                  Hartree/particle
Thermal correction to Energy= 0.325372
Thermal correction to Enthalpy= 0.326316
Thermal correction to Gibbs Free Energy= 0.265089
Sum of electronic and zero-point Energies= -1204.139813
Sum of electronic and thermal Energies= -1204.122226
Sum of electronic and thermal Enthalpies= -1204.121282
Sum of electronic and thermal Free Energies= -1204.182509

E (Thermal) KCal/Mol CV Cal/Mol-Kelvin S Cal/Mol-Kelvin
 204.174 81.369 128.863
```

```
 Cartesian coordinates

Atom X Y Z
 C -0.722401 -0.000984 1.707659
 C 0.120469 -1.165435 1.632549
 C 1.474872 -0.720265 1.500948
 C 1.473927 0.715190 1.497923
 C 0.115152 1.159827 1.624642
 C -0.306146 -2.243229 0.891960
 C 0.609257 -3.044488 0.097325
 C 1.939724 -2.613952 -0.019314
 C 2.329917 -1.393209 0.654764
 C 3.323496 -0.701133 -0.148981
 C 3.320785 0.699057 -0.150592
 C 2.323583 1.387285 0.649025
 C 1.931263 2.611414 -0.028027
 C 0.605352 3.044481 0.096283
 C -0.307865 2.249677 0.896398
 C -1.635498 2.315872 0.312710
 C -2.456294 1.186932 0.416537
 C -1.930128 -0.000118 1.054764
 C -1.636805 -2.315000 0.308370
 C -2.455277 -1.179833 0.376285
 C -0.253746 -3.826199 -0.792469
 C -1.551041 -3.400358 -0.672796
 C 2.936881 -2.788951 -1.084942
 C 3.739671 -1.683365 -1.158851
 C 3.732785 1.682574 -1.161156
 C 2.929906 2.788535 -1.090199
 C -0.261204 3.821018 -0.800408
 C -1.557689 3.402174 -0.676054
 C -3.565271 0.692375 -0.428089
 C -3.553462 -0.689783 -0.445961
 H 0.095110 -4.567382 -1.501418
 H -2.378045 -3.756332 -1.275803
 H 2.976645 -3.632804 -1.763427
 H 4.510068 -1.519979 -1.902713
 H 4.503762 1.520374 -1.904891
 H 2.970663 3.632153 -1.768654
 H 0.088350 4.554027 -1.517552
 H -2.381485 3.744358 -1.289478
 N -4.360429 1.540841 -1.168573
 H -5.169861 1.123636 -1.606835
 H -4.559666 2.436656 -0.745620
 H -4.201245 -1.303657 -1.059971
```

Fragment 2 substituted with one OMe group

```
Molecular formula: C31H12O
Charge = 0 Multiplicity = 1
                                                  Hartree/particle
Thermal correction to Energy= 0.342257
Thermal correction to Enthalpy= 0.343202
Thermal correction to Gibbs Free Energy= 0.278452
Sum of electronic and zero-point Energies= -1263.283836
Sum of electronic and thermal Energies= -1263.264848
Sum of electronic and thermal Enthalpies= -1263.263904
Sum of electronic and thermal Free Energies= -1263.328654

E (Thermal) KCal/Mol CV Cal/Mol-Kelvin S Cal/Mol-Kelvin
 214.770 84.792 136.276
```

```
 Cartesian coordinates

Atom X Y Z
 C -0.902509 0.001918 1.657082
 C -0.057523 -1.159764 1.603299
 C 1.301904 -0.714664 1.510213
 C 1.300026 0.720348 1.506383
 C -0.062644 1.163142 1.596097
 C -0.460688 -2.243266 0.857925
 C 0.475939 -3.043395 0.087947
 C 1.806643 -2.611755 0.004203
 C 2.177532 -1.387576 0.687406
 C 3.193021 -0.697622 -0.089395
 C 3.189823 0.702494 -0.093085
 C 2.171043 1.391158 0.678756
 C 1.796027 2.613418 -0.011228
 C 0.466598 3.043734 0.075303
 C -0.466949 2.249058 0.852789
 C -1.779267 2.313700 0.235396
 C -2.609142 1.189129 0.322335
 C -2.096735 0.000858 0.976087
 C -1.772325 -2.313145 0.235246
 C -2.595253 -.182344 0.286607
 C -0.363758 -3.826466 -0.825940
 C -1.662815 -3.401941 -0.742449
 C 2.833570 -2.788680 -1.031871
 C 3.636671 -1.682216 -1.085305
 C 3.628728 1.684813 -1.093530
 C 2.823051 2.789844 -1.046159
 C -0.376697 3.816405 -0.845833
 C -1.675505 3.395444 -0.754734
 C -3.683019 0.675119 -0.557759
 C -3.666634 -0.697370 -0.580768
 H 0.004863 -4.568586 -1.523774
 H -2.474508 -3.758227 -1.365357
 H 2.893057 -3.633833 -1.707119
 H 4.427039 -1.519878 -1.808153
 H 4.419792 1.522081 -1.815657
 H 2.880966 3.632244 -1.724811
 H -0.009269 4.546922 -1.556467
 H -2.483569 3.734750 -1.390161
 O -4.480693 1.434435 -1.350436
 C -5.189180 2.491869 -0.694303
 H -5.704241 3.046917 -1.479756
 H -5.925619 2.084333 0.008197
 H -4.513311 3.162340 -0.153937
 H -4.300493 -1.301773 -1.216179
```

Fragment 2 substituted with one OH group

```
Molecular formula: C30H10O
Charge = 0 Multiplicity = 1
                                                  Hartree/particle
Thermal correction to Energy= 0.312579
Thermal correction to Enthalpy= 0.313524
Thermal correction to Gibbs Free Energy= 0.252498
Sum of electronic and zero-point Energies= -1224.009417
Sum of electronic and thermal Energies= -1223.992003
Sum of electronic and thermal Enthalpies= -1223.991059
Sum of electronic and thermal Free Energies= -1224.052084

E (Thermal) KCal/Mol CV Cal/Mol-Kelvin S Cal/Mol-Kelvin
 196.147 80.165 128.438
```

```
 Cartesian coordinates

Atom X Y Z
 C -0.713692 -0.015102 1.740359
 C 0.140625 -1.169801 1.663799
 C 1.487875 -0.710986 1.499158
 C 1.469398 0.724272 1.475619
 C 0.108144 1.154213 1.623245
 C -0.287370 -2.263733 0.948734
 C 0.620462 -3.064748 0.145812
 C 1.942065 -2.620775 -0.005029
 C 2.331668 -1.385245 0.644366
 C 3.301226 -0.693827 -0.189116
 C 3.282927 0.705776 -0.209456
 C 2.294213 1.393904 0.600917
 C 1.874090 2.603352 -0.085492
 C 0.545806 3.022764 0.059399
 C -0.341916 2.229225 0.888713
 C -1.680914 2.270512 0.330248
 C -2.486665 1.133521 0.469179
 C -1.936980 -0.039160 1.116426
 C -1.628394 -2.357894 0.392039
 C -2.460460 -1.234644 0.465020
 C -0.252466 -3.869115 -0.715461
 C -1.551450 -3.457588 -0.575085
 C 2.920474 -2.799899 -1.086951
 C 3.708207 -1.686093 -1.192666
 C 3.663446 1.679886 -1.241390
 C 2.849644 2.777623 -1.169329
 C -0.346134 3.773745 -0.833186
 C -1.634705 3.340553 -0.677542
 C -3.589588 0.602872 -0.352263
 C -3.575092 -0.770639 -0.357175
 H 0.090441 -4.616519 -1.420702
 H -2.387311 -3.831490 -1.154198
 H 2.957015 -3.652858 -1.754005
 H 4.461412 -1.524915 -1.954346
 H 4.421381 1.516228 -1.998009
 H 2.867458 3.612374 -1.859532
 H -0.020357 4.500489 -1.567517
 H -2.469804 3.673186 -1.282694
 O -4.438806 1.361428 -1.087004
 H -4.384884 2.276032 -0.780967
 H -4.230798 -1.387918 -0.956305
```

Fragment 2 substituted with one Me group

```
Molecular formula: C31H12
Charge = 0 Multiplicity = 1
                                                  Hartree/particle
Thermal correction to Energy= 0.336471
Thermal correction to Enthalpy= 0.337415
Thermal correction to Gibbs Free Energy= 0.275551
Sum of electronic and zero-point Energies= -1188.088599
Sum of electronic and thermal Energies= -1188.070799
Sum of electronic and thermal Enthalpies= -1188.069855
Sum of electronic and thermal Free Energies= -1188.131719

E (Thermal) KCal/Mol CV Cal/Mol-Kelvin S Cal/Mol-Kelvin
 211.139 81.391 130.204
```

```
 Cartesian coordinates

Atom X Y Z
 C -0.676292 0.005583 1.730159
 C 0.160441 -1.158682 1.641731
 C 1.517119 -0.717970 1.492023
 C 1.520216 0.717002 1.490229
 C 0.164493 1.163673 1.636925
 C -0.276704 -2.243414 0.917055
 C 0.623160 -3.045299 0.106916
 C 1.948911 -2.616036 -0.036777
 C 2.353074 -1.392352 0.630507
 C 3.335742 -0.704812 -0.188864
 C 3.338847 0.695452 -0.189450
 C 2.358300 1.386845 0.628414
 C 1.960305 2.613708 -0.038578
 C 0.637407 3.050039 0.106490
 C -0.267672 2.250744 0.912791
 C -1.605093 2.324733 0.348900
 C -2.424826 1.191574 0.437475
 C -1.893603 0.009245 1.090675
 C -1.614116 -2.309593 0.353182
 C -2.428512 -1.174359 0.440053
 C -0.259131 -3.828574 -0.767753
 C -1.552204 -3.403120 -0.625398
 C 2.929696 -2.795064 -1.116368
 C 3.732140 -1.690340 -1.203958
 C 3.739601 1.679651 -1.203998
 C 2.943134 2.788995 -1.116354
 C -0.240164 3.846191 -0.760583
 C -1.536501 3.431380 -0.618283
 C 3.532388 0.698145 -0.410891
 C -3.522122 -0.674024 -0.397298
 H 0.076273 -4.573350 -1.479425
 H -2.392468 -3.761260 -1.208016
 H 2.958374 -3.640159 -1.793665
 H 4.490749 -1.529860 -1.960516
 H 4.497508 1.515977 -1.960629
 H 2.976737 3.634131 -1.793334
 H 0.098455 4.597967 -1.463358
 H -2.374842 3.814437 -1.186181
 C -4.441926 1.564548 -1.224936
 H -4.940642 2.316885 -0.602663
 H -3.884011 2.108851 -1.997233
 H -5.211212 0.970369 -1.724903
 H -4.182996 -1.294752 -0.991457
```

Fragment 2 substituted with one *i*-Pr group

Molecular formula: C33H16
Charge = 0 Multiplicity = 1
Hartree/particle
Thermal correction to Energy= 0.396077
Thermal correction to Enthalpy= 0.397022
Thermal correction to Gibbs Free Energy= 0.328986
Sum of electronic and zero-point Energies= -1266.662030
Sum of electronic and thermal Energies= -1266.641484
Sum of electronic and thermal Enthalpies= -1266.640539
Sum of electronic and thermal Free Energies= -1266.708575

E (Thermal) KCal/Mol CV Cal/Mol-Kelvin S Cal/Mol-Kelvin
248.542 91.677 143.194

Cartesian coordinates

Atom	X	Y	Z
C	-0.649938	0.018231	1.729010
C	0.192130	-1.142906	1.653934
C	1.549002	-0.696920	1.522223
C	1.546549	0.738182	1.519423
C	0.187139	1.179486	1.646745
C	-0.231182	-2.230031	0.924744
C	0.682314	-3.029076	0.127268
C	2.008113	-2.594312	0.000411
C	2.398509	-1.368405	0.671979
C	3.389865	-0.677730	-0.134519
C	3.387883	0.722532	-0.135630
C	2.393657	1.410466	0.668597
C	1.999823	2.635311	-0.004058
C	0.673175	3.066175	0.122488
C	-0.240622	2.263231	0.915226
C	-1.570998	2.331167	0.332502
C	-2.389112	1.194335	0.411460
C	-1.857764	0.016518	1.072655
C	-1.561276	-2.302687	0.344764
C	-2.381207	-1.170070	0.420220
C	-0.185660	-3.817209	-0.757169
C	-1.482361	-3.397083	-0.631404
C	3.003913	-2.770235	-1.065637
C	3.803335	-1.662425	-1.143370
C	3.798464	1.708074	-1.144993
C	2.996545	2.814340	-1.068432
C	-0.194411	3.858915	-0.756910
C	-1.490917	3.438710	-0.633691
C	-3.494253	0.695690	-0.439230
C	-3.473191	-0.678431	-0.422939
H	0.161684	-4.561286	-1.463833
H	-2.313560	-3.759524	-1.224312
H	3.044778	-3.615568	-1.742021
H	4.571317	-1.499683	-1.889929
H	4.567140	1.547173	-1.891286
H	3.035921	3.659386	-1.745216
H	0.151064	4.611895	-1.455035
H	-2.320029	3.817240	-1.217382
C	-4.412602	1.568147	-1.267524
H	-4.359543	2.582619	-0.852324
C	-3.947535	1.634498	-2.738200
H	-4.016622	0.647308	-3.207274
H	-4.574720	2.326618	-3.310975
H	-2.908916	1.968192	-2.817945
C	-5.880000	1.113628	-1.179472
H	-6.527096	1.798385	-1.737675
H	-6.013671	0.113658	-1.605894
H	-6.226002	1.084741	-0.142171
H	-4.124936	-1.313186	-1.011644

Fragment 2 substituted with one F group

Molecular formula: C30H9F
Charge = 0 Multiplicity = 1
Hartree/particle
Thermal correction to Energy= 0.299916
Thermal correction to Enthalpy= 0.300860
Thermal correction to Gibbs Free Energy= 0.240400
Sum of electronic and zero-point Energies= -1248.031202
Sum of electronic and thermal Energies= -1248.014177
Sum of electronic and thermal Enthalpies= -1248.013233
Sum of electronic and thermal Free Energies= -1248.073693

E (Thermal) KCal/Mol CV Cal/Mol-Kelvin S Cal/Mol-Kelvin
188.200 78.367 127.250

Cartesian coordinates

Atom	X	Y	Z
C	-0.681966	0.000687	1.748224
C	0.157012	-1.161616	1.655593
C	1.512109	-0.717809	1.500213
C	1.512049	0.717171	1.498120
C	0.155208	1.161413	1.650599
C	-0.281575	-2.244986	0.930174
C	0.617475	-3.045929	0.117696
C	1.942299	-2.614822	-0.029632
C	2.346076	-1.390312	0.635801
C	3.323852	-0.700688	-0.187953
C	3.322364	0.699404	-0.189733
C	2.343351	1.388607	0.631608
C	1.939051	2.614712	-0.036765
C	0.617741	3.050330	0.114971
C	-0.279171	2.253890	0.933766
C	-1.614184	2.322140	0.372042
C	-2.429218	1.191355	0.480465
C	-1.905352	0.000185	1.119036
C	-1.620630	-2.314193	0.367989
C	-2.439104	-1.182655	0.456128
C	-0.264964	-3.830289	-0.754800
C	-1.558456	-3.406352	-0.610726
C	2.919654	-2.792249	-1.112457
C	3.719204	-1.685676	-1.203649
C	3.715606	1.683265	-1.207484
C	2.916373	2.790355	-1.118993
C	-0.268217	3.830050	-0.761953
C	-1.561556	3.408676	-0.613648
C	-3.498576	0.671351	-0.370785
C	-3.528082	-0.693518	-0.395379
H	0.070035	-4.574359	-1.467308
H	-2.399001	-3.765210	-1.192419
H	2.947795	-3.637510	-1.789508
H	4.474585	-1.523865	-1.963069
H	4.470830	1.520452	-1.966948
H	2.944772	3.634456	-1.797340
H	0.066539	4.568500	-1.480410
H	-2.408371	3.752809	-1.193652
F	-4.276151	1.476983	-1.104980
H	-4.179234	-1.290668	-1.018889

Fragment 2 substituted with one COOH group

```
Molecular formula: C31H10O2
Charge = 0 Multiplicity = 1
                                                  Hartree/particle
Thermal correction to Energy= 0.325077
Thermal correction to Enthalpy= 0.326021
Thermal correction to Gibbs Free Energy= 0.260775
Sum of electronic and zero-point Energies= -1337.357226
Sum of electronic and thermal Energies= -1337.338190
Sum of electronic and thermal Enthalpies= -1337.337245
Sum of electronic and thermal Free Energies= -1337.402491

E (Thermal) KCal/Mol CV Cal/Mol-Kelvin S Cal/Mol-Kelvin
 203.989 85.489 137.321


 Cartesian coordinates

Atom X Y Z
 C 1.264660 -0.128374 1.424697
 C 0.548347 1.112440 1.438792
 C -0.855581 0.816549 1.504927
 C -1.005521 -0.609015 1.527831
 C 0.306488 -1.192802 1.477134
 C 0.980484 2.156803 0.652278
 C 0.056198 3.050008 -0.024800
 C -1.310128 2.755462 0.028744
 C -1.734030 1.574104 0.765028
 C -2.890228 0.993928 0.109028
 C -3.036449 -0.399274 0.128905
 C -2.022800 -1.188002 0.806082
 C -1.854566 -2.451490 0.107764
 C -0.577174 -3.020359 0.062021
 C 0.514769 -2.314698 0.712333
 C 1.738044 -2.529924 -0.043996
 C 2.678407 -1.490393 -0.091789
 C 2.367780 -0.257841 0.609503
 C 2.214673 2.089191 -0.101578
 C 2.916907 0.872114 -0.116668
 C 0.876661 3.743740 -1.027903
 C 2.127101 3.189188 -1.070812
 C -2.417128 3.037237 -0.894682
 C -3.329132 2.018272 -0.849500
 C -3.681450 -1.337780 -0.799093
 C -2.998683 -2.523813 -0.810897
 C 0.079639 -3.908434 -0.905705
 C 1.418122 -3.630597 -0.965152
 C 3.697546 -1.090377 -1.085797
 C 3.817975 0.283787 -1.096746
 H 0.518463 4.522798 -1.689943
 H 2.906492 3.462048 -1.771829
 H -2.461116 3.882920 -1.570150
 H -4.203106 1.937555 -1.484616
 H -4.521575 -1.097489 -1.439419
 H -3.217708 -3.361036 -1.462502
 H -0.433837 -4.623801 -1.536801
 H 2.137759 -4.077630 -1.636181
 C 4.426382 -1.999464 -1.978593
 O 4.284706 -3.208670 -2.033299
 O 5.320383 -1.351455 -2.772579
 H 5.740133 -2.043188 -3.309976
 H 4.449546 0.830815 -1.784454
```

Fragment 2 substituted with one CF_3 group

Molecular formula: C31H9F3
Charge = 0 Multiplicity = 1
Hartree/particle
Thermal correction to Energy= 0.315591
Thermal correction to Enthalpy= 0.316535
Thermal correction to Gibbs Free Energy= 0.248700
Sum of electronic and zero-point Energies= -1485.825741
Sum of electronic and thermal Energies= -1485.805896
Sum of electronic and thermal Enthalpies= -1485.804952
Sum of electronic and thermal Free Energies= -1485.872787

E (Thermal) KCal/Mol CV Cal/Mol-Kelvin S Cal/Mol-Kelvin
198.036 88.012 142.770

Cartesian coordinates

Atom X Y Z
C -0.528563 0.064392 -1.977097
C -1.340576 -1.102267 -1.790905
C -2.664725 -0.670855 -1.441295
C -2.669492 0.763218 -1.411647
C -1.347219 1.216833 -1.741924
C -0.805457 -2.205794 -1.166673
C -1.578598 -3.028882 -0.253195
C -2.865866 -2.604708 0.094429
C -3.362758 -1.365255 -0.480679
C -4.215722 -0.699645 0.486801
C -4.219812 0.700584 0.516585
C -3.370949 1.411613 -0.422482
C -2.881759 2.628990 0.203160
C -1.596053 3.073562 -0.123939
C -0.816211 2.290818 -1.067537
C 0.586342 2.358047 -0.695308
C 1.386761 1.230992 -0.927966
C 0.770544 0.057955 -1.519195
C 0.596795 -2.281236 -0.804021
C 1.390975 -1.140460 -0.987306
C -0.577342 -3.831590 0.463479
C 0.681309 -3.400989 0.143168
C -3.680910 -2.810757 1.299120
C -4.460158 -1.709273 1.526328
C -4.470550 1.664257 1.596917
C -3.697946 2.779015 1.415433
C -0.601574 3.856129 0.621896
C 0.660629 3.445703 0.291315
C 2.595112 0.702943 -0.267365
C 2.592607 -0.666500 -0.300077
H -0.805180 -4.595563 1.196925
H 1.598071 -3.771992 0.584999
H -3.612296 -3.670897 1.953969
H -5.100449 -1.568072 2.388687
H -5.109036 1.482960 2.453066
H -3.633767 3.610737 2.106472
H -0.835075 4.591283 1.382454
H 1.573218 3.798604 0.750920
C 3.629265 1.524813 0.423194
F 4.706846 0.791129 0.771727
F 3.152364 2.093189 1.563190
F 4.058647 2.543701 -0.356595
H 3.340722 -1.292098 0.169048

Fragment 2 substituted with one CHO group

```
Molecular formula: C31H10O
Charge = 0 Multiplicity = 1
                                               Hartree/particle
Thermal correction to Energy= 0.318537
Thermal correction to Enthalpy= 0.319481
Thermal correction to Gibbs Free Energy= 0.256587
Sum of electronic and zero-point Energies= -1262.111620
Sum of electronic and thermal Energies= -1262.093506
Sum of electronic and thermal Enthalpies= -1262.092562
Sum of electronic and thermal Free Energies= -1262.155456

E (Thermal) KCal/Mol CV Cal/Mol-Kelvin S Cal/Mol-Kelvin
 199.885 81.999 132.372


 Cartesian coordinates

Atom X Y Z
 C -0.310293 0.010313 1.839270
 C 0.514147 -1.151919 1.691467
 C 1.865340 -0.716429 1.471923
 C 1.873406 0.716963 1.478098
 C 0.527196 1.166166 1.704026
 C 0.037034 -2.243072 0.999652
 C 0.888451 -3.047022 0.139495
 C 2.200033 -2.616821 -0.084791
 C 2.644981 -1.390504 0.560576
 C 3.577912 -0.705014 -0.312357
 C 3.584276 0.695960 -0.309787
 C 2.657629 1.386935 0.568590
 C 2.224678 2.619896 -0.068341
 C 0.915084 3.058148 0.154642
 C 0.056921 2.254578 1.010182
 C -1.309429 2.337014 0.521825
 C -2.129114 1.208604 0.655710
 C -1.564395 0.020425 1.267070
 C -1.325171 -2.308469 0.515078
 C -2.134472 -1.167200 0.655308
 C -0.046020 -3.830949 -0.680854
 C -1.328080 -3.405514 -0.461207
 C 3.117934 -2.797054 -1.217345
 C 3.912526 -1.691280 -1.349931
 C 3.928773 1.683625 -1.341115
 C 3.143114 2.795374 -1.201485
 C -0.013122 3.862983 -0.651253
 C -1.300218 3.450943 -0.436954
 C -3.280758 0.704376 -0.124191
 C -3.264929 -0.678226 -0.117951
 H 0.244874 -4.578684 -1.408710
 H -2.202967 -3.764698 -0.989257
 H 3.108511 -3.642154 -1.894831
 H 4.625825 -1.530879 -2.149382
 H 4.639487 1.520845 -2.142275
 H 3.139512 3.642934 -1.876088
 H 0.285420 4.616924 -1.369607
 H -2.185859 3.809055 -0.943430
 C -4.263362 1.472378 -0.882953
 H -5.044079 0.849114 -1.371232
 O -4.277206 2.687907 -1.004908
 H -3.961958 -1.296990 -0.672443
```

Fragment 2 substituted with one CN group

Molecular formula: C31H9N
Charge = 0 Multiplicity = 1

Hartree/particle

Thermal correction to Energy= 0.307535
Thermal correction to Enthalpy= 0.308479
Thermal correction to Gibbs Free Energy= 0.245711
Sum of electronic and zero-point Energies= -1241.039190
Sum of electronic and thermal Energies= -1241.021217
Sum of electronic and thermal Enthalpies= -1241.020273
Sum of electronic and thermal Free Energies= -1241.083040

E (Thermal) KCal/Mol CV Cal/Mol-Kelvin S Cal/Mol-Kelvin
192.981 81.383 132.106

Cartesian coordinates

Atom X Y Z
C 0.324900 -0.004275 1.866258
C -0.499992 1.156209 1.702975
C -1.843666 0.715952 1.450314
C -1.846040 -0.718267 1.451604
C -0.504208 -1.163273 1.707571
C -0.009299 2.247015 1.021242
C -0.843390 3.049871 0.143335
C -2.149099 2.616870 -0.109997
C -2.604283 1.388663 0.522823
C -3.515918 0.701606 -0.372934
C -3.517314 -0.698742 -0.373456
C -2.607435 -1.388683 0.523329
C -2.155128 -2.617296 -0.109083
C -0.849889 -3.052786 0.143585
C -0.015991 -2.252413 1.024100
C 1.359418 -2.324706 0.565716
C 2.165948 -1.191855 0.727059
C 1.593397 -0.005989 1.326907
C 1.364382 2.316232 0.564366
C 2.173340 1.178365 0.720299
C 0.106137 3.836731 -0.655958
C 1.384250 3.413184 -0.411161
C -3.042190 2.796481 -1.262240
C -3.830944 1.688997 -1.414902
C -3.834410 -1.684619 -1.415495
C -3.047353 -2.793419 -1.262672
C 0.099240 -3.841868 -0.654121
C 1.378823 -3.423026 -0.409918
C 3.320705 -0.687935 -0.044135
C 3.314734 0.692888 -0.045231
H -0.171120 4.584331 -1.389189
H 2.268889 3.774236 -0.921260
H -3.019839 3.642774 -1.937910
H -4.525850 1.528513 -2.230284
H -4.528196 -1.522289 -2.231413
H -3.025445 -3.638942 -1.939361
H -0.179245 -4.587651 -1.388682
H 2.265158 -3.779889 -0.918900
C 4.230860 -1.506658 -0.764674
N 4.958047 -2.204435 -1.349690
H 4.013053 1.306393 -0.600216

Fragment 2 substituted with one NO2 group

```
Molecular formula: C30H9NO2
Charge = 0 Multiplicity = 1
                                                Hartree/particle
Thermal correction to Energy= 0.312266
Thermal correction to Enthalpy= 0.313210
Thermal correction to Gibbs Free Energy= 0.248552
Sum of electronic and zero-point Energies= -1353.290164
Sum of electronic and thermal Energies= -1353.271446
Sum of electronic and thermal Enthalpies= -1353.270502
Sum of electronic and thermal Free Energies= -1353.335160

E (Thermal) KCal/Mol CV Cal/Mol-Kelvin S Cal/Mol-Kelvin
 195.950 83.884 136.085
```

```
 Cartesian coordinates

Atom X Y Z
 C 1.234291 -0.137662 1.471472
 C 0.517380 1.102528 1.478704
 C -0.888070 0.806411 1.505274
 C -1.037688 -0.619123 1.508076
 C 0.276306 -1.202057 1.485683
 C 0.970252 2.157377 0.718665
 C 0.064888 3.059078 0.027317
 C -1.301377 2.762588 0.038092
 C -1.744834 1.571604 0.748931
 C -2.881709 0.999079 0.054289
 C -3.027735 -0.394188 0.054653
 C -2.033296 -1.189743 0.751578
 C -1.845574 -2.446492 0.044753
 C -0.568207 -3.015703 0.030256
 C 0.505932 -2.317309 0.717489
 C 1.749396 -2.527615 -0.005775
 C 2.693754 -1.488894 -0.008689
 C 2.360710 -0.257761 0.687200
 C 2.224244 2.100420 -0.000863
 C 2.929541 0.883404 -0.009234
 C 0.913748 3.765697 -0.942843
 C 2.165726 3.212658 -0.957019
 C -2.383028 3.055138 -0.911372
 C -3.294865 2.035172 -0.903347
 C -3.646401 -1.323304 -0.900366
 C -2.963796 -2.509478 -0.905539
 C 0.114355 -3.893583 -0.927917
 C 1.454557 -3.618137 -0.946087
 C 3.732041 -1.052465 -0.955028
 C 3.858831 0.317089 -0.969538
 H 0.574707 4.553148 -1.604908
 H 2.964899 3.494481 -1.631458
 H -2.409527 3.908976 -1.577251
 H -4.151124 1.962057 -1.562916
 H -4.468369 -1.076478 -1.561331
 H -3.164718 -3.339847 -1.571406
 H -0.381072 -4.601067 -1.581719
 H 2.186891 -4.063203 -1.603638
 O 4.342323 -3.124658 -1.718818
 O 5.300995 -1.368544 -2.598522
 N 4.507299 -1.902862 -1.818639
 H 4.514190 0.853834 -1.641121
```

Fragment 1 substituted with two NH_2 group

Molecular formula: C30H14N2
Charge = 0 Multiplicity = 1
Hartree/particle
Thermal correction to Energy= 0.368809
Thermal correction to Enthalpy= 0.369753
Thermal correction to Gibbs Free Energy= 0.304012
Sum of electronic and zero-point Energies= -1260.766890
Sum of electronic and thermal Energies= -1260.747312
Sum of electronic and thermal Enthalpies= -1260.746368
Sum of electronic and thermal Free Energies= -1260.812109

E (Thermal) KCal/Mol CV Cal/Mol-Kelvin S Cal/Mol-Kelvin
231.431 88.349 138.364

Cartesian coordinates

Atom X Y Z
C -1.183429 1.241633 -1.317784
C 0.237414 1.183446 -1.460948
C 0.838906 -0.054381 -1.550560
C 0.077307 -1.259559 -1.479397
C -1.291848 -1.169272 -1.348039
C -1.947154 0.095761 -1.245221
C 0.839442 -2.286148 -0.806456
C 0.127776 -3.291593 -0.156927
C -1.315738 -3.200567 0.014143
C -1.985505 -2.084925 -0.537288
C -2.091321 -3.942850 0.949971
C -3.330450 -3.465755 1.363826
C -3.849599 -2.202886 0.963958
C -3.156796 -1.468686 0.015722
C -3.081538 -0.004489 -0.359272
C -3.485848 1.149319 0.305287
C -2.679495 2.361664 0.262560
C -1.493222 2.346587 -0.505119
C -0.300808 3.082229 -0.195833
C -0.431904 4.104576 0.729612
C -1.696641 4.312230 1.346878
C -2.778361 3.456751 1.167208
C 2.081965 -0.291145 -0.935673
C 2.802765 0.779671 -0.360477
C 2.152595 2.077730 -0.272567
C 0.852708 2.267042 -0.736214
C 3.980784 0.390111 0.352318
C 4.196452 -0.977777 0.631319
C 3.278164 -1.986424 0.245687
C 2.192004 -1.666484 -0.556712
H 0.648795 -4.047696 0.426602
H -1.690945 -4.849598 1.396274
H -3.890333 -4.041228 2.095727
H -4.738492 -1.816185 1.454712
H -4.285609 1.110645 1.041693
H 0.415298 4.708217 1.043625
H -1.791304 5.126848 2.059382
H -3.672683 3.590082 1.770473
H 2.611650 2.826208 0.369157
H 3.426786 -2.992076 0.633858
N 4.869128 1.327085 0.878056
H 5.795257 0.935456 1.018667
H 4.907532 2.195584 0.360519
N 5.367708 -1.291312 1.379459
H 5.346459 -0.880310 2.310849
H 5.501316 -2.291567 1.473948

Fragment 1 substituted with two OMe group

Molecular formula: C32H16O2
Charge = 0 Multiplicity = 1
Hartree/particle
Thermal correction to Energy= 0.402319
Thermal correction to Enthalpy= 0.403263
Thermal correction to Gibbs Free Energy= 0.330565
Sum of electronic and zero-point Energies= -1379.060592
Sum of electronic and thermal Energies= -1379.038253
Sum of electronic and thermal Enthalpies= -1379.037308
Sum of electronic and thermal Free Energies= -1379.110006

E (Thermal) KCal/Mol CV Cal/Mol-Kelvin S Cal/Mol-Kelvin
252.459 95.893 153.006

Cartesian coordinates

Atom X Y Z
C -1.238018 1.271928 -1.319845
C 0.172851 1.245685 -1.540095
C 0.793761 0.024270 -1.686847
C 0.063311 -1.198347 -1.591749
C -1.297954 -1.139595 -1.381443
C -1.973765 0.109011 -1.224629
C 0.881946 -2.215869 -0.975270
C 0.232939 -3.241392 -0.294747
C -1.201255 -3.187054 -0.044196
C -1.923802 -2.080349 -0.543749
C -1.905114 -3.957263 0.924665
C -3.129825 -3.513592 1.412077
C -3.697798 -2.258586 1.056917
C -3.075693 -1.497375 0.081216
C -3.054158 -0.027708 -0.278157
C -3.444673 1.107427 0.425486
C -2.668369 2.337945 0.355946
C -1.525911 2.359445 -0.475345
C -0.335204 3.117953 -0.220925
C -0.436700 4.124175 0.725355
C -1.670187 4.295811 1.412855
C -2.741224 3.418670 1.280094
C 2.073304 -0.192602 -1.142821
C 2.796232 0.881758 -0.576041
C 2.129918 2.170914 -0.438009
C 0.805989 2.332512 -0.829124
C 3.984197 0.506982 0.106633
C 4.276037 -0.859589 0.309526
C 3.375199 -1.893599 -0.065479
C 2.232687 -1.564537 -0.782339
H 0.803566 -3.989314 0.251727
H -1.459657 -4.859075 1.336754
H -3.635269 -4.109486 2.166764
H -4.566474 -1.897828 1.600725
H -4.200507 1.040692 1.204976
H 0.414169 4.740571 1.001799
H -1.743259 5.098397 2.141285
H -3.603943 3.524152 1.932814
H 2.630865 2.913949 0.174922
H 3.556216 -2.908567 0.268751
O 4.757212 1.477648 0.692782
O 5.444755 -1.098018 0.982159
C 6.015481 1.729513 0.058179
H 6.475703 2.550765 0.611180
H 6.663816 0.849554 0.102686
H 5.873300 2.030602 -0.987309
C 5.774743 -2.433270 1.327259
H 6.745264 -2.382022 1.822863
H 5.042600 -2.867430 2.020072
H 5.855788 -3.075520 0.440863

Fragment 1 substituted with two OH group

```
Molecular formula: C30H12O2
Charge = 0 Multiplicity = 1
                                                Hartree/particle
Thermal correction to Energy= 0.342936
Thermal correction to Enthalpy= 0.343880
Thermal correction to Gibbs Free Energy= 0.278371
Sum of electronic and zero-point Energies= -1300.517189
Sum of electronic and thermal Energies= -1300.497814
Sum of electronic and thermal Enthalpies= -1300.496870
Sum of electronic and thermal Free Energies= -1300.562379

E (Thermal) KCal/Mol CV Cal/Mol-Kelvin S Cal/Mol-Kelvin
 215.195 86.714 137.875
```

```
 Cartesian coordinates

Atom X Y Z
 C -1.232034 1.239949 -1.343764
 C 0.185289 1.198950 -1.515804
 C 0.802210 -0.030296 -1.608318
 C 0.059891 -1.244843 -1.503653
 C -1.306942 -1.171846 -1.339375
 C -1.978022 0.084787 -1.238941
 C 0.850677 -2.251397 -0.834911
 C 0.170974 -3.255665 -0.151298
 C -1.269473 -3.183440 0.052600
 C -1.967304 -2.085283 -0.498663
 C -2.010831 -3.922566 1.018006
 C -3.246554 -3.456899 1.454234
 C -3.793122 -2.207670 1.048084
 C -3.134258 -1.477604 0.072887
 C -3.090076 -0.018373 -0.325630
 C -3.495701 1.139388 0.331242
 C -2.709320 2.363026 0.251440
 C -1.540098 2.353509 -0.542001
 C -0.353357 3.113139 -0.272434
 C -0.479594 4.148448 0.638962
 C -1.733560 4.346604 1.280888
 C -2.805239 3.471405 1.140031
 C 2.063298 -0.240590 -1.019800
 C 2.773560 0.853497 -0.470906
 C 2.113272 2.147188 -0.387733
 C 0.801161 2.304372 -0.821353
 C 3.938125 0.484236 0.253921
 C 4.202147 -0.873510 0.499543
 C 3.320895 -1.916410 0.137984
 C 2.199535 -1.608588 -0.622575
 H 0.717197 -3.994945 0.430773
 H -1.586740 -4.816773 1.467585
 H -3.780644 -4.028955 2.207672
 H -4.675763 -1.826486 1.554146
 H -4.277742 1.100298 1.086394
 H 0.364999 4.770029 0.923051
 H -1.825383 5.171599 1.981637
 H -3.687976 3.601293 1.760795
 H 2.601117 2.911520 0.209833
 H 3.513237 -2.921253 0.509708
 O 4.745163 1.442383 0.779379
 H 5.415680 0.991317 1.316969
 O 5.359571 -1.091997 1.225176
 H 5.370848 -1.997303 1.559058
```

Fragment 1 substituted with two Me group

```
Molecular formula: C32H16
Charge = 0 Multiplicity = 1
                                                  Hartree/particle
Thermal correction to Energy= 0.390766
Thermal correction to Enthalpy= 0.391710
Thermal correction to Gibbs Free Energy= 0.324589
Sum of electronic and zero-point Energies= -1228.666860
Sum of electronic and thermal Energies= -1228.646756
Sum of electronic and thermal Enthalpies= -1228.645812
Sum of electronic and thermal Free Energies= -1228.712933

E (Thermal) KCal/Mol CV Cal/Mol-Kelvin S Cal/Mol-Kelvin
 245.209 89.211 141.268


 Cartesian coordinates

Atom X Y Z
 C -1.207936 1.233281 -1.336258
 C 0.213717 1.182912 -1.465094
 C 0.827676 -0.048839 -1.534497
 C 0.074645 -1.259327 -1.451109
 C -1.296673 -1.178360 -1.331332
 C -1.963046 0.082422 -1.252590
 C 0.837354 -2.269472 -0.758123
 C 0.132372 -3.267502 -0.092535
 C -1.313647 -3.187560 0.065681
 C -1.987487 -2.086544 -0.509141
 C -2.087741 -3.920087 1.009871
 C -3.333912 -3.447056 1.406951
 C -3.860488 -2.195934 0.981419
 C -3.167879 -1.471431 0.025519
 C -3.102912 -0.013212 -0.372926
 C -3.521012 1.147358 0.270879
 C -2.724328 2.365614 0.215343
 C -1.531955 2.348198 -0.542576
 C -0.347752 3.097475 -0.234987
 C -0.493963 4.132368 0.673832
 C -1.765389 4.340069 1.277380
 C -2.838971 3.473555 1.102290
 C 2.065719 -0.262041 -0.900871
 C 2.782224 0.812760 -0.329123
 C 2.114123 2.109865 -0.276471
 C 0.816645 2.279533 -0.749991
 C 3.954787 0.442763 0.408126
 C 4.200410 -0.919217 0.663494
 C 3.283314 -1.940658 0.277689
 C 2.182597 -1.629891 -0.499385
 H 0.657549 -4.007050 0.507996
 H -1.681884 -4.814911 1.474671
 H -3.893875 -4.014261 2.145072
 H -4.756183 -1.808689 1.459216
 H -4.325676 1.113466 1.002139
 H 0.346068 4.747225 0.985221
 H -1.872127 5.164956 1.976148
 H -3.739250 3.609347 1.696011
 H 2.563747 2.883731 0.339139
 H 3.449818 -2.948942 0.648827
 C 4.889671 1.492497 0.961206
 H 4.834265 1.556414 2.055784
 H 5.931544 1.262249 0.713469
 H 4.674592 2.483009 0.556750
 C 5.423156 -1.326388 1.457306
 H 6.351431 -1.059659 0.937077
 H 5.459815 -0.836382 2.437506
 H 5.440963 -2.406035 1.625404
```

Fragment 1 substituted with two *i*-Pr group

```
Molecular formula: C36H24
Charge = 0 Multiplicity = 1
                                                  Hartree/particle
Thermal correction to Energy= 0.510365
Thermal correction to Enthalpy= 0.511309
Thermal correction to Gibbs Free Energy= 0.432609
Sum of electronic and zero-point Energies= -1385.803049
Sum of electronic and thermal Energies= -1385.777484
Sum of electronic and thermal Enthalpies= -1385.776540
Sum of electronic and thermal Free Energies= -1385.855240

E (Thermal) KCal/Mol CV Cal/Mol-Kelvin S Cal/Mol-Kelvin
 320.259 110.006 165.638
```

```
 Cartesian coordinates

Atom X Y Z
 C -1.264163 1.272354 -1.262781
 C 0.160461 1.257668 -1.365555
 C 0.807388 0.045058 -1.449495
 C 0.081680 -1.184318 -1.412107
 C -1.293352 -1.139681 -1.318426
 C -1.992193 0.102122 -1.220894
 C 0.855090 -2.194507 -0.733152
 C 0.163329 -3.226583 -0.107212
 C -1.286931 -3.184916 0.025906
 C -1.976509 -2.085269 -0.532711
 C -2.060337 -3.960425 0.935669
 C -3.324580 -3.527570 1.321315
 C -3.873172 -2.278310 0.918866
 C -3.180934 -1.512498 -0.004572
 C -3.144177 -0.043167 -0.364295
 C -3.600271 1.090448 0.301625
 C -2.831700 2.327897 0.291667
 C -1.626886 2.358255 -0.445964
 C -0.464941 3.124426 -0.096904
 C -0.650355 4.130782 0.836494
 C -1.936407 4.293715 1.422777
 C -2.986744 3.408441 1.205994
 C 2.038672 -0.153582 -0.797522
 C 2.728891 0.913196 -0.181745
 C 2.017510 2.190412 -0.116259
 C 0.725912 2.344542 -0.611853
 C 3.928892 0.552468 0.539823
 C 4.210469 -0.819866 0.733061
 C 3.287211 -1.836336 0.321593
 C 2.179941 -1.529700 -0.438565
 H 0.695454 -3.969779 0.482559
 H -1.641711 -4.857661 1.384199
 H -3.884311 -4.127414 2.033338
 H -4.786674 -1.925303 1.389412
 H -4.416198 1.018788 1.017541
 H 0.169889 4.755390 1.179658
 H -2.073633 5.096738 2.141423
 H -3.899636 3.507626 1.787567
 H 2.428183 2.969205 0.513191
 H 3.469902 -2.855673 0.652168
 C 4.858114 1.637853 1.091727
 H 5.778784 1.139009 1.389403
 C 5.473719 -1.380422 1.416852
 H 5.323702 -2.466498 1.414552
 C 4.329357 2.314729 2.377589
 H 4.055762 1.569669 3.129669
 H 5.107322 2.957512 2.804946
 H 3.450840 2.940414 2.203888
 C 5.282359 2.672436 0.029021
 H 5.728209 2.176793 -0.838996
 H 4.447597 3.277435 -0.332675
 H 6.029648 3.354666 0.449155
 C 5.655947 -1.016587 2.904832
 H 5.958278 0.020949 3.064884
 H 4.729974 -1.185369 3.463565
 H 6.433053 -1.650994 3.345698
 C 6.762297 -1.161123 0.595987
 H 7.066780 -0.111902 0.548574
 H 7.590985 -1.722540 1.042035
 H 6.630582 -1.513821 -0.431432
```

Fragment 1 substituted with two F group

Molecular formula: C30H10F2
Charge = 0 Multiplicity = 1
Hartree/particle
Thermal correction to Energy= 0.317497
Thermal correction to Enthalpy= 0.318441
Thermal correction to Gibbs Free Energy= 0.254240
Sum of electronic and zero-point Energies= -1348.556578
Sum of electronic and thermal Energies= -1348.537999
Sum of electronic and thermal Enthalpies= -1348.537054
Sum of electronic and thermal Free Energies= -1348.601256

E (Thermal) KCal/Mol CV Cal/Mol-Kelvin S Cal/Mol-Kelvin
199.233 83.332 135.124

Cartesian coordinates

Atom X Y Z
C -1.200753 1.233071 -1.331807
C 0.218778 1.179400 -1.473406
C 0.826579 -0.055300 -1.555895
C 0.072694 -1.264871 -1.467831
C -1.296303 -1.178773 -1.329956
C -1.957511 0.083899 -1.241228
C 0.839185 -2.279980 -0.787068
C 0.139045 -3.276706 -0.114100
C -1.304482 -3.191983 0.060855
C -1.980706 -2.086663 -0.502624
C -2.069660 -3.924521 1.012368
C -3.309282 -3.447782 1.424583
C -3.836607 -2.193726 1.008128
C -3.153155 -1.468904 0.046054
C -3.088666 -0.010058 -0.350642
C -3.497506 1.151389 0.297600
C -2.700216 2.368830 0.232774
C -1.515626 2.349822 -0.536941
C -0.329579 3.100749 -0.243964
C -0.464843 4.136848 0.665042
C -1.729896 4.345567 1.281127
C -2.805071 3.478451 1.118786
C 2.071781 -0.275928 -0.941317
C 2.776962 0.813014 -0.376871
C 2.131148 2.114445 -0.306657
C 0.829218 2.279939 -0.765441
C 3.913211 0.418221 0.369763
C 4.162257 -0.926914 0.623118
C 3.289348 -1.970114 0.241098
C 2.189078 -1.646973 -0.538174
H 0.668683 -4.020073 0.477515
H -1.661836 -4.822016 1.469897
H -3.862671 -4.014542 2.167797
H -4.725560 -1.805209 1.497144
H -4.295217 1.119387 1.036383
H 0.378725 4.751660 0.966110
H -1.829313 5.171414 1.979561
H -3.699078 3.615719 1.721407
H 2.614281 2.875697 0.298408
H 3.487550 -2.964279 0.628329
F 4.711745 1.344261 0.930440
F 5.244688 -1.236978 1.364360

Fragment 1 substituted with two COOH group

Molecular formula: C32H12O4
Charge = 0 Multiplicity = 1

Hartree/particle

Thermal correction to Energy= 0.367529
Thermal correction to Enthalpy= 0.368473
Thermal correction to Gibbs Free Energy= 0.294526
Sum of electronic and zero-point Energies= -1527.193562
Sum of electronic and thermal Energies= -1527.170913
Sum of electronic and thermal Enthalpies= -1527.169968
Sum of electronic and thermal Free Energies= -1527.243916

E (Thermal) KCal/Mol CV Cal/Mol-Kelvin S Cal/Mol-Kelvin
230.628 97.723 155.635

Cartesian coordinates

Atom X Y Z
C -2.031219 1.218094 -1.316102
C -0.632090 1.164224 -1.588579
C -0.030449 -0.066876 -1.737661
C -0.772829 -1.277028 -1.578894
C -2.123452 -1.194193 -1.313142
C -2.774246 0.066792 -1.157094
C 0.053576 -2.292874 -0.977253
C -0.576112 -3.291928 -0.240535
C -1.996559 -3.211245 0.069088
C -2.723966 -2.105479 -0.425662
C -2.666170 -3.947371 1.087512
C -3.862136 -3.474127 1.615986
C -4.427899 -2.219950 1.254103
C -3.839766 -1.491267 0.233498
C -3.815282 -0.031539 -0.163113
C -4.159618 1.127031 0.526552
C -3.373449 2.345766 0.391014
C -2.268237 2.331309 -0.488835
C -1.059249 3.080169 -0.305503
C -1.106547 4.111169 0.617889
C -2.307299 4.317040 1.352384
C -3.393185 3.450480 1.289196
C 1.264491 -0.282355 -1.234071
C 2.034045 0.791444 -0.731595
C 1.381637 2.092727 -0.600729
C 0.044090 2.259224 -0.935966
C 3.264666 0.403257 -0.109624
C 3.549536 -0.965921 0.059832
C 2.599969 -1.988403 -0.222521
C 1.416282 -1.655605 -0.851562
H 0.010255 -4.034155 0.296237
H -2.214528 -4.844664 1.502052
H -4.341975 -4.043318 2.406841
H -5.267239 -1.833957 1.825809
H -4.881966 1.090569 1.339037
H -0.237179 4.723347 0.840373
H -2.339440 5.139266 2.061322
H -4.225585 3.584179 1.975042
H 1.918170 2.868308 -0.065747
H 2.838987 -2.995088 0.103194
C 4.133654 1.477623 0.460680
C 4.861420 -1.472801 0.570481
O 4.345290 2.550317 -0.066633
O 4.997054 -2.477770 1.237391
O 4.602990 1.173250 1.694387
H 5.164478 1.922390 1.956942
O 5.915852 -0.748025 0.127998
H 6.704877 -1.163367 0.515095

Fragment 1 substituted with two CF_3 group

```
Molecular formula: C32H10F6
Charge = 0 Multiplicity = 1
                                                   Hartree/particle
Thermal correction to Energy= 0.348680
Thermal correction to Enthalpy= 0.349625
Thermal correction to Gibbs Free Energy= 0.272412
Sum of electronic and zero-point Energies= -1824.133455
Sum of electronic and thermal Energies= -1824.109478
Sum of electronic and thermal Enthalpies= -1824.108534
Sum of electronic and thermal Free Energies= -1824.185746

E (Thermal) KCal/Mol CV Cal/Mol-Kelvin S Cal/Mol-Kelvin
 218.800 102.568 162.507
```

```
 Cartesian coordinates

Atom X Y Z
 C -2.418721 1.246415 -1.291169
 C -1.024788 1.194188 -1.590616
 C -0.429477 -0.034207 -1.775271
 C -1.172901 -1.245423 -1.630026
 C -2.518958 -1.164926 -1.341796
 C -3.163369 0.094290 -1.146911
 C -0.340573 -2.274521 -1.061511
 C -0.960861 -3.288182 -0.337355
 C -2.376630 -3.212723 -0.006501
 C -3.108169 -2.094428 -0.465536
 C -3.033034 -3.970692 1.004455
 C -4.219813 -3.506761 1.561047
 C -4.786960 -2.243017 1.236674
 C -4.211844 -1.492618 0.224425
 C -4.188237 -0.024275 -0.138363
 C -4.515444 1.117872 0.585985
 C -3.725051 2.335503 0.468719
 C -2.635177 2.338272 -0.430055
 C -1.418812 3.074225 -0.245823
 C -1.442774 4.079064 0.706812
 C -2.629665 4.272616 1.466521
 C -3.722369 3.415230 1.397071
 C 0.874469 -0.263788 -1.300409
 C 1.663746 0.792952 -0.797158
 C 1.011707 2.091211 -0.616907
 C -0.331876 2.267081 -0.919756
 C 2.926053 0.387041 -0.245350
 C 3.166323 -0.981302 -0.014419
 C 2.198089 -1.984755 -0.302606
 C 1.024516 -1.640208 -0.941563
 H -0.368714 -4.043179 0.174757
 H -2.578015 -4.878511 1.391389
 H -4.689779 -4.092815 2.345429
 H -5.616632 -1.868081 1.829388
 H -5.223949 1.063858 1.409542
 H -0.565349 4.678445 0.932366
 H -2.644095 5.074591 2.198721
 H -4.542367 3.535067 2.100141
 H 1.539666 2.847327 -0.048978
 H 2.387861 -2.995417 0.034018
 C 3.905456 1.477827 0.155398
 C 4.448696 -1.487289 0.633931
 F 3.750704 2.575871 -0.624543
 F 3.707217 1.878965 1.432798
 F 5.193484 1.117517 0.032232
 F 4.780401 -0.780311 1.734259
 F 5.496584 -1.458347 -0.213713
 F 4.320505 -2.774731 1.036922
```

Fragment 1 substituted with two CHO group

```
Molecular formula: C32H12O2
Charge = 0 Multiplicity = 1
                                                  Hartree/particle
Thermal correction to Energy= 0.354849
Thermal correction to Enthalpy= 0.355793
Thermal correction to Gibbs Free Energy= 0.285566
Sum of electronic and zero-point Energies= -1376.705199
Sum of electronic and thermal Energies= -1376.684159
Sum of electronic and thermal Enthalpies= -1376.683215
Sum of electronic and thermal Free Energies= -1376.753442

E (Thermal) KCal/Mol CV Cal/Mol-Kelvin S Cal/Mol-Kelvin
 222.671 90.953 147.805
```

Cartesian coordinates

Atom	X	Y	Z
C	1.612312	1.207289	1.312005
C	0.200012	1.199022	1.512175
C	-0.447541	-0.011644	1.639867
C	0.266004	-1.245343	1.531699
C	1.630436	-1.206892	1.337844
C	2.326730	0.032286	1.202935
C	-0.556963	-2.243406	0.898821
C	0.078455	-3.270262	0.206141
C	1.514664	-3.236400	-0.028796
C	2.247956	-2.146386	0.491799
C	2.213185	-4.004282	-1.003355
C	3.448440	-3.572551	-1.473859
C	4.031845	-2.331446	-1.095822
C	3.414205	-1.573414	-0.114506
C	3.413978	-0.109180	0.265587
C	3.828247	1.030357	-0.417568
C	3.073356	2.273227	-0.336335
C	1.924735	2.302519	0.485305
C	0.749108	3.082657	0.230218
C	0.873035	4.098292	-0.703158
C	2.115520	4.259984	-1.376793
C	3.171350	3.364337	-1.246157
C	-1.718814	-0.191181	1.069577
C	-2.433865	0.899258	0.522805
C	-1.733380	2.177050	0.411542
C	-0.408302	2.303240	0.813204
C	-3.665427	0.537630	-0.131913
C	-3.961817	-0.839905	-0.324032
C	-3.045127	-1.878897	0.007995
C	-1.889385	-1.567433	0.691947
H	-0.502558	-4.001284	-0.351344
H	1.756819	-4.892830	-1.431216
H	3.951363	-4.164776	-2.232737
H	4.911050	-1.977365	-1.626771
H	4.590103	0.962595	-1.190996
H	0.033671	4.730243	-0.978824
H	2.207556	5.070580	-2.093756
H	4.041424	3.464715	-1.889599
H	-2.219431	2.957788	-0.158647
H	-3.295305	-2.882286	-0.319437
C	-4.565033	1.582709	-0.675202
H	-5.417870	1.227786	-1.280491
C	-5.238951	-1.315451	-0.932740
H	-6.041521	-0.571493	-1.078897
O	-4.417139	2.783086	-0.510072
O	-5.436500	-2.477884	-1.239261

Fragment 1 substituted with two CN group

```
Molecular formula: C32H10N2
Charge = 0 Multiplicity = 1
                                                  Hartree/particle
Thermal correction to Energy= 0.332688
Thermal correction to Enthalpy= 0.333633
Thermal correction to Gibbs Free Energy= 0.264780
Sum of electronic and zero-point Energies= -1334.566040
Sum of electronic and thermal Energies= -1334.545526
Sum of electronic and thermal Enthalpies= -1334.544582
Sum of electronic and thermal Free Energies= -1334.613434

E (Thermal) KCal/Mol CV Cal/Mol-Kelvin S Cal/Mol-Kelvin
 208.765 89.480 144.912
```

Cartesian coordinates

Atom	X	Y	Z
C	-1.511957	1.243194	-1.318337
C	-0.106065	1.164292	-1.542291
C	0.476653	-0.079735	-1.667519
C	-0.291058	-1.277400	-1.532314
C	-1.647344	-1.168171	-1.310989
C	-2.279607	0.105437	-1.180533
C	0.495055	-2.306164	-0.900013
C	-0.177409	-3.291159	-0.181365
C	-1.605367	-3.183703	0.079065
C	-2.294202	-2.065377	-0.442063
C	-2.321864	-3.904386	1.076464
C	-3.525472	-3.406767	1.563093
C	-4.055007	-2.143281	1.179122
C	-3.419952	-1.428580	0.176848
C	-3.355858	0.028964	-0.223239
C	-3.703227	1.196163	0.450265
C	-2.892154	2.400667	0.337393
C	-1.758300	2.364221	-0.503997
C	-0.545034	3.094465	-0.282246
C	-0.604362	4.128430	0.637251
C	-1.825563	4.357032	1.329694
C	-2.923168	3.508703	1.231286
C	1.747088	-0.316916	-1.118314
C	2.505838	0.750197	-0.591955
C	1.888949	2.064919	-0.491672
C	0.564858	2.252977	-0.870155
C	3.706619	0.344993	0.073691
C	3.954844	-1.030305	0.293857
C	3.009528	-2.046173	-0.037358
C	1.862904	-1.692808	-0.723836
H	0.376110	-4.043338	0.375946
H	-1.901846	-4.808975	1.507736
H	-4.042375	-3.964073	2.338708
H	-4.905979	-1.740626	1.721180
H	-4.454083	1.175790	1.236903
H	0.267279	4.726058	0.887991
H	-1.867491	5.181406	2.035267
H	-3.776009	3.659020	1.887768
H	2.422038	2.822392	0.076095
H	3.199022	-3.056636	0.308501
C	4.603862	1.336213	0.578916
C	5.155685	-1.435916	0.962712
N	5.297020	2.184801	0.971863
N	6.118674	-1.804113	1.501726

Fragment 1 substituted with two NO_2 group

Molecular formula: C30H10N2O4
Charge = 0 Multiplicity = 1

Hartree/particle

Thermal correction to Energy= 0.341972
Thermal correction to Enthalpy= 0.342917
Thermal correction to Gibbs Free Energy= 0.269799
Sum of electronic and zero-point Energies= -1559.057042
Sum of electronic and thermal Energies= -1559.034948
Sum of electronic and thermal Enthalpies= -1559.034004
Sum of electronic and thermal Free Energies= -1559.107121

E (Thermal) KCal/Mol CV Cal/Mol-Kelvin S Cal/Mol-Kelvin
214.591 94.643 153.888

Cartesian coordinates

Atom	X	Y	Z
C	-1.992009	1.234272	-1.310717
C	-0.593433	1.178085	-1.576838
C	0.005257	-0.053452	-1.735313
C	-0.739874	-1.263969	-1.590020
C	-2.090409	-1.178386	-1.326227
C	-2.737885	0.083157	-1.162103
C	0.080306	-2.286843	-0.993921
C	-0.553955	-3.289896	-0.265396
C	-1.974683	-3.207962	0.039134
C	-2.696390	-2.095078	-0.448124
C	-2.649020	-3.950803	1.049673
C	-3.844700	-3.477466	1.578193
C	-4.405040	-2.218223	1.225115
C	-3.812162	-1.482816	0.212082
C	-3.782539	-0.020117	-0.173065
C	-4.125581	1.134431	0.524146
C	-3.336920	2.352758	0.399768
C	-2.229217	2.342890	-0.476471
C	-1.021355	3.089912	-0.284512
C	-1.068103	4.113633	0.646996
C	-2.270837	4.316057	1.378786
C	-3.357467	3.451168	1.305736
C	1.296717	-0.276203	-1.229231
C	2.063342	0.800663	-0.721169
C	1.419221	2.104260	-0.580636
C	0.082282	2.269496	-0.913294
C	3.274179	0.388384	-0.100535
C	3.540673	-0.971172	0.089141
C	2.616084	-1.998233	-0.208398
C	1.440800	-1.652312	-0.851159
H	0.028461	-4.038577	0.266495
H	-2.201888	-4.853139	1.457653
H	-4.328905	-4.051157	2.362872
H	-5.244959	-1.834776	1.797462
H	-4.851005	1.094037	1.333537
H	-0.198547	4.722532	0.876846
H	-2.303833	5.132741	2.093678
H	-4.191767	3.581418	1.989722
H	1.957080	2.876038	-0.041223
H	2.857353	-3.003454	0.115293
O	4.381342	2.406876	-0.178831
O	4.850776	-2.423649	1.319807
O	4.485762	1.246861	1.670668
O	5.833473	-0.766133	0.291445
N	4.133960	1.417759	0.508167
N	4.843684	-1.411291	0.622278

Fragment 2 substituted with two NH2 group

Molecular formula: C30H12N2
Charge = 0 Multiplicity = 1
Hartree/particle
Thermal correction to Energy= 0.343503
Thermal correction to Enthalpy= 0.344447
Thermal correction to Gibbs Free Energy= 0.278754
Sum of electronic and zero-point Energies= -1259.478192
Sum of electronic and thermal Energies= -1259.458633
Sum of electronic and thermal Enthalpies= -1259.457689
Sum of electronic and thermal Free Energies= -1259.523381

E (Thermal) KCal/Mol CV Cal/Mol-Kelvin S Cal/Mol-Kelvin
215.551 87.523 138.261

Cartesian coordinates

Atom X Y Z
C -0.725530 -0.000051 1.706445
C 0.116774 -1.163523 1.625039
C 1.472234 -0.717747 1.499597
C 1.472244 0.717703 1.499687
C 0.116790 1.163468 1.625178
C -0.306263 -2.245896 0.887861
C 0.609020 -3.041598 0.091141
C 1.939941 -2.610476 -0.022593
C 2.329354 -1.391346 0.655814
C 3.328395 -0.699946 -0.141544
C 3.328384 0.699974 -0.141512
C 2.329349 1.391345 0.655910
C 1.939935 2.610499 -0.022425
C 0.608987 3.041572 0.091274
C -0.306270 2.245770 0.887944
C -1.636270 2.314251 0.300741
C -2.454324 1.181984 0.393016
C -1.928705 -0.000023 1.053076
C -1.636246 -2.314329 0.300671
C -2.454344 -1.182086 0.393086
C -0.252403 -3.814184 -0.808468
C -1.551338 -3.393053 -0.689552
C 2.940209 -2.785310 -1.084281
C 3.745007 -1.680478 -1.152451
C 3.745006 1.680554 -1.152359
C 2.940194 2.785374 -1.084119
C -0.252484 3.814258 -0.808196
C -1.551427 3.393095 -0.689296
C -3.568938 0.695092 -0.422544
C -3.569393 -0.695234 -0.421930
H 0.099253 -4.544609 -1.527271
H -2.371753 -3.730380 -1.310439
H 2.980949 -3.627670 -1.764547
H 4.518295 -1.516906 -1.893358
H 4.518293 1.517022 -1.893275
H 2.980914 3.627762 -1.764352
H 0.099150 4.544798 -1.526893
H -2.372031 3.730379 -1.309915
N -4.359040 -1.544956 -1.198600
H -4.594593 -2.412334 -0.730835
H -5.189714 -1.123662 -1.595393
N -4.356712 1.545023 -1.200738
H -5.179492 1.121720 -1.611232
H -4.601883 2.408613 -0.730936

Fragment 2 substituted with two OMe group

Molecular formula: C32H14O2
Charge = 0 Multiplicity = 1

Hartree/particle

Thermal correction to Energy= 0.377448
Thermal correction to Enthalpy= 0.378392
Thermal correction to Gibbs Free Energy= 0.306973
Sum of electronic and zero-point Energies= -1377.769856
Sum of electronic and thermal Energies= -1377.747870
Sum of electronic and thermal Enthalpies= -1377.746926
Sum of electronic and thermal Free Energies= -1377.818345

E (Thermal) KCal/Mol CV Cal/Mol-Kelvin S Cal/Mol-Kelvin
236.852 94.165 150.315

Cartesian coordinates

Atom	X	Y	Z
C	-0.901397	0.002768	1.654578
C	-0.057827	-1.159153	1.598708
C	1.302769	-0.715079	1.509435
C	1.302870	0.719949	1.507807
C	-0.057661	1.164426	1.596096
C	-0.459964	-2.243662	0.853537
C	0.475805	-3.040164	0.080413
C	1.807184	-2.610301	-0.000228
C	2.179011	-1.388339	0.687669
C	3.198826	-0.699477	-0.084019
C	3.198917	0.700482	-0.085608
C	2.179204	1.391226	0.684527
C	1.807540	2.611682	-0.006123
C	0.476235	3.041934	0.073596
C	-0.459657	2.247323	0.848504
C	-1.771411	2.313100	0.226686
C	-2.600745	1.188883	0.306261
C	-2.096508	0.002103	0.978176
C	-1.771712	-2.310620	0.231836
C	-2.600821	-1.186086	0.308826
C	-0.363545	-3.812814	-0.842226
C	-1.663181	-3.389855	-0.757449
C	2.836233	-2.787486	-1.033426
C	3.642137	-1.682694	-1.081277
C	3.642340	1.681380	-1.085100
C	2.836595	2.786394	-1.039736
C	-0.362989	3.812659	-0.850753
C	-1.662695	3.390124	-0.765016
C	-3.686099	0.691011	-0.552314
C	-3.686037	-0.690026	-0.550891
H	0.006308	-4.543531	-1.551384
H	-2.469106	-3.726670	-1.397028
H	2.895445	-3.630951	-1.710742
H	4.434887	-1.520501	-1.801587
H	4.435056	1.517447	-1.805054
H	2.895909	3.628322	-1.718953
H	0.006987	4.541726	-1.561545
H	-2.468538	3.725727	-1.405334
O	-4.473136	1.444874	-1.362692
O	-4.472875	-1.445838	-1.359727
C	-5.215163	2.477608	-0.706553
H	-5.745206	3.018675	-1.492010
H	-5.940720	2.049358	-0.004575
H	-4.560050	3.168444	-0.164419
C	-5.215940	-2.476077	-0.700879
H	-5.939941	-2.045389	0.001218
H	-5.747822	-3.017646	-1.484747
H	-4.561351	-3.167101	-0.158283

Fragment 2 substituted with two OH group

Molecular formula: C30H10O2
Charge = 0 Multiplicity = 1

Hartree/particle

Thermal correction to Energy= 0.318192
Thermal correction to Enthalpy= 0.319137
Thermal correction to Gibbs Free Energy= 0.255233
Sum of electronic and zero-point Energies= -1299.224756
Sum of electronic and thermal Energies= -1299.205927
Sum of electronic and thermal Enthalpies= -1299.204983
Sum of electronic and thermal Free Energies= -1299.268886

E (Thermal) KCal/Mol CV Cal/Mol-Kelvin S Cal/Mol-Kelvin
199.669 84.824 134.496

Cartesian coordinates

Atom	X	Y	Z
C	-0.711664	-0.009373	1.737115
C	0.139281	-1.164877	1.654993
C	1.489056	-0.708917	1.494878
C	1.475375	0.726152	1.477146
C	0.118273	1.160687	1.627202
C	-0.286480	-2.265522	0.948110
C	0.617339	-3.064760	0.141164
C	1.937610	-2.620113	-0.012779
C	2.330306	-1.385047	0.639833
C	3.304501	-0.696144	-0.189243
C	3.293129	0.703619	-0.205612
C	2.307004	1.396134	0.606526
C	1.892860	2.605277	-0.076629
C	0.562333	3.027532	0.063427
C	-0.328517	2.232733	0.888977
C	-1.669783	2.284242	0.330240
C	-2.477410	1.147275	0.450128
C	-1.932561	-0.030629	1.113256
C	-1.625635	-2.357055	0.391046
C	-2.451970	-1.234170	0.480982
C	-0.260715	-3.865098	-0.722228
C	-1.559700	-3.456142	-0.578041
C	2.916466	-2.799870	-1.093249
C	3.707446	-1.687706	-1.194944
C	3.679483	1.676560	-1.235782
C	2.868466	2.776351	-1.161592
C	-0.325222	3.783132	-0.825911
C	-1.616876	3.351312	-0.673897
C	-3.572449	0.624471	-0.357227
C	-3.555442	-0.754262	-0.347722
H	0.080810	-4.609637	-1.431306
H	-2.403331	-3.817030	-1.152213
H	2.951515	-3.651462	-1.762067
H	4.461825	-1.527482	-1.955778
H	4.437321	1.510903	-1.991968
H	2.888446	3.611747	-1.851167
H	0.003829	4.509587	-1.559146
H	-2.453660	3.679356	-1.278567
O	-4.405738	1.324049	-1.193828
H	-4.657125	2.153551	-0.766321
O	-4.343448	-1.573407	-1.081873
H	-4.939437	-1.015735	-1.606034

Fragment 2 substituted with two Me group

Molecular formula: C32H14
Charge = 0 Multiplicity = 1
Hartree/particle
Thermal correction to Energy= 0.365958
Thermal correction to Enthalpy= 0.366903
Thermal correction to Gibbs Free Energy= 0.301307
Sum of electronic and zero-point Energies= -1227.382263
Sum of electronic and thermal Energies= -1227.362682
Sum of electronic and thermal Enthalpies= -1227.361738
Sum of electronic and thermal Free Energies= -1227.427333

E (Thermal) KCal/Mol CV Cal/Mol-Kelvin S Cal/Mol-Kelvin
229.642 87.352 138.058

Cartesian coordinates

Atom X Y Z
C -0.682252 -0.000154 1.720404
C 0.156810 -1.161382 1.632915
C 1.513062 -0.717564 1.489746
C 1.513011 0.717442 1.489840
C 0.156727 1.161146 1.633065
C -0.275979 -2.245749 0.905602
C 0.629846 -3.047369 0.102653
C 1.955200 -2.614611 -0.036856
C 2.353776 -1.389744 0.631134
C 3.338026 -0.700035 -0.184104
C 3.337977 0.700262 -0.184012
C 2.353677 1.389794 0.631316
C 1.955016 2.614721 -0.036513
C 0.629631 3.047365 0.103049
C -0.276138 2.245576 0.905892
C -1.612926 2.316514 0.338150
C -2.427924 1.179686 0.421314
C -1.894955 -0.000155 1.074137
C -1.612762 -2.316710 0.337853
C -2.427840 -1.179951 0.421162
C -0.246302 -3.841062 -0.767133
C -1.542370 -3.422725 -0.629468
C 2.940501 -2.791934 -1.112527
C 3.740010 -1.684767 -1.197883
C 3.739892 1.685155 -1.197660
C 2.940306 2.792255 -1.112160
C -0.246573 3.841108 -0.766636
C -1.542611 3.422658 -0.629028
C -3.539363 0.689731 -0.418894
C -3.539313 -0.689965 -0.418986
H 0.092791 -4.593186 -1.469326
H -2.378804 -3.802949 -1.202013
H 2.973850 -3.637202 -1.789418
H 4.500729 -1.522575 -1.951991
H 4.500624 1.523116 -1.951789
H 2.973597 3.637615 -1.788939
H 0.092465 4.593349 -1.468729
H -2.379071 3.802897 -1.201526
C -4.432348 -1.595743 -1.210373
H -3.880387 -2.077351 -2.027968
H -4.835795 -2.398094 -0.582157
H -5.274226 -1.060516 -1.654887
C -4.432464 1.595548 -1.210162
H -4.835956 2.397798 -0.581844
H -3.880541 2.077290 -2.027703
H -5.274314 1.060322 -1.654733

Fragment 2 substituted with two *i*-Pr group

```
Molecular formula: C36H22
Charge = 0 Multiplicity = 1
                                                  Hartree/particle
Thermal correction to Energy= 0.485546
Thermal correction to Enthalpy= 0.486490
Thermal correction to Gibbs Free Energy= 0.409757
Sum of electronic and zero-point Energies= -1384.521415
Sum of electronic and thermal Energies= -1384.496493
Sum of electronic and thermal Enthalpies= -1384.495549
Sum of electronic and thermal Free Energies= -1384.572282

E (Thermal) KCal/Mol CV Cal/Mol-Kelvin S Cal/Mol-Kelvin
 304.685 107.997 161.498
```

```
 Cartesian coordinates

Atom X Y Z
 C -0.720947 -0.037768 1.584779
 C 0.145186 -1.176893 1.572803
 C 1.499779 -0.706320 1.550745
 C 1.468464 0.728148 1.548183
 C 0.095515 1.142192 1.575074
 C -0.204808 -2.264408 0.810940
 C 0.786406 -3.059237 0.108838
 C 2.109350 -2.598463 0.078945
 C 2.423209 -1.360979 0.768919
 C 3.457943 -0.653011 0.036841
 C 3.426760 0.747361 0.031288
 C 2.362763 1.416429 0.759567
 C 1.994338 2.631572 0.057410
 C 0.652997 3.035904 0.086866
 C -0.299219 2.216832 0.814382
 C -1.591643 2.263733 0.149691
 C -2.395840 1.113066 0.180640
 C -1.873625 -0.064766 0.839008
 C -1.490064 -2.370533 0.133610
 C -2.342043 -1.249549 0.140817
 C 0.004818 -3.899645 -0.802531
 C -1.307827 -3.513093 -0.780254
 C 3.187755 -2.760122 -0.905835
 C 3.965838 -1.634272 -0.931951
 C 3.889882 1.740027 -0.948113
 C 3.061707 2.829402 -0.932871
 C -0.166072 3.810217 -0.851920
 C -1.461328 3.369364 -0.814487
 C -3.486166 0.623076 -0.685829
 C -3.452396 -0.761144 -0.726750
 H 0.413503 -4.669835 -1.445531
 H -2.081276 -3.955050 -1.391962
 H 3.297203 -3.607500 -1.571967
 H 4.783372 -1.459034 -1.620915
 H 4.714005 1.594014 -1.636005
 H 3.132484 3.674182 -1.607502
 H 0.213467 4.562867 -1.532500
 H -2.247498 3.728600 -1.466082
 C -4.455860 1.592294 -1.345093
 H -4.093300 2.591956 -1.083407
 C -4.381525 -1.640598 -1.541023
 H -5.255169 -1.031033 -1.790546
 C -4.492119 1.532068 -2.884447
 H -4.952447 0.610635 -3.250655
 H -5.078627 2.369320 -3.278825
 H -3.484849 1.595916 -3.307710
 C -5.872699 1.498052 -0.737743
 H -6.510800 2.291104 -1.143051
 H -6.354183 0.540928 -0.958577
 H -5.839734 1.611710 0.349750
 C -3.764601 -2.074798 -2.889564
 H -4.471963 -2.700752 -3.445100
 H -3.523621 -1.205266 -3.506563
 H -2.841957 -2.643096 -2.760047
 C -4.909543 -2.840877 -0.731993
 H -5.623437 -3.416989 -1.330330
 H -4.109427 -3.518139 -0.422016
 H -5.421887 -2.504254 0.174621
```

Fragment 2 substituted with two F group

Molecular formula: C30H8F2
Charge = 0 Multiplicity = 1
Hartree/particle
Thermal correction to Energy= 0.292878
Thermal correction to Enthalpy= 0.293822
Thermal correction to Gibbs Free Energy= 0.231059
Sum of electronic and zero-point Energies= -1347.262148
Sum of electronic and thermal Energies= -1347.244081
Sum of electronic and thermal Enthalpies= -1347.243137
Sum of electronic and thermal Free Energies= -1347.305900

E (Thermal) KCal/Mol CV Cal/Mol-Kelvin S Cal/Mol-Kelvin
183.783 81.361 132.096

Cartesian coordinates

Atom X Y Z
C -0.685281 -0.000002 1.744210
C 0.153541 -1.161873 1.650688
C 1.509339 -0.717440 1.498497
C 1.509330 0.717463 1.498495
C 0.153525 1.161879 1.650690
C -0.280828 -2.251254 0.930961
C 0.617047 -3.049293 0.114915
C 1.940149 -2.614840 -0.033310
C 2.343144 -1.389701 0.634398
C 3.322650 -0.699961 -0.186879
C 3.322640 0.700000 -0.186880
C 2.343120 1.389729 0.634386
C 1.940100 2.614854 -0.033338
C 0.617003 3.049311 0.114933
C -0.280863 2.251257 0.930971
C -1.616967 2.321563 0.367493
C -2.433575 1.191951 0.469877
C -1.909448 -0.000010 1.118655
C -1.616932 -2.321579 0.367491
C -2.433558 -1.191981 0.469880
C -0.266662 -3.828767 -0.762385
C -1.561017 -3.407304 -0.616797
C 2.918632 -2.790929 -1.114655
C 3.718141 -1.684037 -1.203219
C 3.718145 1.684088 -1.203200
C 2.918530 2.790912 -1.114738
C -0.266720 3.828841 -0.762298
C -1.561050 3.407264 -0.616823
C -3.500228 0.684579 -0.383885
C -3.500231 -0.684629 -0.383868
H 0.069114 -4.567267 -1.480288
H -2.405873 -3.752097 -1.199333
H 2.947601 -3.635421 -1.792507
H 4.474327 -1.521480 -1.961673
H 4.474360 1.521549 -1.961630
H 2.947460 3.635376 -1.792627
H 0.069047 4.567383 -1.480161
H -2.405895 3.752023 -1.199395
F -4.275569 -1.459503 -1.149869
F -4.275564 1.459435 -1.149905

Fragment 2 substituted with two COOH group

```
Molecular formula: C32H10O4
Charge = 0 Multiplicity = 1
                                                Hartree/particle
Thermal correction to Energy= 0.342768
Thermal correction to Enthalpy= 0.343712
Thermal correction to Gibbs Free Energy= 0.270970
Sum of electronic and zero-point Energies= -1525.907750
Sum of electronic and thermal Energies= -1525.885632
Sum of electronic and thermal Enthalpies= -1525.884687
Sum of electronic and thermal Free Energies= -1525.957429

E (Thermal) KCal/Mol CV Cal/Mol-Kelvin S Cal/Mol-Kelvin
 215.090 95.747 153.097
```

Cartesian coordinates

```
Atom X Y Z
 C 1.262188 -0.111436 1.426220
 C 0.537881 1.122709 1.444261
 C -0.865997 0.815909 1.502883
 C -1.004335 -0.610250 1.514025
 C 0.313479 -1.181859 1.464427
 C 0.969532 2.167109 0.662052
 C 0.041779 3.061946 -0.010385
 C -1.322298 2.759410 0.034647
 C -1.743116 1.569070 0.759092
 C -2.889517 0.984171 0.090001
 C -3.024425 -0.410732 0.100861
 C -2.010715 -1.194099 0.781043
 C -1.826751 -2.454221 0.076424
 C -0.546365 -3.015206 0.040666
 C 0.535103 -2.302800 0.700581
 C 1.763570 -2.506010 -0.042781
 C 2.699532 -1.455912 -0.073310
 C 2.371877 -0.227267 0.617421
 C 2.212899 2.118342 -0.085201
 C 2.930134 0.906140 -0.092511
 C 0.864518 3.774751 -0.995358
 C 2.120619 3.233572 -1.039700
 C -2.425212 3.038975 -0.894006
 C -3.329595 2.012305 -0.863346
 C -3.654192 -1.349835 -0.837860
 C -2.963360 -2.531048 -0.850730
 C 0.126667 -3.896418 -0.922833
 C 1.464387 -3.610122 -0.965783
 C 3.739818 -1.070525 -1.037335
 C 3.893589 0.309981 -1.029095
 H 0.503803 4.555438 -1.654098
 H 2.901340 3.496288 -1.738392
 H -2.470386 3.888227 -1.564851
 H -4.197519 1.929319 -1.506349
 H -4.490976 -1.112489 -1.483683
 H -3.171327 -3.366571 -1.507970
 H -0.373830 -4.617577 -1.557682
 H 2.192057 -4.065966 -1.622183
 C 4.881892 1.110860 -1.797479
 C 4.403704 -2.046304 -1.924558
 O 4.629348 2.125364 -2.416729
 O 4.473578 -3.240433 -1.698770
 O 6.137429 0.630425 -1.676464
 H 6.698902 1.210150 -2.219484
 O 4.895890 -1.500405 -3.061198
 H 5.325408 -2.228614 -3.540613
```

Fragment 2 substituted with two CF_3 group

```
Molecular formula: C32H8F6
Charge = 0 Multiplicity = 1
                                                  Hartree/particle
Thermal correction to Energy= 0.323897
Thermal correction to Enthalpy= 0.324841
Thermal correction to Gibbs Free Energy= 0.249359
Sum of electronic and zero-point Energies= -1822.849031
Sum of electronic and thermal Energies= -1822.825557
Sum of electronic and thermal Enthalpies= -1822.824613
Sum of electronic and thermal Free Energies= -1822.900095

E (Thermal) KCal/Mol CV Cal/Mol-Kelvin S Cal/Mol-Kelvin
 203.248 100.654 158.865
```

Cartesian coordinates

```
Atom X Y Z
 C -0.490278 0.044726 -1.923504
 C -1.310631 -1.115159 -1.752578
 C -2.645618 -0.677978 -1.449116
 C -2.644462 0.755288 -1.427301
 C -1.308300 1.200012 -1.717665
 C -0.797137 -2.213641 -1.105324
 C -1.601359 -3.033245 -0.215036
 C -2.896125 -2.603487 0.091272
 C -3.371948 -1.363816 -0.504675
 C -4.249367 -0.690368 0.434275
 C -4.248461 0.710327 0.455897
 C -3.370230 1.411180 -0.461863
 C -2.891553 2.629824 0.171835
 C -1.595083 3.063831 -0.119871
 C -0.788640 2.272999 -1.034410
 C 0.606866 2.344753 -0.635311
 C 1.421167 1.216849 -0.865013
 C 0.797351 0.038052 -1.434019
 C 0.593432 -2.294980 -0.703984
 C 1.398804 -1.156335 -0.884330
 C -0.624080 -3.841948 0.525381
 C 0.646119 -3.420971 0.239898
 C -3.747936 -2.800507 1.271360
 C -4.528448 -1.694313 1.470448
 C -4.525542 1.682405 1.522752
 C -3.742949 2.792677 1.357663
 C -0.621121 3.847816 0.646901
 C 0.649451 3.440038 0.347650
 C 2.643448 0.700520 -0.220922
 C 2.618153 -0.679622 -0.222149
 H -0.874315 -4.607103 1.250124
 H 1.547153 -3.798191 0.701957
 H -3.701969 -3.657147 1.932590
 H -5.192507 -1.546256 2.313412
 H -5.189648 1.509529 2.360934
 H -3.695209 3.628317 2.045140
 H -0.872118 4.586204 1.398694
 H 1.546081 3.805087 0.824741
 C 3.575714 -1.619160 0.449584
 C 3.760847 1.551671 0.317691
 F 3.707244 -2.758107 -0.272783
 F 3.112348 -1.988947 1.670641
 F 4.805249 -1.111739 0.626150
 F 4.915213 1.339688 -0.345738
 F 3.994851 1.325670 1.627876
 F 3.482876 2.870650 0.184876
```

Fragment 2 substituted with two CHO group

Molecular formula: C32H10O2
Charge = 0 Multiplicity = 1
Hartree/particle
Thermal correction to Energy= 0.329986
Thermal correction to Enthalpy= 0.330930
Thermal correction to Gibbs Free Energy= 0.263235
Sum of electronic and zero-point Energies= -1375.422947
Sum of electronic and thermal Energies= -1375.402657
Sum of electronic and thermal Enthalpies= -1375.401712
Sum of electronic and thermal Free Energies= -1375.469408

E (Thermal) KCal/Mol CV Cal/Mol-Kelvin S Cal/Mol-Kelvin
207.069 88.860 142.478

Cartesian coordinates

Atom X Y Z
C -0.326030 0.000002 1.801401
C 0.505783 -1.156428 1.670298
C 1.860506 -0.715903 1.470054
C 1.860518 0.715869 1.470060
C 0.505801 1.156418 1.670303
C 0.040339 -2.246435 0.974885
C 0.907686 -3.055818 0.133738
C 2.218997 -2.620831 -0.076455
C 2.650699 -1.387780 0.567794
C 3.588786 -0.700916 -0.297843
C 3.588800 0.700862 -0.297835
C 2.650725 1.387738 0.567806
C 2.219047 2.620801 -0.076433
C 0.907743 3.055809 0.133760
C 0.040375 2.246434 0.974894
C -1.318387 2.328437 0.477900
C -2.140581 1.190092 0.609383
C -1.572646 0.000015 1.210421
C -1.318421 -2.328409 0.477896
C -2.140586 -1.190049 0.609369
C -0.014436 -3.862018 -0.676552
C -1.303639 -3.449213 -0.472042
C 3.150875 -2.800252 -1.197320
C 3.940433 -1.690097 -1.326651
C 3.940469 1.690048 -1.326632
C 3.150931 2.800215 -1.197293
C -0.014360 3.862038 -0.676521
C -1.303571 3.449249 -0.472031
C -3.305201 0.702665 -0.138073
C -3.305165 -0.702614 -0.138129
H 0.287554 -4.621283 -1.387765
H -2.184306 -3.817234 -0.977995
H 3.153048 -3.647600 -1.871970
H 4.660937 -1.529291 -2.119472
H 4.660972 1.529235 -2.119453
H 3.153120 3.647568 -1.871936
H 0.287650 4.621308 -1.387721
H -2.184222 3.817295 -0.977986
C -4.298651 -1.546696 -0.809881
H -5.142031 -1.016069 -1.290998
C -4.298746 1.546735 -0.809764
H -5.142411 1.016123 -1.290396
O -4.247468 -2.766670 -0.855343
O -4.247391 2.766689 -0.855576

Fragment 2 substituted with two CN group

Molecular formula: C32H8N2
Charge = 0 Multiplicity = 1
Hartree/particle
Thermal correction to Energy= 0.307912
Thermal correction to Enthalpy= 0.308856
Thermal correction to Gibbs Free Energy= 0.241636
Sum of electronic and zero-point Energies= -1333.280452
Sum of electronic and thermal Energies= -1333.260538
Sum of electronic and thermal Enthalpies= -1333.259594
Sum of electronic and thermal Free Energies= -1333.326814

E (Thermal) KCal/Mol CV Cal/Mol-Kelvin S Cal/Mol-Kelvin
193.218 87.407 141.477

Cartesian coordinates

Atom X Y Z
C 0.327816 -0.000001 1.861134
C -0.498252 1.158450 1.699606
C -1.843758 0.716697 1.450251
C -1.843718 -0.716822 1.450238
C -0.498184 -1.158498 1.699593
C -0.011085 2.250846 1.019400
C -0.846855 3.055264 0.143187
C -2.150487 2.619119 -0.110332
C -2.602990 1.387720 0.521878
C -3.513611 0.700207 -0.374106
C -3.513570 -0.700387 -0.374114
C -2.602900 -1.387865 0.521840
C -2.150312 -2.619216 -0.110412
C -0.846676 -3.055316 0.143162
C -0.010954 -2.250862 1.019382
C 1.360513 -2.324753 0.559151
C 2.168259 -1.185575 0.718517
C 1.596417 0.000039 1.317158
C 1.360377 2.324826 0.559181
C 2.168189 1.185690 0.718512
C 0.101956 3.845911 -0.653015
C 1.382311 3.426071 -0.410640
C -3.045360 2.797904 -1.260778
C -3.831686 1.688631 -1.413983
C -3.831641 -1.688825 -1.413974
C -3.045075 -2.797954 -1.260958
C 0.102182 -3.845980 -0.652959
C 1.382500 -3.425952 -0.410726
C 3.314220 -0.697779 -0.053757
C 3.314110 0.697948 -0.053866
H -0.175977 4.594723 -1.384583
H 2.268348 3.784572 -0.918562
H -3.025194 3.644559 -1.935871
H -4.526599 1.527888 -2.229208
H -4.526609 -1.528116 -2.229158
H -3.024804 -3.644547 -1.936126
H -0.175710 -4.594838 -1.384495
H 2.268545 -3.784366 -0.918694
C 4.215032 1.525544 -0.772005
C 4.215232 -1.525337 -0.771828
N 4.927435 2.243980 -1.349526
N 4.927711 -2.243743 -1.349293

Fragment 2 substituted with two NO_2 group

Molecular formula: C30H8N2O4
Charge = 0 Multiplicity = 1
Hartree/particle
Thermal correction to Energy= 0.317190
Thermal correction to Enthalpy= 0.318134
Thermal correction to Gibbs Free Energy= 0.246460
Sum of electronic and zero-point Energies= -1557.767231
Sum of electronic and thermal Energies= -1557.745639
Sum of electronic and thermal Enthalpies= -1557.744694
Sum of electronic and thermal Free Energies= -1557.816368

E (Thermal) KCal/Mol CV Cal/Mol-Kelvin S Cal/Mol-Kelvin
199.039 92.723 150.850

Cartesian coordinates

Atom	X	Y	Z
C	1.256887	-0.103013	1.438463
C	0.528788	1.128755	1.458689
C	-0.874412	0.817552	1.503434
C	-1.009102	-0.608681	1.509660
C	0.312182	-1.175810	1.462165
C	0.963564	2.177237	0.685429
C	0.040611	3.072516	0.006942
C	-1.322553	2.763468	0.035807
C	-1.745985	1.569793	0.753349
C	-2.884929	0.983126	0.072326
C	-3.016907	-0.411815	0.080863
C	-2.006847	-1.192661	0.767459
C	-1.816534	-2.454674	0.062680
C	-0.537280	-3.015497	0.038574
C	0.539152	-2.301142	0.704678
C	1.767858	-2.501425	-0.033840
C	2.700621	-1.443578	-0.048701
C	2.376286	-0.208481	0.641177
C	2.215531	2.139874	-0.047788
C	2.940176	0.933909	-0.051228
C	0.873567	3.796617	-0.960851
C	2.135022	3.265175	-0.987586
C	-2.417925	3.041655	-0.901985
C	-3.319733	2.012429	-0.881840
C	-3.639036	-1.353515	-0.861156
C	-2.948024	-2.534576	-0.869334
C	0.141783	-3.889471	-0.926741
C	1.478468	-3.596736	-0.967947
C	3.732851	-1.046107	-0.989464
C	3.879753	0.327146	-0.995423
H	0.520786	4.585671	-1.613683
H	2.934958	3.561559	-1.650697
H	-2.459882	3.892407	-1.570946
H	-4.181797	1.929016	-1.532389
H	-4.472134	-1.117936	-1.512244
H	-3.151701	-3.370752	-1.526698
H	-0.353869	-4.603432	-1.573034
H	2.193911	-4.030529	-1.651900
O	4.924254	2.248510	-1.654761
O	3.873035	-2.571809	-2.691286
O	5.325717	0.407011	-2.767476
O	5.651872	-2.201029	-1.473118
N	4.771023	1.041416	-1.868173
N	4.489626	-2.002235	-1.794013

Transition state structure for the reaction of butadiene with Fragment 1

Molecular formula: C34H18
Charge = 0 Multiplicity = 1

Hartree/particle
Thermal correction to Energy= 0.424186
Thermal correction to Enthalpy= 0.425130
Thermal correction to Gibbs Free Energy= 0.354540
Sum of electronic and zero-point Energies= -1305.947309
Sum of electronic and thermal Energies= -1305.926010
Sum of electronic and thermal Enthalpies= -1305.925066
Sum of electronic and thermal Free Energies= -1305.995656

E (Thermal) KCal/Mol CV Cal/Mol-Kelvin S Cal/Mol-Kelvin
266.180 95.050 148.570

Cartesian coordinates

Atom X Y Z
C -1.484501 -0.455318 -1.124694
C -1.328754 0.964926 -1.080600
C -0.055532 1.486766 -1.026965
C 1.100112 0.641538 -0.985957
C 0.919047 -0.731496 -1.036977
C -0.394321 -1.298410 -1.075821
C 2.109780 1.231285 -0.159777
C 3.016781 0.362538 0.464880
C 2.841029 -1.063059 0.415716
C 1.741989 -1.584087 -0.287965
C 3.583183 -2.036661 1.227269
C 2.892095 -3.286604 1.536364
C 1.567913 -3.571799 1.056991
C 0.988808 -2.768499 0.106796
C -0.432366 -2.532664 -0.331263
C -1.659161 -2.938622 0.191213
C -2.811465 -2.052700 0.168748
C -2.664483 -0.784148 -0.436885
C -3.352308 0.408768 -0.033046
C -4.452009 0.235663 0.790183
C -4.784285 -1.079495 1.221811
C -3.983418 -2.186975 0.968019
C 0.204953 2.619235 -0.236615
C -0.853611 3.332048 0.369144
C -2.197187 2.778625 0.274755
C -2.426765 1.560611 -0.357919
C -0.435147 4.337971 1.286242
C 0.906903 4.448861 1.633371
C 1.903006 3.538734 1.181922
C 1.554331 2.575038 0.249785
H 3.761055 0.752568 1.156785
H -5.028626 1.076876 1.164901
H -5.659630 -1.205181 1.852843
H -4.220722 -3.138421 1.437012
H -2.966200 3.230814 0.897283
H -1.168499 4.977766 1.770287
H 1.196523 5.210138 2.352222
H 2.894876 3.578875 1.623941
H -1.726175 -3.826972 0.815465
H 1.030477 -4.403874 1.505173
H 4.160192 -1.623166 2.050072
H 3.184850 -3.785729 2.451068
C 4.080384 -4.994678 0.428870
H 3.606507 -5.930962 0.708262
H 3.666626 -4.518834 -0.450344
C 5.188531 -2.403499 0.069060
H 4.575583 -2.652051 -0.791715
H 5.646130 -1.420580 -0.013010
C 5.922793 -3.430448 0.686727
H 6.834246 -3.180427 1.224982
C 5.357005 -4.694864 0.877680
H 5.852967 -5.388756 1.553839

Transition state structure for the reaction of butadiene with Fragment 1 substituted with one NH_2 group

Molecular formula: C34H19N
Charge = 0 Multiplicity = 1

Hartree/particle

Thermal correction to Energy= 0.442493
Thermal correction to Enthalpy= 0.443437
Thermal correction to Gibbs Free Energy= 0.372330
Sum of electronic and zero-point Energies= -1361.292056
Sum of electronic and thermal Energies= -1361.270065
Sum of electronic and thermal Enthalpies= -1361.269120
Sum of electronic and thermal Free Energies= -1361.340227

E (Thermal) KCal/Mol CV Cal/Mol-Kelvin S Cal/Mol-Kelvin
277.669 99.638 149.657

Cartesian coordinates

Atom	X	Y	Z
C	-1.756577	-0.513229	-0.934914
C	-1.634541	0.911415	-0.934984
C	-0.374290	1.465485	-0.925561
C	0.803692	0.649146	-0.884166
C	0.653302	-0.729620	-0.888692
C	-0.646038	-1.327648	-0.884709
C	1.812730	1.284645	-0.098117
C	2.752083	0.448908	0.535248
C	2.611022	-0.973162	0.524966
C	1.510372	-1.541326	-0.132590
C	3.438409	-1.910731	1.352091
C	2.712738	-3.182993	1.715618
C	1.413220	-3.529920	1.237966
C	0.787921	-2.732819	0.305175
C	-0.638514	-2.542453	-0.104608
C	-1.849134	-2.964041	0.449598
C	-3.020232	-2.105537	0.426313
C	-2.915321	-0.850214	-0.216092
C	-3.623670	0.336968	0.167705
C	-4.704182	0.161489	1.015589
C	-4.997309	-1.149176	1.488095
C	-4.175923	-2.244672	1.249194
C	-0.125363	2.625195	-0.172274
C	-1.187995	3.328679	0.436974
C	-2.519429	2.741652	0.386047
C	-2.732265	1.500826	-0.207281
C	-0.775425	4.367883	1.319416
C	0.570158	4.518016	1.636717
C	1.578188	3.619265	1.188980
C	1.234250	2.623399	0.288933
H	3.501862	0.877257	1.198133
H	-5.293817	0.999015	1.378032
H	-5.858419	-1.277934	2.137883
H	-4.383601	-3.188418	1.746989
H	-3.286398	3.193230	1.011553
H	-1.514008	5.003104	1.801529
H	0.856011	5.304111	2.329969
H	2.577122	3.693453	1.610265
H	-1.885180	-3.837802	1.096648
H	0.911833	-4.376575	1.701916
C	3.777347	-4.707002	-0.124972
H	3.284826	-5.658388	-0.299250
H	3.411767	-3.878842	-0.717104
C	4.913234	-2.187953	0.586283
H	4.648527	-2.163942	-0.473052
H	5.519769	-1.306944	0.804019
C	5.567546	-3.446672	0.949165
H	6.460140	-3.439410	1.568333
C	4.981335	-4.648730	0.564964
H	5.415967	-5.578528	0.931727
N	3.219895	-3.777140	2.847533
H	2.886123	-4.720617	3.012452
H	4.242714	-3.741418	2.874754
H	3.743313	-1.437454	2.290187

Transition state structure for the reaction of butadiene with Fragment 1 substituted with one OMe group

Molecular formula: C35H20O
Charge = 0 Multiplicity = 1

Hartree/particle

Thermal correction to Energy= 0.459333
Thermal correction to Enthalpy= 0.460277
Thermal correction to Gibbs Free Energy= 0.385924
Sum of electronic and zero-point Energies= -1420.439673
Sum of electronic and thermal Energies= -1420.416224
Sum of electronic and thermal Enthalpies= -1420.415280
Sum of electronic and thermal Free Energies= -1420.489634

E (Thermal) KCal/Mol CV Cal/Mol-Kelvin S Cal/Mol-Kelvin
288.236 103.774 156.490

Cartesian coordinates

Atom X Y Z
C -1.752516 -0.529316 -0.959794
C -1.649255 0.896636 -0.940729
C -0.396155 1.467102 -0.949609
C 0.792650 0.665791 -0.946779
C 0.660315 -0.714365 -0.974134
C -0.630162 -1.329404 -0.948396
C 1.811015 1.303160 -0.172829
C 2.775722 0.471101 0.426177
C 2.652680 -0.951898 0.395481
C 1.545083 -1.526560 -0.249059
C 3.511550 -1.900366 1.169216
C 2.786521 -3.145930 1.583304
C 1.485192 -3.503873 1.147179
C 0.849255 -2.725686 0.195294
C -0.587393 -2.553462 -0.182833
C -1.780097 -2.999702 0.392045
C -2.962676 -2.157585 0.407678
C -2.889614 -0.892109 -0.219361
C -3.605039 0.279551 0.197671
C -4.663719 0.077381 1.066778
C -4.928438 -1.243877 1.526804
C -4.097844 -2.324287 1.254055
C -0.145650 2.618340 -0.183838
C -1.203502 3.298788 0.458898
C -2.528028 2.694913 0.427809
C -2.737873 1.460619 -0.179907
C -0.785079 4.330154 1.347751
C 0.565166 4.493156 1.637820
C 1.574590 3.614577 1.154688
C 1.223797 2.627511 0.247491
H 3.536352 0.898174 1.077411
H -5.256201 0.901501 1.454438
H -5.773059 -1.393665 2.193557
H -4.281574 -3.277806 1.742656
H -3.286770 3.126486 1.077053
H -1.520951 4.948477 1.855313
H 0.856128 5.272664 2.336394
H 2.581526 3.695705 1.555118
H -1.790045 -3.883252 1.026865
H 0.979772 -4.338222 1.621177
C 3.840559 -4.866565 -0.103764
H 3.396683 -5.856424 -0.141417
H 3.399062 -4.135397 -0.767219
C 4.903880 -2.225482 0.249032
H 4.504830 -2.324234 -0.762207
H 5.487126 -1.305024 0.304391
C 5.630686 -3.407962 0.680671
H 6.527601 -3.297176 1.282880
C 5.075670 -4.667545 0.500962
H 5.571471 -5.521601 0.960528
O 3.373612 -3.660936 2.689190
C 2.921430 -4.910407 3.193897
H 2.945108 -5.670517 2.404040
H 3.610751 -5.180516 3.994514
H 1.907232 -4.833943 3.601881
H 3.927492 -1.447049 2.071945

Transition state structure for the reaction of butadiene with Fragment 1 substituted with one OH group

Molecular formula: C34H18O
Charge = 0 Multiplicity = 1

Hartree/particle

Thermal correction to Energy= 0.428917
Thermal correction to Enthalpy= 0.429861
Thermal correction to Gibbs Free Energy= 0.358955
Sum of electronic and zero-point Energies= -1381.165906
Sum of electronic and thermal Energies= -1381.144092
Sum of electronic and thermal Enthalpies= -1381.143148
Sum of electronic and thermal Free Energies= -1381.214054

E (Thermal) KCal/Mol CV Cal/Mol-Kelvin S Cal/Mol-Kelvin
269.150 98.500 149.235

Cartesian coordinates

Atom	X	Y	Z
C	-1.780624	-0.554002	-0.923812
C	-1.658726	0.870351	-0.922337
C	-0.398195	1.424397	-0.924073
C	0.778854	0.607389	-0.893019
C	0.629035	-0.770295	-0.898330
C	-0.670196	-1.368573	-0.886404
C	1.795817	1.242927	-0.113961
C	2.739310	0.408826	0.510911
C	2.597945	-1.015611	0.502023
C	1.491594	-1.583834	-0.148425
C	3.399110	-1.945008	1.355453
C	2.701814	-3.234176	1.657151
C	1.417176	-3.603301	1.187989
C	0.773318	-2.777284	0.287339
C	-0.655541	-2.585186	-0.109952
C	-1.858950	-3.007374	0.458536
C	-3.030592	-2.148823	0.448471
C	-2.932130	-0.892634	-0.193074
C	-3.636253	0.293612	0.199793
C	-4.707267	0.116568	1.059710
C	-4.995005	-1.194311	1.534132
C	-4.175972	-2.289418	1.284915
C	-0.141941	2.583240	-0.171284
C	-1.198948	3.285539	0.449026
C	-2.530618	2.698084	0.410589
C	-2.749190	1.458314	-0.182635
C	-0.778209	4.324261	1.328104
C	0.570272	4.475540	1.632315
C	1.574276	3.577671	1.174266
C	1.221681	2.582643	0.276754
H	3.491883	0.836968	1.170523
H	-5.293081	0.953525	1.429507
H	-5.848460	-1.323766	2.193660
H	-4.377061	-3.233617	1.784311
H	-3.291368	3.148574	1.044343
H	-1.512436	4.958677	1.817782
H	0.862227	5.261389	2.323169
H	2.577285	3.652015	1.585586
H	-1.886203	-3.881603	1.105084
H	0.959852	-4.483255	1.629473
C	3.904320	-4.716036	-0.287675
H	3.445485	-5.659408	-0.562721
H	3.538310	-3.841862	-0.810434
C	4.949556	-2.217185	0.696140
H	4.764781	-2.141470	-0.376543
H	5.543751	-1.357934	1.009109
C	5.548775	-3.500174	1.058533
H	6.396232	-3.532227	1.737473
C	5.019838	-4.684644	0.523586
H	5.430826	-5.631058	0.872785
O	3.243220	-3.924460	2.668078
H	4.229923	-3.899983	2.525682
H	3.655044	-1.487850	2.315949

Transition state structure for the reaction of butadiene with Fragment 1 substituted with one Me group

```
Molecular formula: C35H20
Charge = 0 Multiplicity = 1
                                                  Hartree/particle
Thermal correction to Energy= 0.453321
Thermal correction to Enthalpy= 0.454265
Thermal correction to Gibbs Free Energy= 0.381126
Sum of electronic and zero-point Energies= -1345.237117
Sum of electronic and thermal Energies= -1345.214338
Sum of electronic and thermal Enthalpies= -1345.213393
Sum of electronic and thermal Free Energies= -1345.286533

E (Thermal) KCal/Mol CV Cal/Mol-Kelvin S Cal/Mol-Kelvin
 284.463 100.895 153.935
```

Cartesian coordinates

Atom	X	Y	Z
C	-1.462914	-0.483206	-1.131567
C	-1.318463	0.938705	-1.099887
C	-0.049730	1.471817	-1.049494
C	1.112953	0.636062	-0.998606
C	0.941949	-0.738783	-1.038680
C	-0.365896	-1.316674	-1.073754
C	2.116510	1.241427	-0.177373
C	3.029255	0.382878	0.457869
C	2.862532	-1.040368	0.421325
C	1.768729	-1.577649	-0.276856
C	3.623804	-2.010151	1.238824
C	2.892800	-3.234193	1.630956
C	1.599480	-3.538926	1.113112
C	1.024020	-2.758486	0.131852
C	-0.393390	-2.543393	-0.313725
C	-1.620176	-2.955375	0.208152
C	-2.778751	-2.079452	0.174646
C	-2.641417	-0.815068	-0.442803
C	-3.339521	0.375719	-0.050887
C	-4.439490	0.201527	0.771718
C	-4.762259	-1.112574	1.214142
C	-3.952246	-2.215821	0.971931
C	0.200434	2.612725	-0.268110
C	-0.864581	3.321935	0.330363
C	-2.203484	2.756991	0.238374
C	-2.422440	1.531917	-0.384315
C	-0.455794	4.338486	1.240231
C	0.884759	4.462520	1.588729
C	1.888805	3.556838	1.146136
C	1.549520	2.582568	0.221226
H	3.771148	0.785395	1.145403
H	-5.023370	1.041491	1.137875
H	-5.638074	-1.239688	1.844261
H	-4.183336	-3.164986	1.448621
H	-2.977125	3.208173	0.855909
H	-1.195052	4.976305	1.717863
H	1.167216	5.231782	2.301959
H	2.879580	3.608560	1.589459
H	-1.681948	-3.838461	0.840406
H	1.054996	-4.361690	1.571315
C	4.030850	-5.031250	0.156581
H	3.561712	-6.002873	0.275877
H	3.568591	-4.376085	-0.568029
C	5.123478	-2.391957	0.195997
H	4.597703	-2.555082	-0.742320
H	5.645900	-1.437001	0.190993
C	5.853982	-3.508155	0.691148
H	6.764859	-3.342736	1.261002
C	5.289549	-4.789148	0.668933
H	5.796426	-5.586707	1.209344
C	3.328320	-3.852084	2.934066
H	3.058204	-3.197598	3.774734
H	2.867537	-4.828909	3.103876
H	4.417579	-3.970998	2.962714
H	4.144652	-1.561661	2.085569

Transition state structure for the reaction of butadiene with Fragment 1 substituted with one *i*-Pr group

Molecular formula: C37H24
Charge = 0 Multiplicity = 1

Hartree/particle

Thermal correction to Energy= 0.512928
Thermal correction to Enthalpy= 0.513872
Thermal correction to Gibbs Free Energy= 0.434915
Sum of electronic and zero-point Energies= -1423.809393
Sum of electronic and thermal Energies= -1423.783877
Sum of electronic and thermal Enthalpies= -1423.782933
Sum of electronic and thermal Free Energies= -1423.861890

E (Thermal) KCal/Mol CV Cal/Mol-Kelvin S Cal/Mol-Kelvin
321.867 111.265 166.180

Cartesian coordinates

Atom X Y Z
C -1.625121 -0.337956 -0.785264
C -1.458579 1.081828 -0.761619
C -0.182158 1.597210 -0.799323
C 0.969477 0.744936 -0.830627
C 0.776198 -0.627412 -0.862181
C -0.538801 -1.186821 -0.806073
C 2.034732 1.332818 -0.077991
C 2.975717 0.458151 0.493574
C 2.785383 -0.960472 0.464817
C 1.641533 -1.480027 -0.160890
C 3.598814 -1.952524 1.213332
C 2.859444 -3.157527 1.685942
C 1.535637 -3.437793 1.246992
C 0.911548 -2.649949 0.298019
C -0.529595 -2.414702 -0.046831
C -1.723010 -2.810604 0.559473
C -2.868329 -1.918473 0.608737
C -2.756849 -0.654812 -0.015640
C -3.409030 0.544695 0.425335
C -4.451394 0.384293 1.322449
C -4.760847 -0.926090 1.785091
C -3.985109 -2.039972 1.486077
C 0.137612 2.732289 -0.034950
C -0.873072 3.455365 0.636505
C -2.223032 2.909681 0.636883
C -2.501440 1.688752 0.029948
C -0.387727 4.463551 1.517738
C 0.975210 4.566454 1.774207
C 1.932768 3.646795 1.262883
C 1.516337 2.680377 0.361384
H 3.768715 0.848153 1.129471
H -4.996467 1.231644 1.729139
H -5.592466 -1.042219 2.474448
H -4.195713 -2.986807 1.976717
H -2.945596 3.370437 1.306995
H -1.082863 5.111138 2.045824
H 1.317237 5.329600 2.467680
H 2.952090 3.682047 1.637877
H -1.752873 -3.694641 1.192804
H 0.994747 -4.244514 1.732808
C 3.920847 -4.992893 0.075662
H 3.491756 -5.984173 0.177847
H 3.381445 -4.316736 -0.571694
C 4.958048 -2.330744 0.085441
H 4.387619 -2.501744 -0.826190
H 5.485268 -1.379456 0.026557
C 5.749132 -3.444953 0.511027
H 6.702010 -3.270736 1.003948
C 5.206594 -4.732490 0.508383
H 5.770907 -5.534755 0.980847
C 2.888992 -5.169650 3.316899
H 3.070528 -5.864629 2.494601
H 3.406288 -5.546881 4.204837
H 1.817467 -5.181691 3.544588
C 3.384461 -3.755796 2.985350
H 4.479859 -3.795389 2.881499
C 3.074805 -2.821261 4.182064
H 1.994300 -2.750807 4.347312
H 3.531085 -3.214228 5.097154
H 3.453668 -1.806874 4.030515
H 4.176277 -1.504098 2.021792

Transition state structure for the reaction of butadiene with Fragment 1 substituted with one F group

Molecular formula: C34H17F
Charge = 0 Multiplicity = 1
Hartree/particle
Thermal correction to Energy= 0.416451
Thermal correction to Enthalpy= 0.417396
Thermal correction to Gibbs Free Energy= 0.344646
Sum of electronic and zero-point Energies= -1405.185798
Sum of electronic and thermal Energies= -1405.163522
Sum of electronic and thermal Enthalpies= -1405.162578
Sum of electronic and thermal Free Energies= -1405.235328

E (Thermal) KCal/Mol CV Cal/Mol-Kelvin S Cal/Mol-Kelvin
261.327 98.237 153.115

Cartesian coordinates

Atom	X	Y	Z
C	-1.761349	-0.509530	-0.989239
C	-1.638755	0.914192	-0.967772
C	-0.377232	1.465891	-0.940824
C	0.797439	0.647712	-0.904845
C	0.648973	-0.729047	-0.936348
C	-0.651365	-1.326327	-0.947060
C	1.804552	1.272593	-0.099549
C	2.738622	0.435588	0.524790
C	2.602766	-0.996077	0.490655
C	1.505218	-1.554277	-0.191817
C	3.377959	-1.927813	1.314539
C	2.733571	-3.207231	1.567054
C	1.401676	-3.538032	1.165992
C	0.781599	-2.746954	0.230851
C	-0.649571	-2.550369	-0.184992
C	-1.857907	-2.977517	0.363196
C	-3.030633	-2.118858	0.346742
C	-2.922800	-0.856011	-0.278748
C	-3.632431	0.326073	0.118579
C	-4.714694	0.139233	0.961668
C	-5.008998	-1.176913	1.417149
C	-4.186465	-2.269057	1.166399
C	-0.131360	2.615121	-0.169867
C	-1.197315	3.312063	0.441375
C	-2.528832	2.726371	0.375139
C	-2.739415	1.494468	-0.236812
C	-0.789019	4.341208	1.336975
C	0.555009	4.488815	1.662169
C	1.565289	3.595148	1.209671
C	1.225202	2.609878	0.297133
H	3.480001	0.852642	1.203560
H	-5.305080	0.972016	1.333677
H	-5.871000	-1.313698	2.063961
H	-4.393806	-3.218788	1.652628
H	-3.298770	3.169585	1.002896
H	-1.529964	4.971182	1.822264
H	0.837210	5.267656	2.364894
H	2.562433	3.665122	1.635697
H	-1.894235	-3.858610	1.000083
H	0.928812	-4.385044	1.653285
C	3.776513	-4.682587	0.046285
H	3.215577	-5.611855	0.059971
H	3.462430	-3.970370	-0.707256
C	5.110220	-2.199133	0.333612
H	4.622325	-2.219437	-0.635353
H	5.632040	-1.263345	0.519719
C	5.689200	-3.390499	0.804470
H	6.533813	-3.341389	1.487274
C	5.018615	-4.610090	0.650758
H	5.390205	-5.477410	1.191325
F	3.183691	-3.823742	2.692706
H	3.857511	-1.519992	2.200272

Transition state structure for the reaction of butadiene with Fragment 1 substituted with one COOH group

```
Molecular formula: C35H18O2
Charge = 0 Multiplicity = 1
                                                  Hartree/particle
Thermal correction to Energy= 0.442060
Thermal correction to Enthalpy= 0.443004
Thermal correction to Gibbs Free Energy= 0.366776
Sum of electronic and zero-point Energies= -1494.511390
Sum of electronic and thermal Energies= -1494.487305
Sum of electronic and thermal Enthalpies= -1494.486360
Sum of electronic and thermal Free Energies= -1494.562589

E (Thermal) KCal/Mol CV Cal/Mol-Kelvin S Cal/Mol-Kelvin
 277.397 105.209 160.436
```

Cartesian coordinates

```
Atom X Y Z
 C -1.694340 -0.352471 -0.940508
 C -1.546981 1.068104 -0.871373
 C -0.277390 1.599866 -0.831421
 C 0.884797 0.762591 -0.824882
 C 0.711794 -0.611202 -0.900092
 C -0.597690 -1.188256 -0.925725
 C 1.904186 1.344212 -0.006938
 C 2.831382 0.468463 0.583446
 C 2.659030 -0.951793 0.510157
 C 1.552947 -1.468784 -0.180104
 C 3.451573 -1.950368 1.271265
 C 2.695160 -3.154566 1.683441
 C 1.390397 -3.455735 1.176884
 C 0.813932 -2.663113 0.214168
 C -0.613417 -2.434811 -0.201540
 C -1.828193 -2.859806 0.335896
 C -2.986506 -1.982792 0.347973
 C -2.859318 -0.702427 -0.237642
 C -3.548195 0.477698 0.199929
 C -4.631863 0.281472 1.039067
 C -4.947319 -1.043693 1.452615
 C -4.143212 -2.140370 1.165377
 C -0.011046 2.718831 -0.024021
 C -1.063729 3.411583 0.613898
 C -2.404511 2.849737 0.532275
 C -2.636593 1.642005 -0.119081
 C -0.636589 4.402013 1.543922
 C 0.710302 4.515436 1.870804
 C 1.704658 3.621488 1.384672
 C 1.346734 2.674580 0.438762
 H 3.590128 0.849767 1.264250
 H -5.207570 1.111460 1.439274
 H -5.810195 -1.186859 2.096824
 H -4.364544 -3.101584 1.621780
 H -3.165798 3.284285 1.176443
 H -1.365918 5.026644 2.053111
 H 1.006794 5.263923 2.600133
 H 2.703314 3.659304 1.811280
 H -1.876139 -3.759153 0.94567
 H 0.861432 -4.273349 1.656364
 C 4.016176 -5.080943 0.516614
 H 3.647231 -6.038812 0.867743
 H 3.430572 -4.603654 -0.256184
 C 4.800304 -2.351923 0.082705
 H 4.177603 -2.683808 -0.746493
 H 5.234280 -1.375179 -0.121661
 C 5.689908 -3.321213 0.625331
 H 6.626472 -2.987042 1.064130
 C 5.273808 -4.634096 0.864428
 H 5.904639 -5.272050 1.479395
 C 3.060321 -3.851789 2.935447
 O 2.424218 -4.742479 3.473747
 O 4.207603 -3.368313 3.497565
 H 4.331217 -3.893864 4.304975
 H 4.039323 -1.531373 2.083393
```

Transition state structure for the reaction of butadiene with Fragment 1 substituted with one CF_3 group

Molecular formula: C35H17F3
Charge = 0 Multiplicity = 1
Hartree/particle
Thermal correction to Energy= 0.432470
Thermal correction to Enthalpy= 0.433414
Thermal correction to Gibbs Free Energy= 0.354921
Sum of electronic and zero-point Energies= -1642.978103
Sum of electronic and thermal Energies= -1642.953235
Sum of electronic and thermal Enthalpies= -1642.952290
Sum of electronic and thermal Free Energies= -1643.030784

E (Thermal) KCal/Mol CV Cal/Mol-Kelvin S Cal/Mol-Kelvin
271.379 107.723 165.203

Cartesian coordinates

Atom X Y Z
C -1.855695 -0.151851 -0.832289
C -1.684616 1.265951 -0.763118
C -0.405802 1.776019 -0.737519
C 0.741978 0.919937 -0.747666
C 0.546269 -0.450226 -0.822103
C -0.773057 -1.005908 -0.833192
C 1.780555 1.482923 0.061446
C 2.698601 0.594537 0.640381
C 2.505213 -0.827543 0.566041
C 1.383304 -1.323039 -0.116147
C 3.266758 -1.825685 1.325025
C 2.568759 -3.068932 1.658017
C 1.218273 -3.326798 1.206069
C 0.630983 -2.508093 0.281783
C -0.801627 -2.254329 -0.114208
C -2.014588 -2.659654 0.438888
C -3.158842 -1.763530 0.468141
C -3.017447 -0.484341 -0.115790
C -3.681211 0.706162 0.332758
C -4.757000 0.525750 1.185395
C -5.088866 -0.794861 1.600383
C -4.306778 -1.904076 1.300372
C -0.111109 2.889414 0.067939
C -1.144525 3.598655 0.719341
C -2.495244 3.058900 0.654015
C -2.754974 1.856119 0.003910
C -0.689587 4.581369 1.644218
C 0.662981 4.673098 1.954075
C 1.636507 3.763452 1.455174
C 1.251091 2.823564 0.513336
H 3.466875 0.962775 1.317413
H -5.313844 1.364122 1.594667
H -5.945483 -0.925205 2.255541
H -4.538200 -2.862539 1.757582
H -3.241149 3.504862 1.308252
H -1.402164 5.217383 2.162923
H 0.980612 5.416255 2.679886
H 2.640965 3.784698 1.869026
H -2.070418 -3.559571 1.047307
H 0.682008 -4.149980 1.668302
C 3.662793 -4.762637 0.427807
H 3.198689 -5.692535 0.738080
H 3.177445 -4.259669 -0.397964
C 4.774597 -2.188506 0.011655
H 4.074689 -2.409519 -0.787680
H 5.230506 -1.206587 -0.085290
C 5.546738 -3.236490 0.533298
H 6.506535 -3.010891 0.991972
C 4.983722 -4.498226 0.750943
H 5.527929 -5.209862 1.366738
C 2.876205 -3.634561 3.023613
F 2.201316 -2.953449 3.989362
F 2.506584 -4.930944 3.157147 F
4.185242 -3.548563 3.349291
H 3.908898 -1.441579 2.111464

Transition state structure for the reaction of butadiene with Fragment 1 substituted with one CHO group

Molecular formula: C35H18O
Charge = 0 Multiplicity = 1

Hartree/particle

Thermal correction to Energy= 0.435629
Thermal correction to Enthalpy= 0.436574
Thermal correction to Gibbs Free Energy= 0.362254
Sum of electronic and zero-point Energies= -1419.265331
Sum of electronic and thermal Energies= -1419.242131
Sum of electronic and thermal Enthalpies= -1419.241187
Sum of electronic and thermal Free Energies= -1419.315506

E (Thermal) KCal/Mol CV Cal/Mol-Kelvin S Cal/Mol-Kelvin
273.362 101.622 156.418

Cartesian coordinates

Atom	X	Y	Z
C	-1.669247	0.128279	-0.978713
C	-1.550645	1.549281	-0.869526
C	-0.293394	2.101583	-0.766625
C	0.882466	1.284387	-0.734420
C	0.737666	-0.089826	-0.849985
C	-0.559383	-0.688840	-0.942143
C	1.858114	1.862553	0.137907
C	2.779089	0.989621	0.741946
C	2.633373	-0.430717	0.627785
C	1.565441	-0.949963	-0.118938
C	3.411774	-1.444801	1.382651
C	2.649340	-2.649271	1.770285
C	1.380906	-2.970979	1.188652
C	0.833155	-2.167441	0.218925
C	-0.580894	-1.953319	-0.250714
C	-1.806289	-2.411551	0.231590
C	-2.980207	-1.555433	0.219898
C	-2.853669	-0.259191	-0.329591
C	-3.579998	0.897621	0.109174
C	-4.690435	0.662086	0.902004
C	-4.997027	-0.677759	1.272263
C	-4.162964	-1.753051	0.989725
C	-0.079019	3.204365	0.077718
C	-1.168130	3.862597	0.690437
C	-2.494167	3.280028	0.542636
C	-2.678714	2.085193	-0.146601
C	-0.795639	4.836452	1.660611
C	0.535083	4.964777	2.043311
C	1.563563	4.100333	1.575239
C	1.260009	3.171943	0.592743
H	3.504344	1.367472	1.459990
H	-5.295581	1.472083	1.299672
H	-5.880237	-0.851209	1.880363
H	-4.383103	-2.728627	1.415014
H	-3.287685	3.685131	1.166723
H	-1.555553	5.435464	2.155590
H	0.789183	5.699452	2.802010
H	2.543811	4.144317	2.041998
H	-1.860168	-3.325979	0.817872
H	0.841555	-3.798358	1.640498
C	4.179107	-4.569496	0.884881
H	3.881757	-5.504299	1.347711
H	3.567686	-4.236280	0.058151
C	4.753452	-1.844107	0.165854
H	4.120528	-2.287214	-0.601531
H	5.108135	-0.861918	-0.139026
C	5.722068	-2.691074	0.771379
H	6.644656	-2.249183	1.140042
C	5.401709	-3.991881	1.167832
H	6.081226	-4.511957	1.840067
C	2.981925	-3.317548	3.046740
O	2.272670	-4.128768	3.622706
H	3.948220	-2.991951	3.490205
H	4.003177	-1.037052	2.201754

Transition state structure for the reaction of butadiene with Fragment 1 substituted with one CN group

Molecular formula: C35H17N
Charge = 0 Multiplicity = 1

Hartree/particle

Thermal correction to Energy= 0.424467
Thermal correction to Enthalpy= 0.425411
Thermal correction to Gibbs Free Energy= 0.350895
Sum of electronic and zero-point Energies= -1398.190637
Sum of electronic and thermal Energies= -1398.167457
Sum of electronic and thermal Enthalpies= -1398.166513
Sum of electronic and thermal Free Energies= -1398.241029

E (Thermal) KCal/Mol CV Cal/Mol-Kelvin S Cal/Mol-Kelvin
266.357 101.224 156.832

Cartesian coordinates

Atom X Y Z
C -1.728426 -0.431132 -0.976118
C -1.597400 0.991380 -0.917989
C -0.333764 1.537456 -0.883392
C 0.837151 0.713111 -0.871014
C 0.680021 -0.662709 -0.936161
C -0.622703 -1.254429 -0.957498
C 1.850898 1.313138 -0.058412
C 2.789523 0.453388 0.535527
C 2.635286 -0.969697 0.470346
C 1.532473 -1.506615 -0.212130
C 3.447127 -1.949712 1.232010
C 2.708722 -3.177993 1.629701
C 1.397356 -3.494093 1.146377
C 0.805041 -2.705432 0.188188
C -0.624304 -2.496080 -0.225090
C -1.834207 -2.931177 0.316224
C -3.001968 -2.067061 0.323615
C -2.889021 -0.789220 -0.270265
C -3.590786 0.386146 0.159232
C -4.671599 0.183431 1.000460
C -4.972186 -1.142420 1.423045
C -4.156535 -2.232214 1.142651
C -0.078994 2.665520 -0.084793
C -1.138906 3.351005 0.548919
C -2.473306 2.773387 0.473099
C -2.692724 1.558438 -0.169207
C -0.722112 4.353420 1.470764
C 0.623724 4.485080 1.795124
C 1.627771 3.598917 1.314880
C 1.279498 2.640913 0.376598
H 3.545312 0.848434 1.211647
H -5.256444 1.009476 1.395397
H -5.833066 -1.290639 2.068683
H -4.367522 -3.192764 1.605245
H -3.238733 3.204290 1.114723
H -1.457968 4.973588 1.975885
H 0.912323 5.242439 2.518300
H 2.626321 3.651621 1.739985
H -1.873391 -3.827737 0.930832
H 0.872448 -4.320792 1.615840
C 4.014755 -5.051307 0.312146
H 3.631062 -6.032126 0.573013
H 3.461688 -4.511969 -0.444266
C 4.841359 -2.308859 0.092022
H 4.258984 -2.589991 -0.783439
H 5.292804 -1.327204 -0.038734
C 5.693845 -3.318387 0.622676
H 6.609830 -3.023272 1.128134
C 5.253284 -4.639156 0.756632
H 5.845020 -5.322643 1.360483
C 3.136330 -3.801224 2.843006
N 3.492263 -4.259943 3.854592
H 4.000726 -1.518823 2.064242

Transition state structure for the reaction of butadiene with Fragment 1 substituted with one NO_2 group

```
Molecular formula: C34H17NO2
Charge = 0 Multiplicity = 1
                                                    Hartree/particle
Thermal correction to Energy= 0.429267
Thermal correction to Enthalpy= 0.430211
Thermal correction to Gibbs Free Energy= 0.354935
Sum of electronic and zero-point Energies= -1510.453371
Sum of electronic and thermal Energies= -1510.429763
Sum of electronic and thermal Enthalpies= -1510.428819
Sum of electronic and thermal Free Energies= -1510.504094

E (Thermal) KCal/Mol CV Cal/Mol-Kelvin S Cal/Mol-Kelvin
 269.369 103.405 158.431
```

Cartesian coordinates

```
Atom X Y Z
 C -1.518358 -0.451393 -1.108588
 C -1.356548 0.968818 -1.086598
 C -0.082243 1.489689 -1.051377
 C 1.070162 0.641300 -0.997687
 C 0.883314 -0.732437 -1.023381
 C -0.431409 -1.297058 -1.050338
 C 2.085989 1.243637 -0.190259
 C 2.995153 0.380108 0.444575
 C 2.809823 -1.039539 0.422162
 C 1.703451 -1.571838 -0.257978
 C 3.578118 -2.003177 1.263817
 C 2.786763 -3.200102 1.634828
 C 1.512487 -3.544602 1.125749
 C 0.943814 -2.747682 0.155361
 C -0.470515 -2.519414 -0.286311
 C -1.698436 -2.914082 0.247133
 C -2.845941 -2.024289 0.214820
 C -2.696794 -0.765173 -0.410784
 C -3.377462 0.436222 -0.021996
 C -4.473099 0.280126 0.810214
 C -4.808728 -1.026970 1.263352
 C -4.014534 -2.141790 1.022143
 C 0.186183 2.634704 -0.281672
 C -0.867076 3.359275 0.318732
 C -2.212265 2.807383 0.241504
 C -2.449394 1.579533 -0.369214
 C -0.441219 4.378420 1.217913
 C 0.902847 4.491572 1.556328
 C 1.894149 3.571894 1.114240
 C 1.537846 2.595072 0.198543
 H 3.748059 0.779507 1.121424
 H -5.043985 1.129242 1.175521
 H -5.680584 -1.138972 1.901503
 H -4.252864 -3.084770 1.506979
 H -2.976576 3.272395 0.860250
 H -1.170406 5.027306 1.695896
 H 1.197975 5.262787 2.262007
 H 2.888071 3.615922 1.551019
 H -1.765185 -3.792584 0.884753
 H 1.008199 -4.388739 1.582014
 C 4.524654 -5.343309 0.005416
 H 4.327644 -6.408022 -0.056863
 H 3.890556 -4.700164 -0.593152
 C 5.012038 -2.432079 0.392595
 H 4.634122 -2.695877 -0.594156
 H 5.553048 -1.487430 0.345391
 C 5.766153 -3.485449 1.025207
 H 6.569368 -3.219144 1.702790
 C 5.530412 -4.866451 0.784882
 H 6.141228 -5.573240 1.340182
 O 4.488203 -3.623089 3.066831
 O 2.749169 -4.974880 3.124966
 N 3.338719 -3.981217 2.667433
 H 4.022174 -1.556226 2.153167
```

Transition state structure for the reaction of butadiene with Fragment 2

Molecular formula: C34H16
Charge = 0 Multiplicity = 1
Hartree/particle
Thermal correction to Energy= 0.399111
Thermal correction to Enthalpy= 0.400055
Thermal correction to Gibbs Free Energy= 0.331085
Sum of electronic and zero-point Energies= -1304.672868
Sum of electronic and thermal Energies= -1304.652091
Sum of electronic and thermal Enthalpies= -1304.651146
Sum of electronic and thermal Free Energies= -1304.720117

E (Thermal) KCal/Mol CV Cal/Mol-Kelvin S Cal/Mol-Kelvin
250.446 93.382 145.160

Cartesian coordinates

Atom	X	Y	Z
C	1.237558	-0.175533	2.401603
C	0.523254	1.070295	2.385807
C	-0.880014	0.776329	2.383144
C	-1.035143	-0.650319	2.394850
C	0.272363	-1.238852	2.404440
C	0.989910	2.100369	1.599973
C	0.097929	2.996555	0.884516
C	-1.273365	2.713802	0.892481
C	-1.729218	1.537931	1.611747
C	-2.863317	0.962516	0.912382
C	-3.014804	-0.429709	0.923907
C	-2.029331	-1.224056	1.634136
C	-1.837962	-2.481762	0.934449
C	-0.559224	-3.052169	0.932877
C	0.505611	-2.357266	1.635601
C	1.757637	-2.570649	0.936474
C	2.696466	-1.535035	0.934549
C	2.379528	-0.305577	1.638437
C	2.257496	2.029014	0.900000
C	2.951902	0.815931	0.915740
C	0.958353	3.685624	-0.086552
C	2.206425	3.125172	-0.078906
C	-2.343526	3.001215	-0.072845
C	-3.263104	1.988433	-0.061513
C	-3.627159	-1.361257	-0.034250
C	-2.946737	-2.548019	-0.027785
C	0.131605	-3.925419	-0.025519
C	1.470950	-3.645961	-0.024727
C	3.799535	-1.191618	0.016458
C	3.953144	0.229181	0.004523
H	0.629103	4.465079	-0.762992
H	-2.357239	3.847165	-0.749519
H	-4.113914	1.911990	-0.727846
H	-4.442979	-1.114857	-0.703189
H	-3.143127	-3.382130	-0.690629
H	-0.358101	-4.627172	-0.689826
H	2.199525	-4.095153	-0.689221
H	4.303630	0.753026	-0.874177
H	3.013184	3.396900	-0.749286
H	4.027408	-1.792281	-0.853273
C	5.883695	0.754808	1.085151
H	5.962380	1.830768	0.960292
C	5.575269	-2.099239	1.107487
H	5.422880	-3.169145	0.999756
C	6.799422	-0.085117	0.474580
H	7.536384	0.344640	-0.200886
C	6.648211	-1.483841	0.485603
H	7.275398	-2.071419	-0.181936
H	5.125245	-1.668605	1.993922
H	5.352441	0.443409	1.976219

Transition state structure for the reaction of butadiene with Fragment 2 substituted with one NH_2 group

```
Molecular formula: C34H17N
Charge = 0 Multiplicity = 1
                                                        Hartree/particle
Thermal correction to Energy= 0.417150
Thermal correction to Enthalpy= 0.418095
Thermal correction to Gibbs Free Energy= 0.345692
Sum of electronic and zero-point Energies= -1360.017897
Sum of electronic and thermal Energies= -1359.995513
Sum of electronic and thermal Enthalpies= -1359.994569
Sum of electronic and thermal Free Energies= -1360.066971

E (Thermal) KCal/Mol CV Cal/Mol-Kelvin S Cal/Mol-Kelvin
 261.766 99.189 152.383
```

Cartesian coordinates

```
Atom X Y Z
 C 0.982654 -0.315396 2.247161
 C 0.258917 0.923668 2.313020
 C -1.141403 0.619223 2.322377
 C -1.288343 -0.806284 2.259360
 C 0.020262 -1.384660 2.205100
 C 0.702286 1.999825 1.579713
 C -0.205972 2.926289 0.931949
 C -1.576676 2.632370 0.948777
 C -2.010986 1.417739 1.611906
 C -3.157014 0.872648 0.906050
 C -3.302399 -0.518956 0.846871
 C -2.299285 -1.346061 1.492754
 C -2.117356 -2.561401 0.724260
 C -0.834604 -3.126846 0.664627
 C 0.243874 -2.462360 1.377647
 C 1.483966 -2.641098 0.649503
 C 2.430139 -1.595864 0.695652
 C 2.102800 -0.391213 1.453212
 C 1.960814 1.974846 0.854245
 C 2.662202 0.772153 0.797721
 C 0.634532 3.674222 -0.012835
 C 1.888422 3.127596 -0.056281
 C -2.666540 2.963168 0.020674
 C -3.580927 1.945320 -0.004094
 C -3.930862 -1.400250 -0.147501
 C -3.244526 -2.581938 -0.217895
 C -0.164087 -3.938780 -0.351549
 C 1.178653 -3.652514 -0.363440
 C 3.463950 -1.178222 -0.222174
 C 3.699141 0.249100 -0.141220
 H 0.288955 4.487575 -0.639581
 H -2.697844 3.843455 -0.610136
 H -4.444410 1.899407 -0.656739
 H -4.761779 -1.122947 -0.784896
 H -3.450645 -3.380600 -0.920339
 H -0.663485 -4.600810 -1.048475
 H 1.883018 -4.053478 -1.082486
 H 2.682456 3.446976 -0.720768
 C 5.452299 0.596681 0.535083
 H 5.603830 1.585413 0.106903
 C 5.765966 -2.014125 1.960558
 H 5.914645 -2.983654 2.423718
 C 6.418000 -0.383083 0.199017
 H 7.070980 -0.214024 -0.653981
 C 6.542876 -1.610262 0.917301
 H 7.315041 -2.296859 0.568590
 H 5.001962 -1.383311 2.400719
 H 5.176108 0.652681 1.586237
 N 4.108187 -1.936756 -1.146755
 H 4.140363 -2.929173 -0.950470
 H 5.022247 -1.564896 -1.407751
 H 3.825740 0.756588 -1.094574
```

Transition state structure for the reaction of butadiene with Fragment 2 substituted with one OMe group

Molecular formula: C35H18O
Charge = 0 Multiplicity = 1

Hartree/particle

Thermal correction to Energy= 0.434131
Thermal correction to Enthalpy= 0.435075
Thermal correction to Gibbs Free Energy= 0.358628
Sum of electronic and zero-point Energies= -1419.157970
Sum of electronic and thermal Energies= -1419.134094
Sum of electronic and thermal Enthalpies= -1419.133150
Sum of electronic and thermal Free Energies= -1419.209598

E (Thermal) KCal/Mol CV Cal/Mol-Kelvin S Cal/Mol-Kelvin
272.421 103.064 160.898

Cartesian coordinates

Atom	X	Y	Z
C	1.024156	-0.415179	2.176191
C	0.277724	0.801310	2.324410
C	-1.116200	0.469207	2.336087
C	-1.236124	-0.952719	2.189540
C	0.083239	-1.499471	2.080482
C	0.688566	1.928289	1.651757
C	-0.245003	2.874082	1.072723
C	-1.608967	2.553879	1.092405
C	-2.010880	1.292758	1.688963
C	-3.159148	0.770793	0.970603
C	-3.279791	-0.617199	0.828965
C	-2.250125	-1.461986	1.406618
C	-2.054944	-2.619439	0.558706
C	-0.759669	-3.144352	0.437424
C	0.321563	-2.507605	1.173825
C	1.560415	-2.619141	0.424367
C	2.515919	-1.578361	0.555392
C	2.143016	-0.421977	1.378410
C	1.938351	1.972616	0.917286
C	2.667287	0.792037	0.793830
C	0.570808	3.693769	0.165057
C	1.834716	3.176760	0.077564
C	-2.720646	2.919773	0.204625
C	-3.616943	1.888150	0.133555
C	-3.907437	-1.447445	-0.208960
C	-3.197043	-2.606331	-0.365451
C	-0.097145	-3.864252	-0.644631
C	1.239600	-3.551593	-0.658598
C	3.593809	-1.077445	-0.266436
C	3.765415	0.355109	-0.117791
H	0.202766	4.535719	-0.408889
H	-2.778212	3.835424	-0.371560
H	-4.490218	1.865265	-0.507216
H	-4.754770	-1.148396	-0.814055
H	-3.397372	-3.363028	-1.114503
H	-0.597344	-4.474522	-1.386646
H	1.917834	-3.873870	-1.437033
H	2.614784	3.549553	-0.575512
C	5.371683	0.846875	0.755536
H	5.408433	1.891872	0.454059
C	5.912769	-1.867293	1.852355
H	6.158740	-2.860718	2.212403
C	6.490944	0.058930	0.405644
H	7.143797	0.395394	-0.395209
C	6.725426	-1.222030	0.960814
H	7.604916	-1.756073	0.602290
H	5.042530	-1.397898	2.295941
H	5.016590	0.739110	1.778409
O	4.316900	-1.684177	-1.217256
C	4.664273	-3.065012	-1.076920
H	5.295149	-3.299114	-1.935243
H	5.220115	-3.200168	-0.142200
H	3.790130	-3.715438	-1.077313
H	3.938637	0.896863	-1.044140

Transition state structure for the reaction of butadiene with Fragment 2 substituted with one OH group

Molecular formula: C34H16O
Charge = 0 Multiplicity = 1

Hartree/particle
Thermal correction to Energy= 0.403500
Thermal correction to Enthalpy= 0.404444
Thermal correction to Gibbs Free Energy= 0.332131
Sum of electronic and zero-point Energies= -1379.887495
Sum of electronic and thermal Energies= -1379.865405
Sum of electronic and thermal Enthalpies= -1379.864461
Sum of electronic and thermal Free Energies= -1379.936774

E (Thermal) KCal/Mol CV Cal/Mol-Kelvin S Cal/Mol-Kelvin
253.200 97.556 152.196

Cartesian coordinates

Atom	X	Y	Z
C	0.953272	-0.305689	2.300777
C	0.202624	0.917170	2.348778
C	-1.191816	0.582820	2.323486
C	-1.306336	-0.845285	2.260910
C	0.016704	-1.395374	2.242059
C	0.641123	2.003603	1.627689
C	-0.270692	2.909178	0.954354
C	-1.633220	2.584245	0.933028
C	-2.057965	1.359485	1.586291
C	-3.170944	0.787957	0.850239
C	-3.283303	-0.607061	0.790774
C	-2.281219	-1.409017	1.467555
C	-2.053405	-2.627007	0.710380
C	-0.761315	-3.167891	0.691728
C	0.282538	-2.476035	1.431433
C	1.539861	-2.630450	0.732689
C	2.455187	-1.561956	0.783181
C	2.093107	-0.359421	1.529798
C	1.916488	2.005102	0.933357
C	2.647100	0.818311	0.893115
C	0.578088	3.676074	0.031465
C	1.843978	3.157358	0.021566
C	-2.704886	2.889235	-0.024685
C	-3.593955	1.850190	-0.072831
C	-3.865754	-1.506607	-0.215523
C	-3.154440	-2.674454	-0.261323
C	-0.042276	-3.975100	-0.300123
C	1.292060	-3.659216	-0.278501
C	3.469951	-1.110618	-0.136039
C	3.679858	0.314005	-0.061244
H	0.231212	4.481670	-0.604439
H	-2.739648	3.768092	-0.657171
H	-4.437827	1.783749	-0.748926
H	-4.684252	-1.249632	-0.877042
H	-3.325416	-3.480492	-0.964638
H	-0.508879	-4.655355	-1.002222
H	2.041059	-4.040664	-0.960907
H	2.648605	3.494123	-0.621152
C	5.484696	0.585749	0.514299
H	5.655383	1.559483	0.061494
C	5.865797	-1.983946	2.017772
H	6.036637	-2.935052	2.509488
C	6.384517	-0.444617	0.128460
H	7.027818	-0.285514	-0.733810
C	6.563629	-1.638768	0.905684
H	7.301133	-2.347783	0.530261
H	5.129051	-1.331593	2.473977
H	5.271713	0.664811	1.578426
O	4.168261	-1.856699	-0.976812
H	5.096571	-1.471989	-0.979877
H	3.786419	0.822223	-1.016041

Transition state structure for the reaction of butadiene with Fragment 2 substituted with one Me group

Molecular formula: C35H18
Charge = 0 Multiplicity = 1

Hartree/particle

Thermal correction to Energy= 0.428556
Thermal correction to Enthalpy= 0.429500
Thermal correction to Gibbs Free Energy= 0.357960
Sum of electronic and zero-point Energies= -1343.962589
Sum of electronic and thermal Energies= -1343.940313
Sum of electronic and thermal Enthalpies= -1343.939369
Sum of electronic and thermal Free Energies= -1344.010909

E (Thermal) KCal/Mol CV Cal/Mol-Kelvin S Cal/Mol-Kelvin
268.923 99.202 150.569

Cartesian coordinates

Atom	X	Y	Z
C	0.960759	-0.262930	2.320594
C	0.238343	0.977273	2.347031
C	-1.162828	0.673696	2.354872
C	-1.308445	-0.753713	2.331159
C	0.002460	-1.332796	2.306913
C	0.687573	2.031329	1.583378
C	-0.218510	2.939289	0.902479
C	-1.587693	2.646947	0.920192
C	-2.026892	1.449800	1.614873
C	-3.166636	0.885405	0.915908
C	-3.309752	-0.507660	0.893236
C	-2.309858	-1.313777	1.569323
C	-2.119898	-2.550954	0.835946
C	-0.836461	-3.111350	0.800943
C	0.235100	-2.424899	1.502227
C	1.482306	-2.616380	0.784564
C	2.421072	-1.575801	0.803683
C	2.093856	-0.365311	1.541989
C	1.947335	1.986521	0.868163
C	2.649636	0.779183	0.847884
C	0.626322	3.659193	-0.060608
C	1.878392	3.108462	-0.081153
C	-2.672257	2.952338	-0.023215
C	-3.585214	1.933510	-0.026012
C	-3.929421	-1.418341	-0.080142
C	-3.241340	-2.600392	-0.112668
C	-0.155404	-3.961469	-0.181228
C	1.183210	-3.675640	-0.192815
C	3.527728	-1.204975	-0.099943
C	3.667952	0.226315	-0.070919
H	0.284029	4.453589	-0.712791
H	-2.700148	3.815391	-0.677490
H	-4.444234	1.868797	-0.683021
H	-4.755741	-1.160473	-0.731613
H	-3.441325	-3.418636	-0.794002
H	-0.649008	-4.654583	-0.851674
H	1.899867	-4.114557	-0.874876
H	2.675685	3.404142	-0.752766
C	5.517625	0.827495	0.893404
H	5.592212	1.876542	0.620629
C	5.263854	-1.983086	1.332909
H	5.125243	-3.058751	1.386761
C	6.482108	-0.069042	0.445030
H	7.231837	0.270427	-0.266487
C	6.354857	-1.450941	0.673738
H	7.031459	-2.122801	0.149613
H	4.726250	-1.416710	2.082583
H	5.023291	0.654726	1.842712
C	3.912471	-1.992685	-1.324433
H	4.028382	-3.058480	-1.112965
H	3.150028	-1.887031	-2.108423
H	4.859262	-1.627467	-1.732357
H	3.948237	0.753802	-0.975104

Transition state structure for the reaction of butadiene with Fragment 2 substituted with one *i*-Pr group

Molecular formula: C37H22
Charge = 0 Multiplicity = 1

Hartree/particle

Thermal correction to Energy= 0.488329
Thermal correction to Enthalpy= 0.489273
Thermal correction to Gibbs Free Energy= 0.412596
Sum of electronic and zero-point Energies= -1422.528457
Sum of electronic and thermal Energies= -1422.503654
Sum of electronic and thermal Enthalpies= -1422.502710
Sum of electronic and thermal Free Energies= -1422.579387

E (Thermal) KCal/Mol CV Cal/Mol-Kelvin S Cal/Mol-Kelvin
306.431 109.291 161.381

Cartesian coordinates

Atom	X	Y	Z
C	0.959080	-0.528331	2.328942
C	0.265071	0.724946	2.418204
C	-1.136945	0.448476	2.532336
C	-1.311751	-0.975385	2.511247
C	-0.017859	-1.578237	2.379958
C	0.673896	1.774657	1.626912
C	-0.263050	2.704119	1.021185
C	-1.632326	2.439090	1.144114
C	-2.040163	1.246359	1.865308
C	-3.241843	0.709440	1.254576
C	-3.414401	-0.680436	1.235995
C	-2.380639	-1.509781	1.826910
C	-2.271810	-2.743938	1.073231
C	-1.004918	-3.324240	0.931319
C	0.133134	-2.662876	1.547175
C	1.320661	-2.875585	0.736811
C	2.290587	-1.858472	0.700599
C	2.030027	-0.648081	1.468741
C	1.875399	1.710721	0.820833
C	2.551065	0.491295	0.746537
C	0.520830	3.413257	0.000139
C	1.757266	2.839550	-0.116236
C	-2.780360	2.771720	0.289061
C	-3.711080	1.771243	0.352580
C	-4.126456	-1.572763	0.309645
C	-3.465590	-2.767062	0.216351
C	-0.421527	-4.183200	-0.101220
C	0.918405	-3.927344	-0.214934
C	3.358778	-1.500652	-0.266058
C	3.525120	-0.062633	-0.222144
H	0.145864	4.217512	-0.621256
H	-2.841192	3.639043	-0.357285
H	-4.619533	1.727221	-0.236112
H	-4.996662	-1.295050	-0.272875
H	-3.733767	-3.576677	-0.451752
H	-0.979014	-4.864663	-0.732319
H	1.564951	-4.391878	-0.946297
H	2.507290	3.124309	-0.844686
C	5.313549	0.594965	0.682264
H	5.347512	1.640000	0.385295
C	5.184299	-2.199225	1.194800
H	5.104706	-3.274983	1.302904
C	6.333452	-0.263292	0.268073
H	7.074747	0.092350	-0.444292
C	6.261332	-1.638713	0.532052
H	6.983921	-2.295870	0.052183
H	4.601059	-1.626202	1.903204
H	4.865442	0.440567	1.657844
C	4.170385	-3.722453	-1.341336
H	4.001527	-4.330915	-2.236968
H	5.249397	-3.671742	-1.175661
H	3.726912	-4.243782	-0.490090
C	3.577779	-2.313458	-1.544542
H	2.562035	-2.462056	-1.945528
C	4.379041	-1.577368	-2.631342
H	5.378761	-1.309629	-2.277166
H	4.497852	-2.225320	-3.505818
H	3.878566	-0.665225	-2.969073
H	3.739197	0.473381	-1.137097

Transition state structure for the reaction of butadiene with Fragment 2 substituted with one F group

Molecular formula: C34H15F
Charge = 0 Multiplicity = 1

Hartree/particle

Thermal correction to Energy= 0.391645
Thermal correction to Enthalpy= 0.392589
Thermal correction to Gibbs Free Energy= 0.321655
Sum of electronic and zero-point Energies= -1403.908262
Sum of electronic and thermal Energies= -1403.886505
Sum of electronic and thermal Enthalpies= -1403.885560
Sum of electronic and thermal Free Energies= -1403.956494

E (Thermal) KCal/Mol CV Cal/Mol-Kelvin S Cal/Mol-Kelvin
245.761 96.568 149.293

Cartesian coordinates

Atom X Y Z
C 0.972280 -0.268470 2.389011
C 0.238062 0.965617 2.383863
C -1.160328 0.649818 2.359754
C -1.293306 -0.778881 2.343028
C 0.022981 -1.347967 2.358834
C 0.697484 2.016279 1.620723
C -0.201419 2.912050 0.912954
C -1.568333 2.608942 0.902181
C -2.013373 1.413023 1.594276
C -3.130414 0.833598 0.871194
C -3.258694 -0.560738 0.852513
C -2.268145 -1.353189 1.558026
C -2.048512 -2.593130 0.834840
C -0.761148 -3.143476 0.838068
C 0.280892 -2.448718 1.573145
C 1.546155 -2.632759 0.888913
C 2.465128 -1.587359 0.935440
C 2.126909 -0.366757 1.641897
C 1.971113 1.976160 0.929108
C 2.684902 0.773045 0.927966
C 0.655890 3.629343 -0.039385
C 1.911646 3.085828 -0.032205
C -2.632468 2.898733 -0.068893
C -3.536096 1.871858 -0.086946
C -3.844288 -1.481726 -0.131833
C -3.144208 -2.656969 -0.142047
C -0.041850 -3.982140 -0.131431
C 1.293126 -3.684240 -0.106287
C 3.588472 -1.200376 0.066085
C 3.682348 0.221539 0.002237
H 0.320649 4.415390 -0.705116
H -2.652054 3.757263 -0.729323
H -4.378444 1.795099 -0.763859
H -4.656035 -1.234620 -0.805385
H -3.318565 -3.480003 -0.824536
H -0.512454 -4.670223 -0.823240
H 2.041985 -4.089100 -0.774832
H 2.719516 3.379414 -0.691905
C 5.736636 0.857152 0.908813
H 5.857720 1.892824 0.605296
C 5.238259 -1.930437 1.354823
H 5.027147 -2.993651 1.420052
C 6.567794 -0.120999 0.398625
H 7.297455 0.152751 -0.360480
C 6.331840 -1.490681 0.627109
H 6.895201 -2.211816 0.040009
H 4.842026 -1.341553 2.174887
H 5.212123 0.717142 1.845579
F 3.881327 -1.945350 -1.030176
H 3.981410 0.714455 -0.911502

Transition state structure for the reaction of butadiene with Fragment 2 substituted with one COOH group

Molecular formula: C35H16O2
Charge = 0 Multiplicity = 1

Hartree/particle

Thermal correction to Energy= 0.417255
Thermal correction to Enthalpy= 0.418199
Thermal correction to Gibbs Free Energy= 0.343411
Sum of electronic and zero-point Energies= -1493.237137
Sum of electronic and thermal Energies= -1493.213528
Sum of electronic and thermal Enthalpies= -1493.212584
Sum of electronic and thermal Free Energies= -1493.287371

E (Thermal) KCal/Mol CV Cal/Mol-Kelvin S Cal/Mol-Kelvin
261.831 103.456 157.404

Cartesian coordinates

Atom	X	Y	Z
C	0.940695	-0.240259	2.292286
C	0.227547	1.003683	2.317000
C	-1.175714	0.710280	2.373173
C	-1.330139	-0.714849	2.384955
C	-0.021310	-1.301768	2.329980
C	0.660142	2.045444	1.528667
C	-0.256862	2.946262	0.854836
C	-1.625433	2.659661	0.909086
C	-2.052990	1.476646	1.638603
C	-3.212074	0.905747	0.981524
C	-3.363959	-0.487516	0.992664
C	-2.352477	-1.284302	1.661247
C	-2.190610	-2.543115	0.954453
C	-0.914062	-3.112980	0.896031
C	0.184350	-2.410331	1.543434
C	1.404289	-2.629958	0.789200
C	2.353405	-1.590318	0.749062
C	2.049068	-0.363873	1.478513
C	1.903331	1.984577	0.789134
C	2.598542	0.774510	0.779654
C	0.572620	3.649464	-0.135594
C	1.823799	3.098170	-0.171057
C	-2.732728	2.952796	-0.011311
C	-3.648770	1.937618	0.029119
C	-4.016216	-1.417658	0.059995
C	-3.338256	-2.606086	0.038912
C	-0.265173	-4.002519	-0.071849
C	1.074082	-3.726841	-0.134771
C	3.422584	-1.212569	-0.184320
C	3.641465	0.218186	-0.124936
H	0.219911	4.434582	-0.793429
H	-2.773900	3.803064	-0.681268
H	-4.524058	1.863864	-0.605113
H	-4.857163	-1.169466	-0.576304
H	-3.562009	-3.438428	-0.617394
H	-0.781838	-4.716191	-0.702074
H	1.786214	-4.167647	-0.816511
H	2.608574	3.382182	-0.861932
C	5.297340	0.701646	0.872334
H	5.298153	1.782227	0.756226
C	5.547169	-2.173228	1.129684
H	5.556745	-3.256039	1.066050
C	6.375337	-0.016376	0.336720
H	7.044572	0.482482	-0.359740
C	6.473206	-1.417111	0.457930
H	7.229475	-1.925635	-0.135516
H	4.874431	-1.749456	1.862931
H	4.895312	0.379283	1.828640
O	3.671590	-3.195416	-1.530885
O	4.730363	-1.302070	-2.129657
C	3.917015	-2.021958	-1.295863
H	5.005793	-1.933788	-2.814054
H	3.904049	0.729309	-1.042953

Transition state structure for the reaction of butadiene with Fragment 2 substituted with one CF_3 group

Molecular formula: C35H15F3
Charge = 0 Multiplicity = 1

Hartree/particle

Thermal correction to Energy= 0.407660
Thermal correction to Enthalpy= 0.408604
Thermal correction to Gibbs Free Energy= 0.332393
Sum of electronic and zero-point Energies= -1641.705110
Sum of electronic and thermal Energies= -1641.680850
Sum of electronic and thermal Enthalpies= -1641.679905
Sum of electronic and thermal Free Energies= -1641.756116

E (Thermal) KCal/Mol CV Cal/Mol-Kelvin S Cal/Mol-Kelvin
255.810 105.933 160.399

Cartesian coordinates

Atom	X	Y	Z
C	0.946430	-0.241081	2.279703
C	0.219839	0.996157	2.287592
C	-1.180560	0.687459	2.318658
C	-1.320058	-0.740074	2.329415
C	-0.004946	-1.313555	2.301555
C	0.656091	2.037241	1.499525
C	-0.260580	2.926839	0.808369
C	-1.627185	2.627158	0.843323
C	-2.053956	1.442119	1.568133
C	-3.196402	0.857133	0.892209
C	-3.332904	-0.537056	0.902841
C	-2.323507	-1.321656	1.588926
C	-2.135341	-2.577978	0.884450
C	-0.851654	-3.133773	0.851238
C	0.224041	-2.422986	1.521719
C	1.462142	-2.627074	0.791672
C	2.392411	-1.580065	0.774524
C	2.070718	-0.355402	1.487244
C	1.909256	1.985640	0.775046
C	2.617236	0.780829	0.774208
C	0.573416	3.632570	-0.174952
C	1.828890	3.089888	-0.193773
C	-2.722105	2.906519	-0.096086
C	-3.628789	1.882612	-0.068618
C	-3.958053	-1.474894	-0.040991
C	-3.266544	-2.655281	-0.050463
C	-0.174649	-4.011545	-0.111085
C	1.161157	-3.722983	-0.146282
C	3.504897	-1.205957	-0.131785
C	3.635476	0.222972	-0.135506
H	0.221625	4.411332	-0.840674
H	-2.760660	3.754778	-0.768752
H	-4.492797	1.798676	-0.716789
H	-4.790949	-1.236451	-0.691428
H	-3.469758	-3.490579	-0.709600
H	-0.671744	-4.727020	-0.754772
H	1.877015	-4.171079	-0.820244
H	2.619125	3.376123	-0.877488
C	5.474818	0.822711	0.937245
H	5.513452	1.892781	0.755439
C	5.306269	-2.029071	1.123795
H	5.192753	-3.105816	1.067935
C	6.456588	-0.003748	0.411181
H	7.191727	0.419408	-0.269825
C	6.373090	-1.404382	0.506382
H	7.052159	-2.000734	-0.097619
H	4.773090	-1.553307	1.937462
H	4.956323	0.543619	1.846869
C	3.794935	-1.952364	-1.397217
F	4.045691	-3.274206	-1.205830
F	2.744909	-1.898164	-2.258783
F	4.859237	-1.435690	-2.051912
H	3.957417	0.742998	-1.027365

Transition state structure for the reaction of butadiene with Fragment 2 substituted with one CHO group

Molecular formula: C35H16O
Charge = 0 Multiplicity = 1

Hartree/particle
Thermal correction to Energy= 0.410814
Thermal correction to Enthalpy= 0.411758
Thermal correction to Gibbs Free Energy= 0.338820
Sum of electronic and zero-point Energies= -1417.991376
Sum of electronic and thermal Energies= -1417.968663
Sum of electronic and thermal Enthalpies= -1417.967719
Sum of electronic and thermal Free Energies= -1418.040657

E (Thermal) KCal/Mol CV Cal/Mol-Kelvin S Cal/Mol-Kelvin
257.790 99.886 153.512

Cartesian coordinates

Atom	X	Y	Z
C	0.957187	-0.200850	2.318331
C	0.242149	1.042574	2.325457
C	-1.161883	0.747987	2.339731
C	-1.315555	-0.677076	2.343674
C	-0.004187	-1.262674	2.323067
C	0.696405	2.086337	1.552465
C	-0.201005	2.988531	0.854884
C	-1.570344	2.700647	0.867867
C	-2.017887	1.515490	1.581458
C	-3.156383	0.945230	0.889170
C	-3.307098	-0.448234	0.892463
C	-2.314850	-1.245416	1.588361
C	-2.132079	-2.503764	0.883719
C	-0.854868	-3.072847	0.861376
C	0.224170	-2.371038	1.542154
C	1.462453	-2.583531	0.817504
C	2.411617	-1.542468	0.806949
C	2.087779	-0.321284	1.534590
C	1.961966	2.029956	0.850411
C	2.657481	0.820540	0.857914
C	0.657152	3.696702	-0.107025
C	1.909969	3.147834	-0.106542
C	-2.650563	2.995354	-0.083800
C	-3.566341	1.979292	-0.072899
C	-3.931360	-1.377084	-0.060472
C	-3.252749	-2.565264	-0.064478
C	-0.177707	-3.957088	-0.092917
C	1.161536	-3.677026	-0.120213
C	3.493411	-1.162539	-0.113706
C	3.736813	0.264739	-0.004619
H	0.323643	4.484097	-0.772099
H	-2.673055	3.847425	-0.752309
H	-4.422702	1.906466	-0.732509
H	-4.753476	-1.127969	-0.720548
H	-3.456935	-3.396238	-0.728750
H	-0.676110	-4.666784	-0.741969
H	1.893033	-4.100116	-0.792769
H	2.714713	3.436727	-0.771954
C	5.317236	0.685426	1.123716
H	5.260678	1.769838	1.168464
C	5.746079	-2.182632	0.969058
H	5.821978	-3.239486	0.737516
C	6.455763	0.116460	0.533159
H	7.116374	0.754593	-0.049416
C	6.631280	-1.277151	0.437067
H	7.426037	-1.646452	-0.207605
H	5.046248	-1.918803	1.750635
H	4.899038	0.205661	2.004397
O	3.672130	-3.044640	-1.603856
C	4.010987	-1.921880	-1.248472
H	4.769925	-1.360609	-1.835467
H	4.053706	0.801181	-0.892835

Transition state structure for the reaction of butadiene with Fragment 2 substituted with one CN group

Molecular formula: C35H15N
Charge = 0 Multiplicity = 1

Hartree/particle

Thermal correction to Energy= 0.399695
Thermal correction to Enthalpy= 0.400639
Thermal correction to Gibbs Free Energy= 0.327478
Sum of electronic and zero-point Energies= -1396.918032
Sum of electronic and thermal Energies= -1396.895326
Sum of electronic and thermal Enthalpies= -1396.894382
Sum of electronic and thermal Free Energies= -1396.967543

E (Thermal) KCal/Mol CV Cal/Mol-Kelvin S Cal/Mol-Kelvin
250.812 99.424 153.981

Cartesian coordinates

Atom	X	Y	Z
C	0.965871	-0.246719	2.324630
C	0.240978	0.990742	2.327165
C	-1.160825	0.684316	2.320265
C	-1.302532	-0.743035	2.309719
C	0.011650	-1.318961	2.308638
C	0.697526	2.042110	1.564562
C	-0.199735	2.941782	0.862522
C	-1.566773	2.644277	0.858844
C	-2.013274	1.450359	1.558003
C	-3.140113	0.877322	0.847364
C	-3.279099	-0.516406	0.836211
C	-2.288819	-1.312894	1.536990
C	-2.085691	-2.559263	0.818916
C	-0.802918	-3.118590	0.809061
C	0.256678	-2.421511	1.521323
C	1.507197	-2.614442	0.816163
C	2.438118	-1.565469	0.833219
C	2.110958	-0.350014	1.559183
C	1.968001	1.996791	0.868844
C	2.672864	0.792528	0.874092
C	0.660022	3.659150	-0.090731
C	1.914920	3.116139	-0.085749
C	-2.638272	2.938842	-0.102384
C	-3.546944	1.916408	-0.110077
C	-3.882397	-1.438947	-0.136291
C	-3.192208	-2.619977	-0.145984
C	-0.104706	-3.970834	-0.160521
C	1.232427	-3.677457	-0.160617
C	3.517115	-1.184707	-0.083439
C	3.727359	0.247082	-0.025512
H	0.325947	4.447453	-0.754348
H	-2.659342	3.796090	-0.764239
H	-4.395250	1.843054	-0.779888
H	-4.698437	-1.189304	-0.803585
H	-3.379581	-3.445186	-0.822247
H	-0.587530	-4.666110	-0.836234
H	1.964154	-4.096441	-0.839245
H	2.722422	3.411517	-0.744819
C	5.413981	0.746695	0.975188
H	5.423049	1.825078	0.840828
C	5.586975	-2.127342	1.251377
H	5.570714	-3.210630	1.195178
C	6.466883	0.000774	0.434894
H	7.142481	0.478947	-0.270394
C	6.522669	-1.402466	0.556906
H	7.247097	-1.937407	-0.052289
H	4.950860	-1.683782	2.005915
H	4.997599	0.438695	1.929581
C	3.906931	-1.957124	-1.209623
N	4.203073	-2.583450	-2.148708
H	3.986835	0.766280	-0.940760

Transition state structure for the reaction of butadiene with Fragment 2 substituted with one NO_2 group

Molecular formula: C34H15NO2
Charge = 0 Multiplicity = 1
Hartree/particle
Thermal correction to Energy= 0.404398
Thermal correction to Enthalpy= 0.405343
Thermal correction to Gibbs Free Energy= 0.330467
Sum of electronic and zero-point Energies= -1509.176222
Sum of electronic and thermal Energies= -1509.152750
Sum of electronic and thermal Enthalpies= -1509.151806
Sum of electronic and thermal Free Energies= -1509.226682

E (Thermal) KCal/Mol CV Cal/Mol-Kelvin S Cal/Mol-Kelvin
253.764 102.065 157.589

Cartesian coordinates

Atom	X	Y	Z
C	0.939102	-0.271331	2.282290
C	0.221489	0.967850	2.332393
C	-1.181984	0.668524	2.358608
C	-1.331045	-0.756703	2.323724
C	-0.019742	-1.337986	2.273477
C	0.663072	2.039151	1.589260
C	-0.246461	2.958149	0.930119
C	-1.613472	2.663242	0.949409
C	-2.048616	1.454720	1.633559
C	-3.193973	0.901631	0.938967
C	-3.341022	-0.491551	0.903507
C	-2.339017	-1.306363	1.565218
C	-2.160990	-2.541654	0.822606
C	-0.882089	-3.107461	0.770254
C	0.202536	-2.424127	1.460731
C	1.436720	-2.621759	0.723753
C	2.381075	-1.577118	0.731986
C	2.060831	-0.364710	1.483766
C	1.915674	2.005677	0.866095
C	2.618177	0.798484	0.830019
C	0.596412	3.695784	-0.023855
C	1.850069	3.150304	-0.057344
C	-2.706263	2.982128	0.020791
C	-3.618107	1.962456	0.012906
C	-3.973630	-1.393427	-0.069343
C	-3.291329	-2.578602	-0.115482
C	-0.214234	-3.961872	-0.215250
C	1.126094	-3.682986	-0.243858
C	3.440951	-1.143672	-0.162913
C	3.656310	0.279670	-0.099847
H	0.251552	4.500779	-0.661433
H	-2.739922	3.853447	-0.621816
H	-4.482088	1.906320	-0.638339
H	-4.803719	-1.127580	-0.712584
H	-3.500382	-3.390447	-0.801535
H	-0.717860	-4.652764	-0.880278
H	1.849758	-4.101472	-0.927234
H	2.645025	3.458897	-0.725453
C	5.363093	0.733932	0.750117
H	5.395038	1.794551	0.516783
C	5.814720	-2.095433	1.493300
H	5.948675	-3.158497	1.661395
C	6.372635	-0.073967	0.208279
H	6.962328	0.307572	-0.619399
C	6.582592	-1.430969	0.592134
H	7.338948	-1.985151	0.042924
H	5.066788	-1.604729	2.104785
H	5.047157	0.529417	1.768726
N	4.105930	-1.888667	-1.148702
O	3.871849	-3.101478	-1.288413
O	4.953934	-1.267350	-1.834882
H	3.880399	0.783765	-1.032230

Transition state structure for the reaction of butadiene with Fragment 1 substituted with two NH_2 group

Molecular formula: C34H20N2
Charge = 0 Multiplicity = 1

Hartree/particle
Thermal correction to Energy= 0.460535
Thermal correction to Enthalpy= 0.461479
Thermal correction to Gibbs Free Energy= 0.388109
Sum of electronic and zero-point Energies= -1416.621211
Sum of electronic and thermal Energies= -1416.597981
Sum of electronic and thermal Enthalpies= -1416.597037
Sum of electronic and thermal Free Energies= -1416.670406

E (Thermal) KCal/Mol CV Cal/Mol-Kelvin S Cal/Mol-Kelvin
288.990 105.093 154.419

Cartesian coordinates

Atom	X	Y	Z
C	-1.347409	-0.526112	-1.103618
C	-1.198957	0.895872	-1.117037
C	0.071563	1.425423	-1.093045
C	1.232679	0.587356	-1.023248
C	1.057852	-0.787740	-1.014529
C	-0.252920	-1.359851	-1.026012
C	2.242951	1.211627	-0.230717
C	3.159078	0.369591	0.426629
C	2.989324	-1.050262	0.432218
C	1.888487	-1.607239	-0.235548
C	3.822662	-1.993814	1.265636
C	3.032925	-3.267469	1.631232
C	1.726570	-3.576430	1.150541
C	1.134619	-2.778760	0.198657
C	-0.281836	-2.566646	-0.235331
C	-1.507697	-2.960299	0.304769
C	-2.662120	-2.080163	0.254653
C	-2.522947	-0.833966	-0.398742
C	-3.214188	0.370527	-0.037921
C	-4.310553	0.224164	0.795141
C	-4.635909	-1.075458	1.276762
C	-3.832091	-2.188820	1.061521
C	0.331655	2.588812	-0.349297
C	-0.726589	3.319130	0.235747
C	-2.067915	2.756505	0.171409
C	-2.295795	1.513579	-0.412239
C	-0.307495	4.360222	1.113076
C	1.035839	4.487698	1.449154
C	2.033309	3.565036	1.026593
C	1.684298	2.566124	0.131990
H	3.906935	0.772993	1.101941
H	-4.889589	1.076556	1.139620
H	-5.508977	-1.180957	1.914679
H	-4.065161	-3.123240	1.565666
H	-2.835318	3.229195	0.780636
H	-1.040894	5.014793	1.576935
H	1.326296	5.275665	2.138430
H	3.026773	3.623314	1.462788
H	-1.570144	-3.826993	0.959338
H	1.193419	-4.398107	1.624900
C	4.058020	-4.925258	-0.108337
H	3.562553	-5.888290	-0.183940
H	3.673747	-4.151969	-0.758809
C	5.186037	-2.362068	0.366166
H	4.821467	-2.441952	-0.659435
H	5.793212	-1.458877	0.449326
C	5.877791	-3.579665	0.796588
H	6.803001	-3.517821	1.362468
C	5.284531	-4.810624	0.536307
H	5.736900	-5.708371	0.957763
N	3.480004	-3.867317	2.788615
H	3.092457	-4.791360	2.944574
H	4.499888	-3.888181	2.847380
N	4.369206	-1.301598	2.440842
H	3.681328	-1.279994	3.189416
H	5.185011	-1.792573	2.797674

Transition state structure for the reaction of butadiene with Fragment 1 substituted with two OMe group

Molecular formula: C36H22O2
Charge = 0 Multiplicity = 1

Hartree/particle

Thermal correction to Energy= 0.493990
Thermal correction to Enthalpy= 0.494934
Thermal correction to Gibbs Free Energy= 0.415684
Sum of electronic and zero-point Energies= -1534.921484
Sum of electronic and thermal Energies= -1534.895533
Sum of electronic and thermal Enthalpies= -1534.894589
Sum of electronic and thermal Free Energies= -1534.973839

E (Thermal) KCal/Mol CV Cal/Mol-Kelvin S Cal/Mol-Kelvin
309.984 113.022 166.796

Cartesian coordinates

Atom X Y Z
C -1.391664 -0.511880 -1.051599
C -1.238373 0.905127 -1.161406
C 0.033810 1.430997 -1.186928
C 1.192366 0.594903 -1.070661
C 1.011561 -0.775534 -0.968658
C -0.299631 -1.343649 -0.930715
C 2.214051 1.269493 -0.334650
C 3.134043 0.468746 0.368201
C 2.949707 -0.944256 0.482316
C 1.842197 -1.543102 -0.137100
C 3.775026 -1.832923 1.377081
C 3.004055 -3.081661 1.809544
C 1.697615 -3.420095 1.376450
C 1.095600 -2.681768 0.374064
C -0.322708 -2.495466 -0.059160
C -1.546946 -2.847840 0.514694
C -2.697830 -1.967321 0.418210
C -2.561357 -0.767129 -0.317185
C -3.244956 0.461339 -0.030784
C -4.333765 0.375421 0.820296
C -4.658613 -0.888490 1.389665
C -3.861017 -2.017072 1.240926
C 0.305472 2.641371 -0.527126
C -0.744581 3.413214 0.017989
C -2.088255 2.852061 0.006039
C -2.326153 1.573306 -0.489295
C -0.313500 4.510714 0.817200
C 1.033889 4.657343 1.128661
C 2.024679 3.705265 0.759291
C 1.663469 2.647859 -0.060429
H 3.899632 0.922123 0.991710
H -4.906632 1.251066 1.112726
H -5.526073 -0.948341 2.041029
H -4.093303 -2.915226 1.807510
H -2.847808 3.368257 0.589233
H -1.040261 5.197614 1.243172
H 1.333777 5.490134 1.758625
H 3.023475 3.792033 1.178400
H -1.607524 -3.669755 1.224955
H 1.167879 -4.220561 1.882251
C 4.087640 -4.846710 0.184497
H 3.624457 -5.827573 0.142248
H 3.687419 -4.115599 -0.504469
C 5.156016 -2.230002 0.531241
H 4.820068 -2.369875 -0.497792
H 5.747233 -1.315604 0.593076
C 5.846881 -3.406417 1.048614
H 6.703049 -3.284474 1.703680
C 5.297030 -4.662768 0.847576
H 5.754346 -5.518564 1.341929
O 3.568851 -3.593195 2.924667
O 4.341826 -1.146366 2.492506
C 3.091544 -4.830515 3.434244
H 3.108871 -5.595190 2.648357
H 3.770150 -5.105863 4.242126
H 2.075289 -4.736132 3.833259
C 3.423125 -0.666426 3.470063
H 4.019176 -0.088118 4.180005
H 2.657773 -0.014942 3.032043
H 2.935576 -1.489011 4.004716

Transition state structure for the reaction of butadiene with Fragment 1 substituted with two OH group

```
Molecular formula: C34H18O2
Charge = 0 Multiplicity = 1
                                                         Hartree/particle
Thermal correction to Energy= 0.434778
Thermal correction to Enthalpy= 0.435722
Thermal correction to Gibbs Free Energy= 0.362613
Sum of electronic and zero-point Energies= -1456.377685
Sum of electronic and thermal Energies= -1456.354677
Sum of electronic and thermal Enthalpies= -1456.353732
Sum of electronic and thermal Free Energies= -1456.426841

E (Thermal) KCal/Mol CV Cal/Mol-Kelvin S Cal/Mol-Kelvin
 272.827 103.342 153.871
```

```
 Cartesian coordinates

Atom X Y Z
 C -1.359166 -0.482182 -1.134269
 C -1.211777 0.939699 -1.114275
 C 0.057784 1.470301 -1.068470
 C 1.218144 0.631805 -1.004550
 C 1.043654 -0.742971 -1.031043
 C -0.264805 -1.317476 -1.069532
 C 2.224264 1.246891 -0.196813
 C 3.142223 0.391890 0.442036
 C 2.965032 -1.025806 0.422296
 C 1.864841 -1.580873 -0.259274
 C 3.761837 -1.998846 1.228674
 C 2.937223 -3.198941 1.653757
 C 1.676158 -3.548013 1.138710
 C 1.112344 -2.756570 0.146556
 C -0.296010 -2.540200 -0.301162
 C -1.527668 -2.945998 0.220066
 C -2.681300 -2.065895 0.180323
 C -2.539931 -0.805515 -0.445694
 C -3.235092 0.390156 -0.063670
 C -4.336329 0.225311 0.759056
 C -4.663520 -1.085183 1.210005
 C -3.857444 -2.192826 0.976322
 C 0.310812 2.619279 -0.300834
 C -0.752906 3.336724 0.290048
 C -2.092857 2.772973 0.204404
 C -2.314729 1.541781 -0.405754
 C -0.341612 4.363573 1.187487
 C 0.999782 4.490185 1.532306
 C 2.002667 3.578616 1.099219
 C 1.660970 2.592482 0.187215
 H 3.904644 0.795633 1.103684
 H -4.918214 1.069454 1.118587
 H -5.540658 -1.205216 1.839661
 H -4.093051 -3.138073 1.458461
 H -2.865769 3.232662 0.816519
 H -1.079245 5.008348 1.658161
 H 1.284253 5.268358 2.234988
 H 2.994798 3.636508 1.538847
 H -1.594224 -3.826379 0.855498
 H 1.143126 -4.357329 1.628281
 C 4.066083 -5.111885 0.019970
 H 3.596618 -6.089767 0.028121
 H 3.661977 -4.399370 -0.685798
 C 5.113411 -2.450558 0.343601
 H 4.725125 -2.614137 -0.663383
 H 5.719055 -1.543383 0.355468
 C 5.789873 -3.615599 0.892196
 H 6.647713 -3.479848 1.543294
 C 5.229710 -4.883022 0.731095
 H 5.669296 -5.713987 1.280175
 O 3.377370 -3.783945 2.778857
 H 4.132896 -3.246371 3.083845
 O 4.381647 -1.437214 2.404538
 H 3.696077 -0.929082 2.866024
```

Transition state structure for the reaction of butadiene with Fragment 1 substituted with two Me group

```
Molecular formula: C36H22
Charge = 0 Multiplicity = 1
                                                       Hartree/particle
Thermal correction to Energy= 0.482865
Thermal correction to Enthalpy= 0.483809
Thermal correction to Gibbs Free Energy= 0.408575
Sum of electronic and zero-point Energies= -1384.518909
Sum of electronic and thermal Energies= -1384.494908
Sum of electronic and thermal Enthalpies= -1384.493963
Sum of electronic and thermal Free Energies= -1384.569197

E (Thermal) KCal/Mol CV Cal/Mol-Kelvin S Cal/Mol-Kelvin
 303.002 106.580 158.342
```

Cartesian coordinates

Atom	X	Y	Z
C	-1.426328	-0.479561	-1.122724
C	-1.293531	0.943871	-1.098027
C	-0.030131	1.486851	-1.028955
C	1.137089	0.659264	-0.950118
C	0.978644	-0.716638	-0.978370
C	-0.324222	-1.303800	-1.036872
C	2.120256	1.272333	-0.116170
C	3.030471	0.424163	0.539738
C	2.883419	-1.003607	0.515585
C	1.798063	-1.543181	-0.196184
C	3.683911	-1.984466	1.317969
C	2.925146	-3.226359	1.693517
C	1.626105	-3.509369	1.172974
C	1.054532	-2.729657	0.193839
C	-0.357195	-2.526822	-0.273036
C	-1.590117	-2.945662	0.228501
C	-2.753906	-2.077986	0.169158
C	-2.614611	-0.816258	-0.453549
C	-3.328435	0.371810	-0.081515
C	-4.441652	0.194397	0.722303
C	-4.762799	-1.119384	1.166948
C	-3.940639	-2.218072	0.946005
C	0.198738	2.633266	-0.249492
C	-0.881652	3.338256	0.325903
C	-2.214747	2.763394	0.213319
C	-2.414247	1.533013	-0.405822
C	-0.496166	4.361896	1.238192
C	0.836863	4.495539	1.610893
C	1.854488	3.593585	1.191815
C	1.538891	2.612629	0.265603
H	3.752176	0.856125	1.226068
H	-5.038043	1.032256	1.072823
H	-5.648834	-1.249147	1.782076
H	-4.173498	-3.166044	1.424230
H	-3.002346	3.212686	0.814394
H	-1.248033	4.997375	1.698962
H	1.101454	5.269888	2.325500
H	2.836161	3.653472	1.654050
H	-1.657428	-3.826454	0.863393
H	1.080731	-4.337902	1.619443
C	3.981351	-5.049325	0.251328
H	3.529581	-6.012675	0.467342
H	3.472344	-4.453454	-0.492117
C	5.017056	-2.411192	0.096719
H	4.383777	-2.626500	-0.761181
H	5.525895	-1.456704	-0.028042
C	5.825945	-3.495748	0.548056
H	6.795187	-3.295033	0.996660
C	5.280051	-4.777367	0.644710
H	5.840145	-5.544957	1.176167
C	3.303706	-3.915015	2.985380
H	4.372369	-4.142236	3.033697
H	3.072219	-3.279303	3.851095
H	2.752877	-4.851570	3.103114
C	4.481792	-1.362723	2.471932
H	5.090069	-0.526837	2.118399
H	3.815090	-0.983658	3.254192
H	5.163952	-2.084638	2.922514

Transition state structure for the reaction of butadiene with Fragment 1 substituted with two *i*-Pr group

Molecular formula: C40H30
Charge = 0 Multiplicity = 1
Hartree/particle
Thermal correction to Energy= 0.602601
Thermal correction to Enthalpy= 0.603545
Thermal correction to Gibbs Free Energy= 0.516945
Sum of electronic and zero-point Energies= -1541.632735
Sum of electronic and thermal Energies= -1541.603174
Sum of electronic and thermal Enthalpies= -1541.602230
Sum of electronic and thermal Free Energies= -1541.688830
E (Thermal) KCal/Mol CV Cal/Mol-Kelvin S Cal/Mol-Kelvin
378.138 127.440 182.264

Cartesian coordinates
Atom X Y Z
C -1.371835 -0.660600 -1.065945
C -1.211033 0.753132 -1.200571
C 0.065230 1.270203 -1.209809
C 1.214277 0.429679 -1.061501
C 1.031779 -0.936128 -0.932773
C -0.286204 -1.495874 -0.907850
C 2.221582 1.102305 -0.303585
C 3.113553 0.316337 0.435381
C 2.944363 -1.116050 0.584869
C 1.850205 -1.694541 -0.085039
C 3.700744 -1.992393 1.497814
C 3.155614 -3.389189 1.679423
C 1.725635 -3.614365 1.324578
C 1.093669 -2.838192 0.414720
C -0.334876 -2.633452 -0.026690
C -1.561235 -2.966038 0.541408
C -2.709014 -2.081014 0.411206
C -2.554100 -0.897345 -0.344757
C -3.236595 0.339697 -0.093004
C -4.338077 0.274939 0.743553
C -4.676734 -0.974700 1.334391
C -3.881002 -2.109153 1.220260
C 0.332448 2.489388 -0.562897
C -0.723007 3.277260 -0.051724
C -2.069995 2.724393 -0.079678
C -2.305769 1.438605 -0.557363
C -0.299934 4.385148 0.736818
C 1.041720 4.528421 1.073235
C 2.032222 3.562377 0.741131
C 1.679553 2.495083 -0.068349
H 3.851035 0.812417 1.052970
H -4.911105 1.158887 1.009705
H -5.553005 -1.017332 1.975131
H -4.123465 -2.994125 1.802982
H -2.836601 3.254356 0.481441
H -1.030174 5.084178 1.136320
H 1.334729 5.369920 1.694730
H 3.022750 3.649184 1.179565
H -1.633321 -3.773679 1.266534
H 1.226557 -4.457111 1.795554
C 3.864025 -4.516969 0.189211
H 3.308527 -5.446602 0.295077
H 3.431471 -3.869381 -0.569856
C 5.412414 -2.215624 -0.113606
H 4.576759 -2.198191 -0.796326
H 5.978294 -1.291549 -0.078411
C 6.029523 -3.429744 0.174906
H 7.069277 -3.444586 0.498427
C 5.272256 -4.594576 0.261820
H 5.742064 -5.515262 0.597679
C 2.607228 -3.879705 4.181552
H 1.548401 -3.762689 3.935428
H 2.688796 -4.662690 4.944113
H 2.947553 -2.947173 4.637815
C 3.411370 -0.484303 3.541959
H 3.823240 -0.297894 4.540344
H 3.219679 0.486611 3.080181
H 2.445475 -0.981078 3.661419
C 3.461590 -4.266530 2.953317
H 3.098994 -5.254076 2.642234
C 4.914426 -4.502352 3.426260
H 4.978021 -5.491362 3.893638
H 5.638805 -4.472532 2.615405
H 5.230910 -3.783316 4.187938
C 4.404441 -1.347778 2.719550
H 4.683639 -2.168876 3.372434
C 5.718610 -0.572331 2.501944
H 6.040993 -0.138851 3.455416
H 6.515179 -1.233655 2.154981
H 5.632037 0.249179 1.786306

Transition state structure for the reaction of butadiene with Fragment 1 substituted with two F group

Molecular formula: C34H16F2
Charge = 0 Multiplicity = 1

Hartree/particle
Thermal correction to Energy= 0.408827
Thermal correction to Enthalpy= 0.409771
Thermal correction to Gibbs Free Energy= 0.336442
Sum of electronic and zero-point Energies= -1504.419574
Sum of electronic and thermal Energies= -1504.396646
Sum of electronic and thermal Enthalpies= -1504.395702
Sum of electronic and thermal Free Energies= -1504.469031

E (Thermal) KCal/Mol CV Cal/Mol-Kelvin S Cal/Mol-Kelvin
256.543 101.293 154.333

Cartesian coordinates

Atom	X	Y	Z
C	-1.479151	-0.463182	-1.115390
C	-1.333810	0.958602	-1.084904
C	-0.063848	1.489971	-1.043397
C	1.097196	0.652324	-0.999422
C	0.924824	-0.721149	-1.042325
C	-0.383154	-1.297285	-1.069922
C	2.108359	1.259162	-0.185342
C	3.028499	0.403869	0.441716
C	2.857410	-1.014555	0.395035
C	1.756077	-1.566454	-0.287177
C	3.651531	-1.984531	1.190336
C	2.850512	-3.170234	1.595569
C	1.587593	-3.520729	1.122737
C	1.007246	-2.737037	0.126935
C	-0.405062	-2.523815	-0.306893
C	-1.628970	-2.935545	0.226539
C	-2.785797	-2.058740	0.202611
C	-2.652503	-0.794340	-0.416431
C	-3.346129	0.396605	-0.019242
C	-4.439205	0.223409	0.813032
C	-4.759357	-1.090241	1.258638
C	-3.952705	-2.194344	1.010003
C	0.193671	2.630366	-0.263036
C	-0.866182	3.340373	0.343364
C	-2.206029	2.776568	0.261380
C	-2.431279	1.552286	-0.360650
C	-0.449608	4.356364	1.250551
C	0.893451	4.478928	1.589754
C	1.893322	3.572460	1.140073
C	1.545854	2.599513	0.216666
H	3.770860	0.793796	1.131923
H	-5.019192	1.063902	1.183913
H	-5.629606	-1.216170	1.896524
H	-4.180271	-3.143056	1.489052
H	-2.974295	3.227802	0.885460
H	-1.184763	4.994649	1.733712
H	1.181509	5.247071	2.301786
H	2.886898	3.621488	1.576963
H	-1.686005	-3.818854	0.858796
H	1.075130	-4.342776	1.612744
C	4.189249	-5.178449	0.101294
H	3.797741	-6.187519	0.175980
H	3.717310	-4.538630	-0.632348
C	5.036757	-2.411264	0.261967
H	4.621997	-2.650095	-0.717910
H	5.570280	-1.461347	0.222503
C	5.771816	-3.482724	0.879875
H	6.578374	-3.242711	1.565190
C	5.307125	-4.803742	0.809879
H	5.798908	-5.550481	1.429977
F	4.238684	-1.407372	2.315747
F	3.334442	-3.774706	2.691829

Transition state structure for the reaction of butadiene with Fragment 1 substituted with two COOH group

```
Molecular formula: C36H18O4
Charge = 0 Multiplicity = 1
                                                    Hartree/particle
Thermal correction to Energy= 0.459196
Thermal correction to Enthalpy= 0.460140
Thermal correction to Gibbs Free Energy= 0.378617
Sum of electronic and zero-point Energies= -1683.052598
Sum of electronic and thermal Energies= -1683.025833
Sum of electronic and thermal Enthalpies= -1683.024889
Sum of electronic and thermal Free Energies= -1683.106412

E (Thermal) KCal/Mol CV Cal/Mol-Kelvin S Cal/Mol-Kelvin
 288.150 115.796 171.580


 Cartesian coordinates

Atom X Y Z
 C -1.500710 -0.456006 -1.143204
 C -1.361408 0.966676 -1.137277
 C -0.098045 1.501522 -1.011194
 C 1.056722 0.670969 -0.865013
 C 0.894546 -0.704565 -0.870989
 C -0.410544 -1.284932 -0.987244
 C 1.998573 1.288709 0.018589
 C 2.867768 0.464776 0.736876
 C 2.727004 -0.982380 0.724774
 C 1.673806 -1.520708 -0.046416
 C 3.394037 -1.937637 1.584562
 C 2.861292 -3.335995 1.699376
 C 1.456947 -3.559904 1.206718
 C 0.906278 -2.716989 0.307767
 C -0.494106 -2.497906 -0.220024
 C -1.744827 -2.899460 0.237566
 C -2.901454 -2.025825 0.108352
 C -2.722226 -0.776477 -0.526444
 C -3.445473 0.421694 -0.210108
 C -4.597126 0.263419 0.542448
 C -4.947048 -1.039486 0.995101
 C -4.121478 -2.146368 0.833089
 C 0.098501 2.660015 -0.238619
 C -1.005373 3.381165 0.268222
 C -2.334748 2.812198 0.098458
 C -2.510356 1.572532 -0.509909
 C -0.659416 4.418831 1.180250
 C 0.653673 4.552214 1.618056
 C 1.684964 3.636722 1.267723
 C 1.409287 2.643601 0.342692
 H 3.544903 0.892687 1.463734
 H -5.204816 1.110457 0.848619
 H -5.861283 -1.153153 1.570605
 H -4.380967 -3.083993 1.317579
 H -3.148575 3.275740 0.651693
 H -1.429484 5.066732 1.590838
 H 0.886221 5.337390 2.331795
 H 2.642045 3.696413 1.778409
 H -1.844600 -3.768902 0.883370
 H 0.935841 -4.432825 1.590122
 C 3.791743 -4.490828 0.749878
 H 3.307281 -5.436089 0.981211
 H 3.552611 -4.154977 -0.258794
 C 5.540164 -2.357131 -0.073503
 H 4.622465 -2.407090 -0.641184
 H 6.145608 -1.470141 -0.229232
 C 6.016290 -3.419585 0.660744
 H 7.022379 -3.367422 1.069462
 C 5.189730 -4.468686 1.073636
 H 5.566820 -5.189894 1.790229
 C 2.906909 -3.923071 3.127382
 C 4.472375 -1.644463 2.519372
 O 4.961242 -2.488871 3.267459
 O 3.263687 -5.041193 3.422408
 O 2.293421 -3.098574 4.006270
 H 2.334573 -3.552701 4.864676
 O 4.953318 -0.367956 2.529419
 H 5.650730 -0.375484 3.205025
```

Transition state structure for the reaction of butadiene with Fragment 1 substituted with two CF_3 group

Molecular formula: C36H16F6
Charge = 0 Multiplicity = 1

Hartree/particle
Thermal correction to Energy= 0.440664
Thermal correction to Enthalpy= 0.441608
Thermal correction to Gibbs Free Energy= 0.356404
Sum of electronic and zero-point Energies= -1979.989832
Sum of electronic and thermal Energies= -1979.961633
Sum of electronic and thermal Enthalpies= -1979.960689
Sum of electronic and thermal Free Energies= -1980.045893

E (Thermal) KCal/Mol CV Cal/Mol-Kelvin S Cal/Mol-Kelvin
276.521 120.397 179.326

Cartesian coordinates

Atom X Y Z
C -1.398424 -0.467746 -1.135493
C -1.267673 0.955741 -1.092970
C -0.005805 1.499440 -1.000404
C 1.158620 0.671060 -0.914380
C 1.005425 -0.703470 -0.960251
C -0.296921 -1.292228 -1.042218
C 2.127555 1.269467 -0.053127
C 3.033644 0.423836 0.602415
C 2.894165 -1.011850 0.553516
C 1.818612 -1.534990 -0.179808
C 3.681634 -2.026154 1.320690
C 2.952300 -3.283458 1.674016
C 1.614930 -3.514792 1.158676
C 1.069966 -2.724778 0.191829
C -0.341139 -2.521102 -0.294874
C -1.575203 -2.946269 0.192242
C -2.740988 -2.080969 0.124087
C -2.594402 -0.813572 -0.484252
C -3.314214 0.369744 -0.108780
C -4.436073 0.182226 0.680541
C -4.761018 -1.135768 1.108953
C -3.934833 -2.231143 0.887137
C 0.212270 2.634773 -0.200831
C -0.876738 3.331499 0.368323
C -2.207312 2.757555 0.229300
C -2.397884 1.535270 -0.408751
C -0.505068 4.341339 1.301387
C 0.821669 4.468839 1.697586
C 1.845023 3.571770 1.282289
C 1.543036 2.607323 0.335115
H 3.740414 0.853647 1.299252
H -5.037293 1.015780 1.032861
H -5.653099 -1.271995 1.713549
H -4.170332 -3.183662 1.354456
H -3.003411 3.198251 0.825364
H -1.263936 4.970017 1.759792
H 1.074845 5.231335 2.428556
H 2.818276 3.620887 1.762647
H -1.646668 -3.831759 0.819806
H 1.054338 -4.335039 1.587209
C 3.974033 -5.020199 0.453490
H 3.531056 -5.925660 0.855483
H 3.435964 -4.572989 -0.370673
C 5.033368 -2.476836 -0.139194
H 4.237608 -2.737053 -0.828976
H 5.473062 -1.506442 -0.341068
C 5.862808 -3.506023 0.337218
H 6.866049 -3.266539 0.677407
C 5.320396 -4.751210 0.657750
H 5.911143 -5.438129 1.257535
C 4.615037 -1.393671 2.360222
C 3.153145 -3.982264 3.027179
F 5.431599 -0.460060 1.797876
F 3.911647 -0.749107 3.318143
F 5.434254 -2.260710 2.974391
F 2.835153 -3.147783 4.046758
F 2.328338 -5.057299 3.143361
F 4.385517 -4.456172 3.284082

Transition state structure for the reaction of butadiene with Fragment 1 substituted with two CHO group

Molecular formula: C36H18O2
Charge = 0 Multiplicity = 1

Hartree/particle
Thermal correction to Energy= 0.476638
Thermal correction to Enthalpy= 0.477582
Thermal correction to Gibbs Free Energy= 0.402143
Sum of electronic and zero-point Energies= -1522.843870
Sum of electronic and thermal Energies= -1522.820649
Sum of electronic and thermal Enthalpies= -1522.819705
Sum of electronic and thermal Free Energies= -1522.895144

E (Thermal) KCal/Mol CV Cal/Mol-Kelvin S Cal/Mol-Kelvin
299.095 99.664 158.774

Cartesian coordinates

Atom	X	Y	Z
C	-1.815819	0.006933	-0.742634
C	-1.618409	1.417211	-0.755971
C	-0.353245	1.895996	-0.690287
C	0.765818	1.021284	-0.571048
C	0.551696	-0.326225	-0.559725
C	-0.773623	-0.852618	-0.622289
C	1.766288	1.606099	0.244813
C	2.617342	0.757603	0.926633
C	2.411696	-0.675574	0.934829
C	1.329492	-1.166438	0.227147
C	3.117980	-1.669151	1.775185
C	2.492439	-2.991594	1.914836
C	1.092371	-3.185135	1.473978
C	0.540226	-2.341111	0.604546
C	-0.877776	-2.057863	0.142784
C	-2.109096	-2.400172	0.639222
C	-3.234738	-1.479502	0.536735
C	-3.026653	-0.258095	-0.094898
C	-3.698253	0.963587	0.218544
C	-4.820708	0.859635	0.986416
C	-5.208894	-0.427508	1.457021
C	-4.439587	-1.550002	1.289368
C	-0.078758	3.055072	0.044129
C	-1.115769	3.821004	0.564441
C	-2.475701	3.300828	0.459700
C	-2.718329	2.079770	-0.113842
C	-0.692754	4.862299	1.434954
C	0.624398	4.952615	1.807779
C	1.603102	3.987485	1.437697
C	1.250960	3.002244	0.561599
H	3.332737	1.188244	1.600576
H	-5.383419	1.721147	1.300774
H	-6.106002	-0.500203	2.046235
H	-4.727942	-2.467667	1.772093
H	-3.240632	3.803185	1.027364
H	-1.408386	5.551886	1.848315
H	0.917360	5.736452	2.483724
H	2.571212	4.021679	1.906263
H	-2.222986	-3.253628	1.285689
H	0.569045	-4.014435	1.907937
C	3.411020	-4.247114	0.581774
H	2.859848	-5.165340	0.691135
H	3.076238	-3.643833	-0.240537
C	4.941671	-1.906957	0.440884
H	4.220920	-1.916840	-0.348806
H	5.491790	-0.985872	0.527998
C	5.510203	-3.088290	0.889557
H	6.462701	-3.059661	1.389425
C	4.768420	-4.251093	0.926384
H	5.176161	-5.114238	1.423531
C	2.819961	-3.860900	3.115136
C	4.010831	-1.154706	2.838932
O	4.398822	-1.752534	3.798418
O	1.997276	-4.538269	3.648071
H	3.853593	-3.884772	3.425507
H	4.333886	-0.124489	2.695975

Transition state structure for the reaction of butadiene with Fragment 1 substituted with two CN group

Molecular formula: C36H16N2
Charge = 0 Multiplicity = 1

Hartree/particle

Thermal correction to Energy= 0.424532
Thermal correction to Enthalpy= 0.425476
Thermal correction to Gibbs Free Energy= 0.346820
Sum of electronic and zero-point Energies= -1490.421025
Sum of electronic and thermal Energies= -1490.395979
Sum of electronic and thermal Enthalpies= -1490.395035
Sum of electronic and thermal Free Energies= -1490.473691

E (Thermal) KCal/Mol CV Cal/Mol-Kelvin S Cal/Mol-Kelvin
266.398 107.478 165.546

Cartesian coordinates

Atom	X	Y	Z
C	-1.451363	-0.492097	-1.106597
C	-1.297412	0.929218	-1.083933
C	-0.025790	1.454283	-1.024813
C	1.128077	0.610382	-0.952246
C	0.951299	-0.763906	-0.983321
C	-0.362328	-1.333668	-1.030397
C	2.123754	1.213322	-0.119240
C	3.025390	0.360768	0.531830
C	2.850407	-1.065338	0.494394
C	1.767713	-1.602887	-0.215491
C	3.610418	-2.036811	1.314226
C	2.934844	-3.343194	1.587457
C	1.571399	-3.594881	1.121507
C	1.005138	-2.785652	0.185823
C	-0.412568	-2.555491	-0.272017
C	-1.642090	-2.954398	0.247108
C	-2.794729	-2.070424	0.195082
C	-2.639302	-0.811653	-0.428052
C	-3.332696	0.387061	-0.052745
C	-4.441386	0.225558	0.760625
C	-4.778289	-1.082415	1.210065
C	-3.974679	-2.193592	0.983922
C	0.224446	2.599488	-0.248738
C	-0.842664	3.319751	0.332376
C	-2.183749	2.762975	0.230375
C	-2.405013	1.534642	-0.385731
C	-0.437292	4.338450	1.241387
C	0.899236	4.454798	1.607217
C	1.901564	3.538312	1.183928
C	1.565570	2.564812	0.257660
H	3.754511	0.760303	1.231616
H	-5.022974	1.071971	1.115018
H	-5.660007	-1.197596	1.833752
H	-4.216196	-3.137138	1.465892
H	-2.960838	3.224229	0.835745
H	-1.177702	4.984080	1.706220
H	1.177785	5.224356	2.321124
H	2.885531	3.581314	1.642241
H	-1.714664	-3.832842	0.884183
H	1.040666	-4.423221	1.579947
C	3.992467	-4.865168	0.342534
H	3.475768	-5.788206	0.586388
H	3.595796	-4.339054	-0.517345
C	5.233140	-2.362653	0.062581
H	4.573825	-2.533563	-0.781115
H	5.704251	-1.384102	0.053735
C	5.938234	-3.449966	0.606042
H	6.874629	-3.269738	1.126555
C	5.318332	-4.689294	0.740398
H	5.790160	-5.449596	1.356471
C	4.355625	-1.466974	2.415841
C	3.251581	-3.980193	2.841404
N	3.479595	-4.523798	3.844539
N	4.969007	-0.958360	3.262911

Transition state structure for the reaction of butadiene with Fragment 1 substituted with two NO_2 group

Molecular formula: C34H16N2O4
Charge = 0 Multiplicity = 1

Hartree/particle

Thermal correction to Energy= 0.466964
Thermal correction to Enthalpy= 0.467908
Thermal correction to Gibbs Free Energy= 0.393973
Sum of electronic and zero-point Energies= -1704.430568
Sum of electronic and thermal Energies= -1704.407805
Sum of electronic and thermal Enthalpies= -1704.406861
Sum of electronic and thermal Free Energies= -1704.480797

E (Thermal) KCal/Mol CV Cal/Mol-Kelvin S Cal/Mol-Kelvin
293.024 99.592 155.610

Cartesian coordinates

Atom X Y Z
C -1.159847 -0.689594 -1.170665
C -0.918713 0.708789 -1.302796
C 0.359598 1.155085 -1.298510
C 1.455510 0.259512 -1.113849
C 1.201925 -1.082378 -0.999762
C -0.144086 -1.568248 -0.993741
C 2.478514 0.883338 -0.365995
C 3.306625 0.060954 0.384459
C 3.051279 -1.341175 0.486840
C 1.962114 -1.873981 -0.157134
C 3.697649 -2.223127 1.560414
C 3.207962 -3.720804 1.357206
C 1.703799 -3.883705 1.130049
C 1.136459 -3.026553 0.304473
C -0.279152 -2.706010 -0.135939
C -1.509604 -2.964038 0.407444
C -2.607073 -2.018163 0.244960
C -2.368648 -0.861024 -0.487761
C -2.998115 0.402816 -0.271784
C -4.112687 0.398749 0.514763
C -4.533185 -0.830786 1.097218
C -3.801236 -1.987427 1.015991
C 0.676484 2.363230 -0.668723
C -0.330541 3.200996 -0.203017
C -1.704764 2.714249 -0.246420
C -1.990766 1.455731 -0.709461
C 0.133345 4.296726 0.575010
C 1.456534 4.376605 0.927927
C 2.400893 3.354143 0.630117
C 2.009208 2.313362 -0.160818
H 4.036544 0.498277 1.042542
H -4.644804 1.300783 0.761167
H -5.423008 -0.824763 1.701539
H -4.109878 -2.851376 1.578446
H -2.447335 3.286006 0.283970
H -0.556832 5.038751 0.937519
H 1.780352 5.202453 1.536102
H 3.373764 3.394270 1.087851
H -1.638638 -3.757815 1.122983
H 1.198464 -4.689182 1.623255
C 3.952854 -4.374954 0.155585
H 3.981537 -5.445003 0.332506
H 3.331551 -4.231163 -0.718201
C 5.231320 -2.121070 1.601857
H 5.505131 -1.075909 1.587800
H 5.601664 -2.516332 2.537426
C 5.884853 -2.845175 0.461759
H 6.872441 -2.518957 0.185849
C 5.328481 -3.865205 -0.150275
H 5.847297 -4.374523 -0.944639
O 1.988590 -1.748704 3.087514
O 2.750321 -4.668667 3.435227
O 4.721881 -4.855665 2.705224
O 3.940679 -1.173531 3.642167
N 3.158455 -1.678903 2.897377
N 3.590829 -4.488583 2.612678

Transition state structure for the reaction of butadiene with Fragment 2 substituted with two NH_2 group

Molecular formula: C34H18N2
Charge = 0 Multiplicity = 1

Hartree/particle
Thermal correction to Energy= 0.435154
Thermal correction to Enthalpy= 0.436098
Thermal correction to Gibbs Free Energy= 0.360891
Sum of electronic and zero-point Energies= -1415.349855
Sum of electronic and thermal Energies= -1415.325970
Sum of electronic and thermal Enthalpies= -1415.325026
Sum of electronic and thermal Free Energies= -1415.400233

E (Thermal) KCal/Mol CV Cal/Mol-Kelvin S Cal/Mol-Kelvin
273.063 104.938 158.286

Cartesian coordinates

Atom	X	Y	Z
C	1.198103	-0.335793	2.316520
C	0.454268	0.894673	2.301390
C	-0.940132	0.573259	2.362182
C	-1.064536	-0.854623	2.399633
C	0.253568	-1.417605	2.369569
C	0.870322	1.930163	1.493685
C	-0.069271	2.800868	0.806337
C	-1.434661	2.484980	0.868710
C	-1.836587	1.309056	1.616667
C	-2.981593	0.699076	0.966733
C	-3.100492	-0.696382	0.998642
C	-2.072805	-1.460957	1.682785
C	-1.879929	-2.723465	0.995123
C	-0.589387	-3.269530	0.957997
C	0.481413	-2.541157	1.611608
C	1.719194	-2.743514	0.875508
C	2.637115	-1.696569	0.839841
C	2.306788	-0.458443	1.512185
C	2.114509	1.886846	0.755588
C	2.847981	0.678328	0.771856
C	0.734300	3.491622	-0.202655
C	1.999212	2.957308	-0.234781
C	-2.544803	2.736893	-0.059611
C	-3.440065	1.703307	-0.003910
C	-3.725750	-1.652463	0.075059
C	-3.019505	-2.824802	0.073187
C	0.091342	-4.149177	-0.002022
C	1.425997	-3.849351	-0.050006
C	3.774863	-1.368571	-0.080330
C	3.773883	0.094305	-0.157891
H	0.363284	4.249879	-0.881489
H	-2.602309	3.574382	-0.744503
H	-4.312991	1.599908	-0.637159
H	-4.569761	-1.432631	-0.567587
H	-3.220928	-3.671474	-0.572106
H	-0.405528	-4.871577	-0.638632
H	2.149915	-4.279406	-0.728534
H	2.761532	3.233289	-0.953548
C	6.554669	0.693227	1.842815
H	7.041166	1.610682	2.155899
C	5.348358	-1.883107	0.807321
H	5.233102	-2.947799	0.607554
C	7.072484	-0.064091	0.838248
H	7.993172	0.288290	0.371317
C	6.535121	-1.282989	0.313631
H	7.081290	-1.773719	-0.488950
H	5.076929	-1.630403	1.830728
H	5.659673	0.411170	2.386883
N	4.530879	0.724694	-1.091566
H	4.755869	1.691829	-0.896925
H	5.346284	0.186509	-1.385104
N	3.892159	-2.141688	-1.301908
H	3.294771	-1.753632	-2.028668
H	4.848159	-2.133699	-1.648207

Transition state structure for the reaction of butadiene with Fragment 2 substituted with two OMe group

Molecular formula: C36H20O2
Charge = 0 Multiplicity = 1

Hartree/particle
Thermal correction to Energy= 0.469135
Thermal correction to Enthalpy= 0.470080
Thermal correction to Gibbs Free Energy= 0.389330
Sum of electronic and zero-point Energies= -1533.639687
Sum of electronic and thermal Energies= -1533.613411
Sum of electronic and thermal Enthalpies= -1533.612467
Sum of electronic and thermal Free Energies= -1533.693217

E (Thermal) KCal/Mol CV Cal/Mol-Kelvin S Cal/Mol-Kelvin
294.387 112.298 169.953

Cartesian coordinates

Atom	X	Y	Z
C	1.107474	-0.300792	2.385539
C	0.306947	0.894245	2.440401
C	-1.066199	0.503196	2.555044
C	-1.120412	-0.929667	2.561015
C	0.219884	-1.426476	2.454423
C	0.633900	1.962360	1.634900
C	-0.376789	2.802317	1.010421
C	-1.720294	2.419259	1.129196
C	-2.030044	1.209052	1.867051
C	-3.173823	0.557250	1.255742
C	-3.226104	-0.842883	1.262671
C	-2.132069	-1.569545	1.879480
C	-1.914074	-2.809065	1.156753
C	-0.602851	-3.292327	1.048011
C	0.462295	-2.522005	1.659802
C	1.669413	-2.650230	0.861294
C	2.516911	-1.547597	0.791850
C	2.178175	-0.349796	1.522103
C	1.840927	1.997415	0.837705
C	2.623102	0.824334	0.780804
C	0.348990	3.558068	-0.013267
C	1.633991	3.085665	-0.117004
C	-2.883874	2.634552	0.257983
C	-3.725345	1.558039	0.330949
C	-3.850123	-1.810883	0.349920
C	-3.092537	-2.949136	0.290385
C	0.072174	-4.129215	0.045689
C	1.387252	-3.764897	-0.059981
C	3.614325	-1.14709	-0.166240
C	3.526905	0.320943	-0.212625
H	-0.088200	4.315896	-0.652082
H	-3.013114	3.482040	-0.404476
H	-4.620965	1.425261	-0.263936
H	-4.733584	-1.619402	-0.247230
H	-3.286732	-3.792959	-0.360744
H	-0.416271	-4.876251	-0.568559
H	2.098706	-4.176865	-0.764076
H	2.362909	3.401834	-0.851299
C	6.419128	0.729996	1.256764
H	6.920800	1.684553	1.374468
C	5.092630	-1.871129	0.735480
H	4.814633	-2.915284	0.609469
C	6.959523	-0.258761	0.482754
H	7.907383	-0.051247	-0.013305
C	6.348047	-1.505320	0.201453
H	6.799215	-2.138690	-0.556925
H	4.875087	-1.507730	1.738574
H	5.503884	0.593777	1.820768
O	3.896169	1.123334	-1.227648
O	3.736392	-1.826312	-1.390540
C	5.089679	0.875280	-1.979319
H	4.949835	1.381178	-2.936118
H	5.938584	1.308781	-1.442156
H	5.261362	-0.192852	-2.128288
C	2.686256	-1.566355	-2.332768
H	1.709724	-1.862457	-1.936022
H	2.653448	-0.511051	-2.621986
H	2.917049	-2.175510	-3.209408

Transition state structure for the reaction of butadiene with Fragment 2 substituted with two OH group

Molecular formula: C34H16O2
Charge = 0 Multiplicity = 1

Hartree/particle
Thermal correction to Energy= 0.442374
Thermal correction to Enthalpy= 0.443318
Thermal correction to Gibbs Free Energy= 0.377938
Sum of electronic and zero-point Energies= -1446.002006
Sum of electronic and thermal Energies= -1445.982636
Sum of electronic and thermal Enthalpies= -1445.981692
Sum of electronic and thermal Free Energies= -1446.047072

E (Thermal) KCal/Mol CV Cal/Mol-Kelvin S Cal/Mol-Kelvin
277.594 89.133 137.604

Cartesian coordinates

Atom X Y Z
C 1.477698 -0.412786 2.604413
C 0.773273 0.824236 2.632653
C -0.626766 0.539634 2.574361
C -0.785359 -0.875636 2.507068
C 0.513541 -1.467052 2.529322
C 1.260584 1.882067 1.932959
C 0.393698 2.809348 1.234981
C -0.947419 2.535608 1.181012
C -1.434639 1.332942 1.824405
C -2.528646 0.798969 1.052544
C -2.679857 -0.565412 0.984718
C -1.737889 -1.397713 1.690118
C -1.520924 -2.611234 0.932791
C -0.272063 -3.177339 0.949561
C 0.762173 -2.527490 1.722903
C 2.035966 -2.721506 1.073652
C 2.968975 -1.728816 1.170593
C 2.630003 -0.515552 1.875791
C 2.528281 1.823861 1.255242
C 3.204692 0.628754 1.224297
C 1.297863 3.549919 0.322549
C 2.514022 2.987347 0.329955
C -1.998639 2.882988 0.194476
C -2.898519 1.892523 0.120419
C -3.272651 -1.457259 -0.042561
C -2.612952 -2.623691 -0.070790
C 0.468583 -4.040656 -0.003730
C 1.783297 -3.786785 0.069013
C 4.268332 -1.446992 0.426113
C 4.203864 0.125560 0.177137
H 1.000589 4.374759 -0.296085
H -2.000354 3.769187 -0.410540
H -3.732231 1.863409 -0.554727
H -4.072638 -1.181840 -0.702705
H -2.802818 -3.426313 -0.757288
H 0.008296 -4.724623 -0.690690
H 2.541239 -4.222177 -0.550356
H 3.339349 3.286509 -0.285903
C 5.571813 0.847458 0.158980
H 5.636603 1.375135 -0.785334
C 5.454197 -1.856778 1.310933
H 5.510708 -2.939293 1.281858
C 6.783988 -0.029871 0.326763
H 7.724070 0.396654 0.018236
C 6.743329 -1.238725 0.844712
H 7.648831 -1.809923 0.959126
H 5.265206 -1.580940 2.345519
H 5.585458 1.608680 0.935034
O 4.397342 -2.141165 -0.772642
H 4.098585 -1.582035 -1.474672
O 3.643261 0.327423 -1.113795
H 2.700157 0.297146 -1.063543

Transition state structure for the reaction of butadiene with Fragment 2 substituted with two Me group

```
Molecular formula: C36H20
Charge = 0 Multiplicity = 1
                                                  Hartree/particle
Thermal correction to Energy= 0.457977
Thermal correction to Enthalpy= 0.458921
Thermal correction to Gibbs Free Energy= 0.384520
Sum of electronic and zero-point Energies= -1383.249667
Sum of electronic and thermal Energies= -1383.225896
Sum of electronic and thermal Enthalpies= -1383.224951
Sum of electronic and thermal Free Energies= -1383.299352

E (Thermal) KCal/Mol CV Cal/Mol-Kelvin S Cal/Mol-Kelvin
 287.385 105.060 156.589
```

Cartesian coordinates

Atom	X	Y	Z
C	1.304335	-0.186839	2.311956
C	0.591687	1.058511	2.332105
C	-0.810436	0.766208	2.387473
C	-0.966042	-0.660152	2.396955
C	0.340003	-1.248901	2.347992
C	1.028143	2.087319	1.529386
C	0.107781	2.992354	0.863356
C	-1.263084	2.712588	0.927595
C	-1.690462	1.532824	1.656476
C	-2.852812	0.962722	1.000849
C	-3.004665	-0.429773	1.009964
C	-1.991343	-1.228521	1.674238
C	-1.828965	-2.481832	0.961021
C	-0.550692	-3.051487	0.902941
C	0.543445	-2.358202	1.559837
C	1.771710	-2.574099	0.817179
C	2.718540	-1.541522	0.796640
C	2.416496	-0.313510	1.508305
C	2.271768	2.022977	0.783908
C	2.973635	0.810015	0.777908
C	0.929220	3.696202	-0.127287
C	2.177959	3.138875	-0.174482
C	-2.372065	3.008315	0.009637
C	-3.291193	1.995698	0.051144
C	-3.656392	-1.356142	0.072835
C	-2.976983	-2.543361	0.045474
C	0.099574	-3.929483	-0.076083
C	1.439394	-3.656176	-0.126644
C	3.846180	-1.213070	-0.108239
C	4.000056	0.233634	-0.122769
H	0.576375	4.487045	-0.778124
H	-2.412574	3.858910	-0.660154
H	-4.168507	1.924414	-0.580511
H	-4.498835	-1.106066	-0.560828
H	-3.200966	-3.373856	-0.613182
H	-0.415395	-4.634299	-0.717649
H	2.133349	-4.119659	-0.815456
H	2.953232	3.433480	-0.869405
C	5.780738	0.754550	1.173561
H	5.864135	1.830591	1.051356
C	5.471655	-2.060601	1.207024
H	5.322169	-3.132398	1.114722
C	6.776177	-0.078975	0.682985
H	7.586364	0.348334	0.096082
C	6.623953	-1.474739	0.700882
H	7.322222	-2.081672	0.128511
H	4.949734	-1.608168	2.041650
H	5.177255	0.446974	2.018950
C	4.455639	0.947408	-1.373852
H	3.675425	0.917809	-2.146925
H	4.688969	1.995971	-1.177824
H	5.353080	0.485120	-1.791551
C	4.142665	-2.031402	-1.343559
H	4.157079	-3.101502	-1.125673
H	3.383215	-1.858561	-2.118483
H	5.114587	-1.770915	-1.768761

Transition state structure for the reaction of butadiene with Fragment 2 substituted with two *i*-Pr group

```
Molecular formula: C40H28
Charge = 0 Multiplicity = 1
                                                Hartree/particle
Thermal correction to Energy= 0.577703
Thermal correction to Enthalpy= 0.578647
Thermal correction to Gibbs Free Energy= 0.495174
Sum of electronic and zero-point Energies= -1540.363119
Sum of electronic and thermal Energies= -1540.334511
Sum of electronic and thermal Enthalpies= -1540.333567
Sum of electronic and thermal Free Energies= -1540.417039

E (Thermal) KCal/Mol CV Cal/Mol-Kelvin S Cal/Mol-Kelvin
 362.514 125.307 175.683

 Cartesian coordinates
Atom X Y Z
 C 1.027636 -0.450098 2.116077
 C 0.300684 0.767216 2.301757
 C -1.075092 0.435501 2.534597
 C 1.199644 -0.991892 2.481793
 C 0.097469 -1.538426 2.218547
 C 0.605091 1.842566 1.504642
 C -0.413030 2.762499 1.035565
 C -1.757056 2.448467 1.268948
 C -2.060567 1.218262 1.977478
 C -3.291467 0.658117 1.455369
 C -3.414288 -0.736845 1.400863
 C -2.305189 -1.544922 1.872007
 C -2.216448 -2.747272 1.066767
 C -0.945882 -3.273396 0.795339
 C 0.218809 -2.589062 1.338532
 C 1.348783 -2.744791 0.448939
 C 2.309548 -1.692458 0.386091
 C 2.027899 -0.496962 1.172418
 C 1.752242 1.859114 0.608859
 C 2.486476 0.672312 0.463184
 C 0.260724 3.559879 0.007386
 C 1.507922 3.054595 -0.231894
 C -2.988219 2.771210 0.534128
 C -3.873954 1.733472 0.638763
 C -4.171405 -1.624696 0.506139
 C -3.477300 -2.788006 0.313174
 C -0.420256 -4.071707 -0.307200
 C 0.903174 -3.765930 -0.508888
 C 3.348965 -1.336839 -0.552185
 C 3.612293 0.152202 -0.453655
 H -0.184806 4.386171 -0.533391
 H -3.136056 3.657925 -0.070431
 H -4.828441 1.676735 0.129228
 H -5.097907 -1.361518 0.010148
 H -3.771339 -3.584103 -0.360338
 H -1.001499 -4.746176 -0.924014
 H 1.498683 -4.181704 -1.309294
 H 2.172035 3.440836 -0.989887
 C 4.984982 0.528907 0.642572
 H 4.982305 1.615149 0.656991
 C 5.559061 -2.242384 1.136451
 H 5.740578 -3.307363 1.219427
 C 6.237554 -0.059865 0.277737
 H 6.959946 0.515173 -0.295442
 C 6.463624 -1.421320 0.494614
 H 7.365112 -1.870797 0.080439
 H 4.734312 -1.856796 1.718809
 H 4.582128 0.154241 1.582818
 C 4.075622 -3.736547 -1.150340
 H 3.853529 -4.495103 -1.910478
 H 5.149884 -3.763607 -0.958015
 H 3.561347 -4.013644 -0.230135
 C 4.532687 2.298491 -1.676971
 H 5.593634 2.245182 -1.418421
 H 4.467972 2.802407 -2.647069
 H 4.053615 2.943447 -0.939452
 C 3.680346 -2.333339 -1.674805
 H 2.713332 -2.464068 -2.190848
 C 3.905607 0.891015 -1.789279
 H 4.669976 0.297891 -2.285739
 C 2.669233 0.897319 -2.709299
 H 2.265169 -0.109883 -2.845463
 H 1.862646 1.518101 -2.311832
 H 2.934218 1.288480 -3.697764
 C 4.706802 -1.979868 -2.769805
 H 5.665210 -1.671159 -2.343874
 H 4.893803 -2.880017 -3.364896
 H 4.361853 -1.213479 -3.465092
```

Transition state structure for the reaction of butadiene with Fragment 2 substituted with two F group

Molecular formula: C34H14F2
Charge = 0 Multiplicity = 1

Hartree/particle

Thermal correction to Energy= 0.384305
Thermal correction to Enthalpy= 0.385249
Thermal correction to Gibbs Free Energy= 0.311638
Sum of electronic and zero-point Energies= -1503.137732
Sum of electronic and thermal Energies= -1503.114855
Sum of electronic and thermal Enthalpies= -1503.113910
Sum of electronic and thermal Free Energies= -1503.187521

E (Thermal) KCal/Mol CV Cal/Mol-Kelvin S Cal/Mol-Kelvin
241.155 99.740 154.927

Cartesian coordinates

Atom	X	Y	Z
C	1.265646	-0.177497	2.388173
C	0.548948	1.069143	2.372539
C	-0.854255	0.774181	2.380198
C	-1.009075	-0.652098	2.392643
C	0.298451	-1.241299	2.392240
C	1.007066	2.099121	1.581814
C	0.110879	2.991683	0.868021
C	-1.260509	2.710326	0.890248
C	-1.709896	1.535989	1.616028
C	-2.851100	0.960820	0.927769
C	-3.002298	-0.431146	0.940101
C	-2.009395	-1.225734	1.639926
C	-1.823635	-2.481459	0.935115
C	-0.544397	-3.050284	0.919860
C	0.523609	-2.358633	1.619765
C	1.770437	-2.573429	0.911309
C	2.708973	-1.543138	0.921253
C	2.407466	-0.307829	1.627559
C	2.269809	2.030151	0.872810
C	2.966016	0.822607	0.901357
C	0.962836	3.671300	-0.117044
C	2.211270	3.110915	-0.119548
C	-2.339253	2.997394	-0.064935
C	-3.259708	1.985572	-0.043098
C	-3.622750	-1.361004	-0.013871
C	-2.940877	-2.546953	-0.016663
C	0.140342	-3.913869	-0.051725
C	1.479769	-3.634342	-0.062004
C	3.810584	-1.194526	0.020995
C	3.960694	0.230728	0.007523
H	0.625191	4.440856	-0.800540
H	-2.358227	3.842142	-0.742854
H	-4.116815	1.908718	-0.701152
H	-4.444773	-1.113824	-0.674771
H	-3.141988	-3.379613	-0.679778
H	-0.355316	-4.605641	-0.721962
H	2.206400	-4.060124	-0.742016
H	3.011354	3.358656	-0.805221
C	5.879967	0.749191	1.165341
H	5.937536	1.825842	1.037714
C	5.556339	-2.088293	1.166774
H	5.375869	-3.152000	1.044694
C	6.762544	-0.078160	0.501403
H	7.460882	0.358167	-0.208485
C	6.604473	-1.481446	0.502936
H	7.186852	-2.063816	-0.206701
H	5.140457	-1.656538	2.069924
H	5.368563	0.421803	2.063005
F	4.327304	0.841001	-1.142080
F	4.027016	-1.884770	-1.122796

Transition state structure for the reaction of butadiene with Fragment 2 substituted with two COOH group

Molecular formula: C36H16O4
Charge = 0 Multiplicity = 1
Hartree/particle
Thermal correction to Energy= 0.434543
Thermal correction to Enthalpy= 0.435488
Thermal correction to Gibbs Free Energy= 0.353916
Sum of electronic and zero-point Energies= -1681.786522
Sum of electronic and thermal Energies= -1681.759807
Sum of electronic and thermal Enthalpies= -1681.758863
Sum of electronic and thermal Free Energies= -1681.840434

E (Thermal) KCal/Mol CV Cal/Mol-Kelvin S Cal/Mol-Kelvin
272.680 114.164 171.681

Cartesian coordinates

Atom X Y Z
C -0.494110 0.084687 -1.815893
C -1.346640 -1.064589 -1.756420
C -2.695687 -0.609229 -1.575053
C -2.674659 0.823141 -1.521939
C -1.313568 1.252384 -1.670403
C -0.922853 -2.186115 -1.082745
C -1.822585 -3.010805 -0.297620
C -3.135157 -2.563616 -0.113348
C -3.527129 -1.303180 -0.725902
C -4.486574 -0.634866 0.130868
C -4.468160 0.765488 0.183236
C -3.490656 1.471977 -0.623367
C -3.064461 2.667887 0.080896
C -1.735752 3.085336 -0.060624
C -0.849465 2.294326 -0.902289
C 0.496117 2.340973 -0.363466
C 1.309076 1.195281 -0.506352
C 0.734838 0.041390 -1.190428
C 0.420506 -2.307629 -0.556497
C 1.246804 -1.182384 -0.624128
C -0.935084 -3.863928 0.507662
C 0.364197 -3.466745 0.352989
C -4.107059 -2.770867 0.968673
C -4.885675 -1.654964 1.111606
C -4.842281 1.718960 1.237527
C -4.030796 2.819023 1.177628
C -0.837250 3.842331 0.813771
C 0.451602 3.412938 0.639997
C 2.452893 0.660464 0.219480
C 2.492661 -0.786624 0.113731
H -1.268801 -4.646527 1.178297
H -4.143570 -3.642356 1.610980
H -5.630566 -1.511901 1.885216
H -5.592023 1.538722 1.998421
H -4.042050 3.639525 1.884914
H -1.154698 4.573204 1.547561
H 1.307288 3.741959 1.210417
H 1.215131 -3.885962 0.874124
C 3.883885 -1.391231 -1.144176
H 3.778818 -2.454408 -0.960697
C 4.623765 1.384528 -1.808688
H 4.896819 2.424229 -1.954903
C 5.110469 -0.807027 -0.805961
H 5.774308 -1.347224 -0.138758
C 5.455990 0.527285 -1.157198
H 6.403611 0.907169 -0.782930
H 3.685117 1.070684 -2.247838
H 3.388240 -1.034966 -2.042318
O 4.324343 0.675584 1.647319
O 3.555826 -2.583383 1.397389
O 3.349115 2.652893 1.173317
O 2.139551 -1.171033 2.418124
C 3.437887 1.297673 1.066345
C 2.844818 -1.602775 1.352307
H 4.076776 2.905708 1.764588
H 2.395456 -1.747341 3.158429

Transition state structure for the reaction of butadiene with Fragment 2 substituted with two CF_3 group

Molecular formula: C36H14F6
Charge = 0 Multiplicity = 1

Hartree/particle

Thermal correction to Energy= 0.415949
Thermal correction to Enthalpy= 0.416894
Thermal correction to Gibbs Free Energy= 0.333133
Sum of electronic and zero-point Energies= -1978.725199
Sum of electronic and thermal Energies= -1978.697452
Sum of electronic and thermal Enthalpies= -1978.696507
Sum of electronic and thermal Free Energies= -1978.780268

E (Thermal) KCal/Mol CV Cal/Mol-Kelvin S Cal/Mol-Kelvin
261.012 118.569 176.289

Cartesian coordinates

Atom X Y Z
C 1.323786 -0.187644 2.233089
C 0.615193 1.054441 2.279454
C -0.786835 0.763871 2.384508
C -0.941679 -0.661075 2.394993
C 0.365040 -1.247667 2.296403
C 1.028951 2.081712 1.466743
C 0.088413 2.992210 0.836620
C -1.278111 2.714581 0.940961
C -1.685335 1.530455 1.679089
C -2.865642 0.963816 1.056362
C -3.017264 -0.429397 1.066986
C 1.985023 -1.227128 1.699574
C -1.842667 -2.481585 0.979332
C -0.567744 -3.047478 0.880763
C 0.547177 -2.351360 1.499195
C 1.755814 -2.573652 0.725427
C 2.713017 -1.544735 0.698937
C 2.413449 -0.312085 1.397115
C 2.255562 2.028087 0.691484
C 2.969631 0.817161 0.681229
C 0.884352 3.708023 -0.165024
C 2.134522 3.162500 -0.247509
C -2.414821 3.015057 0.059550
C -3.331411 2.001502 0.124386
C -3.696934 -1.356682 0.150189
C -3.019425 -2.544184 0.101517
C 0.054814 -3.932349 -0.108721
C 1.392891 -3.669299 -0.196774
C 3.892722 -1.216749 -0.152230
C 4.048252 0.231624 -0.165026
H 0.513927 4.502620 -0.801162
H -2.474977 3.867516 -0.606089
H -4.226241 1.932295 -0.482351
H -4.557557 -1.106424 -0.458356
H -3.262915 -3.373812 -0.550975
H -0.478006 -4.638680 -0.733609
H 2.064284 -4.135229 -0.900653
H 2.888778 3.463469 -0.957362
C 5.789692 0.755441 1.201392
H 5.876285 1.827715 1.069703
C 5.475684 -2.068210 1.231053
H 5.326399 -3.136626 1.126578
C 6.801799 -0.085480 0.763153
H 7.635659 0.336378 0.208661
C 6.647689 -1.479686 0.778883
H 7.368301 -2.085848 0.236758
H 4.900765 -1.605479 2.023592
H 5.128963 0.448422 2.002889
C 4.214474 -2.104204 -1.332193
C 4.548343 1.010285 -1.359120
F 4.158032 -3.420467 -0.988414
F 5.433282 -1.916849 -1.864791
F 3.312568 -1.931387 -2.328866
F 5.696889 0.556455 -1.887817
F 3.627099 1.018859 -2.352857
F 4.777320 2.313479 -1.037906

Transition state structure for the reaction of butadiene with Fragment 2 substituted with two CHO group

Molecular formula: C36H16O2
Charge = 0 Multiplicity = 1

Hartree/particle

Thermal correction to Energy= 0.421895
Thermal correction to Enthalpy= 0.422839
Thermal correction to Gibbs Free Energy= 0.345468
Sum of electronic and zero-point Energies= -1531.295427
Sum of electronic and thermal Energies= -1531.270627
Sum of electronic and thermal Enthalpies= -1531.269683
Sum of electronic and thermal Free Energies= -1531.347054

E (Thermal) KCal/Mol CV Cal/Mol-Kelvin S Cal/Mol-Kelvin
264.743 106.979 162.841

Cartesian coordinates

Atom	X	Y	Z
C	1.304360	-0.244931	2.390574
C	0.594761	0.996818	2.442687
C	-0.808976	0.707303	2.503709
C	-0.966580	-0.716743	2.489585
C	0.341568	-1.305176	2.416790
C	1.026907	2.051311	1.674407
C	0.109800	2.978499	1.038468
C	-1.259129	2.691417	1.087485
C	-1.686051	1.490616	1.788035
C	-2.846619	0.936309	1.121053
C	-3.000036	-0.457074	1.103108
C	-1.990278	-1.268926	1.755139
C	-1.833518	-2.516541	1.025462
C	-0.560540	-3.091359	0.958040
C	0.541494	-2.402032	1.614042
C	1.760006	-2.615708	0.856978
C	2.715021	-1.577047	0.833465
C	2.403621	-0.356025	1.562265
C	2.269132	2.008992	0.928952
C	2.950777	0.787935	0.877058
C	0.942735	3.728071	0.088335
C	2.195889	3.181544	0.034255
C	-2.369304	3.008747	0.178629
C	-3.284477	1.991884	0.194910
C	-3.656604	-1.368730	0.154598
C	-2.983169	-2.558900	0.110935
C	0.086370	-3.972878	-0.019089
C	1.427422	-3.705151	-0.075679
C	3.810855	-1.216201	-0.073947
C	4.028667	0.238452	-0.018724
H	0.599674	4.554345	-0.522382
H	-2.412229	3.875380	-0.469768
H	-4.160714	1.934268	-0.439601
H	-4.497761	-1.106446	-0.475638
H	-3.211141	-3.378505	-0.559635
H	-0.432243	-4.676770	-0.658524
H	2.142661	-4.141008	-0.756767
H	2.990594	3.530817	-0.612857
C	5.629428	0.753817	1.005616
H	5.582626	1.838834	0.955698
C	6.051176	-2.110124	1.134587
H	6.123250	-3.185684	1.015441
C	6.755794	0.128294	0.445310
H	7.405387	0.701372	-0.207809
C	6.928821	-1.268337	0.497308
H	7.713419	-1.705012	-0.116355
H	5.355865	-1.763724	1.887704
H	5.237428	0.362672	1.940326
O	4.044970	-3.232133	-1.347149
O	5.292070	0.765954	-2.013499
C	4.375214	-2.071767	-1.125058
C	4.299309	0.921676	-1.332511
H	5.140567	-1.580777	-1.750055
H	3.486793	1.597482	-1.666779

Transition state structure for the reaction of butadiene with Fragment 2 substituted with two CN group

Molecular formula: C36H14N2
Charge = 0 Multiplicity = 1

Hartree/particle

Thermal correction to Energy= 0.399884
Thermal correction to Enthalpy= 0.400828
Thermal correction to Gibbs Free Energy= 0.323964
Sum of electronic and zero-point Energies= -1489.153517
Sum of electronic and thermal Energies= -1489.129018
Sum of electronic and thermal Enthalpies= -1489.128074
Sum of electronic and thermal Free Energies= -1489.204938

E (Thermal) KCal/Mol CV Cal/Mol-Kelvin S Cal/Mol-Kelvin
250.931 105.544 161.774

Cartesian coordinates

Atom	X	Y	Z
C	1.275011	-0.181418	2.351271
C	0.563201	1.062629	2.351434
C	-0.842216	0.770481	2.380328
C	-0.997385	-0.654940	2.390906
C	0.312354	-1.243049	2.368490
C	1.014131	2.096183	1.562766
C	0.110122	2.999057	0.870686
C	-1.258613	2.715120	0.900946
C	-1.701612	1.533194	1.623683
C	-2.846640	0.961405	0.941918
C	-2.998270	-0.431226	0.952271
C	-2.001733	-1.225961	1.643852
C	-1.824302	-2.485828	0.938967
C	-0.548627	-3.057744	0.914969
C	0.529357	-2.360577	1.595507
C	1.765625	-2.577194	0.871088
C	2.704649	-1.541410	0.867005
C	2.404793	-0.309975	1.564197
C	2.266579	2.031169	0.836842
C	2.960977	0.817823	0.849236
C	0.955614	3.697750	-0.107280
C	2.204634	3.141873	-0.128492
C	-2.346926	3.007263	-0.041976
C	-3.264135	1.992865	-0.019333
C	-3.629009	-1.362579	0.005180
C	-2.951491	-2.550704	-0.001295
C	0.125678	-3.936961	-0.050358
C	1.465108	-3.663447	-0.077177
C	3.831794	-1.214639	-0.043099
C	3.989479	0.243456	-0.055432
H	0.614951	4.481389	-0.772774
H	-2.373247	3.856010	-0.714352
H	-4.125796	1.918610	-0.671617
H	-4.455582	-1.114580	-0.649608
H	-3.160784	-3.383634	-0.661244
H	-0.375872	-4.639635	-0.704394
H	2.179599	-4.107445	-0.757483
H	2.996314	3.410744	-0.815092
C	5.833415	0.748251	1.105262
H	5.901844	1.822735	0.967906
C	5.529301	-2.078808	1.132797
H	5.367981	-3.145815	1.016486
C	6.792177	-0.086173	0.539892
H	7.551532	0.346961	-0.105604
C	6.642502	-1.478380	0.553929
H	7.291293	-2.075782	-0.080955
H	5.056199	-1.641368	2.004163
H	5.281988	0.437878	1.984947
C	4.080398	-1.987230	-1.222347
C	4.391676	0.925170	-1.247842
N	4.274049	-2.646856	-2.161916
N	4.717285	1.512222	-2.199062

Transition state structure for the reaction of butadiene with Fragment 2 substituted with two NO_2 group

```
Molecular formula: C34H14N2O4
Charge = 0 Multiplicity = 1
                                                    Hartree/particle
Thermal correction to Energy= 0.409021
Thermal correction to Enthalpy= 0.409965
Thermal correction to Gibbs Free Energy= 0.328365
Sum of electronic and zero-point Energies= -1713.654808
Sum of electronic and thermal Energies= -1713.628471
Sum of electronic and thermal Enthalpies= -1713.627527
Sum of electronic and thermal Free Energies= -1713.709128

E (Thermal) KCal/Mol CV Cal/Mol-Kelvin S Cal/Mol-Kelvin
 256.665 111.186 171.743
```

Cartesian coordinates

Atom	X	Y	Z
C	0.953340	-0.395953	2.185942
C	0.211092	0.827299	2.221103
C	-1.187195	0.499930	2.256548
C	-1.306065	-0.927938	2.237646
C	0.018046	-1.482416	2.192054
C	0.630478	1.900526	1.469597
C	-0.297753	2.797624	0.806138
C	-1.656974	2.471947	0.825883
C	-2.067250	1.259103	1.521274
C	-3.199738	0.675411	0.831205
C	-3.317714	-0.720852	0.811246
C	-2.300242	-1.506862	1.483881
C	-2.095758	-2.747657	0.755532
C	-0.806542	-3.289683	0.713780
C	0.262760	-2.576437	1.397151
C	1.500694	-2.757392	0.661912
C	2.419193	-1.688812	0.653156
C	2.073466	-0.476393	1.385203
C	1.883503	1.892602	0.746177
C	2.609697	0.694789	0.727629
C	0.531595	3.550494	-0.146399
C	1.798872	3.036457	-0.176793
C	-2.755895	2.758642	-0.105061
C	-3.645065	1.718871	-0.104448
C	-3.929833	-1.647190	-0.151410
C	-3.223711	-2.818905	-0.182824
C	-0.119725	-4.139963	-0.262381
C	1.215173	-3.834803	-0.293055
C	3.459848	-1.250104	-0.256261
C	3.656888	0.166266	-0.177155
H	0.171734	4.345937	-0.787434
H	-2.807249	3.622149	-0.756729
H	-4.506234	1.638096	-0.756712
H	-4.763685	-1.406035	-0.799391
H	-3.414852	-3.642440	-0.859928
H	-0.608362	-4.846342	-0.922221
H	1.947705	-4.244719	-0.972300
H	2.590105	3.360020	-0.840224
C	5.396043	0.588319	0.849655
H	5.346197	1.652717	0.659681
C	6.183468	-2.214862	1.579464
H	6.477499	-3.243223	1.758687
C	6.423515	-0.134478	0.257166
H	6.949716	0.314862	-0.578768
C	6.793690	-1.474868	0.627007
H	7.588518	-1.932429	0.044024
H	5.385014	-1.826969	2.202855
H	5.013991	0.265383	1.812581
N	4.222159	-2.024606	-1.141873
N	3.853790	0.940816	-1.467888
O	5.117916	-1.421433	-1.777699
O	4.645671	1.877269	-1.501060
O	4.009240	-3.242924	-1.240454
O	3.095456	0.627439	-2.373343

Adduct of the reaction of butadiene with Fragment 1

Molecular formula: C34H18
Charge = 0 Multiplicity = 1
Hartree/particle
Thermal correction to Energy= 0.428248
Thermal correction to Enthalpy= 0.429192
Thermal correction to Gibbs Free Energy= 0.360282
Sum of electronic and zero-point Energies= -1306.005183
Sum of electronic and thermal Energies= -1305.984569
Sum of electronic and thermal Enthalpies= -1305.983625
Sum of electronic and thermal Free Energies= -1306.052535

E (Thermal) KCal/Mol CV Cal/Mol-Kelvin S Cal/Mol-Kelvin
268.729 93.207 145.033

Cartesian coordinates

Atom X Y Z
C -1.244710 -0.497274 -1.312506
C -1.054092 0.920163 -1.287819
C 0.229744 1.412999 -1.252332
C 1.369131 0.542380 -1.208971
C 1.155817 -0.831553 -1.229668
C -0.175291 -1.364856 -1.250827
C 2.388999 1.113132 -0.396825
C 3.277184 0.220659 0.236531
C 3.073678 -1.192933 0.195454
C 1.968838 -1.691154 -0.489869
C 3.876415 -2.182556 1.028346
C 3.180669 -3.601376 1.228614
C 1.738409 -3.745212 0.773253
C 1.177444 -2.883813 -0.090506
C -0.247527 -2.592581 -0.506316
C -1.479944 -2.963732 0.024042
C -2.609342 -2.047955 -0.001680
C -2.430165 -0.789606 -0.618769
C -3.087062 0.425061 -0.227303
C -4.189602 0.287863 0.598973
C -4.554100 -1.013849 1.044793
C -3.782506 -2.144257 0.800815
C 0.520299 2.549309 -0.479037
C -0.517342 3.293602 0.124636
C -1.874137 2.772019 0.044992
C -2.135114 1.550299 -0.568974
C -0.069864 4.296519 1.031734
C 1.275487 4.375052 1.374376
C 2.245927 3.434836 0.927820
C 1.869628 2.473726 0.003995
H 4.032065 0.602602 0.921540
H -4.745045 1.147029 0.964841
H -5.431393 -1.110755 1.678153
H -4.043862 -3.084742 1.278957
H -2.629214 3.251682 0.663989
H -0.784831 4.959080 1.512692
H 1.587084 5.134134 2.086314
H 3.239431 3.451699 1.367741
H -1.567252 -3.847852 0.651574
H 1.177404 -4.573697 1.201995
H 4.010228 -1.756390 2.029796
H 3.174206 -3.782736 2.310754
C 4.034953 -4.767178 0.633987
H 3.589203 -5.723711 0.925935
H 3.971201 -4.719077 -0.462858
C 5.310806 -2.319323 0.418964
H 5.205831 -2.452674 -0.667534
H 5.844267 -1.373776 0.556122
C 6.078764 -3.481054 0.990512
H 7.110301 -3.341900 1.304521
C 5.469668 -4.666383 1.078669
H 5.975918 -5.544267 1.472253

Adduct of the reaction of butadiene with Fragment 1 substituted with one NH_2 group

Molecular formula: C34H19N
Charge = 0 Multiplicity = 1
Hartree/particle
Thermal correction to Energy= 0.446023
Thermal correction to Enthalpy= 0.446968
Thermal correction to Gibbs Free Energy= 0.375802
Sum of electronic and zero-point Energies= -1361.335476
Sum of electronic and thermal Energies= -1361.313607
Sum of electronic and thermal Enthalpies= -1361.312662
Sum of electronic and thermal Free Energies= -1361.383828

E (Thermal) KCal/Mol CV Cal/Mol-Kelvin S Cal/Mol-Kelvin
279.884 98.736 149.781

Cartesian coordinates

Atom X Y Z
C -1.369100 -0.697969 -1.278014
C -1.222485 0.724895 -1.259979
C 0.045812 1.257305 -1.232220
C 1.211579 0.422229 -1.192038
C 1.041252 -0.957494 -1.209026
C -0.272984 -1.531735 -1.218943
C 2.216741 1.025982 -0.384583
C 3.134287 0.163743 0.247899
C 2.976081 -1.255844 0.208225
C 1.885830 -1.789108 -0.472456
C 3.814901 -2.216194 1.036024
C 3.166302 -3.659572 1.267673
C 1.718734 -3.830037 0.819051
C 1.133324 -2.998520 -0.055157
C -0.302640 -2.756183 -0.466426
C -1.519146 -3.162922 0.073610
C -2.677317 -2.283888 0.048494
C -2.541024 -1.023907 -0.576091
C -3.233642 0.171319 -0.186701
C -4.327049 0.003634 0.646038
C -4.648177 -1.306537 1.100192
C -3.842459 -2.413179 0.857875
C 0.304981 2.404066 -0.462972
C -0.752179 3.117726 0.143792
C -2.092592 2.554713 0.071739
C -2.318852 1.323929 -0.537713
C -0.331834 4.135980 1.046683
C 1.011829 4.256459 1.383490
C 2.008539 3.345260 0.934843
C 1.657909 2.371183 0.014554
H 3.877285 0.570498 0.931368
H -4.907067 0.846774 1.011055
H -5.518475 -1.427659 1.738988
H -4.070888 -3.358766 1.342722
H -2.858943 3.012271 0.693589
H -1.064854 4.777094 1.529508
H 1.303003 5.025957 2.092858
H 3.002700 3.393008 1.370919
H -1.574507 -4.044695 0.707988
H 1.178926 -4.660421 1.271587
C 4.019477 -4.758234 0.536056
H 3.593694 -5.742532 0.760655
H 3.920328 -4.616778 -0.550228
C 5.248573 -2.299009 0.410059
H 5.136984 -2.384999 -0.680540
H 5.763590 -1.349690 0.585378
C 6.052949 -3.469496 0.908735
H 7.090018 -3.326251 1.202296
C 5.466129 -4.669893 0.945565
H 5.995723 -5.559121 1.278250
N 3.166917 -3.867420 2.734451
H 2.788024 -4.791649 2.942589
H 4.136088 -3.890097 3.050986
H 3.923231 -1.795359 2.041542

Adduct of the reaction of butadiene with Fragment 1 substituted with one OMe group

Molecular formula: C35H20O
Charge = 0 Multiplicity = 1

Hartree/particle

Thermal correction to Energy= 0.462604
Thermal correction to Enthalpy= 0.463548
Thermal correction to Gibbs Free Energy= 0.389154
Sum of electronic and zero-point Energies= -1420.488800
Sum of electronic and thermal Energies= -1420.465567
Sum of electronic and thermal Enthalpies= -1420.464623
Sum of electronic and thermal Free Energies= -1420.539017

E (Thermal) KCal/Mol CV Cal/Mol-Kelvin S Cal/Mol-Kelvin
290.288 102.758 156.574

Cartesian coordinates

Atom	X	Y	Z
C	-1.387668	-0.694445	-1.281367
C	-1.244520	0.728487	-1.249577
C	0.022699	1.264276	-1.259751
C	1.191153	0.432175	-1.272506
C	1.024479	-0.947402	-1.303160
C	-0.287794	-1.525285	-1.273256
C	2.222436	1.027767	-0.491488
C	3.162197	0.160323	0.098449
C	3.008100	-1.259849	0.043045
C	1.897818	-1.787157	-0.609518
C	3.868530	-2.224738	0.839231
C	3.234571	-3.668055	1.076818
C	1.778927	-3.848780	0.661242
C	1.164291	-3.002836	-0.180134
C	0.286712	-2.758965	-0.535837
C	-1.481094	-3.176742	0.043375
C	-2.642254	-2.301616	0.071758
C	-2.532320	-1.033466	-0.541322
C	-3.213867	0.154207	-0.111680
C	-4.276085	-0.027889	0.757709
C	-4.576679	-1.344760	1.206423
C	-3.776718	-2.445368	0.921226
C	0.306126	2.401269	-0.484311
C	-0.730614	3.103553	0.169213
C	-2.071279	2.537488	0.137328
C	-2.315723	1.314293	-0.480247
C	-0.281085	4.110750	1.070426
C	1.073329	4.230703	1.361237
C	2.055755	3.328538	0.865418
C	1.675047	2.366111	-0.055209
H	3.925399	0.560009	0.763486
H	-4.845166	0.808506	1.154089
H	-5.423114	-1.476978	1.874421
H	-3.984486	-3.397890	1.401800
H	-2.816118	2.984124	0.792375
H	-0.998158	4.743116	1.587522
H	1.387419	4.991486	2.070220
H	3.064625	3.373186	1.266621
H	-1.510435	-4.067146	0.667432
H	1.253363	-4.684334	1.116007
C	4.096927	-4.763417	0.377047
H	3.686696	-5.754656	0.590104
H	3.975556	-4.612743	-0.704809
C	5.291547	-2.297062	0.191592
H	5.165635	-2.395084	-0.896767
H	5.793785	-1.337925	0.349715
C	6.118023	-3.447833	0.698472
H	7.154869	-3.284835	0.980846
C	5.545564	-4.651406	0.768167
H	6.082545	-5.529310	1.117802
O	3.293853	-3.788624	2.518704
C	3.181415	-5.073297	3.099837
H	4.062079	-5.700259	2.905492
H	3.106314	-4.909587	4.177713
H	2.285120	-5.621603	2.776427
H	3.999092	-1.831214	1.852141

Adduct of the reaction of butadiene with Fragment 1 substituted with one OH group

```
Molecular formula: C34H18O
Charge = 0 Multiplicity = 1
                                                   Hartree/particle
Thermal correction to Energy= 0.433062
Thermal correction to Enthalpy= 0.434006
Thermal correction to Gibbs Free Energy= 0.362876
Sum of electronic and zero-point Energies= -1381.218216
Sum of electronic and thermal Energies= -1381.196482
Sum of electronic and thermal Enthalpies= -1381.195538
Sum of electronic and thermal Free Energies= -1381.266668

E (Thermal) KCal/Mol CV Cal/Mol-Kelvin S Cal/Mol-Kelvin
 271.750 97.926 149.705
```

```
 Cartesian coordinates

Atom X Y Z
 C -1.374990 -0.724374 -1.258632
 C -1.217560 0.697203 -1.260792
 C 0.054963 1.220206 -1.247535
 C 1.213966 0.376253 -1.201233
 C 1.033754 -1.001954 -1.200114
 C -0.285152 -1.565868 -1.195367
 C 2.227084 0.983193 -0.405150
 C 3.136095 0.123240 0.240706
 C 2.968047 -1.296837 0.220253
 C 1.875082 -1.831501 -0.455860
 C 3.762321 -2.246563 1.105382
 C 3.175865 -3.724260 1.196842
 C 1.712287 -3.882018 0.824311
 C 1.115620 -3.034513 -0.027764
 C -0.320275 -2.780578 -0.427198
 C -1.535924 -3.170541 0.126606
 C -2.687069 -2.282263 0.097421
 C -2.545052 -1.031934 -0.545028
 C -3.226313 0.173586 -0.168048
 C -4.315444 0.025757 0.674097
 C -4.643292 -1.275422 1.148568
 C -3.847495 -2.391439 0.916331
 C 0.326956 2.375496 -0.495344
 C -0.721526 3.105714 0.106906
 C -2.066495 2.551850 0.050167
 C -2.305229 1.314366 -0.540475
 C -0.288712 4.133425 0.992957
 C 1.057593 4.248652 1.321133
 C 2.044818 3.323426 0.880594
 C 1.681712 2.339144 -0.023890
 H 3.878885 0.535958 0.920602
 H -4.886778 0.878278 1.030907
 H -5.510143 -1.381121 1.794705
 H -4.079612 -3.328472 1.415713
 H -2.825864 3.023842 0.669769
 H -1.014353 4.786897 1.470310
 H 1.358273 5.026156 2.017667
 H 3.041738 3.369892 1.310499
 H -1.593216 -4.043972 0.772068
 H 1.204174 -4.716683 1.299178
 C 4.026865 -4.709393 0.335792
 H 3.597982 -5.711727 0.422362
 H 3.950615 -4.413396 -0.719786
 C 5.272946 -2.230129 0.679612
 H 5.314194 -2.092127 -0.409914
 H 5.755557 -1.346857 1.108861
 C 6.034161 -3.486317 1.016524
 H 7.057342 -3.405411 1.376005
 C 5.459271 -4.675852 0.805485
 H 5.987601 -5.606247 0.997605
 O 3.245471 -4.148035 2.565143
 H 4.188023 -4.247968 2.768974
 H 3.706624 -1.880985 2.137636
```

Adduct of the reaction of butadiene with Fragment 1 substituted with one Me group

```
Molecular formula: C35H20
Charge = 0 Multiplicity = 1
                                                  Hartree/particle
Thermal correction to Energy= 0.457294
Thermal correction to Enthalpy= 0.458238
Thermal correction to Gibbs Free Energy= 0.387461
Sum of electronic and zero-point Energies= -1345.297397
Sum of electronic and thermal Energies= -1345.275484
Sum of electronic and thermal Enthalpies= -1345.274540
Sum of electronic and thermal Free Energies= -1345.345317

E (Thermal) KCal/Mol CV Cal/Mol-Kelvin S Cal/Mol-Kelvin
 286.956 99.065 148.963


 Cartesian coordinates

Atom X Y Z
 C -1.349993 -0.851123 -1.172476
 C -1.156473 0.559099 -1.310141
 C 0.129489 1.046891 -1.360239
 C 1.265952 0.179081 -1.246746
 C 1.050467 -1.187030 -1.115733
 C -0.282407 -1.712742 -1.045079
 C 2.302846 0.827402 -0.516045
 C 3.186079 0.009872 0.213995
 C 2.979502 -1.401104 0.336489
 C 1.874085 -1.964162 -0.298176
 C 3.612654 -2.268962 1.429311
 C 3.243112 -3.794410 1.264144
 C 1.739457 -3.981112 1.027854
 C 1.098593 -3.124363 0.213907
 C -0.338565 -2.854486 -0.170757
 C -1.555332 -3.156578 0.432074
 C -2.682769 -2.240916 0.337854
 C -2.517312 -1.059572 -0.418809
 C -3.161774 0.193413 -0.148467
 C -4.242330 0.154431 0.716909
 C -4.595830 -1.087083 1.315477
 C -3.832296 -2.241209 1.178518
 C 0.441077 2.258979 -0.720818
 C -0.580197 3.070234 -0.178424
 C -1.939697 2.549950 -0.168487
 C -2.217708 1.269522 -0.637687
 C -0.109654 4.163787 0.603503
 C 1.243127 4.271890 0.906577
 C 2.199637 3.282337 0.545398
 C 1.799712 2.228865 -0.260204
 H 3.928727 0.475854 0.857498
 H -4.786829 1.051644 0.998071
 H -5.455597 -1.108976 1.979161
 H -4.081560 -3.121124 1.765916
 H -2.678318 3.098160 0.412276
 H -0.811992 4.878529 1.024617
 H 1.572921 5.102397 1.524338
 H 3.202544 3.340764 0.959676
 H -1.627363 -3.967142 1.153713
 H 1.240666 -4.820844 1.507009
 C 4.014449 -4.385827 0.043550
 H 3.939384 -5.481205 0.073066
 H 3.516105 -4.082869 -0.886087
 C 5.138520 -2.079677 1.588757
 H 5.396970 -1.024534 1.441254
 H 5.428793 -2.296072 2.627274
 C 5.958471 -2.933652 0.659451
 H 7.014978 -2.687420 0.570112
 C 5.460289 -3.970684 -0.017295
 H 6.111126 -4.550509 -0.669600
 C 3.645433 -4.555865 2.542692
 H 3.172528 -4.117990 3.428589
 H 3.327292 -5.602107 2.477385
 H 4.727960 -4.550422 2.691069
 H 3.146389 -1.938483 2.371550
```

Adduct of the reaction of butadiene with Fragment 1 substituted with one *i*-Pr group

Molecular formula: C37H24
Charge = 0 Multiplicity = 1
Hartree/particle
Thermal correction to Energy= 0.516960
Thermal correction to Enthalpy= 0.517904
Thermal correction to Gibbs Free Energy= 0.440875
Sum of electronic and zero-point Energies= -1423.865355
Sum of electronic and thermal Energies= -1423.840614
Sum of electronic and thermal Enthalpies= -1423.839670
Sum of electronic and thermal Free Energies= -1423.916699

E (Thermal) KCal/Mol CV Cal/Mol-Kelvin S Cal/Mol-Kelvin
324.397 109.509 162.121

Cartesian coordinates

Atom X Y Z
C -1.351829 -0.832431 -1.117533
C -1.116698 0.561282 -1.336022
C 0.180614 0.995882 -1.485646
C 1.289826 0.090665 -1.398172
C 1.033941 -1.258217 -1.187364
C -0.309978 -1.727783 -1.010964
C 2.392445 0.730887 -0.762999
C 3.289450 -0.085962 -0.048980
C 3.041397 -1.481153 0.153817
C 1.877954 -2.027912 -0.383870
C 3.710271 -2.316229 1.252652
C 3.271548 -3.839740 1.195048
C 1.748984 -3.968006 1.055712
C 1.095190 -3.128921 0.233367
C -0.352745 -2.822955 -0.078313
C -1.540754 -3.049033 0.609348
C -2.638586 -2.096216 0.536423
C -2.477764 -0.959469 -0.286682
C -3.059157 0.328932 -0.040832
C -4.085946 0.372682 0.887611
C -4.446235 -0.824476 1.566981
C -3.733944 -2.012304 1.442723
C 0.574486 2.223005 -0.925344
C -0.381570 3.097882 -0.363370
C -1.756060 2.632203 -0.248136
C -2.108412 1.343214 -0.637208
C 0.175371 4.207006 0.335427
C 1.547215 4.275973 0.551936
C 2.443371 3.234242 0.182752
C 1.956938 2.161095 -0.545591
H 4.086794 0.380557 0.524963
H -4.580199 1.302417 1.155648
H -5.264214 -0.781700 2.280607
H -3.977957 -2.852948 2.087096
H -2.437684 3.235070 0.347976
H -0.473336 4.967460 0.762482
H 1.944062 5.120585 1.108065
H 3.471272 3.272580 0.533228
H -1.597869 -3.820887 1.373565
H 1.232809 -4.753676 1.599809
C 3.929121 -4.462300 -0.079772
H 3.766017 -5.544007 -0.096932
H 3.403402 -4.081664 -0.964157
C 5.249856 -2.175403 1.304677
H 5.531254 -1.142122 1.069646
H 5.600452 -2.331257 2.335667
C 5.982997 -3.116833 0.387747
H 7.047315 -2.937279 0.246905
C 5.395757 -4.151493 -0.217415
H 5.981118 -4.798860 -0.868166
C 3.526092 -6.105973 2.466370
H 4.067071 -6.600301 1.656088
H 3.879323 -6.547084 3.404178
H 2.466297 -6.366730 2.369863
C 3.759527 -4.583097 2.493184
H 4.844784 -4.443451 2.545996
C 3.151945 -4.005214 3.785653
H 2.059086 -4.084635 3.789329
H 3.523945 -4.558132 4.654444
H 3.405236 -2.953232 3.941600
H 3.319457 -1.898459 2.190455

Adduct of the reaction of butadiene with Fragment 1 substituted with one F group

```
Molecular formula: C34H17F
Charge = 0 Multiplicity = 1
                                                  Hartree/particle
Thermal correction to Energy= 0.420378
Thermal correction to Enthalpy= 0.421322
Thermal correction to Gibbs Free Energy= 0.350582
Sum of electronic and zero-point Energies= -1405.249395
Sum of electronic and thermal Energies= -1405.227912
Sum of electronic and thermal Enthalpies= -1405.226967
Sum of electronic and thermal Free Energies= -1405.297707

E (Thermal) KCal/Mol CV Cal/Mol-Kelvin S Cal/Mol-Kelvin
 263.791 96.439 148.885
```

Cartesian coordinates

```
Atom X Y Z
 C -1.376242 -0.674744 -1.291200
 C -1.228434 0.747716 -1.253223
 C 0.040438 1.278637 -1.221674
 C 1.205019 0.441769 -1.195723
 C 1.033815 -0.936872 -1.232716
 C -0.280968 -1.510182 -1.248199
 C 2.213014 1.033586 -0.381799
 C 3.131612 0.162845 0.235865
 C 2.973190 -1.256299 0.174098
 C 1.880517 -1.779529 -0.511137
 C 3.805423 -2.229187 0.991446
 C 3.146621 -3.655122 1.203105
 C 1.709345 -3.837587 0.758425
 C 1.126060 -2.992728 -0.105842
 C -0.309790 -2.745050 -0.513682
 C -1.523480 -3.158253 0.027339
 C -2.681131 -2.278198 0.018499
 C -2.545769 -1.009850 -0.589062
 C -3.235915 0.180254 -0.180567
 C -4.325989 0.001558 0.654359
 C -4.646395 -1.314372 1.091945
 C -3.842583 -2.418184 0.831269
 C 0.303093 2.414259 -0.437147
 C -0.751729 3.120261 0.182522
 C -2.092656 2.559123 0.107035
 C -2.321814 1.337122 -0.518880
 C -0.327783 4.126044 1.097636
 C 1.016991 4.241436 1.431791
 C 2.011652 3.335592 0.968138
 C 1.657241 2.374435 0.035816
 H 3.877386 0.559076 0.922331
 H -4.904018 0.839913 1.033202
 H -5.514031 -1.443428 1.732695
 H -4.069400 -3.370178 1.304009
 H -2.856734 3.008554 0.737481
 H -1.058799 4.761275 1.591069
 H 1.310843 5.001426 2.150146
 H 3.007297 3.377076 1.401362
 H -1.576296 -4.049179 0.648889
 H 1.190705 -4.675698 1.216409
 C 4.027495 -4.800400 0.650669
 H 3.591406 -5.750223 0.973602
 H 3.963260 -4.776658 -0.446658
 C 5.242586 -2.330032 0.386329
 H 5.146155 -2.474465 -0.699700
 H 5.745297 -1.367305 0.518270
 C 6.040765 -3.460633 0.979237
 H 7.065719 -3.286836 1.296131
 C 5.457114 -4.654291 1.102413
 H 5.973738 -5.509042 1.529369
 F 3.119958 -3.854757 2.607742
 H 3.912510 -1.843422 2.010958
```

Adduct of the reaction of butadiene with Fragment 1 substituted with one COOH group

```
Molecular formula: C35H18O2
Charge = 0 Multiplicity = 1
                                                  Hartree/particle
Thermal correction to Energy= 0.445853
Thermal correction to Enthalpy= 0.446797
Thermal correction to Gibbs Free Energy= 0.371182
Sum of electronic and zero-point Energies= -1494.560790
Sum of electronic and thermal Energies= -1494.537323
Sum of electronic and thermal Enthalpies= -1494.536379
Sum of electronic and thermal Free Energies= -1494.611995

E (Thermal) KCal/Mol CV Cal/Mol-Kelvin S Cal/Mol-Kelvin
 279.777 103.464 159.147


 Cartesian coordinates

Atom X Y Z
 C -1.310686 -0.887495 -1.094530
 C -1.123848 0.523121 -1.238443
 C 0.159066 1.012764 -1.331793
 C 1.299629 0.146438 -1.256610
 C 1.090146 -1.219188 -1.116393
 C -0.238568 -1.747590 -1.002985
 C 2.360523 0.796293 -0.561875
 C 3.271615 -0.019228 0.134734
 C 3.070143 -1.430519 0.265471
 C 1.941998 -1.994041 -0.326282
 C 3.745568 -2.288354 1.339201
 C 3.364526 -3.808790 1.176859
 C 1.847620 -4.015354 1.001818
 C 1.184875 -3.153577 0.212335
 C -0.264643 -2.889471 -0.128097
 C -1.459098 -3.193250 0.516529
 C -2.590605 -2.279568 0.459276
 C -2.451833 -1.097854 -0.302111
 C -3.088399 0.154177 -0.009991
 C -4.139091 0.113315 0.891374
 C -4.470831 -1.128748 1.501093
 C -3.710990 -2.281909 1.338265
 C 0.490816 2.224845 -0.702473
 C -0.512586 3.033986 -0.124709
 C -1.870314 2.512042 -0.069386
 C -2.162622 1.231687 -0.530148
 C -0.017184 4.127292 0.642055
 C 1.344934 4.237361 0.899007
 C 2.290140 3.249919 0.504066
 C 1.864444 2.197002 -0.288890
 H 4.037376 0.446950 0.750158
 H -4.675036 1.009669 1.190998
 H -5.307775 -1.151817 2.193204
 H -3.939155 -3.162366 1.933245
 H -2.589624 3.058895 0.536302
 H -0.705809 4.840434 1.087739
 H 1.694195 5.067576 1.506276
 H 3.306500 3.309450 0.883858
 H -1.505510 -4.003862 1.240062
 H 1.380533 -4.871184 1.480216
 C 4.097605 -4.428919 -0.049962
 H 4.000264 -5.517072 0.009321
 H 3.576206 -4.117401 -0.962636
 C 5.278084 -2.108041 1.444584
 H 5.536923 -1.061324 1.246155
 H 5.589278 -2.288436 2.481978
 C 6.070053 -3.000865 0.527241
 H 7.131481 -2.780418 0.430380
 C 5.547467 -4.036300 -0.132502
 H 6.181103 -4.640785 -0.778487
 C 3.749808 -4.626045 2.420041
 O 3.886715 -5.829668 2.430422
 O 3.850536 -3.890599 3.554332
 H 4.047305 -4.527510 4.262625
 H 3.316953 -1.955068 2.293269
```

Adduct of the reaction of butadiene with Fragment 1 substituted with one CF_3 group

Molecular formula: C35H17F3
Charge = 0 Multiplicity = 1
Hartree/particle
Thermal correction to Energy= 0.436399
Thermal correction to Enthalpy= 0.437344
Thermal correction to Gibbs Free Energy= 0.361208
Sum of electronic and zero-point Energies= -1643.038655
Sum of electronic and thermal Energies= -1643.014623
Sum of electronic and thermal Enthalpies= -1643.013679
Sum of electronic and thermal Free Energies= -1643.089815

E (Thermal) KCal/Mol CV Cal/Mol-Kelvin S Cal/Mol-Kelvin
273.845 105.985 160.242

Cartesian coordinates

Atom X Y Z
C -1.323607 -0.879721 -1.143387
C -1.131126 0.530617 -1.283656
C 0.154357 1.017065 -1.356158
C 1.291053 0.147343 -1.263647
C 1.076973 -1.218353 -1.131796
C -0.255218 -1.742820 -1.036950
C 2.340379 0.792601 -0.547213
C 3.235090 -0.026425 0.165580
C 3.030412 -1.438230 0.285133
C 1.915632 -1.998127 -0.332136
C 3.708071 -2.300878 1.356113
C 3.305452 -3.824227 1.201547
C 1.790133 -4.007570 1.008069
C 1.146520 -3.152096 0.198771
C -0.296955 -2.883540 -0.161380
C -1.500913 -3.184201 0.466671
C -2.629432 -2.268344 0.392233
C -2.476757 -1.087401 -0.367685
C -3.114977 0.165975 -0.085354
C -4.178777 0.127856 0.800549
C -4.522040 -1.113096 1.406121
C -3.762329 -2.267859 1.255082
C 0.478904 2.227244 -0.719225
C -0.531565 3.039035 -0.157800
C -1.891257 2.520686 -0.125149
C -2.179150 1.241314 -0.591307
C -0.045936 4.130150 0.618249
C 1.311857 4.235573 0.899029
C 2.260212 3.244965 0.520208
C 1.844586 2.194449 -0.281226
H 3.987552 0.437894 0.798270
H -4.716961 1.025559 1.091956
H -5.368762 -1.133847 2.086267
H -4.000572 -3.147299 1.847539
H -2.618859 3.068799 0.469351
H -0.739854 4.845198 1.052464
H 1.653275 5.064180 1.512855
H 3.269948 3.300473 0.917821
H -1.558909 -3.993609 1.190693
H 1.300948 -4.838857 1.503710
C 4.056558 -4.472037 -0.005860
H 4.044865 -5.561984 0.114435
H 3.471097 -4.273456 -0.910858
C 5.241698 -2.106591 1.413567
H 5.471343 -1.044756 1.273593
H 5.609590 -2.353207 2.417058
C 5.989157 -2.923140 0.395694
H 7.016794 -2.633040 0.187417
C 5.464245 -3.983443 -0.218218
H 6.057960 -4.540999 -0.940042
C 3.666627 -4.583736 2.489477
F 3.090066 -4.018605 3.576690
F 3.227733 -5.867451 2.438153
F 4.992439 -4.647923 2.732408
H 3.295262 -1.966746 2.318010

Adduct of the reaction of butadiene with Fragment 1 substituted with one CHO group

Molecular formula: C35H18O
Charge = 0 Multiplicity = 1
Hartree/particle
Thermal correction to Energy= 0.438953
Thermal correction to Enthalpy= 0.439898
Thermal correction to Gibbs Free Energy= 0.366550
Sum of electronic and zero-point Energies= -1419.313119
Sum of electronic and thermal Energies= -1419.290449
Sum of electronic and thermal Enthalpies= -1419.289505
Sum of electronic and thermal Free Energies= -1419.362852

E (Thermal) KCal/Mol CV Cal/Mol-Kelvin S Cal/Mol-Kelvin
275.447 100.286 154.372

Cartesian coordinates

Atom X Y Z
C -1.324291 -0.877365 -1.160579
C -1.134012 0.534697 -1.283545
C 0.150568 1.025928 -1.335101
C 1.288748 0.158837 -1.235573
C 1.077191 -1.209383 -1.120849
C -0.254804 -1.738583 -1.048509
C 2.327116 0.801666 -0.501640
C 3.214605 -0.021546 0.216338
C 3.011398 -1.434011 0.319893
C 1.906946 -1.995497 -0.318171
C 3.655137 -2.307284 1.403746
C 3.275159 -3.833183 1.197369
C 1.773014 -4.022823 0.997699
C 1.134308 -3.161511 0.188198
C -0.304721 -2.888760 -0.185667
C -1.516820 -3.199641 0.421724
C -2.646380 -2.285597 0.342269
C -2.487272 -1.096174 -0.403073
C -3.132974 0.152412 -0.116346
C -4.208401 0.102141 0.754724
C -4.556071 -1.146206 1.342440
C -3.790996 -2.297182 1.189514
C 0.462840 2.231448 -0.683570
C -0.557543 3.034612 -0.127580
C -1.915842 2.511484 -0.117079
C -2.193524 1.235565 -0.598546
C -0.085633 4.120231 0.664396
C 1.268217 4.227929 0.962945
C 2.224898 3.244311 0.586813
C 1.823180 2.199400 -0.229004
H 3.957182 0.439063 0.863670
H -4.752998 0.995240 1.048410
H -5.411734 -1.176571 2.010968
H -4.034463 -3.183423 1.769582
H -2.652827 3.051821 0.472989
H -0.787536 4.828693 1.096608
H 1.598926 5.052111 1.588525
H 3.229458 3.300087 0.997396
H -1.581982 -4.017254 1.135761
H 1.289294 -4.844897 1.514721
C 4.059839 -4.412648 -0.024782
H 3.998405 -5.509397 -0.003221
H 3.544443 -4.111008 -0.944313
C 5.181293 -2.119354 1.556410
H 5.432133 -1.060538 1.426308
H 5.482660 -2.352445 2.588570
C 5.996376 -2.950061 0.602699
H 7.049246 -2.693376 0.504777
C 5.499447 -3.977702 -0.089438
H 6.147366 -4.538754 -0.760204
C 3.727961 -4.597479 2.440365
H 4.826305 -4.731889 2.531931
O 2.980116 -5.052045 3.279016
H 3.187675 -1.998741 2.351517

Adduct of the reaction of butadiene with Fragment 1 substituted with one CN group

Molecular formula: C35H17N
Charge = 0 Multiplicity = 1
Hartree/particle
Thermal correction to Energy= 0.428143
Thermal correction to Enthalpy= 0.429087
Thermal correction to Gibbs Free Energy= 0.355951
Sum of electronic and zero-point Energies= -1398.239555
Sum of electronic and thermal Energies= -1398.217054
Sum of electronic and thermal Enthalpies= -1398.216110
Sum of electronic and thermal Free Energies= -1398.289246

E (Thermal) KCal/Mol CV Cal/Mol-Kelvin S Cal/Mol-Kelvin
268.664 99.698 153.928

Cartesian coordinates

Atom X Y Z
C -1.344559 -0.693373 -1.271416
C -1.199118 0.729530 -1.233821
C 0.068496 1.263260 -1.204066
C 1.234575 0.428619 -1.177262
C 1.065303 -0.950471 -1.211122
C -0.248210 -1.527055 -1.228785
C 2.242198 1.023812 -0.365926
C 3.166482 0.155657 0.247936
C 3.007530 -1.262845 0.189555
C 1.913361 -1.790425 -0.488953
C 3.869809 -2.229853 0.982368
C 3.188980 -3.670071 1.241026
C 1.737503 -3.853527 0.778000
C 1.161450 -3.005389 -0.086267
C -0.275279 -2.762260 -0.495611
C -1.487950 -3.177178 0.046650
C -2.646876 -2.299033 0.037859
C -2.513592 -1.030393 -0.569532
C -3.205276 0.158702 -0.160788
C -4.294160 -0.021772 0.675216
C -4.612266 -1.338221 1.113094
C -3.807358 -2.440952 0.851751
C 0.329675 2.400011 -0.420816
C -0.726176 3.104021 0.199287
C -2.065916 2.540078 0.125289
C -2.293300 1.317134 -0.499691
C -0.303536 4.111092 1.113629
C 1.041215 4.229582 1.446808
C 2.037407 3.325610 0.982982
C 1.684153 2.363570 0.051112
H 3.917080 0.553476 0.928187
H -4.872976 0.815611 1.054896
H -5.478790 -1.468339 1.755029
H -4.032195 -3.393097 1.324961
H -2.830451 2.988597 0.755754
H -1.035444 4.744911 1.607455
H 1.333884 4.990222 2.164842
H 3.033102 3.369202 1.415777
H -1.539413 -4.067497 0.669098
H 1.204802 -4.691378 1.219537
C 4.057035 -4.823504 0.606608
H 3.638156 -5.787169 0.907977
H 3.927947 -4.745197 -0.481135
C 5.260514 -2.371586 0.279739
H 5.082714 -2.538002 -0.792445
H 5.779730 -1.411818 0.354997
C 6.086288 -3.499073 0.836144
H 7.124580 -3.326378 1.105707
C 5.507059 -4.692890 0.984184
H 6.037982 -5.550658 1.386063
C 3.180214 -3.889371 2.703112
N 3.163971 -4.076935 3.849391
H 4.064782 -1.796927 1.968357

Adduct of the reaction of butadiene with Fragment 1 substituted with one NO_2 group

```
Molecular formula: C34H17NO2
Charge = 0 Multiplicity = 1
                                                    Hartree/particle
Thermal correction to Energy= 0.433107
Thermal correction to Enthalpy= 0.434051
Thermal correction to Gibbs Free Energy= 0.359606
Sum of electronic and zero-point Energies= -1510.504663
Sum of electronic and thermal Energies= -1510.481532
Sum of electronic and thermal Enthalpies= -1510.480588
Sum of electronic and thermal Free Energies= -1510.555033

E (Thermal) KCal/Mol CV Cal/Mol-Kelvin S Cal/Mol-Kelvin
 271.779 102.044 156.684
```

```
 Cartesian coordinates

Atom X Y Z
 C -1.231308 -0.657132 -1.213003
 C -0.994926 0.747936 -1.340131
 C 0.305130 1.198143 -1.374029
 C 1.413334 0.296353 -1.252615
 C 1.156432 -1.062567 -1.132136
 C -0.191701 -1.550880 -1.080858
 C 2.460835 0.909638 -0.505168
 C 3.314676 0.063786 0.225974
 C 3.065360 -1.341754 0.334711
 C 1.949254 -1.867443 -0.311490
 C 3.675996 -2.222017 1.430796
 C 3.234152 -3.710946 1.235560
 C 1.729844 -3.881851 1.010797
 C 1.131941 -3.002729 0.190057
 C -0.291830 -2.694556 -0.214281
 C -1.520877 -2.963555 0.378451
 C -2.620274 -2.015402 0.276490
 C -2.411743 -0.835593 -0.471734
 C -3.021808 0.434116 -0.201146
 C -4.111654 0.421723 0.653337
 C -4.507775 -0.811877 1.241479
 C -3.777183 -1.987180 1.106310
 C 0.645860 2.396388 -0.723203
 C -0.357093 3.233799 -0.186415
 C -1.731139 2.753631 -0.194037
 C -2.041821 1.484846 -0.674470
 C 0.136953 4.307943 0.607828
 C 1.488832 4.374644 0.926477
 C 2.419660 3.359568 0.569612
 C 1.997499 2.324268 -0.248024
 H 4.064649 0.502498 0.879500
 H -4.632736 1.332773 0.934063
 H -5.374217 -0.811588 1.896557
 H -4.057439 -2.862355 1.686462
 H -2.459552 3.319870 0.382122
 H -0.548586 5.040370 1.026009
 H 1.835726 5.190960 1.553447
 H 3.418979 3.385469 0.995445
 H -1.622740 -3.775034 1.095185
 H 1.234279 -4.726340 1.477318
 C 4.022627 -4.407855 0.100796
 H 3.860626 -5.485974 0.186506
 H 3.563009 -4.102310 -0.846898
 C 5.209842 -2.090151 1.592686
 H 5.508730 -1.060737 1.364349
 H 5.469736 -2.245220 2.646617
 C 6.012723 -3.040076 0.747807
 H 7.085825 -2.864647 0.707900
 C 5.489868 -4.077669 0.093105
 H 6.131507 -4.730867 -0.493944
 O 3.827116 -3.866413 3.560827
 O 3.378477 -5.708881 2.503675
 N 3.524727 -4.491499 2.550144
 H 3.223184 -1.889495 2.373457
```

Adduct of the reaction of butadiene with Fragment 2

Molecular formula: C34H16
Charge = 0 Multiplicity = 1
Hartree/particle
Thermal correction to Energy= 0.403845
Thermal correction to Enthalpy= 0.404789
Thermal correction to Gibbs Free Energy= 0.337919
Sum of electronic and zero-point Energies= -1304.751498
Sum of electronic and thermal Energies= -1304.731604
Sum of electronic and thermal Enthalpies= -1304.730660
Sum of electronic and thermal Free Energies= -1304.797531

E (Thermal) KCal/Mol CV Cal/Mol-Kelvin S Cal/Mol-Kelvin
253.417 90.804 140.742

Cartesian coordinates

Atom X Y Z
C -0.514143 -0.064126 1.765768
C 0.312284 -1.234814 1.733002
C 1.680884 -0.810425 1.674338
C 1.699991 0.623533 1.674095
C 0.343111 1.084150 1.732516
C -0.082139 -2.313138 0.972734
C 0.861633 -3.127007 0.224131
C 2.197322 -2.715802 0.175120
C 2.566086 -1.493690 0.869983
C 3.609487 -0.820033 0.123120
C 3.628031 0.581172 0.122749
C 2.602948 1.282641 0.869334
C 2.266847 2.513859 0.173980
C 0.942565 2.960493 0.222862
C -0.022530 2.172276 0.971794
C -1.313169 2.258863 0.325468
C -2.149157 1.144812 0.361759
C -1.698021 -0.048572 1.035929
C -1.374766 -2.365785 0.326670
C -2.180946 -1.229963 0.362584
C 0.030552 -3.905699 -0.706959
C -1.262056 -3.466633 -0.648514
C 3.251515 -2.908360 -0.831721
C 4.067990 -1.812276 -0.862284
C 4.112627 1.560364 -0.863212
C 3.325651 2.677840 -0.833067
C 0.132558 3.760418 -0.708791
C -1.171245 3.355855 -0.650268
C -3.330268 0.766460 -0.520700
C -3.351749 -0.820738 -0.519843
H 0.404850 -4.657644 -1.390940
H 3.320452 -3.756237 -1.502494
H 4.881329 -1.660039 -1.561603
H 4.921510 1.386237 -1.562595
H 3.416929 3.523274 -1.504256
H 0.526764 4.501785 -1.393143
H -1.967175 3.732414 -1.281826
H -3.186514 -1.169978 -1.544900
H -2.067795 -3.822349 -1.279711
H -3.155014 1.109933 -1.546020
C -4.710153 -1.411861 -0.047787
H -4.748524 -2.478259 -0.291056
C -4.672182 1.394883 -0.050125
H -4.681511 2.461512 -0.295246
C -5.873240 -0.660969 -0.647022
H -6.711858 -1.217174 -1.058334
C -5.855099 0.674766 -0.648252
H -6.678382 1.252747 -1.060517
H -4.722424 1.334163 1.046895
H -4.758415 -1.347835 1.049124

Adduct of the reaction of butadiene with Fragment 2 substituted with one NH_2 group

Molecular formula: C34H17N
Charge = 0 Multiplicity = 1
Hartree/particle
Thermal correction to Energy= 0.421883
Thermal correction to Enthalpy= 0.422827
Thermal correction to Gibbs Free Energy= 0.353785
Sum of electronic and zero-point Energies= -1360.084606
Sum of electronic and thermal Energies= -1360.063472
Sum of electronic and thermal Enthalpies= -1360.062528
Sum of electronic and thermal Free Energies= -1360.131570

E (Thermal) KCal/Mol CV Cal/Mol-Kelvin S Cal/Mol-Kelvin
264.736 96.290 145.312

Cartesian coordinates

Atom X Y Z
C 1.445527 -0.303058 2.628292
C 0.729780 0.938001 2.602460
C -0.672853 0.641725 2.563353
C -0.824610 -0.783867 2.570294
C 0.485850 -1.366815 2.609466
C 1.212068 1.975094 1.835881
C 0.338377 2.870232 1.095796
C -1.030085 2.583100 1.063578
C -1.501823 1.402164 1.767903
C -2.611075 0.824807 1.036231
C -2.758882 -0.569126 1.041980
C -1.793708 -1.359767 1.779324
C -1.582481 -2.621220 1.087589
C -0.304545 -3.187121 1.119848
C 0.739851 -2.483668 1.847171
C 2.008146 -2.693408 1.182910
C 2.947019 -1.663837 1.213503
C 2.614914 -0.430492 1.886195
C 2.496971 1.907445 1.176826
C 3.197391 0.702965 1.208776
C 1.227267 3.567007 0.153047
C 2.475335 3.012088 0.199772
C -2.074246 2.868901 0.068523
C -2.988037 1.852044 0.051998
C -3.345251 -1.503648 0.068345
C -2.665768 -2.689931 0.095715
C 0.417146 -4.072427 0.192286
C 1.753429 -3.790720 0.226589
C 4.177872 -1.412189 0.343211
C 4.319338 0.189440 0.324074
H 0.916591 4.349046 -0.529113
H -2.073639 3.717747 -0.604555
H -3.820265 1.773236 -0.637185
H -4.143259 -1.258595 -0.622234
H -2.844539 -3.526157 -0.569391
H -0.053113 -4.783104 -0.476543
H 2.510437 -4.221240 -0.414122
H 3.303712 3.292127 -0.440307
C 5.720955 0.705396 0.757897
H 5.845496 1.742704 0.431391
C 5.445180 -2.063640 0.970720
H 5.370683 -3.146735 0.846345
C 6.829510 -0.177673 0.240390
H 7.717305 0.276482 -0.192706
C 6.702312 -1.503733 0.350058
H 7.480887 -2.181347 0.009134
H 5.443537 -1.861203 2.050641
H 5.760824 0.727017 1.856933
N 4.003913 -2.011339 -0.986143
H 3.211346 -1.581822 -1.459084
H 4.828547 -1.810112 -1.549001
H 4.159545 0.509972 -0.712394

Adduct of the reaction of butadiene with Fragment 2 substituted with one OMe group

Molecular formula: C35H18O
Charge = 0 Multiplicity = 1
Hartree/particle
Thermal correction to Energy= 0.438373
Thermal correction to Enthalpy= 0.439317
Thermal correction to Gibbs Free Energy= 0.366714
Sum of electronic and zero-point Energies= -1419.229731
Sum of electronic and thermal Energies= -1419.207184
Sum of electronic and thermal Enthalpies= -1419.206240
Sum of electronic and thermal Free Energies= -1419.278843

E (Thermal) KCal/Mol CV Cal/Mol-Kelvin S Cal/Mol-Kelvin
275.083 100.368 152.805

Cartesian coordinates

Atom X Y Z
C 1.426438 -0.360263 2.648095
C 0.686780 0.865500 2.691228
C -0.709249 0.545122 2.615449
C -0.831886 -0.880721 2.527761
C 0.489456 -1.439111 2.547031
C 1.157462 1.956897 1.995874
C 0.273847 2.880327 1.302823
C -1.088396 2.570979 1.238166
C -1.544588 1.338949 1.860565
C -2.633865 0.788399 1.079013
C -2.752682 -0.605372 0.992257
C -1.779703 -1.422429 1.688467
C -1.533280 -2.630330 0.918251
C -0.243658 -3.169578 0.931169
C 0.777586 -2.495520 1.714761
C 2.064677 -2.645694 1.067134
C 2.989626 -1.610153 1.191152
C 2.608186 -0.421178 1.918835
C 2.447491 1.951083 1.344079
C 3.168046 0.758960 1.308245
C 1.156895 3.647558 0.411108
C 2.413026 3.110206 0.433127
C -2.126587 2.899491 0.250225
C -3.020265 1.869015 0.158017
C -3.307674 -1.486038 -0.048099
C -2.603825 -2.657437 -0.089422
C 0.506762 -3.981593 -0.037251
C 1.836955 -3.683233 0.040183
C 4.273498 -1.270825 0.417619
C 4.297719 0.321741 0.392363
H 0.837298 4.462806 -0.226501
H -2.134235 3.788748 -0.368405
H -3.842364 1.818359 -0.545765
H -4.102113 -1.212347 -0.731975
H -2.756685 -3.451588 -0.810159
H 0.061424 -4.655934 -0.758571
H 2.595345 -4.091975 -0.612674
H 3.239929 3.438559 -0.185401
C 5.664414 0.968049 0.767125
H 5.741202 1.948702 0.286626
C 5.516177 -1.819140 1.169550
H 5.530458 -2.911445 1.129710
C 6.851727 0.103101 0.429870
H 7.756201 0.574753 0.053974
C 6.793587 -1.212795 0.644427
H 7.644975 -1.859030 0.446559
H 5.384519 -1.561693 2.229345
H 5.672338 1.166265 1.848951
O 4.201442 -1.686338 -0.952337
C 4.927146 -2.817135 -1.396583
H 4.645549 -2.953785 -2.443764
H 6.012577 -2.664954 -1.346172
H 4.679282 -3.738875 -0.852457
H 4.065520 0.586539 -0.643666

Adduct of the reaction of butadiene with Fragment 2 substituted with one OH group

Molecular formula: C34H16O
Charge = 0 Multiplicity = 1
Hartree/particle
Thermal correction to Energy= 0.408832
Thermal correction to Enthalpy= 0.409776
Thermal correction to Gibbs Free Energy= 0.340948
Sum of electronic and zero-point Energies= -1379.962860
Sum of electronic and thermal Energies= -1379.941862
Sum of electronic and thermal Enthalpies= -1379.940918
Sum of electronic and thermal Free Energies= -1380.009746

E (Thermal) KCal/Mol CV Cal/Mol-Kelvin S Cal/Mol-Kelvin
256.546 95.523 144.861

Cartesian coordinates

Atom X Y Z
C 1.426377 -0.318583 2.643866
C 0.699759 0.915877 2.634129
C -0.699878 0.607360 2.577833
C -0.838334 -0.819543 2.554320
C 0.476979 -1.391659 2.594035
C 1.179247 1.971858 1.891136
C 0.302002 2.872999 1.162058
C -1.063512 2.575255 1.115092
C -1.529987 1.376419 1.792064
C -2.627812 0.804083 1.039492
C -2.761542 -0.590853 1.013992
C -1.794650 -1.387650 1.742246
C -1.565164 -2.631378 1.024386
C -0.282253 -3.185287 1.055702
C 0.747429 -2.490303 1.810791
C 2.024791 -2.676778 1.157562
C 2.953228 -1.643163 1.226679
C 2.602667 -0.421451 1.909635
C 2.466403 1.924487 1.233309
C 3.174548 0.724581 1.244873
C 1.188321 3.591831 0.233876
C 2.439251 3.043785 0.273699
C -2.102108 2.871741 0.117392
C -3.006674 1.847676 0.073438
C -3.330319 -1.509242 0.014961
C -2.639041 -2.688803 0.021751
C 0.457263 -4.038721 0.111919
C 1.789968 -3.746124 0.167494
C 4.198998 -1.364408 0.401007
C 4.277293 0.226112 0.322997
H 0.873571 4.382088 -0.436793
H -2.103229 3.733509 -0.539025
H -3.832316 1.775060 -0.624264
H -4.124351 -1.256472 -0.677380
H -2.802930 -3.511388 -0.663821
H -0.000166 -4.730473 -0.584929
H 2.553243 -4.150376 -0.483015
H 3.266617 3.337891 -0.361277
C 5.683103 0.807055 0.644322
H 5.772762 1.810769 0.217574
C 5.471554 -1.954214 1.068217
H 5.413943 -3.045105 1.018729
C 6.795010 -0.096329 0.168204
H 7.664917 0.340474 -0.316207
C 6.706068 -1.413408 0.385871
H 7.498107 -2.094308 0.084654
H 5.477443 -1.680457 2.132553
H 5.771167 0.932753 1.733331
O 4.044802 -1.935523 -0.896694
H 4.892237 -1.815553 -1.350682
H 4.030216 0.477703 -0.714407

Adduct of the reaction of butadiene with Fragment 2 substituted with one Me group

```
Molecular formula: C35H18
Charge = 0 Multiplicity = 1
                                                Hartree/particle
Thermal correction to Energy= 0.432974
Thermal correction to Enthalpy= 0.433918
Thermal correction to Gibbs Free Energy= 0.364303
Sum of electronic and zero-point Energies= -1344.037591
Sum of electronic and thermal Energies= -1344.016271
Sum of electronic and thermal Enthalpies= -1344.015327
Sum of electronic and thermal Free Energies= -1344.084942

E (Thermal) KCal/Mol CV Cal/Mol-Kelvin S Cal/Mol-Kelvin
 271.695 96.834 146.517
```

```
 Cartesian coordinates

Atom X Y Z
 C 1.415542 -0.265370 2.625777
 C 0.685198 0.967913 2.597534
 C -0.713513 0.653380 2.572863
 C -0.847190 -0.773955 2.599222
 C 0.470387 -1.339002 2.632238
 C 1.147736 2.001719 1.815839
 C 0.258665 2.877101 1.071013
 C -1.105775 2.569287 1.049182
 C -1.556839 1.391219 1.771543
 C -2.664315 0.789374 1.055813
 C -2.796614 -0.605777 1.085119
 C -1.817094 -1.373293 1.827669
 C -1.597385 -2.644121 1.157692
 C -0.312867 -3.196498 1.191035
 C 0.733169 -2.463253 1.884842
 C 1.997388 -2.668135 1.207976
 C 2.916200 -1.614840 1.189268
 C 2.576022 -0.386482 1.869754
 C 2.432970 1.948887 1.156995
 C 3.148295 0.753833 1.197322
 C 1.136034 3.580742 0.122360
 C 2.393576 3.047537 0.173196
 C -2.160422 2.827274 0.057547
 C -3.060938 1.798342 0.060610
 C -3.380117 -1.564352 0.132898
 C -2.688651 -2.743314 0.176907
 C 0.406254 -4.104877 0.286934
 C 1.739179 -3.806980 0.299972
 C 4.119502 -1.329252 0.275429
 C 4.303697 0.257265 0.351438
 H 0.812440 4.351611 -0.566434
 H -2.175383 3.666443 -0.627412
 H -3.896237 1.699367 -0.622200
 H -4.185626 -1.340405 -0.556152
 H -2.863926 -3.593172 -0.471631
 H -0.059338 -4.853306 -0.342658
 H 2.483918 -4.301108 -0.310355
 H 3.216340 3.335847 -0.470326
 C 5.683401 0.697525 0.919799
 H 5.826768 1.766411 0.733158
 C 5.389667 -2.074224 0.798961
 H 5.319825 -3.134478 0.534724
 C 6.804479 -0.126884 0.339196
 H 7.706983 0.367158 -0.011748
 C 6.662520 -1.453703 0.278583
 H 7.444666 -2.092225 -0.124154
 H 5.390147 -2.033123 1.897527
 H 5.667459 0.578395 2.013170
 C 3.863738 -1.724322 -1.191970
 H 3.723130 -2.802003 -1.303529
 H 2.971695 -1.226336 -1.584331
 H 4.718894 -1.433521 -1.810035
 H 4.233682 0.659379 -0.664967
```

Adduct of the reaction of butadiene with Fragment 2 substituted with one *i*-Pr group

```
Molecular formula: C37H22
Charge = 0 Multiplicity = 1
                                                   Hartree/particle
Thermal correction to Energy= 0.493025
Thermal correction to Enthalpy= 0.493969
Thermal correction to Gibbs Free Energy= 0.419785
Sum of electronic and zero-point Energies= -1422.598654
Sum of electronic and thermal Energies= -1422.574883
Sum of electronic and thermal Enthalpies= -1422.573939
Sum of electronic and thermal Free Energies= -1422.648123

E (Thermal) KCal/Mol CV Cal/Mol-Kelvin S Cal/Mol-Kelvin
 309.378 106.828 156.134
```

```
 Cartesian coordinates

Atom X Y Z
 C 1.571278 -0.583392 2.622508
 C 0.926179 0.674783 2.840720
 C -0.491249 0.458929 2.798994
 C -0.720748 -0.936477 2.559057
 C 0.555342 -1.579605 2.449110
 C 1.447738 1.805853 2.251917
 C 0.602404 2.858087 1.709360
 C -0.779996 2.649169 1.677451
 C -1.298388 1.387574 2.180156
 C -2.456317 1.004798 1.396097
 C -2.680637 -0.358531 1.161471
 C -1.740739 -1.313102 1.713485
 C -1.614255 -2.437877 0.804232
 C -0.365109 -3.057969 0.692224
 C 0.735207 -2.543524 1.486841
 C 1.989051 -2.720315 0.773127
 C 3.002454 -1.779874 0.976157
 C 2.710487 -0.643781 1.833266
 C 2.700734 1.782871 1.532897
 C 3.323971 0.550916 1.322506
 C 1.495497 3.657233 0.857831
 C 2.707197 3.034423 0.751725
 C -1.830387 3.157265 0.783134
 C -2.799732 2.206809 0.620533
 C -3.341455 -1.076267 0.059328
 C -2.726381 -2.280185 -0.145266
 C 0.280629 -3.819375 -0.383270
 C 1.633604 -3.638816 -0.334492
 C 4.352122 -1.451527 0.294964
 C 4.284233 0.139964 0.208917
 H 1.207778 4.558976 0.331128
 H -1.798481 4.109924 0.268334
 H -3.651275 2.292514 -0.043783
 H -4.142617 -0.674302 -0.549180
 H -2.966658 -2.975886 -0.940194
 H -0.242094 -4.384221 -1.145663
 H 2.324773 -4.052367 -1.054232
 H 3.524499 3.373955 0.126718
 C 5.633185 0.905081 0.181342
 H 5.880110 1.189777 -0.849894
 C 5.460065 -1.864829 1.322606
 H 5.670183 -2.933397 1.228875
 C 6.805605 0.168488 0.762865
 H 7.756072 0.699111 0.764387
 C 6.737594 -1.072922 1.242927
 H 7.630309 -1.541190 1.653745
 H 5.056677 -1.747631 2.337126
 H 5.508973 1.855037 0.717662
 C 4.922655 -3.612509 -1.031009
 H 4.738590 -4.100243 -1.994558
 H 5.990599 -3.726472 -0.816392
 H 4.372330 -4.164526 -0.264977
 C 4.528502 -2.123819 -1.109390
 H 3.539251 -2.064147 -1.581970
 C 5.509462 -1.419989 -2.065479
 H 6.518425 -1.374287 -1.646701
 H 5.563806 -1.977750 -3.006743
 H 5.194213 -0.403678 -2.314925
 H 3.760388 0.373831 -0.728288
```

Adduct of the reaction of butadiene with Fragment 2 substituted with one F group

Molecular formula: C34H15F
Charge = 0 Multiplicity = 1
Hartree/particle
Thermal correction to Energy= 0.396110
Thermal correction to Enthalpy= 0.397055
Thermal correction to Gibbs Free Energy= 0.328384
Sum of electronic and zero-point Energies= -1403.994056
Sum of electronic and thermal Energies= -1403.973274
Sum of electronic and thermal Enthalpies= -1403.972330
Sum of electronic and thermal Free Energies= -1404.041001

E (Thermal) KCal/Mol CV Cal/Mol-Kelvin S Cal/Mol-Kelvin
248.563 94.023 144.530

Cartesian coordinates

Atom	X	Y	Z
C	1.406596	-0.280580	2.664810
C	0.675774	0.951257	2.628903
C	-0.722802	0.637150	2.576548
C	-0.856265	-0.790586	2.578935
C	0.461189	-1.357570	2.631321
C	1.152688	1.992926	1.864673
C	0.273779	2.877614	1.117768
C	-1.090789	2.575622	1.075935
C	-1.553891	1.388253	1.774937
C	-2.649392	0.798714	1.032045
C	-2.778370	-0.596620	1.032419
C	-1.809521	-1.376497	1.776105
C	-1.574205	-2.631488	1.079093
C	-0.289714	-3.179842	1.121930
C	0.735838	-2.472061	1.870542
C	2.013339	-2.661351	1.220433
C	2.937199	-1.623066	1.274710
C	2.585762	-0.391799	1.935602
C	2.440995	1.937450	1.208306
C	3.153932	0.742350	1.245998
C	1.158992	3.581739	0.177276
C	2.411544	3.038729	0.228123
C	-2.129822	2.850447	0.072467
C	-3.031226	1.823082	0.046832
C	-3.342367	-1.534598	0.048818
C	-2.646362	-2.710842	0.076257
C	0.453779	-4.037119	0.184039
C	1.784448	-3.735868	0.235556
C	4.151570	-1.345467	0.405313
C	4.270190	0.230483	0.347556
H	0.842326	4.358185	-0.508386
H	-2.133190	3.699935	-0.599702
H	-3.856360	1.735014	-0.649614
H	-4.136912	-1.297024	-0.648264
H	-2.806209	-3.546026	-0.594798
H	-0.000599	-4.736219	-0.507380
H	2.546812	-4.144987	-0.414070
H	3.238730	3.322939	-0.411398
C	5.676942	0.762757	0.733713
H	5.774827	1.800264	0.399616
C	5.438588	-2.034770	0.893391
H	5.346862	-3.106387	0.694058
C	6.774104	-0.106297	0.169023
H	7.642506	0.363678	-0.285097
C	6.660719	-1.434396	0.242332
H	7.429031	-2.094346	-0.150434
H	5.497519	-1.922134	1.985260
H	5.754881	0.789361	1.830579
F	3.918364	-1.822974	-0.898807
H	4.078267	0.497758	-0.696705

Adduct of the reaction of butadiene with Fragment 2 substituted with one COOH group

Molecular formula: C35H16O2
Charge = 0 Multiplicity = 1
Hartree/particle
Thermal correction to Energy= 0.421402
Thermal correction to Enthalpy= 0.422346
Thermal correction to Gibbs Free Energy= 0.349082
Sum of electronic and zero-point Energies= -1493.303727
Sum of electronic and thermal Energies= -1493.280956
Sum of electronic and thermal Enthalpies= -1493.280012
Sum of electronic and thermal Free Energies= -1493.353276

E (Thermal) KCal/Mol CV Cal/Mol-Kelvin S Cal/Mol-Kelvin
264.434 101.242 154.199

Cartesian coordinates

Atom X Y Z
C 1.428098 -0.266968 2.622311
C 0.693662 0.963108 2.579877
C -0.704032 0.643268 2.551389
C -0.832878 -0.784362 2.585138
C 0.486501 -1.345392 2.629144
C 1.156140 1.992717 1.792184
C 0.266311 2.861011 1.039655
C -1.096873 2.549587 1.015925
C -1.546582 1.373859 1.742976
C -2.648868 0.764711 1.025837
C -2.775344 -0.630688 1.060580
C -1.796179 -1.391024 1.810732
C -1.567895 -2.663256 1.144801
C -0.281890 -3.210431 1.186246
C 0.754929 -2.475416 1.891420
C 2.021682 -2.677010 1.221290
C 2.933515 -1.622032 1.212236
C 2.592382 -0.386346 1.871516
C 2.441288 1.937394 1.131885
C 3.158846 0.744959 1.181659
C 1.142464 3.559595 0.086367
C 2.400194 3.028140 0.139869
C -2.148794 2.799249 0.019341
C -3.045691 1.767326 0.024329
C -3.350130 -1.594567 0.108446
C -2.653388 -2.769930 0.158640
C 0.447500 -4.104850 0.274321
C 1.779300 -3.802144 0.294093
C 4.144168 -1.349053 0.303377
C 4.305567 0.244144 0.321829
H 0.817278 4.324754 -0.607949
H -2.164134 3.635095 -0.669605
H -3.878388 1.662059 -0.660674
H -4.153859 -1.376186 -0.584437
H -2.821973 -3.622867 -0.487573
H -0.013640 -4.843234 -0.370329
H 2.533234 -4.260403 -0.333020
H 3.222145 3.311739 -0.506589
C 5.697170 0.696592 0.848496
H 5.827541 1.762103 0.635699
C 5.421325 -2.074631 0.815321
H 5.344436 -3.136800 0.578383
C 6.804889 -0.133943 0.250888
H 7.686538 0.357771 -0.151690
C 6.670140 -1.462419 0.231922
H 7.432143 -2.111183 -0.190037
H 5.438636 -1.984989 1.909761
H 5.708844 0.599655 1.943772
O 4.362964 -2.796365 -1.657378
O 2.899926 -1.095692 -1.733217
C 3.849533 -1.838544 -1.119397
H 2.753499 -1.507945 -2.601194
H 4.203997 0.621158 -0.698406

Adduct of the reaction of butadiene with Fragment 2 substituted with one CF_3 group

Molecular formula: C35H15F3
Charge = 0 Multiplicity = 1
Hartree/particle
Thermal correction to Energy= 0.412011
Thermal correction to Enthalpy= 0.412955
Thermal correction to Gibbs Free Energy= 0.338274
Sum of electronic and zero-point Energies= -1641.778771
Sum of electronic and thermal Energies= -1641.755355
Sum of electronic and thermal Enthalpies= -1641.754411
Sum of electronic and thermal Free Energies= -1641.829091

E (Thermal) KCal/Mol CV Cal/Mol-Kelvin S Cal/Mol-Kelvin
258.541 103.759 157.178

Cartesian coordinates

Atom X Y Z
C 1.421767 -0.264588 2.553580
C 0.689283 0.967314 2.538963
C -0.709138 0.650870 2.560058
C -0.840091 -0.775782 2.602236
C 0.480041 -1.338473 2.594995
C 1.125885 1.995818 1.736787
C 0.214723 2.864786 1.012310
C -1.149009 2.554350 1.032746
C -1.576674 1.381216 1.777918
C -2.703575 0.772210 1.100153
C -2.833172 -0.622995 1.145257
C -1.830551 -1.382083 1.864423
C -1.629373 -2.659772 1.199990
C -0.344016 -3.208598 1.197847
C 0.722707 -2.465382 1.847637
C 1.966860 -2.678018 1.132781
C 2.886890 -1.625647 1.091076
C 2.558831 -0.389452 1.764974
C 2.392135 1.941116 1.042732
C 3.110032 0.748571 1.075883
C 1.064323 3.563404 0.034960
C 2.324512 3.034537 0.054653
C -2.233044 2.802918 0.070992
C -3.131023 1.772522 0.108943
C -3.444322 -1.591857 0.221144
C -2.751026 -2.770080 0.255553
C 0.348547 -4.128316 0.285714
C 1.680315 -3.832617 0.251452
C 4.114508 -1.332050 0.199957
C 4.265608 0.260663 0.228806
H 0.719936 4.327945 -0.650699
H -2.269228 3.635971 -0.620508
H -3.985312 1.666237 -0.548747
H -4.270040 -1.375260 -0.445927
H -2.944734 -3.625799 -0.379742
H -0.135341 -4.882558 -0.322695
H 2.401844 -4.316939 -0.388028
H 3.127722 3.320045 -0.614167
C 5.633917 0.736161 0.804009
H 5.763477 1.796827 0.568208
C 5.386778 -2.050185 0.756397
H 5.336183 -3.112729 0.508926
C 6.789065 -0.090491 0.302523
H 7.697362 0.406904 -0.026782
C 6.670144 -1.418957 0.276485
H 7.473485 -2.059339 -0.076619
H 5.332615 -1.986519 1.851111
H 5.587359 0.673369 1.900954
C 3.900790 -1.748841 -1.263265
F 3.959398 -3.094453 -1.438660
F 2.704885 -1.334879 -1.729452
F 4.844514 -1.231614 -2.082553
H 4.191867 0.643433 -0.791928

Adduct of the reaction of butadiene with Fragment 2 substituted with one CHO group

Molecular formula: C35H16O
Charge = 0 Multiplicity = 1
Hartree/particle
Thermal correction to Energy= 0.414639
Thermal correction to Enthalpy= 0.415584
Thermal correction to Gibbs Free Energy= 0.343974
Sum of electronic and zero-point Energies= -1418.057272
Sum of electronic and thermal Energies= -1418.035275
Sum of electronic and thermal Enthalpies= -1418.034331
Sum of electronic and thermal Free Energies= -1418.105941

E (Thermal) KCal/Mol CV Cal/Mol-Kelvin S Cal/Mol-Kelvin
260.190 97.944 150.716

Cartesian coordinates

Atom X Y Z
C 1.434229 -0.251972 2.631832
C 0.695621 0.976062 2.595536
C -0.700354 0.651515 2.547708
C -0.824369 -0.777061 2.562540
C 0.496506 -1.333981 2.613648
C 1.162660 2.017148 1.825630
C 0.278209 2.891347 1.074117
C -1.083446 2.575438 1.031429
C -1.536633 1.389209 1.739196
C -2.629003 0.785446 1.002780
C -2.750631 -0.610608 1.018369
C -1.776706 -1.376996 1.769463
C -1.536815 -2.639610 1.089485
C -0.249386 -3.182790 1.137612
C 0.777127 -2.453810 1.864407
C 2.050695 -2.639556 1.202518
C 2.963062 -1.583422 1.221014
C 2.606711 -0.357688 1.892365
C 2.455685 1.975077 1.179239
C 3.177690 0.784895 1.224503
C 1.162490 3.604977 0.139243
C 2.422091 3.078771 0.200968
C -2.125807 2.834274 0.027076
C -3.018904 1.799326 0.009717
C -3.311709 -1.564112 0.047814
C -2.611027 -2.737339 0.090145
C 0.492219 -4.060106 0.219095
C 1.822187 -3.751264 0.255403
C 4.162869 -1.292645 0.306050
C 4.338516 0.294590 0.376603
H 0.842535 4.378527 -0.548159
H -2.137059 3.678836 -0.651176
H -3.843999 1.699879 -0.685222
H -4.108446 -1.339502 -0.651043
H -2.768795 -3.582105 -0.569289
H 0.040486 -4.788476 -0.443140
H 2.582116 -4.195330 -0.374405
H 3.251114 3.376400 -0.430021
C 5.725230 0.714186 0.937262
H 5.862680 1.788597 0.780576
C 5.440678 -2.073192 0.713401
H 5.354007 -3.098992 0.348586
C 6.832629 -0.089549 0.302785
H 7.724485 0.420231 -0.051967
C 6.691448 -1.412327 0.189270
H 7.461349 -2.029830 -0.264695
H 5.479473 -2.121571 1.809815
H 5.724379 0.560547 2.025995
O 4.294549 -2.502505 -1.807792
C 3.775864 -1.641005 -1.134508
H 2.940497 -1.026536 -1.537298
H 4.267827 0.707769 -0.637580

Adduct of the reaction of butadiene with Fragment 2 substituted with one CN group

```
Molecular formula: C35H15N
Charge = 0 Multiplicity = 1
                                                  Hartree/particle
Thermal correction to Energy= 0.403830
Thermal correction to Enthalpy= 0.404774
Thermal correction to Gibbs Free Energy= 0.333950
Sum of electronic and zero-point Energies= -1396.986376
Sum of electronic and thermal Energies= -1396.964620
Sum of electronic and thermal Enthalpies= -1396.963675
Sum of electronic and thermal Free Energies= -1397.034500

E (Thermal) KCal/Mol CV Cal/Mol-Kelvin S Cal/Mol-Kelvin
 253.407 97.266 149.062
```

```
 Cartesian coordinates

Atom X Y Z
 C 1.430462 -0.287821 2.607471
 C 0.700589 0.945163 2.598476
 C -0.698844 0.632069 2.571246
 C -0.833566 -0.795276 2.569213
 C 0.484544 -1.362626 2.589644
 C 1.162590 1.992936 1.834770
 C 0.272161 2.883369 1.109749
 C -1.092348 2.578884 1.086967
 C -1.543062 1.387066 1.787542
 C -2.652164 0.801485 1.061798
 C -2.783713 -0.593739 1.060187
 C -1.802727 -1.377091 1.783753
 C -1.583501 -2.632145 1.081719
 C -0.299910 -3.184262 1.100124
 C 0.743280 -2.472711 1.819584
 C 2.006104 -2.662942 1.137503
 C 2.926575 -1.618067 1.167045
 C 2.592485 -0.393561 1.851114
 C 2.442536 1.947560 1.163325
 C 3.156365 0.752120 1.182714
 C 1.144803 3.599472 0.166133
 C 2.400431 3.061696 0.197628
 C -2.149301 2.858898 0.104016
 C -3.050627 1.831151 0.088676
 C -3.368312 -1.529855 0.086633
 C -2.675628 -2.708404 0.100343
 C 0.421299 -4.054715 0.158750
 C 1.754002 -3.759249 0.179221
 C 4.158561 -1.329073 0.294553
 C 4.293058 0.272876 0.297232
 H 0.818282 4.381726 -0.508135
 H -2.164613 3.711535 -0.563896
 H -3.887340 1.746533 -0.594195
 H -4.174565 -1.289732 -0.595908
 H -2.850147 -3.542078 -0.568798
 H -0.046469 -4.765765 -0.510981
 H 2.496191 -4.193669 -0.477078
 H 3.218715 3.357186 -0.448112
 C 5.688423 0.759559 0.783397
 H 5.808340 1.812898 0.513345
 C 5.444144 -2.017205 0.872840
 H 5.382149 -3.095115 0.703990
 C 6.807582 -0.088115 0.234201
 H 7.686807 0.393531 -0.184675
 C 6.694330 -1.417667 0.282074
 H 7.471077 -2.074287 -0.098510
 H 5.424113 -1.866404 1.960587
 H 5.706046 0.722772 1.882166
 C 3.974859 -1.792800 -1.089559
 N 3.844422 -2.165563 -2.182045
 H 4.156939 0.628559 -0.728239
```

Adduct of the reaction of butadiene with Fragment 2 substituted with one NO_2 group

Molecular formula: C34H15NO2
Charge = 0 Multiplicity = 1
Hartree/particle
Thermal correction to Energy= 0.408692
Thermal correction to Enthalpy= 0.409636
Thermal correction to Gibbs Free Energy= 0.336815
Sum of electronic and zero-point Energies= -1509.245304
Sum of electronic and thermal Energies= -1509.222822
Sum of electronic and thermal Enthalpies= -1509.221878
Sum of electronic and thermal Free Energies= -1509.294699

E (Thermal) KCal/Mol CV Cal/Mol-Kelvin S Cal/Mol-Kelvin
256.458 99.758 153.265

Cartesian coordinates

Atom	X	Y	Z
C	1.422412	-0.278881	2.625581
C	0.688051	0.951286	2.605542
C	-0.709744	0.631448	2.564977
C	-0.838253	-0.796535	2.566922
C	0.482167	-1.357817	2.601871
C	1.153674	1.997555	1.842074
C	0.266483	2.881107	1.104390
C	-1.096233	2.570033	1.068517
C	-1.548722	1.378898	1.768757
C	-2.647389	0.785035	1.033388
C	-2.772878	-0.610689	1.036694
C	-1.796357	-1.386183	1.774096
C	-1.564509	-2.644080	1.080498
C	-0.279391	-3.191383	1.116023
C	0.753490	-2.472050	1.841975
C	2.023766	-2.661393	1.173866
C	2.935642	-1.607581	1.202996
C	2.589478	-0.381468	1.877382
C	2.440465	1.955154	1.182284
C	3.157438	0.762561	1.210682
C	1.145112	3.597768	0.167072
C	2.402446	3.065323	0.212285
C	-2.143813	2.840770	0.073041
C	-3.040274	1.808866	0.052125
C	-3.343035	-1.554160	0.061495
C	-2.646088	-2.729920	0.088386
C	0.454878	-4.064291	0.186900
C	1.786475	-3.765342	0.220275
C	4.160594	-1.307517	0.338962
C	4.296473	0.274347	0.331711
H	0.821354	4.375007	-0.514250
H	-2.155585	3.690259	-0.598932
H	-3.868924	1.717452	-0.639612
H	-4.142771	-1.320647	-0.630937
H	-2.810137	-3.567543	-0.578436
H	-0.003387	-4.782030	-0.482345
H	2.539698	-4.204015	-0.420965
H	3.224854	3.359885	-0.428487
C	5.696540	0.751500	0.807708
H	5.809124	1.811650	0.562328
C	5.438928	-2.023088	0.817822
H	5.356232	-3.090389	0.613625
C	6.797375	-0.082421	0.199851
H	7.667227	0.408950	-0.227427
C	6.673061	-1.411898	0.200905
H	7.428940	-2.062904	-0.227311
H	5.466104	-1.904932	1.909770
H	5.741513	0.685067	1.904456
N	3.851062	-1.768373	-1.114446
O	4.403477	-2.781398	-1.533998
O	3.033851	-1.108500	-1.744387
H	4.153317	0.617081	-0.694970

Adduct of the reaction of butadiene with Fragment 1 substituted with two NH_2 groups

Molecular formula: C34H20N2
Charge = 0 Multiplicity = 1
Hartree/particle
Thermal correction to Energy= 0.464215
Thermal correction to Enthalpy= 0.465159
Thermal correction to Gibbs Free Energy= 0.392126
Sum of electronic and zero-point Energies= -1416.665882
Sum of electronic and thermal Energies= -1416.642886
Sum of electronic and thermal Enthalpies= -1416.641941
Sum of electronic and thermal Free Energies= -1416.714974

E (Thermal) KCal/Mol CV Cal/Mol-Kelvin S Cal/Mol-Kelvin
291.299 104.091 153.711

Cartesian coordinates

Atom X Y Z
C -1.339367 -0.701972 -1.274459
C -1.200297 0.721406 -1.262924
C 0.065509 1.258802 -1.218500
C 1.233597 0.428616 -1.154723
C 1.072470 -0.951534 -1.168576
C -0.239744 -1.529478 -1.194295
C 2.225033 1.040989 -0.337186
C 3.137613 0.190289 0.314826
C 2.989889 -1.231720 0.276972
C 1.912623 -1.777531 -0.416896
C 3.870267 -2.176623 1.097160
C 3.174856 -3.665829 1.323256
C 1.727809 -3.813882 0.876443
C 1.155743 -2.985363 -0.008365
C -0.275737 -2.749779 -0.436660
C -1.497064 -3.159935 0.089176
C -2.659090 -2.286591 0.043240
C -2.519527 -1.029779 -0.586885
C -3.223151 0.164465 -0.214224
C -4.327228 -0.003612 0.604374
C -4.648497 -1.312379 1.062265
C -3.834398 -2.416652 0.837528
C 0.308808 2.411170 -0.452325
C -0.760309 3.124103 0.134240
C -2.096907 2.554556 0.046178
C -2.309357 1.319498 -0.559853
C -0.357675 4.149481 1.037290
C 0.980329 4.276771 1.393473
C 1.987375 3.366923 0.965279
C 1.654704 2.386420 0.045225
H 3.863651 0.583537 1.019180
H -4.916119 0.839032 0.956157
H -5.526874 -1.433734 1.689899
H -4.065055 -3.360475 1.324851
H -2.874048 3.011917 0.654719
H -1.100402 4.790515 1.505206
H 1.257849 5.051298 2.102911
H 2.974342 3.419248 1.416599
H -1.557234 -4.039143 0.726823
H 1.170373 -4.631309 1.332761
C 4.007714 -4.762338 0.563516
H 3.587379 -5.745625 0.801903
H 3.872229 -4.619643 -0.517978
C 5.215366 -2.337835 0.303230
H 4.971451 -2.503040 -0.754098
H 5.741332 -1.381351 0.359689
C 6.056088 -3.481320 0.804107
H 7.105353 -3.326277 1.043032
C 5.468520 -4.677656 0.915797
H 6.005731 -5.558852 1.257664
N 4.215985 -1.512051 2.356563
H 3.452702 -1.646833 3.016331
H 5.021619 -1.973141 2.774700
N 3.170370 -3.928178 2.778426
H 2.651804 -4.786464 2.956836
H 4.124725 -4.109381 3.085971

Adduct of the reaction of butadiene with Fragment 1 substituted with two OMe group

Molecular formula: C36H22O2
Charge = 0 Multiplicity = 1

Hartree/particle

Thermal correction to Energy= 0.496968
Thermal correction to Enthalpy= 0.497913
Thermal correction to Gibbs Free Energy= 0.418842
Sum of electronic and zero-point Energies= -1534.964934
Sum of electronic and thermal Energies= -1534.939206
Sum of electronic and thermal Enthalpies= -1534.938262
Sum of electronic and thermal Free Energies= -1535.017332

E (Thermal) KCal/Mol CV Cal/Mol-Kelvin S Cal/Mol-Kelvin
311.852 112.341 166.417

Cartesian coordinates

Atom	X	Y	Z
C	-1.364813	-0.736475	-1.254560
C	-1.218789	0.685672	-1.277415
C	0.050562	1.217060	-1.277921
C	1.215343	0.381353	-1.228389
C	1.048331	-0.997968	-1.215058
C	-0.267446	-1.568061	-1.188000
C	2.228828	1.004689	-0.444504
C	3.144814	0.161355	0.208393
C	2.992651	-1.260486	0.193558
C	1.905016	-1.816970	-0.471917
C	3.861365	-2.177993	1.055673
C	3.236944	-3.692158	1.188151
C	1.760958	-3.836702	0.847640
C	1.153806	-3.012566	-0.018500
C	-0.288053	-2.770069	-0.400723
C	-1.495715	-3.162603	0.167779
C	-2.654760	-2.284966	0.133917
C	-2.527113	-1.043292	-0.527832
C	-3.215745	0.161896	-0.163478
C	-4.298287	0.017463	0.687696
C	-4.612365	-1.279200	1.183284
C	-3.808731	-2.391806	0.962090
C	0.318987	2.382925	-0.540408
C	-0.731041	3.113761	0.058618
C	-2.072298	2.550251	0.016277
C	-2.306214	1.304291	-0.558122
C	-0.300702	4.154107	0.930948
C	1.046276	4.280688	1.251820
C	2.036963	3.356541	0.817027
C	1.675840	2.360492	-0.075152
H	3.872017	0.574195	0.900465
H	-4.874147	0.870500	1.035910
H	-5.474157	-1.382537	1.836573
H	-4.029679	-3.323336	1.476724
H	-2.830941	3.023879	0.635582
H	-1.028336	4.808048	1.404639
H	1.345293	5.067009	1.939147
H	3.034982	3.411811	1.243020
H	-1.541298	-4.026950	0.826476
H	1.226550	-4.636556	1.352798
C	4.048727	-4.666191	0.272729
H	3.629945	-5.673583	0.346143
H	3.877400	-4.345070	-0.763804
C	5.273011	-2.233591	0.362446
H	5.093563	-2.224364	-0.720269
H	5.787809	-1.294902	0.583913
C	6.102757	-3.442295	0.695749
H	7.149546	-3.325080	0.963640
C	5.514251	-4.637117	0.611009
H	6.045281	-5.563673	0.812565
O	3.915524	-1.533658	2.321984
O	3.397614	-4.021002	2.575145
C	5.016942	-1.759497	3.191986
H	4.762706	-1.241997	4.120606
H	5.168946	-2.819643	3.400014
H	5.948483	-1.324848	2.803052
C	3.330624	-5.381528	2.956145
H	4.202952	-5.956224	2.616944
H	3.316661	-5.386301	4.048729
H	2.421519	-5.886841	2.601487

Adduct of the reaction of butadiene with Fragment 1 substituted with two OH group

Molecular formula: C34H18O2
Charge = 0 Multiplicity = 1
Hartree/particle
Thermal correction to Energy= 0.438395
Thermal correction to Enthalpy= 0.439339
Thermal correction to Gibbs Free Energy= 0.366811
Sum of electronic and zero-point Energies= -1456.432357
Sum of electronic and thermal Energies= -1456.409608
Sum of electronic and thermal Enthalpies= -1456.408664
Sum of electronic and thermal Free Energies= -1456.481192

E (Thermal) KCal/Mol CV Cal/Mol-Kelvin S Cal/Mol-Kelvin
275.097 102.363 152.648

Cartesian coordinates

Atom X Y Z
C -1.348980 -0.694954 -1.277519
C -1.201247 0.727380 -1.251800
C 0.067887 1.257529 -1.214797
C 1.231560 0.420106 -1.172050
C 1.062226 -0.959236 -1.203278
C -0.253734 -1.529816 -1.218956
C 2.233887 1.019633 -0.356164
C 3.147840 0.156284 0.275580
C 2.993404 -1.263040 0.213875
C 1.906874 -1.799174 -0.472570
C 3.851896 -2.218062 1.037005
C 3.163491 -3.692810 1.245341
C 1.715024 -3.839042 0.823950
C 1.144796 -3.005045 -0.058539
C -0.288530 -2.756742 -0.472222
C -1.506165 -3.165300 0.063507
C -2.663709 -2.285589 0.037826
C -2.523736 -1.023235 -0.581013
C -3.216989 0.170781 -0.189247
C -4.313502 0.000292 0.638873
C -4.637280 -1.311386 1.086728
C -3.831513 -2.417619 0.842886
C 0.324839 2.400088 -0.438249
C -0.734464 3.112779 0.166069
C -2.074960 2.551623 0.084921
C -2.300081 1.324146 -0.531704
C -0.317330 4.126375 1.075700
C 1.024725 4.243160 1.420216
C 2.022399 3.332422 0.972868
C 1.674826 2.363542 0.045991
H 3.878141 0.542909 0.980600
H -4.894329 0.842350 1.005065
H -5.509932 -1.434329 1.721893
H -4.062133 -3.365059 1.322950
H -2.843721 3.006959 0.705428
H -1.051908 4.766619 1.557267
H 1.313316 5.009018 2.134513
H 3.014328 3.375633 1.414197
H -1.563370 -4.050482 0.692867
H 1.178915 -4.648963 1.311524
C 4.006894 -4.794276 0.531976
H 3.567034 -5.765912 0.773618
H 3.909164 -4.656572 -0.553591
C 5.244511 -2.353720 0.351665
H 5.089463 -2.481968 -0.727874
H 5.762326 -1.400393 0.486151
C 6.043731 -3.511062 0.888141
H 7.074525 -3.359155 1.196537
C 5.451133 -4.707717 0.953531
H 5.967056 -5.591960 1.318171
O 4.138292 -1.660501 2.317380
H 3.406438 -1.923121 2.897144
O 3.147473 -3.974482 2.650941
H 4.077670 -4.012890 2.925879

Adduct of the reaction of butadiene with Fragment 1 substituted with two Me group

Molecular formula: C36H22
Charge = 0 Multiplicity = 1
Hartree/particle
Thermal correction to Energy= 0.486508
Thermal correction to Enthalpy= 0.487452
Thermal correction to Gibbs Free Energy= 0.414303
Sum of electronic and zero-point Energies= -1384.581305
Sum of electronic and thermal Energies= -1384.557986
Sum of electronic and thermal Enthalpies= -1384.557042
Sum of electronic and thermal Free Energies= -1384.630191

E (Thermal) KCal/Mol CV Cal/Mol-Kelvin S Cal/Mol-Kelvin
305.288 105.148 153.955

Cartesian coordinates

Atom X Y Z
C -1.338688 -0.872218 -1.158337
C -1.140184 0.535161 -1.315373
C 0.147378 1.018903 -1.361909
C 1.279719 0.149883 -1.222716
C 1.059687 -1.212510 -1.070175
C -0.274912 -1.734701 -1.007892
C 2.312912 0.806883 -0.495543
C 3.185984 -0.001238 0.256598
C 2.971865 -1.409503 0.409900
C 1.871099 -1.978164 -0.229151
C 3.591765 -2.256739 1.544605
C 3.241627 -3.797336 1.303280
C 1.735960 -3.997643 1.082744
C 1.090255 -3.137860 0.275830
C -0.343196 -2.865887 -0.120537
C -1.566371 -3.155422 0.475219
C -2.689610 -2.236512 0.359046
C -2.513624 -1.066445 -0.412545
C -3.156409 0.192319 -0.166127
C -4.244604 0.169009 0.690328
C -4.607079 -1.062613 1.303625
C -3.846136 -2.221061 1.189775
C 0.457351 2.240468 -0.740300
C -0.566477 3.062729 -0.219619
C -1.927389 2.545682 -0.212210
C -2.205182 1.258636 -0.663286
C -0.099549 4.168162 0.547671
C 1.251150 4.278456 0.859169
C 2.208337 3.280987 0.522155
C 1.812496 2.214807 -0.268607
H 3.928752 0.476055 0.891252
H -4.788850 1.071758 0.953696
H -5.472557 -1.072314 1.960143
H -4.103370 -3.091654 1.787551
H -2.669219 3.105110 0.353581
H -0.803442 4.891333 0.951431
H 1.578066 5.118537 1.465419
H 3.208136 3.344598 0.943151
H -1.648170 -3.956757 1.206079
H 1.244224 -4.840551 1.563360
C 3.982567 -4.300469 0.020175
H 3.918917 -5.396347 -0.009728
H 3.451060 -3.953727 -0.874065
C 5.128165 -2.077038 1.643516
H 5.371562 -1.010917 1.557292
H 5.457307 -2.353677 2.656290
C 5.926973 -2.859963 0.639303
H 6.975121 -2.589091 0.525632
C 5.419348 -3.867977 -0.071495
H 6.050768 -4.402852 -0.778972
C 2.962451 -1.737584 2.863931
H 3.187066 -0.674464 2.991953
H 1.874950 -1.850260 2.857458
H 3.359222 -2.264002 3.736554
C 3.691288 -4.668655 2.494611
H 3.178132 -4.398533 3.421182
H 3.458422 -5.718773 2.287468
H 4.768945 -4.600326 2.662892

Adduct of the reaction of butadiene with Fragment 1 substituted with two *i*-Pr group

Molecular formula: C40H30
Charge = 0 Multiplicity = 1
Hartree/particle
Thermal correction to Energy= 0.605927
Thermal correction to Enthalpy= 0.606871
Thermal correction to Gibbs Free Energy= 0.523284
Sum of electronic and zero-point Energies= -1541.701974
Sum of electronic and thermal Energies= -1541.673540
Sum of electronic and thermal Enthalpies= -1541.672596
Sum of electronic and thermal Free Energies= -1541.756183

E (Thermal) KCal/Mol CV Cal/Mol-Kelvin S Cal/Mol-Kelvin
380.225 125.646 175.925

Cartesian coordinates
Atom X Y Z
C -1.358883 -0.831449 -1.074314
C -1.106228 0.547651 -1.356259
C 0.196295 0.962430 -1.511778
C 1.294013 0.053583 -1.355224
C 1.022036 -1.276177 -1.066929
C -0.328654 -1.730226 -0.905022
C 2.400257 0.714635 -0.756574
C 3.287853 -0.073218 0.005348
C 3.005026 -1.441845 0.328303
C 1.836847 -2.000578 -0.194963
C 3.683749 -2.235111 1.471933
C 3.214025 -3.795979 1.363357
C 1.690640 -3.936725 1.233514
C 1.038817 -3.094063 0.414436
C -0.400457 -2.786576 0.069838
C -1.603401 -2.968398 0.744687
C -2.687187 -2.004551 0.612355
C -2.500019 -0.907792 -0.258123
C -3.069024 0.397568 -0.081014
C -4.108765 0.495250 0.828944
C -4.493781 -0.665566 1.556005
C -3.794864 -1.866500 1.496503
C 0.598148 2.214440 -1.017722
C -0.355987 3.124593 -0.510779
C -1.736105 2.676943 -0.384737
C -2.098575 1.373012 -0.711370
C 0.204556 4.264818 0.133309
C 1.575370 4.334653 0.355838
C 2.467058 3.268721 0.048571
C 1.978611 2.161255 -0.625226
H 4.119313 0.412819 0.504529
H -4.595746 1.441739 1.046988
H -5.321827 -0.580889 2.254117
H -4.059302 -2.674069 2.174288
H -2.417577 3.316717 0.171774
H -0.440981 5.051509 0.515262
H 1.974741 5.204276 0.870071
H 3.492581 3.319393 0.404517
H -1.684910 -3.707098 1.538847
H 1.183471 -4.739286 1.759792
C 3.776265 -4.356279 0.005227
H 3.549317 -5.423685 -0.059111
H 3.220980 -3.900989 -0.820800
C 5.237886 -2.173796 1.355654
H 5.531033 -1.151948 1.091984
H 5.684694 -2.338499 2.347419
C 5.886518 -3.116054 0.382898
H 6.946714 -2.957365 0.192484
C 5.238555 -4.113436 -0.215984
H 5.758955 -4.762496 -0.918231
C 4.002105 -0.226488 3.180579
H 3.816107 0.029800 4.229035
H 5.085374 -0.247963 3.045035
H 3.594126 0.590646 2.579514
C 4.180418 -6.095573 2.231498
H 4.993465 -6.135807 1.504128
H 4.527784 -6.601465 3.138736
H 3.337149 -6.678818 1.842873
C 3.306638 -1.571871 2.873887
H 3.689833 -2.271660 3.624109
C 3.767065 -4.651874 2.583281
H 4.681544 -4.154120 2.922070
C 2.825152 -4.747145 3.805678
H 2.007085 -5.453508 3.626355
H 3.385848 -5.130787 4.664722
H 2.379270 -3.797884 4.102182
C 1.804162 -1.366215 3.139623
H 1.367602 -0.670015 2.417157
H 1.225247 -2.288030 3.096578
H 1.667691 -0.930654 4.135744

Adduct of the reaction of butadiene with Fragment 1 substituted with two F group

Molecular formula: C34H16F2
Charge = 0 Multiplicity = 1
Hartree/particle
Thermal correction to Energy= 0.412725
Thermal correction to Enthalpy= 0.413669
Thermal correction to Gibbs Free Energy= 0.341323
Sum of electronic and zero-point Energies= -1504.485415
Sum of electronic and thermal Energies= -1504.463146
Sum of electronic and thermal Enthalpies= -1504.462202
Sum of electronic and thermal Free Energies= -1504.534548

E (Thermal) KCal/Mol CV Cal/Mol-Kelvin S Cal/Mol-Kelvin
258.989 99.496 152.266

Cartesian coordinates

Atom X Y Z
C -1.363077 -0.687245 -1.285144
C -1.215221 0.735173 -1.255774
C 0.053671 1.266050 -1.217392
C 1.216791 0.428477 -1.174837
C 1.047499 -0.950715 -1.212723
C -0.268331 -1.522498 -1.229570
C 2.218452 1.026937 -0.356292
C 3.132981 0.162904 0.272601
C 2.976169 -1.254897 0.202318
C 1.890681 -1.791854 -0.483345
C 3.823287 -2.212527 1.017240
C 3.150521 -3.666864 1.232677
C 1.705017 -3.828360 0.813614
C 1.129406 -2.998301 -0.069191
C -0.303037 -2.750569 -0.48484
C -1.520044 -3.159991 0.051473
C -2.677740 -2.280746 0.026995
C -2.537767 -1.017167 -0.589248
C -3.231201 0.175825 -0.195277
C -4.327449 0.003364 0.632711
C -4.650969 -1.309223 1.078124
C -3.845169 -2.414839 0.832204
C 0.310118 2.406677 -0.437783
C -0.749790 3.117722 0.167301
C -2.089846 2.556236 0.084208
C -2.314505 1.330016 -0.535160
C -0.333683 4.129323 1.079533
C 1.007954 4.245830 1.425645
C 2.006045 3.336197 0.977413
C 1.659268 2.369823 0.047694
H 3.864084 0.551608 0.975622
H -4.908320 0.844485 1.000837
H -5.523205 -1.433331 1.713516
H -4.075218 -3.363082 1.310780
H -2.859037 3.009958 0.705271
H -1.068848 4.768102 1.562016
H 1.295596 5.009733 2.142254
H 2.997239 3.378365 1.420345
H -1.576735 -4.045868 0.679782
H 1.178735 -4.644456 1.301358
C 4.017529 -4.783652 0.599260
H 3.578485 -5.744824 0.880879
H 3.936626 -4.703467 -0.493955
C 5.235632 -2.333363 0.385269
H 5.110554 -2.470402 -0.697657
H 5.729251 -1.367151 0.521107
C 6.036730 -3.471160 0.959258
H 7.056921 -3.298555 1.289087
C 5.451104 -4.666245 1.047260
H 5.958418 -5.533458 1.459070
F 3.173916 -3.911944 2.611198
F 4.013264 -1.657029 2.286118

Adduct of the reaction of butadiene with Fragment 1 substituted with two COOH group

Molecular formula: C36H18O4
Charge = 0 Multiplicity = 1
Hartree/particle
Thermal correction to Energy= 0.463094
Thermal correction to Enthalpy= 0.464039
Thermal correction to Gibbs Free Energy= 0.383038
Sum of electronic and zero-point Energies= -1683.108559
Sum of electronic and thermal Energies= -1683.082236
Sum of electronic and thermal Enthalpies= -1683.081292
Sum of electronic and thermal Free Energies= -1683.162293

E (Thermal) KCal/Mol CV Cal/Mol-Kelvin S Cal/Mol-Kelvin
290.596 114.144 170.481

Cartesian coordinates

Atom X Y Z
C -1.287226 -0.886653 -1.110150
C -1.100904 0.523613 -1.256040
C 0.182216 1.018204 -1.317397
C 1.322598 0.156380 -1.203016
C 1.114962 -1.209922 -1.063490
C -0.215010 -1.742310 -0.983170
C 2.361564 0.815186 -0.484383
C 3.256796 0.008688 0.240886
C 3.057781 -1.404075 0.364585
C 1.945654 -1.979399 -0.244890
C 3.778700 -2.263806 1.436453
C 3.334059 -3.786041 1.287227
C 1.820994 -3.982138 1.094004
C 1.177376 -3.140147 0.272925
C -0.261785 -2.879254 -0.103150
C -1.473342 -3.184542 0.508202
C -2.605336 -2.274914 0.414298
C -2.449673 -1.096842 -0.349627
C -3.098489 0.154587 -0.082523
C -4.173787 0.115183 0.789262
C -4.518150 -1.124864 1.396368
C -3.750246 -2.276206 1.261362
C 0.491952 2.235111 -0.685932
C -0.530578 3.043934 -0.142070
C -1.887059 2.516786 -0.121908
C -2.162104 1.232447 -0.582614
C -0.061453 4.142983 0.633003
C 1.292082 4.258456 0.929848
C 2.251641 3.272000 0.568634
C 1.852658 2.214618 -0.232186
H 3.991101 0.474558 0.891773
H -4.720893 1.011273 1.068833
H -5.374156 -1.147149 2.064833
H -3.992138 -3.154246 1.854500
H -2.625243 3.064655 0.459726
H -0.765279 4.855909 1.054632
H 1.620570 5.092065 1.543932
H 3.255051 3.334279 0.980507
H -1.536743 -3.991805 1.234042
H 1.359282 -4.824470 1.595996
C 4.018141 -4.429451 0.025471
H 3.860258 -5.511460 0.084867
H 3.471263 -4.092846 -0.862427
C 5.328700 -2.089706 1.360690
H 5.544404 -1.064058 1.046577
H 5.748573 -2.173657 2.366805
C 6.043071 -3.047722 0.454372
H 7.103114 -2.853198 0.302618
C 5.474411 -4.103680 -0.125592
H 6.061974 -4.757969 -0.766092
C 3.742317 -4.660546 2.483498
C 3.338112 -1.642657 2.778261
O 3.345919 -5.794323 2.644081
O 3.882412 -0.693150 3.298548
O 4.607802 -4.082988 3.345486
H 4.784045 -4.747245 4.033704
O 2.232878 -2.213362 3.309123
H 2.032498 -1.705181 4.114203

Adduct of the reaction of butadiene with Fragment 1 substituted with two CF_3 group

Molecular formula: C36H16F6
Charge = 0 Multiplicity = 1

Hartree/particle
Thermal correction to Energy= 0.444354
Thermal correction to Enthalpy= 0.445299
Thermal correction to Gibbs Free Energy= 0.362355
Sum of electronic and zero-point Energies= -1980.055966
Sum of electronic and thermal Energies= -1980.028506
Sum of electronic and thermal Enthalpies= -1980.027562
Sum of electronic and thermal Free Energies= -1980.110505

E (Thermal) KCal/Mol CV Cal/Mol-Kelvin S Cal/Mol-Kelvin
278.837 119.014 174.569

Cartesian coordinates

Atom X Y Z
C -1.312662 -0.918111 -1.110270
C -1.112274 0.486723 -1.288294
C 0.175173 0.967853 -1.362529
C 1.305059 0.097563 -1.220791
C 1.084446 -1.260607 -1.045000
C -0.250145 -1.781701 -0.963233
C 2.352363 0.762438 -0.523120
C 3.237564 -0.032864 0.227018
C 3.012451 -1.436534 0.414260
C 1.900288 -2.011611 -0.196556
C 3.703054 -2.288842 1.515068
C 3.304661 -3.832747 1.261330
C 1.788254 -4.042346 1.088523
C 1.127794 -3.172759 0.312389
C -0.310015 -2.902895 -0.064179
C -1.522833 -3.179819 0.556910
C -2.644501 -2.258297 0.445510
C -2.476941 -1.099227 -0.344293
C -3.111439 0.164818 -0.104557
C -4.184542 0.156530 0.771022
C -4.540489 -1.065164 1.407332
C -3.785774 -2.227817 1.296364
C 0.498898 2.197250 -0.764040
C -0.515378 3.028869 -0.239399
C -1.876529 2.514419 -0.202840
C -2.165360 1.221632 -0.631047
C -0.034566 4.143964 0.504983
C 1.320836 4.255735 0.794454
C 2.270728 3.251371 0.457808
C 1.860746 2.175932 -0.312724
H 3.991915 0.454230 0.834106
H -4.720864 1.064599 1.032047
H -5.393701 -1.062333 2.079569
H -4.034536 -3.088778 1.911115
H -2.608044 3.083719 0.366398
H -0.730745 4.873831 0.909801
H 1.658314 5.102617 1.384946
H 3.275803 3.317186 0.865187
H -1.594586 -3.969901 1.300565
H 1.322077 -4.893022 1.572338
C 4.001805 -4.299191 -0.067882
H 3.942253 -5.389593 -0.115762
H 3.398857 -3.936213 -0.905865
C 5.246909 -2.088555 1.507456
H 5.452226 -1.018198 1.410441
H 5.657762 -2.379815 2.479173
C 5.965857 -2.839837 0.426759
H 6.997081 -2.549106 0.240068
C 5.419276 -3.842839 -0.256433
H 5.995095 -4.360067 -1.020845
C 3.732312 -4.799973 2.391365
C 3.208062 -1.747125 2.888122
F 2.967872 -4.668523 3.496080
F 3.567178 -6.087253 1.988126
F 5.020267 -4.674327 2.763339
F 3.832083 -2.329500 3.935688
F 3.476695 -0.421833 3.001457
F 1.884494 -1.897415 3.069950

Adduct of the reaction of butadiene with Fragment 1 substituted with two CHO group

```
Molecular formula: C36H18O2
Charge = 0 Multiplicity = 1
                                                  Hartree/particle
Thermal correction to Energy= 0.449710
Thermal correction to Enthalpy= 0.450654
Thermal correction to Gibbs Free Energy= 0.372121
Sum of electronic and zero-point Energies= -1532.616393
Sum of electronic and thermal Energies= -1532.591482
Sum of electronic and thermal Enthalpies= -1532.590538
Sum of electronic and thermal Free Energies= -1532.669072

E (Thermal) KCal/Mol CV Cal/Mol-Kelvin S Cal/Mol-Kelvin
 282.197 107.666 165.287


 Cartesian coordinates

Atom X Y Z
 C -1.318227 -0.879295 -1.158213
 C -1.128390 0.532778 -1.283667
 C 0.155698 1.026218 -1.330918
 C 1.293523 0.160952 -1.220056
 C 1.082620 -1.207412 -1.102251
 C -0.249086 -1.739401 -1.038058
 C 2.327895 0.809069 -0.484646
 C 3.214893 -0.009579 0.238221
 C 3.016203 -1.424837 0.337799
 C 1.907205 -1.989544 -0.291603
 C 3.678081 -2.289547 1.431739
 C 3.273078 -3.823218 1.224908
 C 1.771638 -4.016986 1.021873
 C 1.133360 -3.158541 0.208766
 C -0.303355 -2.888152 -0.174317
 C -1.518582 -3.198401 0.427764
 C -2.648161 -2.285477 0.339591
 C -2.485412 -1.097445 -0.407256
 C -3.133631 0.151376 -0.127510
 C -4.213651 0.102125 0.737762
 C -4.563805 -1.145243 1.326392
 C -3.797525 -2.296169 1.180553
 C 0.463296 2.234456 -0.681976
 C -0.561398 3.037614 -0.134009
 C -1.918582 2.512134 -0.128571
 C -2.192551 1.234202 -0.607460
 C -0.095558 4.126170 0.657593
 C 1.256318 4.236770 0.963779
 C 2.216614 3.253638 0.596131
 C 1.820836 2.206761 -0.220055
 H 3.955182 0.450231 0.888195
 H -4.760507 0.995341 1.026624
 H -5.422982 -1.174372 1.990326
 H -4.043873 -3.181227 1.761061
 H -2.659497 3.053620 0.455401
 H -0.800962 4.834431 1.084210
 H 1.581989 5.062217 1.590070
 H 3.217944 3.310763 1.013897
 H -1.587433 -4.015320 1.142198
 H 1.296472 -4.847973 1.532171
 C 4.037267 -4.405654 -0.014394
 H 3.947237 -5.500138 0.006901
 H 3.512044 -4.088151 -0.922868
 C 5.208908 -2.116165 1.527219
 H 5.458346 -1.062740 1.360677
 H 5.526320 -2.304214 2.561063
 C 5.996836 -2.972877 0.578169
 H 7.052151 -2.730663 0.470925
 C 5.482859 -4.001962 -0.097006
 H 6.114800 -4.583516 -0.764981
 C 3.741888 -4.626927 2.442071
 H 4.837809 -4.628126 2.607907
 C 3.024970 -1.778065 2.734429
 H 1.919207 -1.845082 2.751631
 O 3.006491 -5.257929 3.169612
 O 3.638975 -1.302254 3.663873
```

Adduct of the reaction of butadiene with Fragment 1 substituted with two CN group

Molecular formula: C36H16N2
Charge = 0 Multiplicity = 1
Hartree/particle
Thermal correction to Energy= 0.427747
Thermal correction to Enthalpy= 0.428691
Thermal correction to Gibbs Free Energy= 0.351452
Sum of electronic and zero-point Energies= -1490.466136
Sum of electronic and thermal Energies= -1490.441767
Sum of electronic and thermal Enthalpies= -1490.440823
Sum of electronic and thermal Free Energies= -1490.518061

E (Thermal) KCal/Mol CV Cal/Mol-Kelvin S Cal/Mol-Kelvin
268.415 106.284 162.562

Cartesian coordinates

Atom X Y Z
C -1.330166 -0.727734 -1.247177
C -1.182837 0.695290 -1.249428
C 0.085455 1.227933 -1.218630
C 1.247548 0.391024 -1.148938
C 1.078113 -0.987667 -1.147954
C -0.236775 -1.561792 -1.166561
C 2.244431 1.008105 -0.339796
C 3.156327 0.160300 0.315234
C 2.991993 -1.259071 0.291437
C 1.913870 -1.811627 -0.390779
C 3.856956 -2.218752 1.125499
C 3.208046 -3.715807 1.276720
C 1.734881 -3.866219 0.881638
C 1.154153 -3.016439 0.023783
C -0.276957 -2.774991 -0.399262
C -1.496402 -3.172685 0.140111
C -2.653308 -2.293333 0.090104
C -2.509059 -1.042961 -0.551601
C -3.204107 0.158375 -0.187541
C -4.304411 0.003653 0.638534
C -4.630819 -1.299033 1.110144
C -3.824638 -2.410136 0.892186
C 0.338998 2.385850 -0.463680
C -0.724101 3.108340 0.122099
C -2.063458 2.544692 0.045632
C -2.285685 1.305180 -0.547980
C -0.312679 4.138104 1.016061
C 1.026904 4.262118 1.367359
C 2.027467 3.344051 0.943048
C 1.685456 2.360642 0.029538
H 3.884609 0.576781 1.005995
H -4.886876 0.852422 0.985775
H -5.506034 -1.409028 1.743846
H -4.057538 -3.347703 1.389745
H -2.835280 3.011848 0.653188
H -1.050562 4.785467 1.482517
H 1.310159 5.038889 2.071514
H 3.015214 3.393630 1.392515
H -1.557370 -4.044826 0.786774
H 1.208611 -4.700284 1.335714
C 4.054883 -4.748144 0.433290
H 3.627360 -5.742476 0.581581
H 3.907741 -4.489701 -0.623547
C 5.270849 -2.308547 0.412275
H 5.063144 -2.372603 -0.663400
H 5.788952 -1.360942 0.573526
C 6.091499 -3.491251 0.840986
H 7.122354 -3.349839 1.150618
C 5.506679 -4.690952 0.819642
H 6.023457 -5.597682 1.117807
C 3.299870 -4.167102 2.681082
C 4.078818 -1.618753 2.454690
N 3.321199 -4.604964 3.755994
N 4.288235 -1.074502 3.458860

Adduct of the reaction of butadiene with Fragment 1 substituted with two NO_2 group

```
Molecular formula: C34H16N2O4
Charge = 0 Multiplicity = 1
                                                 Hartree/particle
Thermal correction to Energy= 0.437682
Thermal correction to Enthalpy= 0.438627
Thermal correction to Gibbs Free Energy= 0.359034
Sum of electronic and zero-point Energies= -1714.990855
Sum of electronic and thermal Energies= -1714.965173
Sum of electronic and thermal Enthalpies= -1714.964229
Sum of electronic and thermal Free Energies= -1715.043821

E (Thermal) KCal/Mol CV Cal/Mol-Kelvin S Cal/Mol-Kelvin
 274.650 111.215 167.516


 Cartesian coordinates

Atom X Y Z
 C -1.207257 -0.672351 -1.211975
 C -0.968068 0.731575 -1.344869
 C 0.331801 1.184411 -1.359230
 C 1.435638 0.284649 -1.202524
 C 1.178472 -1.074913 -1.080855
 C -0.170727 -1.564760 -1.051322
 C 2.469957 0.908683 -0.445527
 C 3.315150 0.073226 0.304834
 C 3.060815 -1.331535 0.413480
 C 1.951676 -1.871364 -0.234768
 C 3.710002 -2.217802 1.492725
 C 3.217460 -3.702357 1.313970
 C 1.714203 -3.868196 1.094384
 C 1.126798 -3.008456 0.250491
 C -0.287836 -2.700540 -0.177441
 C -1.528513 -2.964899 0.392837
 C -2.625417 -2.018375 0.259916
 C -2.402807 -0.845019 -0.494447
 C -3.018385 0.426714 -0.247247
 C -4.124638 0.421412 0.585618
 C -4.531884 -0.807403 1.176476
 C -3.798615 -1.983421 1.066450
 C 0.659391 2.389285 -0.713654
 C -0.355304 3.230060 -0.204828
 C -1.727788 2.747321 -0.234918
 C -2.029026 1.473631 -0.709252
 C 0.120920 4.311398 0.590406
 C 1.465744 4.382395 0.936738
 C 2.404881 3.365827 0.607916
 C 2.000760 2.324406 -0.211078
 H 4.045307 0.513162 0.976659
 H -4.650872 1.334601 0.849011
 H -5.410435 -0.801416 1.815086
 H -4.089183 -2.853020 1.649691
 H -2.467962 3.318201 0.321272
 H -0.574154 5.045447 0.989354
 H 1.798105 5.202681 1.566132
 H 3.393521 3.394058 1.057297
 H -1.643425 -3.768656 1.115937
 H 1.222196 -4.692440 1.594343
 C 3.960489 -4.371498 0.115191
 H 3.777350 -5.447904 0.189197
 H 3.459298 -4.040860 -0.800914
 C 5.249936 -2.074888 1.552345
 H 5.514162 -1.049051 1.283821
 H 5.584120 -2.212631 2.583235
 C 5.989026 -3.031890 0.662015
 H 7.057569 -2.855044 0.565329
 C 5.427694 -4.066734 0.039360
 H 6.030900 -4.726973 -0.578787
 O 2.165473 -2.178744 3.334704
 O 3.715678 -0.654428 3.285807
 O 4.342142 -4.078022 3.401213
 O 3.144988 -5.691463 2.579450
 N 3.153053 -1.648517 2.842535
 N 3.598225 -4.553056 2.553657
```

Adduct of the reaction of butadiene with Fragment 2 substituted with two NH_2 group

Molecular formula: C34H18N2
Charge = 0 Multiplicity = 1
Hartree/particle
Thermal correction to Energy= 0.439875
Thermal correction to Enthalpy= 0.440819
Thermal correction to Gibbs Free Energy= 0.369099
Sum of electronic and zero-point Energies= -1415.414059
Sum of electronic and thermal Energies= -1415.391539
Sum of electronic and thermal Enthalpies= -1415.390595
Sum of electronic and thermal Free Energies= -1415.462315

E (Thermal) KCal/Mol CV Cal/Mol-Kelvin S Cal/Mol-Kelvin
276.026 102.010 150.948

Cartesian coordinates

Atom X Y Z
C 1.472356 -0.282358 2.585647
C 0.750886 0.954251 2.562158
C -0.651120 0.654106 2.548570
C -0.797151 -0.771595 2.568040
C 0.515912 -1.349210 2.593430
C 1.217501 1.987071 1.782395
C 0.328701 2.881587 1.060998
C -1.038673 2.587704 1.047448
C -1.494514 1.406541 1.761482
C -2.611322 0.820944 1.048614
C -2.752730 -0.573732 1.064287
C -1.774539 -1.356242 1.794015
C -1.568587 -2.622225 1.109037
C -0.288721 -3.185051 1.130187
C 0.762177 -2.471823 1.838305
C 2.023266 -2.684448 1.163812
C 2.958799 -1.651143 1.169346
C 2.631889 -0.408616 1.827703
C 2.497111 1.926741 1.110019
C 3.204905 0.722644 1.137394
C 1.203980 3.596836 0.120911
C 2.456658 3.051860 0.151088
C -2.099075 2.865751 0.067318
C -3.007009 1.843282 0.066305
C -3.348712 -1.517269 0.105561
C -2.664843 -2.701247 0.132424
C 0.425620 -4.078172 0.204466
C 1.762097 -3.793842 0.223104
C 4.183869 -1.424150 0.295363
C 4.352851 0.213126 0.257757
H 0.885749 4.391893 -0.542483
H -2.113037 3.712532 -0.608214
H -3.848189 1.758020 -0.611147
H -4.156771 -1.280103 -0.576007
H -2.849917 -3.542742 -0.524246
H -0.049336 -4.797684 -0.451445
H 2.514711 -4.231014 -0.418862
H 3.276943 3.367892 -0.480764
C 5.726277 0.645352 0.852479
H 5.883196 1.711576 0.651864
C 5.437995 -2.086522 0.948227
H 5.355219 -3.165929 0.803128
C 6.856459 -0.203049 0.326716
H 7.746789 0.271331 -0.076634
C 6.717872 -1.529925 0.378401
H 7.490303 -2.204844 0.019782
H 5.408310 -1.901658 2.031497
H 5.670920 0.550410 1.945335
N 4.010877 -2.039712 -1.019758
H 3.132808 -1.730097 -1.431162
H 4.739997 -1.673925 -1.630880
N 4.325956 0.662914 -1.139220
H 3.371440 0.678226 -1.489752
H 4.670259 1.616414 -1.205021

Adduct of the reaction of butadiene with Fragment 2 substituted with two OMe group

Molecular formula: C36H20O2
Charge = 0 Multiplicity = 1
Hartree/particle
Thermal correction to Energy= 0.472928
Thermal correction to Enthalpy= 0.473872
Thermal correction to Gibbs Free Energy= 0.397105
Sum of electronic and zero-point Energies= -1533.708002
Sum of electronic and thermal Energies= -1533.683034
Sum of electronic and thermal Enthalpies= -1533.682090
Sum of electronic and thermal Free Energies= -1533.758858

E (Thermal) KCal/Mol CV Cal/Mol-Kelvin S Cal/Mol-Kelvin
296.767 109.839 161.571

Cartesian coordinates

Atom X Y Z
C 1.458905 -0.340166 2.605334
C 0.716127 0.882895 2.651761
C -0.681545 0.558833 2.609507
C -0.801197 -0.867143 2.532257
C 0.522167 -1.421296 2.527895
C 1.168450 1.968682 1.937347
C 0.267956 2.888036 1.259917
C -1.093723 2.576310 1.227561
C -1.533085 1.345303 1.865887
C -2.636851 0.787634 1.110710
C -2.753660 -0.607330 1.035157
C -1.764602 -1.417211 1.715791
C -1.529703 -2.628435 0.946956
C -0.238094 -3.162938 0.936341
C 0.795844 -2.481606 1.696092
C 2.070596 -2.631862 1.025662
C 2.995989 -1.595237 1.130392
C 2.627903 -0.402174 1.855245
C 2.444276 1.962929 1.258109
C 3.172408 0.775825 1.232715
C 1.132386 3.656353 0.350613
C 2.388527 3.121518 0.345129
C -2.153902 2.896033 0.260538
C -3.045846 1.862098 0.192514
C -3.326842 -1.495261 0.010851
C -2.620218 -2.664597 -0.038706
C 0.496418 -3.976763 -0.042771
C 1.827170 -3.674474 0.007745
C 4.278306 -1.280692 0.355717
C 4.326766 0.359044 0.326145
H 0.796530 4.466151 -0.285545
H -2.176926 3.781225 -0.363509
H -3.881814 1.804705 -0.494214
H -4.135626 -1.227478 -0.658411
H -2.784791 -3.462948 -0.752175
H 0.039260 -4.654255 -0.753700
H 2.573649 -4.081183 -0.659950
H 3.197682 3.430973 -0.301815
C 5.672407 0.916995 0.890221
H 5.768162 1.958331 0.569071
C 5.502002 -1.833780 1.141673
H 5.505855 -2.925813 1.100990
C 6.877297 0.088920 0.528230
H 7.784742 0.581380 0.188370
C 6.802527 -1.236707 0.666966
H 7.645526 -1.887319 0.449597
H 5.340886 -1.578732 2.197758
H 5.581569 0.947673 1.984593
O 4.202900 -1.739202 -0.991390
O 4.030950 0.882384 -0.958334
C 4.954159 -2.864738 -1.408727
H 4.682934 -3.025092 -2.455235
H 6.036801 -2.694025 -1.352684
H 4.715692 -3.779543 -0.849466
C 5.048358 0.905044 -1.949275
H 5.453088 -0.088168 -2.153080
H 4.566337 1.286862 -2.852757
H 5.871828 1.583181 -1.685372

Adduct of the reaction of butadiene with Fragment 2 substituted with two OH group

Molecular formula: C34H16O2
Charge = 0 Multiplicity = 1
Hartree/particle
Thermal correction to Energy= 0.414155
Thermal correction to Enthalpy= 0.415099
Thermal correction to Gibbs Free Energy= 0.344447
Sum of electronic and zero-point Energies= -1455.178554
Sum of electronic and thermal Energies= -1455.156474
Sum of electronic and thermal Enthalpies= -1455.155530
Sum of electronic and thermal Free Energies= -1455.226182

E (Thermal) KCal/Mol CV Cal/Mol-Kelvin S Cal/Mol-Kelvin
259.886 99.997 148.699

Cartesian coordinates

Atom X Y Z
C 1.441573 -0.283962 2.630156
C 0.708363 0.947109 2.606546
C -0.691215 0.632182 2.566292
C -0.823069 -0.794895 2.562440
C 0.495616 -1.360013 2.598702
C 1.175869 1.993308 1.843711
C 0.290424 2.881323 1.107990
C -1.073498 2.578464 1.077132
C -1.528984 1.386006 1.774348
C -2.630734 0.799134 1.039146
C -2.759547 -0.596768 1.035453
C -1.783800 -1.378814 1.766713
C -1.554171 -2.631217 1.063534
C -0.268415 -3.178085 1.091741
C 0.764196 -2.469109 1.829444
C 2.035848 -2.654958 1.166121
C 2.961052 -1.616704 1.215570
C 2.615973 -0.389354 1.891712
C 2.460210 1.945607 1.181444
C 3.182613 0.756162 1.223062
C 1.170558 3.592930 0.167166
C 2.424220 3.052410 0.205356
C -2.122202 2.856336 0.084801
C -3.022517 1.827760 0.062465
C -3.332260 -1.531409 0.053591
C -2.635936 -2.707799 0.070747
C 0.467198 -4.037517 0.150215
C 1.798544 -3.736581 0.190070
C 4.190999 -1.348808 0.367646
C 4.325728 0.272095 0.333428
H 0.846784 4.369211 -0.515431
H -2.132890 3.708990 -0.583302
H -3.853898 1.742062 -0.626858
H -4.133363 -1.291681 -0.635244
H -2.802043 -3.540820 -0.601520
H 0.007535 -4.740556 -0.533754
H 2.556850 -4.146828 -0.462960
H 3.244304 3.318441 -0.447943
C 5.710726 0.751696 0.826425
H 5.821889 1.801597 0.541907
C 5.460095 -2.019625 0.954287
H 5.380628 -3.098453 0.791386
C 6.821349 -0.104756 0.269411
H 7.694461 0.371859 -0.167747
C 6.708683 -1.435047 0.337768
H 7.486635 -2.095905 -0.035382
H 5.469187 -1.864396 2.042079
H 5.728940 0.715446 1.924342
O 3.963223 -1.831264 -0.956392
H 4.786279 -1.670049 -1.444885
O 4.219983 0.738984 -1.003829
H 3.545506 0.186775 -1.430256

Adduct of the reaction of butadiene with Fragment 2 substituted with two Me group

```
Molecular formula: C36H20
Charge = 0 Multiplicity = 1
                                                  Hartree/particle
Thermal correction to Energy= 0.462351
Thermal correction to Enthalpy= 0.463295
Thermal correction to Gibbs Free Energy= 0.390888
Sum of electronic and zero-point Energies= -1383.317396
Sum of electronic and thermal Energies= -1383.294803
Sum of electronic and thermal Enthalpies= -1383.293859
Sum of electronic and thermal Free Energies= -1383.366266

E (Thermal) KCal/Mol CV Cal/Mol-Kelvin S Cal/Mol-Kelvin
 290.129 102.714 152.393


 Cartesian coordinates

Atom X Y Z
 C 1.453900 -0.280343 2.526161
 C 0.723946 0.949435 2.549085
 C -0.675039 0.637312 2.580942
 C -0.807675 -0.789766 2.584551
 C 0.509865 -1.354788 2.555509
 C 1.157011 1.992268 1.766291
 C 0.236615 2.894837 1.095852
 C -1.127969 2.587918 1.122582
 C -1.549800 1.391483 1.831666
 C -2.684402 0.805004 1.146974
 C -2.813949 -0.591219 1.150089
 C -1.806202 -1.373351 1.837605
 C -1.612064 -2.630184 1.134223
 C -0.327262 -3.183239 1.110002
 C 0.743285 -2.463191 1.777953
 C 1.989655 -2.666543 1.069024
 C 2.917589 -1.619533 1.051740
 C 2.584558 -0.387564 1.727189
 C 2.417153 1.957559 1.053691
 C 3.134376 0.756088 1.038728
 C 1.077206 3.645713 0.153927
 C 2.336128 3.115458 0.134628
 C -2.222913 2.869940 0.182324
 C -3.120843 1.838319 0.194176
 C -3.433333 -1.530837 0.201640
 C -2.740684 -2.710159 0.194909
 C 0.361388 -4.081254 0.173435
 C 1.697000 -3.794209 0.154972
 C 4.168067 -1.361011 0.206639
 C 4.305096 0.265371 0.180865
 H 0.733725 4.456313 -0.477156
 H -2.266165 3.725637 -0.480663
 H -3.981801 1.754884 -0.458193
 H -4.264703 -1.293802 -0.451393
 H -2.941198 -3.546521 -0.463779
 H -0.125099 -4.816835 -0.455557
 H 2.415337 -4.283968 -0.488370
 H 3.131993 3.463048 -0.509994
 C 5.643643 0.719413 0.865716
 H 5.790530 1.788241 0.682401
 C 5.400785 -2.001235 0.939816
 H 5.358279 -3.088279 0.821797
 C 6.820672 -0.099557 0.403948
 H 7.726435 0.392384 0.059185
 C 6.703154 -1.429519 0.443319
 H 7.506637 -2.091735 0.131601
 H 5.306082 -1.805572 2.017235
 H 5.532293 0.608318 1.953529
 C 4.084981 -1.986533 -1.197260
 H 4.036713 -3.074748 -1.117113
 H 3.204112 -1.654267 -1.751232
 H 4.975869 -1.749839 -1.783981
 C 4.287050 0.844552 -1.245913
 H 3.348559 0.640214 -1.766719
 H 4.419123 1.928016 -1.213366
 H 5.110482 0.441274 -1.840498
```

Adduct of the reaction of butadiene with Fragment 2 substituted with two *i*-Pr group

Molecular formula: C40H28
Charge = 0 Multiplicity = 1
Hartree/particle
Thermal correction to Energy= 0.581838
Thermal correction to Enthalpy= 0.582783
Thermal correction to Gibbs Free Energy= 0.501076
Sum of electronic and zero-point Energies= -1540.436881
Sum of electronic and thermal Energies= -1540.409009
Sum of electronic and thermal Enthalpies= -1540.408065
Sum of electronic and thermal Free Energies= -1540.489772

E (Thermal) KCal/Mol CV Cal/Mol-Kelvin S Cal/Mol-Kelvin
365.109 123.461 171.965

Cartesian coordinates

Atom X Y Z
C 1.631862 -0.634525 2.477295
C 0.998997 0.602552 2.793519
C -0.416967 0.383354 2.827301
C -0.654489 -1.000587 2.535532
C 0.613893 -1.636552 2.338708
C 1.488609 1.759768 2.237894
C 0.618109 2.855225 1.841447
C -0.764128 2.634562 1.855375
C -1.252966 1.338845 2.297845
C -2.442888 0.991487 1.545841
C -2.672264 -0.360380 1.251581
C -1.710852 -1.339983 1.718609
C -1.625603 -2.425361 0.758828
C -0.385991 -3.051791 0.575603
C 0.747158 -2.571807 1.343108
C 1.975352 -2.741228 0.585155
C 2.985351 -1.789913 0.753485
C 2.705048 -0.647255 1.602730
C 2.696660 1.789090 1.438362
C 3.276577 0.564393 1.071881
C 1.484168 3.751177 1.063142
C 2.687363 3.142451 0.839906
C -1.859515 3.186657 1.044256
C -2.826554 2.236152 0.861583
C -3.378678 -1.028862 0.146504
C -2.775316 -2.223264 -0.136375
C 0.215870 -3.784859 -0.545015
C 1.573992 -3.627179 -0.532820
C 4.338621 -1.447171 0.096260
C 4.163742 0.167852 -0.137840
H 1.195971 4.723387 0.682435
H -1.858584 4.169287 0.588081
H -3.705075 2.354986 0.238785
H -4.203009 -0.600590 -0.411007
H -3.050254 -2.882404 -0.951004
H -0.337992 -4.316913 -1.309133
H 2.236383 -4.020126 -1.291304
H 3.499108 3.578108 0.273343
C 5.507651 0.941557 -0.149446
H 5.926273 0.947322 -1.164501
C 5.394885 -1.717719 1.242665
H 5.810809 -2.720321 1.106720
C 6.565107 0.448665 0.790855
H 7.414994 1.108266 0.955798
C 6.521777 -0.739629 1.386879
H 7.325141 -1.037912 2.058919
H 4.881279 -1.769906 2.210252
H 5.300877 1.994232 0.075617
C 4.861716 -3.808276 -0.851542
H 4.717346 -4.391083 -1.767898
H 5.871027 -4.036515 -0.494583
H 4.158431 -4.176080 -0.102881
C 3.479363 1.894162 -2.056008
H 4.469438 2.352205 -2.002356
H 3.190208 1.883226 -3.112451
H 2.770148 2.543256 -1.539650
C 4.700141 -2.299329 -1.164086
H 3.860193 -2.207712 -1.861866
C 3.424192 0.444912 -1.526152
H 3.988838 -0.143716 -2.254432
C 1.956794 -0.011945 -1.614535
H 1.823366 -1.084504 -1.480932
H 1.332956 0.499784 -0.875393
H 1.562697 0.243723 -2.604266
C 5.977481 -1.855929 -1.905109
H 6.840950 -1.848208 -1.233202
H 6.193775 -2.561962 -2.714143
H 5.897885 -0.864828 -2.355499

Adduct of the reaction of butadiene with Fragment 2 substituted with two F group

```
Molecular formula: C34H14F2
Charge = 0 Multiplicity = 1
                                                Hartree/particle
Thermal correction to Energy= 0.388544
Thermal correction to Enthalpy= 0.389488
Thermal correction to Gibbs Free Energy= 0.319310
Sum of electronic and zero-point Energies= -1503.230641
Sum of electronic and thermal Energies= -1503.209050
Sum of electronic and thermal Enthalpies= -1503.208106
Sum of electronic and thermal Free Energies= -1503.278283

E (Thermal) KCal/Mol CV Cal/Mol-Kelvin S Cal/Mol-Kelvin
 243.815 97.100 147.701
```

```
 Cartesian coordinates

Atom X Y Z
 C 1.418114 -0.267713 2.657290
 C 0.684827 0.963101 2.613693
 C -0.714672 0.647177 2.572348
 C -0.845840 -0.780190 2.583034
 C 0.473032 -1.344919 2.630504
 C 1.154169 2.001449 1.840669
 C 0.271367 2.878603 1.089630
 C -1.092146 2.576308 1.058959
 C -1.549541 1.391922 1.769139
 C -2.649196 0.797434 1.037061
 C -2.777262 -0.598040 1.047330
 C -1.803257 -1.372120 1.789476
 C -1.570196 -2.630297 1.097236
 C -0.284310 -3.175045 1.133792
 C 0.744650 -2.462260 1.872920
 C 2.017434 -2.650073 1.212896
 C 2.941134 -1.612124 1.259170
 C 2.595336 -0.381001 1.924507
 C 2.439479 1.945290 1.180704
 C 3.158901 0.757404 1.243167
 C 1.152881 3.573650 0.137286
 C 2.405334 3.032549 0.184421
 C -2.138198 2.843677 0.061115
 C -3.038458 1.815246 0.048057
 C -3.346147 -1.542037 0.072231
 C -2.648490 -2.717076 0.101678
 C 0.454382 -4.032412 0.192350
 C 1.784451 -3.727548 0.232645
 C 4.146422 -1.340776 0.377776
 C 4.293444 0.259386 0.366796
 H 0.829495 4.336341 -0.560472
 H -2.146459 3.688476 -0.616784
 H -3.867729 1.721605 -0.642670
 H -4.145015 -1.309023 -0.621387
 H -2.810888 -3.555976 -0.564030
 H -0.003366 -4.733955 -0.494303
 H 2.542355 -4.136275 -0.422518
 H 3.224456 3.286787 -0.475163
 C 5.686635 0.730630 0.824650
 H 5.780352 1.789212 0.565725
 C 5.431226 -2.052033 0.843250
 H 5.330312 -3.113108 0.597612
 C 6.782186 -0.107139 0.211253
 H 7.644275 0.385558 -0.228643
 C 6.660338 -1.435848 0.220462
 H 7.417847 -2.083069 -0.211684
 H 5.487372 -1.987878 1.939103
 H 5.731833 0.671114 1.921284
 F 4.125069 0.708668 -0.942407
 F 3.897416 -1.768869 -0.925735
```

Adduct of the reaction of butadiene with Fragment 2 substituted with two COOH group

Molecular formula: C36H16O4
Charge = 0 Multiplicity = 1
Hartree/particle
Thermal correction to Energy= 0.438343
Thermal correction to Enthalpy= 0.439287
Thermal correction to Gibbs Free Energy= 0.360161
Sum of electronic and zero-point Energies= -1681.841789
Sum of electronic and thermal Energies= -1681.816208
Sum of electronic and thermal Enthalpies= -1681.815264
Sum of electronic and thermal Free Energies= -1681.894390

E (Thermal) KCal/Mol CV Cal/Mol-Kelvin S Cal/Mol-Kelvin
275.064 111.933 166.534

Cartesian coordinates

Atom X Y Z
C 1.470931 -0.213559 2.541837
C 0.730456 1.011947 2.509319
C -0.665570 0.686169 2.527248
C -0.785305 -0.740278 2.588937
C 0.538504 -1.294122 2.603795
C 1.165756 2.034005 1.701904
C 0.254683 2.899426 0.974869
C -1.105570 2.572730 0.979417
C -1.529537 1.399604 1.726973
C -2.642767 0.772950 1.043911
C -2.761181 -0.623179 1.106440
C -1.763094 -1.365419 1.849945
C -1.547621 -2.654521 1.211132
C -0.261081 -3.201344 1.237997
C 0.793281 -2.437678 1.885507
C 2.039388 -2.648269 1.180543
C 2.931583 -1.576885 1.093348
C 2.603062 -0.337783 1.746616
C 2.442323 1.993785 1.020334
C 3.157579 0.798144 1.053892
C 1.114688 3.628565 0.029131
C 2.381448 3.118309 0.062390
C -2.182989 2.802609 0.005001
C -3.068965 1.761425 0.039715
C -3.355118 -1.610742 0.191148
C -2.656681 -2.784987 0.254318
C 0.449155 -4.134335 0.349844
C 1.777398 -3.820896 0.320251
C 4.123108 -1.321324 0.160604
C 4.370959 0.295794 0.268629
H 0.777455 4.411760 -0.638886
H -2.221893 3.630608 -0.692424
H -3.914728 1.641534 -0.626643
H -4.174034 -1.410088 -0.489213
H -2.839635 -3.652629 -0.367962
H -0.022694 -4.907103 -0.244641
H 2.513977 -4.313513 -0.301624
H 3.199684 3.445043 -0.566346
C 5.673923 0.574856 1.086456
H 5.848204 1.651416 1.101740
C 5.380371 -2.120626 0.658625
H 5.309488 -3.147561 0.292339
C 6.839876 -0.176551 0.501655
H 7.745691 0.360087 0.239400
C 6.686349 -1.480892 0.270133
H 7.461941 -2.087655 -0.186035
H 5.310980 -2.173984 1.752835
H 5.486550 0.258400 2.120848
O 4.778001 -1.987584 -2.082445
O 5.445838 1.764649 -1.357697
O 2.585221 -1.810833 -1.641404
O 3.474751 0.852949 -1.903009
C 3.885811 -1.737078 -1.300419
C 4.525195 1.031799 -1.071749
H 2.569611 -2.034926 -2.587225
H 3.675876 1.371320 -2.700753

Adduct of the reaction of butadiene with Fragment 2 substituted with two CF_3 group

Molecular formula: C36H14F6
Charge = 0 Multiplicity = 1
Hartree/particle
Thermal correction to Energy= 0.419958
Thermal correction to Enthalpy= 0.420903
Thermal correction to Gibbs Free Energy= 0.339406
Sum of electronic and zero-point Energies= -1978.791172
Sum of electronic and thermal Energies= -1978.764311
Sum of electronic and thermal Enthalpies= -1978.763367
Sum of electronic and thermal Free Energies= -1978.844863
E (Thermal) KCal/Mol CV Cal/Mol-Kelvin S Cal/Mol-Kelvin
263.528 116.719 171.524

Cartesian coordinates

Atom X Y Z
C 1.581608 -0.043972 2.455205
C 0.792177 1.151315 2.382710
C -0.586712 0.773336 2.481403
C -0.645440 -0.649302 2.640562
C 0.699608 -1.150132 2.626389
C 1.153429 2.130260 1.493652
C 0.182392 2.912023 0.753157
C -1.162530 2.532818 0.832250
C -1.508636 1.396521 1.670169
C -2.620892 0.682541 1.076556
C -2.680735 -0.709706 1.238789
C -1.625753 -1.360056 1.989318
C -1.384219 -2.683534 1.434827
C -0.077438 -3.180389 1.444887
C 0.974488 -2.329326 1.978154
C 2.190574 -2.534295 1.214746
C 3.029595 -1.428692 1.009746
C 2.674337 -0.171627 1.614766
C 2.418335 2.115125 0.780656
C 3.189853 0.961619 0.876067
C 0.982394 3.628555 -0.252120
C 2.276231 3.191340 -0.225141
C -2.285601 2.655088 -0.109731
C -3.125599 1.584545 0.028414
C -3.269659 -1.781866 0.420786
C -2.524478 -2.923065 0.537781
C 0.628667 -4.155224 0.601538
C 1.937786 -3.792486 0.475893
C 4.046947 -1.120527 -0.126864
C 4.458597 0.412397 0.212127
H 0.589866 4.351906 -0.956402
H -2.384698 3.431713 -0.858362
H -3.989426 1.387692 -0.594989
H -4.120018 -1.660247 -0.239351
H -2.696533 -3.838329 -0.015419
H 0.166056 -4.997117 0.101444
H 2.660827 -4.310416 -0.137719
H 3.053584 3.515335 -0.901063
C 5.567925 0.438651 1.334611
H 6.099047 1.391225 1.264088
C 5.235887 -2.111040 -0.232608
H 5.610287 -2.113351 -1.263017
C 6.547087 -0.696285 1.350175
H 7.416658 -0.558849 1.989327
C 6.386229 -1.836746 0.686145
H 7.136214 -2.620590 0.761763
H 4.853240 -3.121369 -0.053409
H 5.064307 0.468292 2.306968
C 4.983018 1.259188 -0.956277
C 3.290918 -1.224702 -1.482939
F 2.871831 -2.493144 -1.702997
F 2.209081 -0.431196 -1.541798
F 4.083670 -0.929046 -2.538483
F 4.070141 1.500682 -1.916377
F 5.368499 2.486522 -0.511327
F 6.062231 0.694920 -1.535258

Adduct of the reaction of butadiene with Fragment 2 substituted with two CHO group

Molecular formula: C36H16O2
Charge = 0 Multiplicity = 1
Hartree/particle
Thermal correction to Energy= 0.425388
Thermal correction to Enthalpy= 0.426332
Thermal correction to Gibbs Free Energy= 0.350176
Sum of electronic and zero-point Energies= -1531.354139
Sum of electronic and thermal Energies= -1531.330142
Sum of electronic and thermal Enthalpies= -1531.329198
Sum of electronic and thermal Free Energies= -1531.405354

E (Thermal) KCal/Mol CV Cal/Mol-Kelvin S Cal/Mol-Kelvin
266.935 105.118 160.284

Cartesian coordinates

Atom X Y Z
C 1.445267 -0.270968 2.592653
C 0.709229 0.957331 2.583878
C -0.688360 0.637573 2.554074
C -0.814777 -0.790566 2.550418
C 0.505192 -1.350848 2.577562
C 1.168061 2.009068 1.825929
C 0.275275 2.903180 1.108762
C -1.086748 2.587643 1.075676
C -1.532064 1.388411 1.766856
C -2.633776 0.797604 1.033657
C -2.757161 -0.598542 1.029887
C -1.776548 -1.377442 1.759342
C -1.548295 -2.632516 1.061448
C -0.262603 -3.182029 1.091722
C 0.772959 -2.462507 1.813449
C 2.041688 -2.650204 1.143290
C 2.956102 -1.595587 1.160993
C 2.604362 -0.371007 1.832635
C 2.451178 1.976103 1.157594
C 3.166534 0.777451 1.169318
C 1.150485 3.645346 0.188607
C 2.410686 3.118627 0.219382
C -2.141646 2.864585 0.089413
C -3.034605 1.829410 0.063667
C -3.332021 -1.538622 0.054221
C -2.634837 -2.714699 0.074135
C 0.469021 -4.060306 0.166004
C 1.801887 -3.761956 0.197914
C 4.177875 -1.326691 0.277648
C 4.327481 0.303800 0.290044
H 0.827042 4.444964 -0.466424
H -2.161034 3.720905 -0.573644
H -3.867405 1.742611 -0.623668
H -4.136194 -1.303477 -0.632477
H -2.803682 -3.550886 -0.593359
H 0.010537 -4.786951 -0.493447
H 2.555916 -4.220558 -0.429174
H 3.234263 3.442764 -0.404005
C 5.707145 0.738607 0.864909
H 5.838493 1.805569 0.674291
C 5.460478 -2.015821 0.828629
H 5.400115 -3.083172 0.606823
C 6.831570 -0.072775 0.275594
H 7.697770 0.437291 -0.134029
C 6.711576 -1.401466 0.256622
H 7.473951 -2.046512 -0.168811
H 5.453769 -1.911232 1.921667
H 5.673595 0.605208 1.954239
O 4.618052 -2.623733 -1.733406
O 5.012722 1.551742 -1.682994
C 3.898813 -1.855439 -1.135702
C 4.167588 0.896539 -1.116275
H 2.940207 -1.517824 -1.581534
H 3.177747 0.725709 -1.588122

Adduct of the reaction of butadiene with Fragment 2 substituted with two CN group

```
Molecular formula: C36H14N2
Charge = 0 Multiplicity = 1
                                                  Hartree/particle
Thermal correction to Energy= 0.403522
Thermal correction to Enthalpy= 0.404466
Thermal correction to Gibbs Free Energy= 0.329720
Sum of electronic and zero-point Energies= -1489.213912
Sum of electronic and thermal Energies= -1489.190306
Sum of electronic and thermal Enthalpies= -1489.189362
Sum of electronic and thermal Free Energies= -1489.264109

E (Thermal) KCal/Mol CV Cal/Mol-Kelvin S Cal/Mol-Kelvin
 253.214 103.830 157.318


 Cartesian coordinates

Atom X Y Z
 C 1.458585 -0.273307 2.553518
 C 0.725460 0.956068 2.557385
 C -0.674471 0.639775 2.565092
 C -0.804018 -0.787351 2.565866
 C 0.516281 -1.350554 2.558462
 C 1.167024 2.003667 1.784613
 C 0.258934 2.898485 1.088062
 C -1.103560 2.588554 1.092151
 C -1.535176 1.391458 1.798039
 C -2.656134 0.803143 1.093749
 C -2.782705 -0.592893 1.094380
 C -1.785659 -1.372589 1.799048
 C -1.576491 -2.628493 1.094314
 C -0.291900 -3.178356 1.090491
 C 0.762379 -2.461007 1.786484
 C 2.014135 -2.653301 1.084647
 C 2.933092 -1.606435 1.098444
 C 2.604485 -0.377379 1.773852
 C 2.432941 1.967262 1.082940
 C 3.148626 0.772235 1.097836
 C 1.112146 3.631656 0.140307
 C 2.370645 3.102351 0.138141
 C -2.182229 2.866912 0.132857
 C -3.078214 1.834257 0.132135
 C -3.383269 -1.532097 0.133520
 C -2.687827 -2.709172 0.135346
 C 0.415621 -4.054360 0.143951
 C 1.748825 -3.760124 0.141394
 C 4.173416 -1.340722 0.235889
 C 4.321512 0.287719 0.236349
 H 0.771036 4.420863 -0.518193
 H -2.214410 3.720471 -0.533089
 H -3.927030 1.747401 -0.535248
 H -4.202761 -1.294938 -0.534042
 H -2.873211 -3.543842 -0.529476
 H -0.061471 -4.770792 -0.513299
 H 2.479992 -4.200308 -0.522708
 H 3.168683 3.402897 -0.526826
 C 5.694316 0.721842 0.870276
 H 5.827597 1.793520 0.707065
 C 5.446789 -2.015513 0.867224
 H 5.386114 -3.093365 0.701670
 C 6.838636 -0.090626 0.324766
 H 7.718063 0.412656 -0.064592
 C 6.718234 -1.420133 0.323019
 H 7.493137 -2.072161 -0.067721
 H 5.384663 -1.857186 1.951953
 H 5.603264 0.574792 1.954517
 C 4.272151 0.839978 -1.125265
 C 4.022483 -1.874002 -1.125733
 N 3.920018 -2.360829 -2.174777
 N 4.261096 1.336932 -2.174508
```

Adduct of the reaction of butadiene with Fragment 2 substituted with two NO_2 group

```
Molecular formula: C34H14N2O4
Charge = 0 Multiplicity = 1
                                                  Hartree/particle
Thermal correction to Energy= 0.412789
Thermal correction to Enthalpy= 0.413733
Thermal correction to Gibbs Free Energy= 0.335727
Sum of electronic and zero-point Energies= -1713.719419
Sum of electronic and thermal Energies= -1713.694383
Sum of electronic and thermal Enthalpies= -1713.693439
Sum of electronic and thermal Free Energies= -1713.771445

E (Thermal) KCal/Mol CV Cal/Mol-Kelvin S Cal/Mol-Kelvin
 259.029 109.048 164.178
```

```
 Cartesian coordinates

Atom X Y Z
 C 1.462663 -0.226965 2.548985
 C 0.725624 1.000604 2.532356
 C -0.672331 0.676978 2.543988
 C -0.795409 -0.749750 2.580945
 C 0.528440 -1.306263 2.584155
 C 1.163781 2.036937 1.744322
 C 0.255803 2.912301 1.025360
 C -1.103986 2.588910 1.022937
 C -1.532070 1.404648 1.752783
 C -2.645059 0.792188 1.057081
 C -2.766649 -0.604284 1.095822
 C -1.771171 -1.359974 1.828072
 C -1.557426 -2.639352 1.166673
 C -0.273002 -3.188126 1.185569
 C 0.781803 -2.437711 1.847185
 C 2.027838 -2.640960 1.137749
 C 2.924451 -1.571743 1.082764
 C 2.595167 -0.340890 1.753403
 C 2.439545 2.003901 1.060613
 C 3.151733 0.808153 1.089226
 C 1.116373 3.643621 0.081490
 C 2.381710 3.131173 0.106681
 C -2.178756 2.836710 0.050543
 C -3.067071 1.797568 0.068037
 C -3.361332 -1.575378 0.163387
 C -2.665868 -2.751888 0.207312
 C 0.436647 -4.107594 0.282928
 C 1.765659 -3.800471 0.259528
 C 4.132672 -1.291252 0.196098
 C 4.356959 0.310703 0.307141
 H 0.777472 4.422255 -0.590600
 H -2.212709 3.673962 -0.635684
 H -3.910504 1.689075 -0.602987
 H -4.177196 -1.360915 -0.516207
 H -2.847832 -3.607176 -0.431754
 H -0.036660 -4.862926 -0.331994
 H 2.496409 -4.277076 -0.379561
 H 3.190271 3.443667 -0.541361
 C 5.683364 0.637825 1.032825
 H 5.840687 1.716124 0.996281
 C 5.392464 -2.089227 0.647012
 H 5.311044 -3.109778 0.264220
 C 6.842408 -0.131130 0.454869
 H 7.747530 0.400035 0.181211
 C 6.694093 -1.439923 0.253511
 H 7.473535 -2.050765 -0.189477
 H 5.330227 -2.165558 1.740154
 H 5.529214 0.372200 2.087347
 N 3.884474 -1.687088 -1.288408
 N 4.380453 0.995035 -1.082814
 O 4.880331 -1.721051 -2.005071
 O 5.355643 1.676371 -1.375987
 O 2.748541 -1.950349 -1.646244
 O 3.377112 0.864593 -1.771868
```

Aromatized adduct for the reaction of butadiene with Fragment 1

Molecular formula: C34H14
Charge = 0 Multiplicity = 1
Hartree/particle
Thermal correction to Energy= 0.381663
Thermal correction to Enthalpy= 0.382608
Thermal correction to Gibbs Free Energy= 0.316562
Sum of electronic and zero-point Energies= -1303.689500
Sum of electronic and thermal Energies= -1303.670057
Sum of electronic and thermal Enthalpies= -1303.669113
Sum of electronic and thermal Free Energies= -1303.735159

E (Thermal) CV S
KCal/Mol Cal/Mol-Kelvin Cal/Mol-Kelvin
Total 239.497 89.001 139.005

Cartesian coordinates

Atom X Y Z
C -1.354713 -0.424365 -1.380729
C -1.142959 0.983749 -1.258970
C 0.147518 1.464526 -1.303205
C 1.267961 0.584483 -1.431071
C 1.031662 -0.773437 -1.537878
C -0.296782 -1.300840 -1.494399
C 2.380771 1.087751 -0.677956
C 3.312975 0.160604 -0.203920
C 3.081792 -1.264306 -0.314597
C 1.888450 -1.692462 -0.917198
C 3.817547 -2.305916 0.387151
C 3.175657 -3.580476 0.595216
C 1.810585 -3.824283 0.184953
C 1.147498 -2.890316 -0.561627
C -0.306309 -2.585958 -0.842790
C -1.484476 -2.988396 -0.220406
C -2.601622 -2.067909 -0.064621
C -2.470876 -0.765867 -0.597591
C -3.068464 0.416094 -0.046216
C -4.083689 0.218870 0.874932
C -4.417327 -1.111862 1.252526
C -3.686834 -2.222494 0.844542
C 0.528245 2.532857 -0.472690
C -0.438389 3.233441 0.282666
C -1.803142 2.725464 0.293262
C -2.139491 1.560119 -0.389182
C 0.105748 4.166158 1.211118
C 1.479246 4.217978 1.422310
C 2.392507 3.311591 0.814951
C 1.917627 2.420558 -0.133260
H 4.119288 0.492971 0.444177
H -4.587632 1.049033 1.362118
H -5.226451 -1.254758 1.963112
H -3.907607 -3.196216 1.274015
H -2.488507 3.157147 1.019390
H -0.551998 4.793360 1.807237
H 1.866497 4.922953 2.152420
H 3.425056 3.295757 1.152884
H -1.519381 -3.914892 0.348422
H 1.328607 -4.733773 0.534939
C 3.886299 -4.586408 1.291469
H 3.400009 -5.544774 1.454401
C 5.124637 -2.120544 0.883478
H 5.624584 -1.172392 0.710406
C 5.793048 -3.131250 1.552965
H 6.803945 -2.964815 1.913112
C 5.169163 -4.374355 1.760210
H 5.694866 -5.165900 2.285654

Aromatized adduct for the reaction of butadiene with Fragment 2

```
Molecular formula: C34H12
Charge = 0 Multiplicity = 1
                                                  Hartree/particle
Thermal correction to Energy= 0.356921
Thermal correction to Enthalpy= 0.357865
Thermal correction to Gibbs Free Energy= 0.293496
Sum of electronic and zero-point Energies= -1302.409936
Sum of electronic and thermal Energies= -1302.391102
Sum of electronic and thermal Enthalpies= -1302.390158
Sum of electronic and thermal Free Energies= -1302.454527

 E (Thermal) CV S
 KCal/Mol Cal/Mol-Kelvin Cal/Mol-Kelvin
Total 223.971 86.938 135.476
```

Cartesian coordinates

Atom	X	Y	Z
C	-0.375285	-0.065864	2.016806
C	0.440528	-1.237453	1.886994
C	1.793873	-0.812059	1.672021
C	1.812927	0.622392	1.671661
C	0.471353	1.083588	1.886281
C	-0.043076	-2.317656	1.184816
C	0.808237	-3.130050	0.332534
C	2.128344	-2.715878	0.123499
C	2.577838	-1.494511	0.769657
C	3.525379	-0.818671	-0.096433
C	3.544045	0.581966	-0.096769
C	2.614876	1.283292	0.769038
C	2.197984	2.515782	0.122155
C	0.889333	2.965005	0.330917
C	0.016721	2.175990	1.183626
C	-1.344208	2.267094	0.691600
C	-2.175777	1.150197	0.829398
C	-1.627874	-0.049279	1.437557
C	-1.405974	-2.372687	0.692767
C	-2.207514	-1.233992	0.829866
C	-0.124606	-3.907020	-0.495428
C	-1.403711	-3.470591	-0.287599
C	3.054838	-2.906640	-1.001416
C	3.861769	-1.809614	-1.129184
C	3.906557	1.562990	-1.130141
C	3.129121	2.681172	-1.002945
C	-0.022454	3.765875	-0.497699
C	-1.312728	3.363809	-0.289584
C	-3.366271	0.689832	0.087456
C	-3.385333	-0.742423	0.087703
H	0.167245	-4.655052	-1.222561
H	3.043105	-3.753086	-1.677386
H	4.584262	-1.657190	-1.921899
H	4.624665	1.390853	-1.922801
H	3.139825	3.527175	-1.679493
H	0.289366	4.505107	-1.225546
H	-2.172193	3.737232	-0.831899
H	-2.272830	-3.821652	-0.829467
C	-4.400238	-1.426186	-0.577818
H	-4.430745	-2.511557	-0.572742
C	-4.362468	1.400217	-0.578401
H	-4.363843	2.486020	-0.573827
C	-5.396963	-0.698595	-1.236556
H	-6.197508	-1.225777	-1.746860
C	-5.378306	0.699253	-1.236840
H	-6.164480	1.247406	-1.747389

NH_2-H

Molecular formula: NH3
Charge = 0 Multiplicity = 1
Hartree/particle
Thermal correction to Energy= 0.037302
Thermal correction to Enthalpy= 0.038246
Thermal correction to Gibbs Free Energy= 0.015363
Sum of electronic and zero-point Energies= -56.523329
Sum of electronic and thermal Energies= -56.520467
Sum of electronic and thermal Enthalpies= -56.519522
Sum of electronic and thermal Free Energies= -56.542406

E (Thermal) KCal/Mol CV Cal/Mol-Kelvin S Cal/Mol-Kelvin
23.407 6.325 48.162

Cartesian coordinates

Atom X Y Z
N 1.738631 0.297904 0.000000
H 2.135986 -0.639399 0.000000
H 2.136359 0.766353 0.811607
H 2.136359 0.766353 -0.811607

OMe-H

Molecular formula: CH4O
Charge = 0 Multiplicity = 1
Hartree/particle
Thermal correction to Energy= 0.054706
Thermal correction to Enthalpy= 0.055650
Thermal correction to Gibbs Free Energy= 0.028675
Sum of electronic and zero-point Energies= -115.672560
Sum of electronic and thermal Energies= -115.669258
Sum of electronic and thermal Enthalpies= -115.668314
Sum of electronic and thermal Free Energies= -115.695289

E (Thermal) KCal/Mol CV Cal/Mol-Kelvin S Cal/Mol-Kelvin
34.328 8.709 56.774

Cartesian coordinates

Atom X Y Z
C -0.661664 -0.019442 0.000000
H -1.082201 0.989439 -0.000001
H -1.038268 -0.542915 -0.891823
H -1.038268 -0.542914 0.891823
O 0.749367 0.122045 0.000000
H 1.133788 -0.763319 0.000000

OH-H

Molecular formula: H2O
Charge = 0 Multiplicity = 1
Hartree/particle
Thermal correction to Energy= 0.024204
Thermal correction to Enthalpy= 0.025148
Thermal correction to Gibbs Free Energy= 0.003058
Sum of electronic and zero-point Energies= -76.398368
Sum of electronic and thermal Energies= -76.395533
Sum of electronic and thermal Enthalpies= -76.394589
Sum of electronic and thermal Free Energies= -76.416679

E (Thermal) KCal/Mol CV Cal/Mol-Kelvin S Cal/Mol-Kelvin
15.188 6.003 46.493

Cartesian coordinates

Atom X Y Z
O -0.022803 0.000000 -0.016114
H 0.025652 0.000000 0.947893
H 0.902087 0.000000 -0.292233

Me-H

Molecular formula: CH4
Charge = 0 Multiplicity = 1
Hartree/particle
Thermal correction to Energy= 0.047885
Thermal correction to Enthalpy= 0.048829
Thermal correction to Gibbs Free Energy= 0.025352
Sum of electronic and zero-point Energies= -40.478995
Sum of electronic and thermal Energies= -40.476129
Sum of electronic and thermal Enthalpies= -40.475185
Sum of electronic and thermal Free Energies= -40.498662

E (Thermal) KCal/Mol CV Cal/Mol-Kelvin S Cal/Mol-Kelvin
30.048 6.444 49.411

Cartesian coordinates

Atom X Y Z
C -1.849376 0.360502 -0.024210
H -1.485388 -0.669029 -0.024210
H -1.485370 0.875259 0.867385
H -1.485370 0.875259 -0.915804
H -2.941352 0.360515 -0.024210

***i*-Pr-H**

Molecular formula: C3H8
Charge = 0 Multiplicity = 1
Hartree/particle
Thermal correction to Energy= 0.108198
Thermal correction to Enthalpy= 0.109142
Thermal correction to Gibbs Free Energy= 0.078019
Sum of electronic and zero-point Energies= -119.051677
Sum of electronic and thermal Energies= -119.047155
Sum of electronic and thermal Enthalpies= -119.046211
Sum of electronic and thermal Free Energies= -119.077334

E (Thermal) KCal/Mol CV Cal/Mol-Kelvin S Cal/Mol-Kelvin
67.895 14.756 65.504

Cartesian coordinates
Atom X Y Z
C -1.052150 -0.567362 0.021602
H -0.670815 -1.596369 -0.007419
H -0.670610 -0.077848 -0.883883
C -0.497238 0.146176 1.258108
H 0.597913 0.150175 1.263954
H -0.834021 1.188658 1.297235
H -0.833195 -0.342209 2.180229
C -2.583118 -0.590875 -0.019382
H -2.993978 0.425395 -0.027542
H -2.954739 -1.106904 -0.911022
H -2.993991 -1.104246 0.857739

F-H

Molecular formula: HF
Charge = 0 Multiplicity = 1
Hartree/particle
Thermal correction to Energy= 0.011666
Thermal correction to Enthalpy= 0.012611
Thermal correction to Gibbs Free Energy= -0.007107
Sum of electronic and zero-point Energies= -100.418156
Sum of electronic and thermal Energies= -100.415795
Sum of electronic and thermal Enthalpies= -100.414851
Sum of electronic and thermal Free Energies= -100.434569

E (Thermal) KCal/Mol CV Cal/Mol-Kelvin S Cal/Mol-Kelvin

7.321 4.968 41.499

Cartesian coordinates
Atom X Y Z
F 0.000000 0.000000 0.092540
H 0.000000 0.000000 -0.832859

COOH-H

Molecular formula: CH2O2
Charge = 0 Multiplicity = 1
Hartree/particle
Thermal correction to Energy= 0.036839
Thermal correction to Enthalpy= 0.037784
Thermal correction to Gibbs Free Energy= 0.009512
Sum of electronic and zero-point Energies= -189.720503
Sum of electronic and thermal Energies= -189.717263
Sum of electronic and thermal Enthalpies= -189.716319
Sum of electronic and thermal Free Energies= -189.744591

E (Thermal) KCal/Mol CV Cal/Mol-Kelvin S Cal/Mol-Kelvin
23.117 8.911 59.502

Cartesian coordinates

Atom X Y Z
C -0.130515 0.361852 -0.000032
H -0.034904 1.465190 0.000011
O -1.178538 -0.219002 0.000019
O 1.058604 -0.281510 0.000003
H 1.777465 0.367795 0.000009

CF_3-H

Molecular formula: CHF3
Charge = 0 Multiplicity = 1
Hartree/particle
Thermal correction to Energy= 0.028929
Thermal correction to Enthalpy= 0.029873
Thermal correction to Gibbs Free Energy= 0.000362
Sum of electronic and zero-point Energies= -338.201920
Sum of electronic and thermal Energies= -338.198450
Sum of electronic and thermal Enthalpies= -338.197506
Sum of electronic and thermal Free Energies= -338.227017

E (Thermal) KCal/Mol CV Cal/Mol-Kelvin S Cal/Mol-Kelvin
18.153 10.185 62.111

Cartesian coordinates

Atom X Y Z
C 0.000000 0.000000 0.339252
H 0.000000 0.000000 1.433090
F 0.000000 1.258016 -0.128467
F 1.089474 -0.629008 -0.128467
F -1.089474 -0.629008 -0.128467

CHO-H

Molecular formula: CH2O
Charge = 0 Multiplicity = 1
Hartree/particle
Thermal correction to Energy= 0.029586
Thermal correction to Enthalpy= 0.030530
Thermal correction to Gibbs Free Energy= 0.005055
Sum of electronic and zero-point Energies= -114.476480
Sum of electronic and thermal Energies= -114.473614
Sum of electronic and thermal Enthalpies= -114.472669
Sum of electronic and thermal Free Energies= -114.498144

E (Thermal) KCal/Mol CV Cal/Mol-Kelvin S Cal/Mol-Kelvin
18.565 6.411 53.616

Cartesian coordinates

Atom X Y Z
C 0.000006 0.529016 0.000000
H -0.937820 1.123848 0.000000
H 0.937738 1.124007 0.000000
O 0.000006 -0.677744 0.000000

CN-H

Molecular formula: CHN
Charge = 0 Multiplicity = 1
Hartree/particle
Thermal correction to Energy= 0.019005
Thermal correction to Enthalpy= 0.019949
Thermal correction to Gibbs Free Energy= -0.002906
Sum of electronic and zero-point Energies= -93.408114
Sum of electronic and thermal Energies= -93.405576
Sum of electronic and thermal Enthalpies= -93.404632
Sum of electronic and thermal Free Energies= -93.427487

E (Thermal) KCal/Mol CV Cal/Mol-Kelvin S Cal/Mol-Kelvin
11.926 6.388 48.103

Cartesian coordinates

Atom X Y Z
C 0.000000 0.000000 -0.502180
H 0.000000 0.000000 -1.570809
N 0.000000 0.000000 0.654841

NO_2-H

Molecular formula: HNO2
Charge = 0 Multiplicity = 1
Hartree/particle

Thermal correction to Energy= 0.024930
Thermal correction to Enthalpy= 0.025874
Thermal correction to Gibbs Free Energy= -0.001180
Sum of electronic and zero-point Energies= -205.667710
Sum of electronic and thermal Energies= -205.664750
Sum of electronic and thermal Enthalpies= -205.663806
Sum of electronic and thermal Free Energies= -205.690860

E (Thermal) KCal/Mol CV Cal/Mol-Kelvin S Cal/Mol-Kelvin
15.644 7.173 56.941
Cartesian coordinates

Atom X Y Z
O 0.000000 1.100354 -0.220639
O 0.000000 -1.100354 -0.220639
N 0.000000 0.000000 0.310793
H 0.000000 0.000000 1.354666

H_2

Molecular formula: H2
Charge = 0 Multiplicity = 1
Hartree/particle
Thermal correction to Energy= 0.012534
Thermal correction to Enthalpy= 0.013478
Thermal correction to Gibbs Free Energy= -0.001314
Sum of electronic and zero-point Energies= -1.168366
Sum of electronic and thermal Energies= -1.166005
Sum of electronic and thermal Enthalpies= -1.165061
Sum of electronic and thermal Free Energies= -1.179853

E (Thermal) KCal/Mol CV Cal/Mol-Kelvin S Cal/Mol-Kelvin
7.865 4.968 31.132
Cartesian coordinates

Atom X Y Z
H 0.000000 0.000000 0.371396
H 0.000000 0.000000 -0.371396
